# Flavonoids as dual-target inhibitors against α-glucosidase and α-amylase: a systematic review of in vitro studies

**DOI:** 10.1007/s13659-023-00424-w

**Published:** 2024-01-08

**Authors:** Thua-Phong Lam, Ngoc-Vi Nguyen Tran, Long-Hung Dinh Pham, Nghia Vo-Trong Lai, Bao-Tran Ngoc Dang, Ngoc-Lam Nguyen Truong, Song-Ky Nguyen-Vo, Thuy-Linh Hoang, Tan Thanh Mai, Thanh-Dao Tran

**Affiliations:** 1 Faculty of Pharmacy, University of Medicine and Pharmacy at Ho Chi Minh City, 700000 Ho Chi Minh City, Vietnam; 2 Faculty of Pharmacy, Uppsala University, 75105 Uppsala, Sweden; 3 Department of Chemistry, Imperial College London, London, W12 0BZ UK; 4 California Northstate University College of Pharmacy, California, 95757 USA

**Keywords:** Systematic review, Flavonoids, Dual-target, Glucosidase, Amylase, PRISMA, SAR

## Abstract

Diabetes mellitus remains a major global health issue, and great attention is directed at natural therapeutics. This systematic review aimed to assess the potential of flavonoids as antidiabetic agents by investigating their inhibitory effects on α-glucosidase and α-amylase, two key enzymes involved in starch digestion. Six scientific databases (PubMed, Virtual Health Library, EMBASE, SCOPUS, Web of Science, and WHO Global Index Medicus) were searched until August 21, 2022, for in vitro studies reporting IC_50_ values of purified flavonoids on α-amylase and α-glucosidase, along with corresponding data for acarbose as a positive control. A total of 339 eligible articles were analyzed, resulting in the retrieval of 1643 flavonoid structures. These structures were rigorously standardized and curated, yielding 974 unique compounds, among which 177 flavonoids exhibited inhibition of both α-glucosidase and α-amylase are presented. Quality assessment utilizing a modified CONSORT checklist and structure–activity relationship (SAR) analysis were performed, revealing crucial features for the simultaneous inhibition of flavonoids against both enzymes. Moreover, the review also addressed several limitations in the current research landscape and proposed potential solutions. The curated datasets are available online at https://github.com/MedChemUMP/FDIGA.

**Graphical Abstract:**

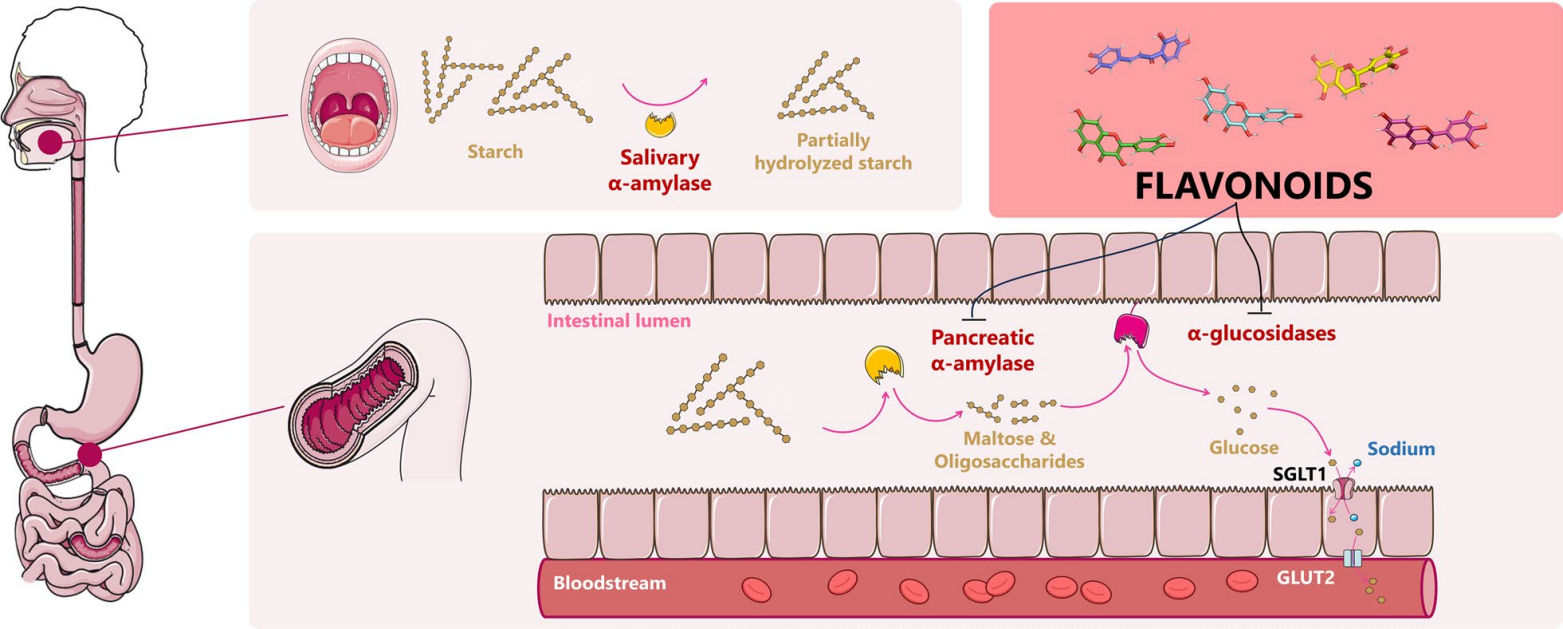

**Supplementary Information:**

The online version contains supplementary material available at 10.1007/s13659-023-00424-w.

## Introduction

Diabetes mellitus (DM) is a chronic condition imposing a significant burden on society due to its severe complications and enormous expenditure spent on long-term treatment [1]. According to the International Diabetes Federation report in 2021 [1], DM has been attributed to an estimated 7 million deaths worldwide. Diabetic patients are also prone to life-threatening microvascular and macrovascular complications with subsequent multiple organ dysfunction. These abnormalities stem from inadequate insulin secretion and/or insulin resistance, characterized by the persistent elevation of blood glucose [2]. Type II diabetes mellitus (T2DM) is associated with insulin resistance, necessitating constant medical intervention [2]. In particular, myriads of therapeutic mechanisms have been discovered, some of which aim to stimulate insulin secretion, recover insulin sensitivity, prevent the absorption of carbohydrates, or decrease gluconeogenesis [3]. Currently, available anti-diabetic therapeutics include sulfonylureas, meglitinides, thiazolidinediones, biguanides, dipeptidyl peptidase-IV inhibitors (DPP-4i), glucagon-like-peptide-1 receptor agonist (GLP1-RA), sodium-glucose transporter-2 inhibitors (SGLT-2i), and α-glucosidase inhibitors [4, 5].

One of the key features of patients with T2DM is postprandial hyperglycemia, which is inextricably associated with starch hydrolysis. The starch digestion process commences with the breakdown of polysaccharides into linear and branched malto-oligosaccharides by salivary α-amylase (EC 3.2.1.1). The degradation is intermitted in the stomach and continues in the small intestine with the pancreatic α-amylase, yielding maltose, maltotriose, or smaller oligosaccharides. Following this, four different α-glucosidases, maltase (synonym: α-glucosidase, EC 3.2.1.20), glucoamylase (EC 3.2.1.3), sucrase (EC 3.2.1.48), and isomaltase (EC 3.2.1.10), are responsible for further degradation. Human bodies simultaneously produce these four enzymes as two multifunctional complexes, maltase-glucoamylase (MGAM) and sucrase-isomaltase (SI), which cleave α-glycosidic bonds between disaccharides or oligosaccharides to release free monosaccharides [6, 7]. The hydrolyzed monomers are then transported into intestinal epithelial cells via specific transporters, such as SGLT1 (for glucose and galactose) or GLUT5 (for fructose), before entering the bloodstream through facilitated diffusion via the GLUT2 transporter in the basolateral membrane [8]. The schematic illustration of starch metabolism is depicted in Additional file 1: Fig. S1.

The concept of attenuating the glucose uptake process to maintain blood glucose levels within normal range has become a promising strategy for managing T2DM [9]. Delaying carbohydrate digestion can decrease the portion of glucose entering blood vessels, thereby reducing postprandial glucose levels. Based on the established mode of action, in 1995, the first α-glucosidase inhibitor (AGI) named acarbose was developed and approved by FDA, followed by miglitol in 1996 [3]. However, the use of AGI agents in clinical practice is accompanied by several gastrointestinal side effects, such as flatulence, abdominal distention, and diarrhea [10]. The underlying reason for these events is the substantial inhibitory effects against pancreatic α-amylases compared to α-glucosidases. As a result, a considerable amount of complex carbohydrates and starch remain intact throughout the gastrointestinal tract. They are eventually degraded by bacterial enzymes in the colon, resulting in gas and bloating. Hence, in the search for safer and more effective AGI agents, it should be noted that compounds having modest α-amylase inhibitory effects are preferable [10].

Flavonoids are natural phenolic compounds characterized by a C6-C3-C6 skeleton consisting of two benzene rings (A and B rings) linked through a three-carbon bridge. In most cases, this three-carbon system forms a heterocyclic pyran ring (C ring). These compounds are well-known for their diverse health-promoting properties, including anti-inflammatory [11], anti-oxidative [12], anti-infective [13], and anti-obesity effects [14]. Notably, several studies have highlighted the potential of flavonoids as anti-diabetic agents due to their strong inhibition of α-glucosidase and moderate inhibition of α-amylase, making them promising candidates for the development of anti-diabetic drugs with minimal gastrointestinal side effects [15–17].

Over the years, the α-amylase and α-glucosidase inhibitory effects of flavonoids have been recorded in numerous reviews [18–23]. Nevertheless, to the best of our knowledge, there is no systematic review about the concurrent inhibition of flavonoids against α-amylase and α-glucosidase that has been reported until this project commenced (August 2022). Therefore, this systematic review aims to present the current evidence supporting the simultaneous inhibition of flavonoids against the two starch-digestive enzymes and propose a structure–activity relationship (SAR) analysis that could be useful for developing more effective and safer anti-diabetic therapeutic agents in the future.

## Results

### Systematic search and study selection

The initial search resulted in an accumulation of 9,694 records from the six databases. Following the duplicate removal process, the acquired literature was filtered based on their language and article type characteristics by the Zotero and Rayyan programs. The results are 4,408 records that would undergo the title and abstract screening process. We obtained 570 full-text articles and further evaluated them for inclusion eligibility. 231 reports were excluded, thus a total of 339 studies were included in the present review. These studies went through a data extraction process, and flavonoids were grouped based on their SMILES and systematically scrutinized for their capacity to inhibit the two digestive enzymes. Finally, 177 flavonoids, which have concurrent IC_50_ values against both α-glucosidase and α-amylase, are presented in the current review. The PRISMA flowchart of this systematic review could be found in Additional file 1: Fig. S2.

### Study characteristics

A total of 339 research articles, involving at least 1,643 flavonoid structures, were published from 1998 to 2022. Of these, 51 studies conducting both α-glucosidase and α-amylase inhibition assays were recorded. Thirty-six studies involved α-amylase inhibition assay, whereas 252 remaining studies involved only α-glucosidase inhibition assay. Among the included studies, various flavonoid sources have been documented. As anticipated, most flavonoids were derived from natural sources, accounting for 76% of the included studies. Different sources of the enzymes used in the literature were also observed. Yeast α-glucosidase, especially one derived from *Saccharomyces cerevisiae*, is the most common source of α-glucosidase used in the literature with the proportion standing at over 70 percent (190 over 303 studies). On the other side, porcine pancreatic α-amylase is the most popular source used in the α-amylase involved assay, followed by enzymes from human sources such as human salivary and human pancreatic ones.

A variety of assay methods used in the literature were also documented and characterized based on the substrate and the principle of detection used in the studies. For the α-glucosidase inhibition assay, a chromogenic method that employed the use of a synthetic substrate *p*-nitrophenyl-α-*D*-glucopyranoside (pNPG) is the most popular method used to evaluate the inhibitory activity of the flavonoids. In this methodology, the pNPG substrate will be hydrolyzed by the enzyme and therefore release a colorimetric *p*-nitrophenol compound. The amount of released *p*-nitrophenol will be measured using the absorbance at around 405 nm. Other assay methods were employed to assess the inhibitory ability of the flavonoids against α-glucosidase such as the enzymatic method employing glucose oxidase. In the α-amylase inhibition assay, six different assay principles were recorded, with the most cited technique being the reducing sugar method (51 over 87 studies). In this approach, the liberated sugars from the starch will act as reducing agents and oxidize the 3,5-dinitrosalicylic acid (DNSA) molecule to yield the deep orange solution which absorbs light strongly at 540 nm [24]. Other assay principles include the chromogenic method (23/87), turbidimetric method (3/87), iodine–starch method (8/87), and enzymatic method (2/87). The detailed characteristics of the included studies are available in Additional file 1: Table S4.

### Main results

Although our database initially included 1,643 flavonoid structures, only 177 compounds were found to be able to concurrently inhibit both α-glucosidase and α-amylase after merging the duplicates. The original database of 177 presented compounds and the full dataset of 1,643 entries are available online at https://github.com/MedChemUMP/FDIGA. Unlike other reviews which take a study-by-study approach [18, 22]. we used a structure-based approach to summarize results from various studies reporting the same chemical structure. We believe that this approach will provide audiences with a more comprehensive evaluation of compound potency compared to acarbose. To limit variations between studies, we used the median and interquartile range of pIC_50_ values and stated inhibitory mechanisms if reported by any of the studies.

#### Flavans and flavan derivatives

##### Flavans

**Fig. 1:**
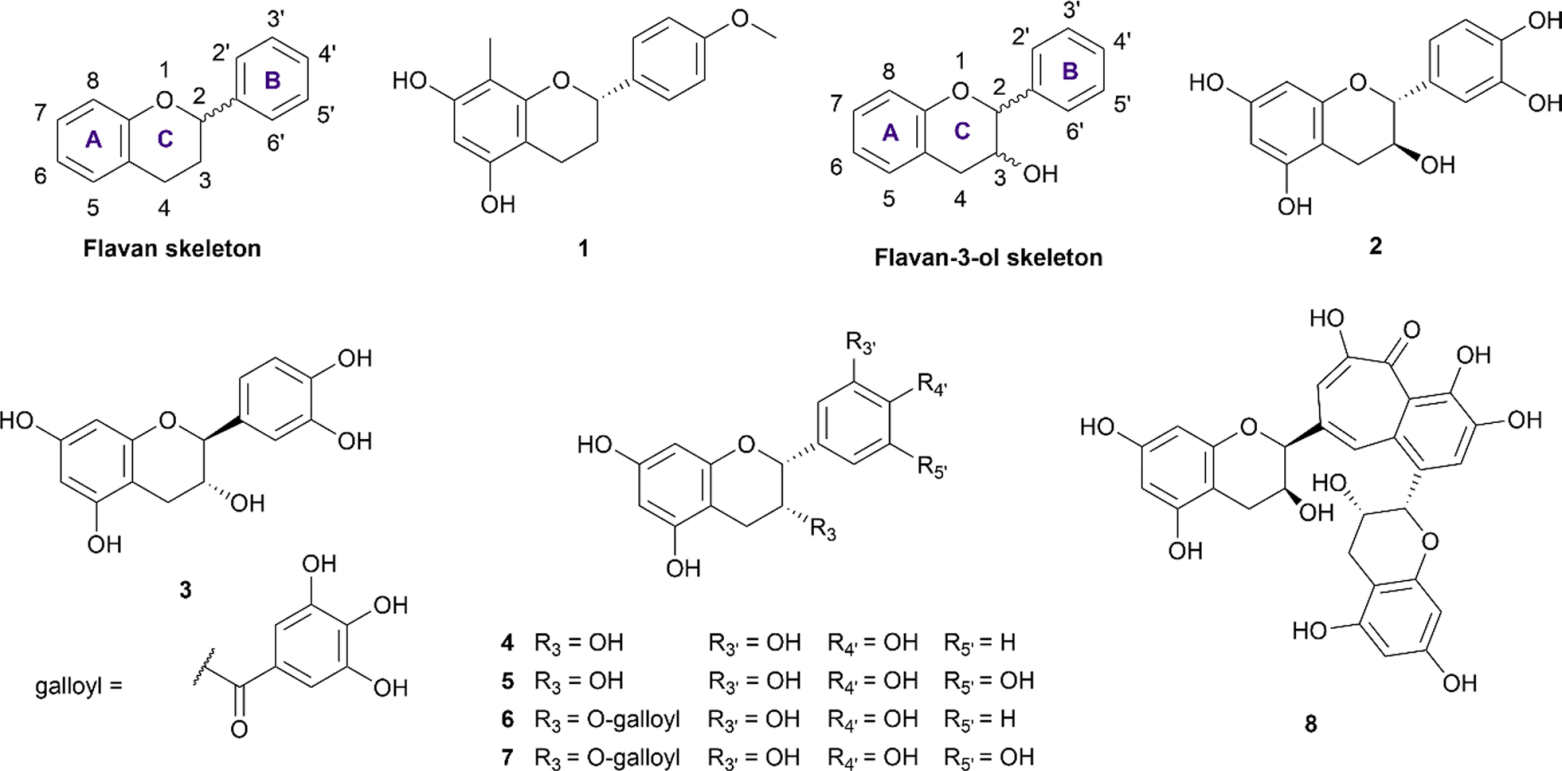
Chemical structures of retrieved flavan and flavan-3-ol derivatives

Flavans are widely distributed in nature and originate from the reduction of flavanone. This flavonoid class is characterized by the absence of a double bond between the C_2_ and C_3_ positions. However, the most basic flavan skeleton (Fig. 1) is not usually observed in plant tissues as the C_3_ or C_4_ positions are usually hydroxylated or ketonized to form other flavan subclasses. In the present review, we only record (2*S*)-4′-hydroxy-5,7-dimethoxy-8-methylflavan (**1**), a flavan extracted from the plant of *Dracaena angustifolia*, as a dual-target inhibitor. This compound was able to inhibit α-glucosidase and α-amylase at the concentration of 370 µM and 6.03 mM, respectively [25]. In comparison with acarbose in the same condition, compound **1** exhibited stronger inhibition on α-glucosidase but weaker in the α-amylase inhibition assay.

##### Flavanols (catechins)

Flavanol inherits the flavan scaffold where the C_3_ and/or C_4_ position is hydroxylated to form three different subclasses: flavan-3-ol, flavan-4-ol, flavan-3,4-diol, and the most well-known example of which being catechin. In the current review, seven flavan-3-ol structures (**2–8**) were recorded to have concurrent anti-α-glucosidase and anti-α-amylase activity (Fig. 1). Based on the two chiral centres C_2_ and C_3_, the flavanols can exist in four different stereoisomers namely (*2R,3S*), (*2S,3R*), (*2R,3R*), (*2S,3S*). Additional file 1: Table S6 summarizes the potency of included flavan and flavanol derivatives against α-glucosidase and α-amylase.

(+)-Catechin (**2**) is the most well-studied flavan-3-ol with the IC_50_ values against the α-glucosidase and α-amylase ranging from 1.12 to 1276.51 µM and 0.31 µM to at most 70.87 mM, respectively. Considering α-glucosidase inhibition, the current evidence suggests that this compound can inhibit the enzyme as potently as the standard drug in a competitive manner [26]. On the other hand, this compound exhibits two to three times weaker inhibition against the α-amylase than acarbose [27–31]. The wide range of reported results could stem from the various origin of the enzyme and assay protocol. On the contrary, (−)-catechin (**3**), a *3R* isomer of **2**, despite achieving low IC_50_ against both enzymes, compares far less favorable than that of the standard drug acarbose, indicating a weaker inhibitory activity of this compound [32]. (−)-Epicatechin (**4**), another (*2R,3R*) isomer of **2,** has also been documented for its inhibitory activity against two digestive enzymes. Most studies suggest compound **4** to be a weak inhibitor, in comparison with acarbose in the same condition [33–40]. However, the results are still controversial, not only in the reported IC_50_ value but also in the underlying mechanism. In a study reported by Giang et al. [41], this compound was able to inhibit ten times better than acarbose in both inhibition assays. However, this is the only study that suggests **4** as a strong inhibitor against α-amylase. Thus, further evidence is needed to confirm the potency of this compound. (−)-Epigallocatechin (**5**) was reported in three studies [35, 42, 43], all of which consented that this compound was able to inhibit the two enzymes but with weaker activity in comparison to acarbose.

In plant tissues, flavanols are usually esterized with gallic acid to form their gallate derivatives. In this review, two gallate derivatives, namely (−)-epicatechin gallate (ECG, **6**) and (−)-epigallocatechin gallate (EGCG, **7**) are reported to have concurrent inhibitory activity against α-glucosidase and α-amylase. Together with theaflavin (**8**), these two compounds are famous for their antioxidant, anti-inflammatory, anti-diabetic activity, and abundance in tea tissue (*Camellia sinensis*) [44]. The current evidence suggests that both **6** and **7** can inhibit α-glucosidase stronger than acarbose in the same condition. On the other hand, these two compounds also exhibit weak inhibition against α-amylase, indicating their potential as promising drug candidates which can minimize the undesirable effects of acarbose. The inhibitory mechanisms of the two compounds were reported to be non-competitive against α-glucosidase and in a mixed, competitive, or non-competitive manner towards α-amylase [45–48]. However, the inhibition pattern of theaflavin (**8**) is the opposite. This compound was reported to be a weak α-glucosidase inhibitor but a strong α-amylase inhibitor with the IC_50_ of 16.17 µM and 0.46 µM, respectively [32].

##### Flavanones

**Fig. 2:**
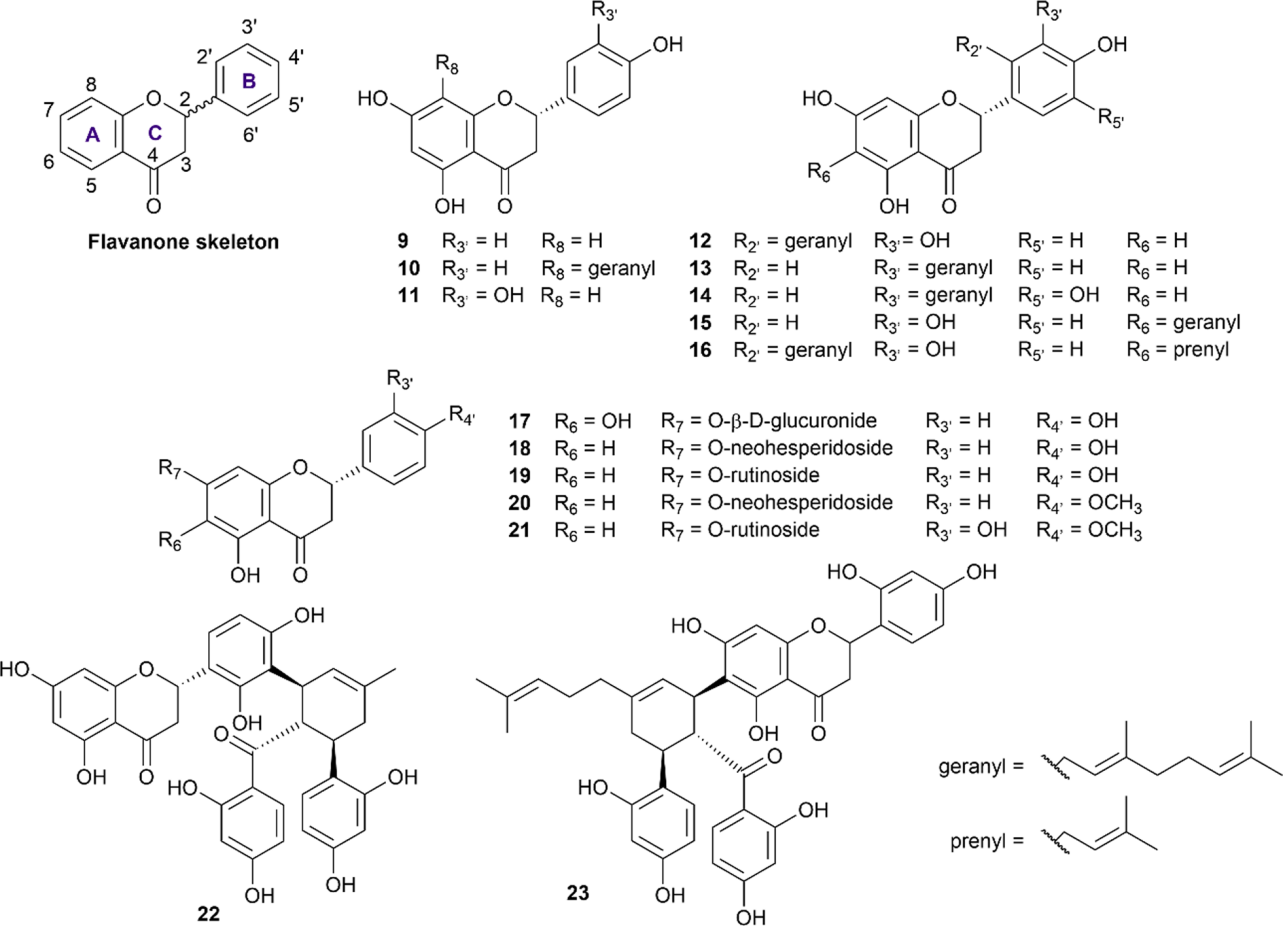
Chemical structures of retrieved flavanone derivatives

Flavanones (also called dihydroflavones) are characterized by the absence of the double bond between C_2_ and C_3_ in the C-ring and the presence of the ketone group in C_4_ (Fig. 2). In our review, 15 flavanone structures (**9**–**23**) that have sufficient evidence for their abilities to inhibit both α-glucosidase and α-amylase were included and summarized in Additional file 1: Table S7.

Naringenin (**9**) is the most well-studied flavanone with 16 studies employing this compound in their inhibition assay [33, 41, 49–62]. Concerning the α-glucosidase inhibition, naringenin was recorded to exhibit stronger inhibitory activity than acarbose in the same condition in the majority of the reported results. For α-amylase inhibition, this compound also exhibited strong inhibition against this enzyme with the IC_50_ values ranging from 6.20 to at most 121.50 µM [33, 41, 51]. The geranylation of naringenin at the C_8_ position forming 8-geranylnaringenin (**10**) was reported to enhance the inhibitory activity of this compound against both enzymes.^5^ Eriodictyol (**11**), a C_3’_ hydroxyl derivative of naringenin, was also reported in the literature for its ability to inhibit both enzymes [63–67]. In comparison with acarbose, this compound was a stronger inhibitor, regardless of the enzyme type employed. Propolin D (**12**), Propolin H (**13**), Propolin F (**14**), Propolin C (**15**), and Propolin G (**16**) are five geranylated flavanone aglycones derived from propolis of the Australian honeybees (*Apis mellifera*) reported by Uddin et al. [51]. In his study, the geranylation at either C_2’_ or C_3’_ position significantly enhanced the inhibitory activity of flavanone compounds against both enzymes. However, the geranylation or prenylation at the C_6_ position reduced such activity against α-glucosidase but had less impact on the α-amylase inhibition manner.

Isocarthamidin-7-*O*-glucuronide (**17**) is a flavanone glycoside derived from the shoot of *Scutellaria baicalensis* reported by Li et al. [68]. This compound was a weak inhibitor against both α-glucosidase and α-amylase, in comparison with acarbose, with the respective values being 4.6 mM and 6.3 mM [68]. Different glycoside derivatives of naringenin were also recorded in the literature, with naringin (**18**) and narirutin (**19**) being the two representatives. Overall, naringin (**18**) was recorded to exhibit strong inhibition against both enzymes in the majority of studies involved [17, 60, 61, 69–71]. However, in two studies reported by Kong et al. [70] and Zhang et al. [60], this compound was reported to inhibit α-glucosidase not as well as acarbose in the same condition, as the IC_50_ values stood at 27.2 mM and 22.1 mM, respectively. Therefore, further evidence is needed to confirm the potency of this compound. Narirutin (**19**), a 7-*O*-rutinoside of naringenin (**9**), showed comparable inhibitory activity to acarbose against α-glucosidase but far stronger inhibitory activity against α-amylase [17, 72]. Likewise, poncirin (**20**) also exhibited similar activity against α-glucosidase but was reported to be stronger than acarbose in the α-amylase inhibition assay [17, 69]. Hesperidin (**21**), a flavanone glycoside that has long been known for its anti-atherogenic and venous protection activity [73], is also included in our review. In the literature, hesperidin was reported to be a potent antidiabetic therapeutic agent, as it exhibited strong inhibitions against both α-glucosidase and α-amylase, reported by at least four studies [17, 55, 69, 71]. Kuwanon L (**22**) and Sanggenon G (**23**) are two flavanone compounds derived from the root bark of *Morus alba* [74]. In their study, Zhao et al. described these two compounds as strong α-glucosidase inhibitors and moderate α-amylase inhibitors [74].

##### Flavanonols

**Fig. 3:**
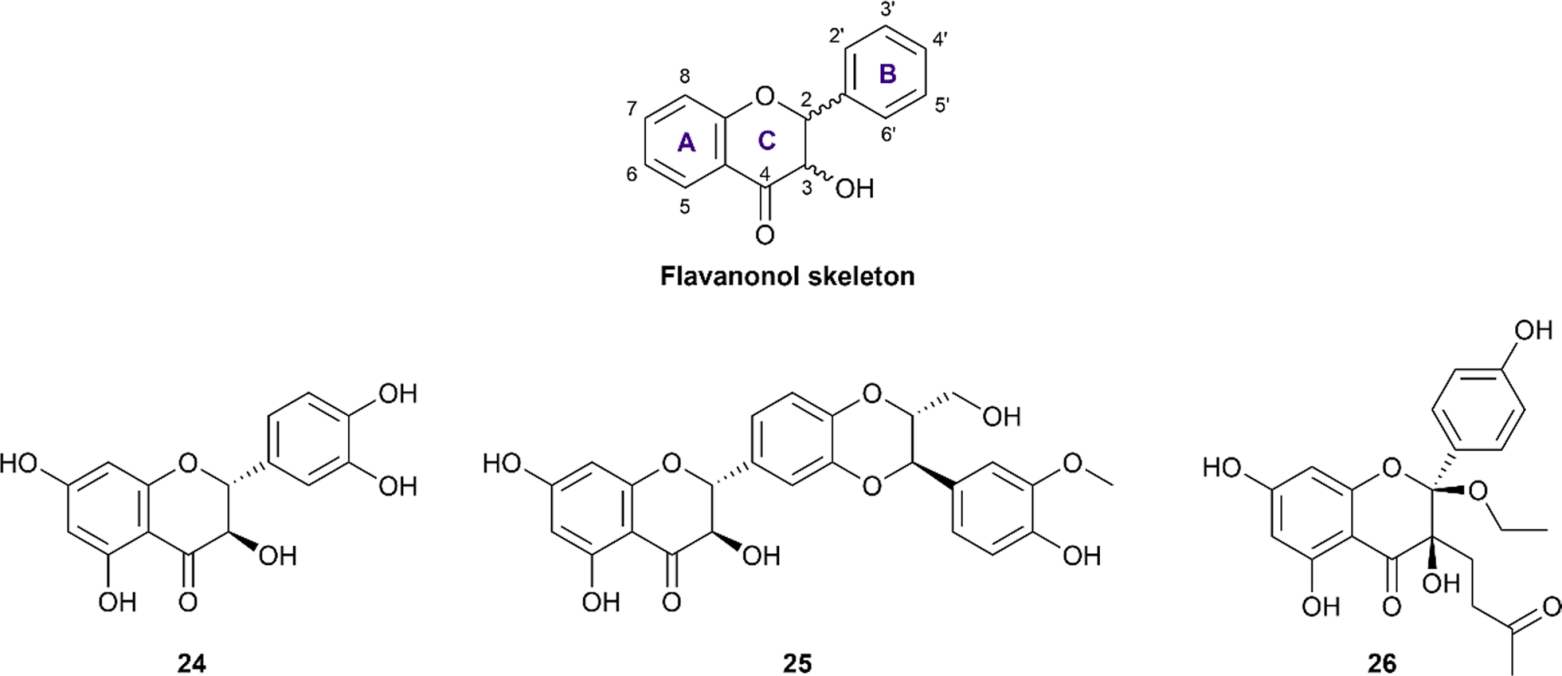
Chemical structures of retrieved flavanonol derivatives

Flavanonol (also referred to as 2,3-dihydroflavonol) is a small flavonoid subclass resulting from the hydroxylation at the C_3_ position of flavanone structures (Fig. 3). In this review, three representatives, namely taxifolin (**24**), silibinin (**25**), and dysosmaflavanone (**26**) are included and their pIC_50_ are presented in Additional file 1: Table S8.

Taxifolin (also called dihydroquercetin, **24**) was reported in six studies for its potency to inhibit both α-glucosidase and α-amylase [65, 75–79]. The results showed that this compound exhibited comparable inhibitory activity to that of acarbose in the same condition while inhibiting α-amylase more weakly. Concerning the underlying mechanism, Su et al. reported that this flavonoid was able to inhibit both enzymes competitively [78]. Silibinin (**25**), a flavanonol-lignan hybrid, was reported for its ability to inhibit two carbohydrate-hydrolyzing enzymes in a study conducted by Yang et al.[80]. In this study, silibinin exhibited strong inhibition against α-glucosidase, yet moderate inhibition against α-amylase, in comparison with acarbose. The mechanism of a non-competitive manner was also reported in his study. Additionally, dysosmaflavanone (**26**), a deoxygenated flavanonol from *Dysosma difformis,* is also included in this review and has been recorded to exhibit weak inhibition against both enzymes in one study [81].

#### Flavones

**Fig. 4:**
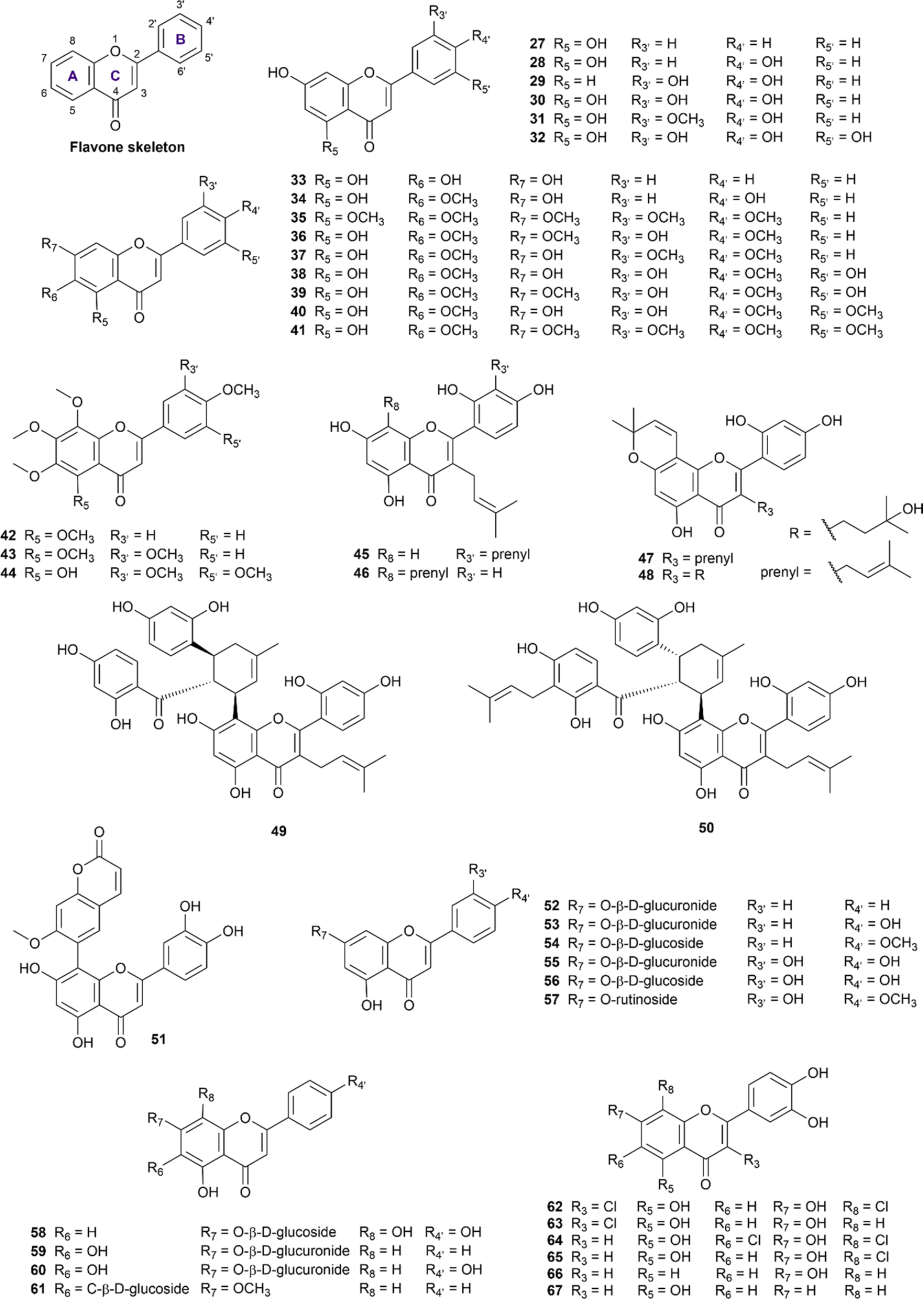
Chemical structures of retrieved flavone derivatives

The flavone scaffold constituted a large proportion of our included dataset, with 40 structures (numbered **27–67**) recorded of activity on both enzymes of interest (Fig. 4). Flavone compounds are distinguished from flavanones by the presence of the C_2_ = C_3_ double bond, thus constructing the chromene core (4*H*-1-benzopyran). Hydroxy and methoxy substituents are abundant in this sub-class of flavonoids and vary their positions in all three rings A, B, and C. A summary of flavone inhibitory potency against α-glucosidase and α-amylase is available in Additional file 1: Table S9.

From our curated data, chrysin (**27**), apigenin (**28**), and luteolin (**30**) were widely researched flavones, of which apigenin (**28**) was the subject of 30 studies [33, 34, 53, 54, 59, 65, 68, 82–103]. Despite various origins from numerous plants, there is a general consensus regarding apigenin’s activity as a more potent α-glucosidase inhibitor than acarbose but a weaker α-amylase antagonist, making apigenin a potential candidate for nutritional and pharmaceutical purposes. However, it should also be noted that 9 out of 30 studies [33, 34, 53, 65, 82, 84, 88, 91, 93] disregard apigenin’s stronger inhibition on α-glucosidase than acarbose, but there was no dispute over the activity on α-amylase. Apigenin’s IC_50_ on α-glucosidase was reported in a large range from 9.04 µM to 34.3 mM but these results should be taken into account in relation to the acarbose reference and experimental protocols.

Luteolin (**30**) exhibits a similar inhibition profile on both enzymes but to a lesser extent of consent on α-glucosidase. Eighteen out of 31 recorded findings [52, 63, 86, 87, 89, 92, 94, 96, 99, 100, 103–110] showed stronger activity on α-glucosidase than acarbose, in contrast with the remaining 13 studies [33, 53, 65, 82, 84, 88, 91, 111–116], making a 6:4 conflict. There was greater unity in the results of α-amylase inhibition, confirming a moderate but generally weaker activity than acarbose on α-amylase (IC_50_ ranging from 14.57 µM to 1 mM) [33, 82, 117, 118]. On a side note, 5-deoxyluteolin (**29**) is structurally related to luteolin (**30**) but is more limited in experimental data [33, 94, 116]. The currently available results suggest that the absence of the O_5_ oxygen atom decreased 5-deoxyluteolin’s activity on both enzymes compared to acarbose.

Chrysin (**27**) is next on the list of commonly researched flavones, with eight studies [68, 71, 103, 119–123] conducted on α-glucosidase and one studied on α-amylase [68]. The comparison of activity on α-glucosidase is inconsistent and equally divided between stronger [71, 103, 120, 121] and weaker inhibition than acarbose [68, 119, 122, 123]. Meanwhile, the only reported result on α-amylase showed weak inhibition at the IC_50_ of 1.771 µM [68].

Chrysoeriol (**31**) and tricetin (**32**) are closely related to luteolin but lesser researched flavones. Interestingly, there was great consensus in the three studies confirming chrysoeriol’s stronger activity on α-glucosidase than acarbose [49, 94, 124], and one result reporting on its generally weak inhibition of α-amylase at 1270 µM [125], making chrysoeriol (**31**) an attractive ligand for further optimization. Meanwhile, tricetin (**32**) was the subject of only one paper and was found to weakly inhibit both enzymes [82].

Proceeding to the next group of R_6_-substituted flavones, baicalein (**33**) is a major topic of ten studies [52, 65, 68, 94, 111, 113, 119, 122, 123, 126], two out of which reported IC_50_ on both α-glucosidase and α-amylase [68, 111]. The included literature commonly demonstrated this compound’s moderate but weaker inhibition on both enzymes than acarbose.

Hispidulin (**34**) is another intriguing case for antidiabetic profile optimization. In two studies [99, 127], hispidulin was shown to be a stronger inhibitor of α-glucosidase than acarbose in the same experimental conditions. As for α-amylase inhibition, hispidulin exhibited a weak activity with the IC_50_ at 30.08 µM, much higher than that of acarbose in the same settings [97].

Eupatorin (**36**) and eupatillin (**37**) are two flavones shown to strongly inhibit both α-glucosidase and α-amylase at nanomolar concentrations in a paper by Gulcin et al. [128]. These activity profiles were significantly stronger than acarbose in the same procedure and conditions [128], thus should be further studied for their molecular interactions with enzyme targets.

In another notable report [129], four methoxylated flavones at R_6_ and R_4’_ (**38, 39, 40, 41**) all showed an appealing profile with stronger glucosidase inhibition than acarbose (IC_50_ ranging from 49 to 77 µM) and a simultaneous weak amylase activity (IC_50_ varying from 120 to 338 µM). Analysis of the structure–activity relationship of R_4’_-methoxylated flavones can be examined to further enhance these profiles.

Other less data-abundant methoxylated flavones include tangeretin (**42**), nobiletin (**43**), and gardenin A (**44**), whose collective results were not yet conclusive about their activity potency. Promptly, it can be noted that nobiletin (**43**) inhibited α-glucosidase stronger than acarbose and moderately inhibited α-amylase [69, 130, 131]. Meanwhile, gardenin A (**44**) inhibited both enzymes with a much stronger activity than acarbose in the same conditions [128].

The next group of structural-related compounds includes kuwanon T (**45**), kuwanon C (**46**), morusin (**47**), morusinol (**48**), kuwanon G (moracenin B) (**49**), moracenin A (**50**), and 8-(7-methoxycoumarin-6-yl)luteolin (**51**). This group is characterized by the presence of a prenyl group at the R_3_ position (except for compound **51**), and more bulky side chains at the R_8_ position for compounds (**49**), (**50**), and (**51**). All seven compounds exhibited inhibitory profiles on both enzymes but noticeably compared much more favorably and selectively on α-glucosidase than acarbose [74, 132–134]. Once again, these gathered results facilitate future efforts in investigating these interesting scaffolds.

The next ten compounds (**52–61**) of this subclass feature flavone glycosides, most commonly glycosylated at the R_7_ oxygen. Of these glycosides, luteolin-7-*O*-β-D-glucoside (**56**) is the most well-researched with five studies [65, 89, 99, 110, 135], outlining a comparable activity to acarbose on α-glucosidase and a weaker inhibition on α-amylase [135]. Relatedly, luteolin-7-*O*-β-D-glucuronide (**55**) and diosmin (**57**) all displayed equivalent profiles to acarbose on both enzymes [135, 136]. On a side note, apigenin-7-*O*-β-D-glucuronide (**53**) and baicalin (**59**)’s results on α-glucosidase vary significantly between studies (ranging from 13.63 µM to 1.217 mM and 36.3 µM to 1.324 mM, respectively) [33, 68, 113, 119], thus careful consideration is recommended when interpreting these results. Meanwhile, swertisin (**61**) is one of the uncommon flavone C-glycosides at the R_6_ position and was found by two separate studies to inhibit α-glucosidase more strongly than acarbose [137] while maintaining a negligible activity on α-amylase (IC_50_ at 4.245 mM) [138]. This interesting structure and attractive activity profile may hence motivate future molecular interaction studies.

The last six compounds (**62–67**) are flavone derivatives that were synthesized and evaluated for their simultaneous inhibition against α-glucosidase and α-amylase by Proença et al. in two consecutive publications [94, 139]. Overall, these compounds exhibited strong inhibition against the α-glucosidase and moderate inhibition against the α-amylase, in comparison with acarbose in the same condition.

#### Flavonols

**Fig. 5:**
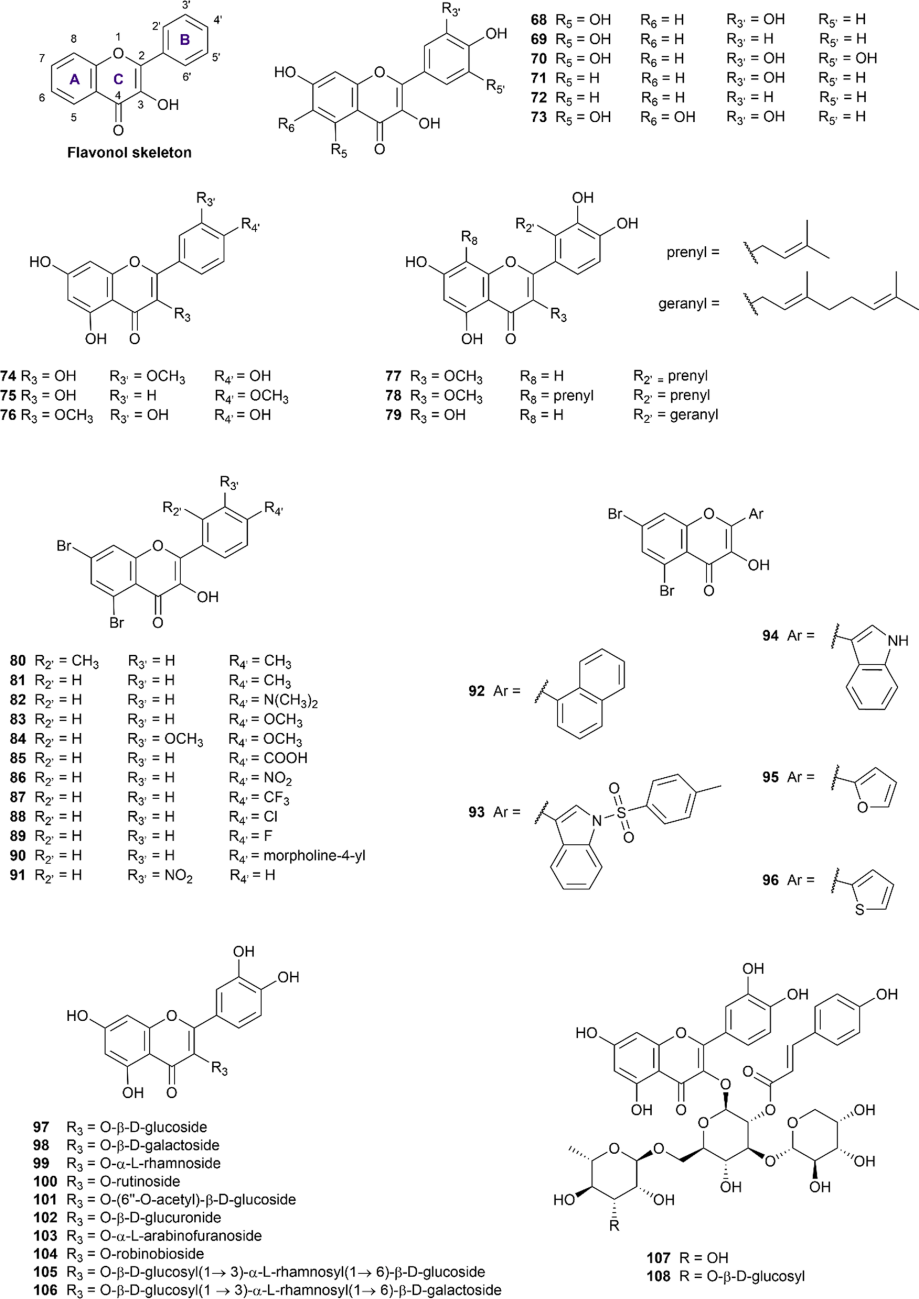

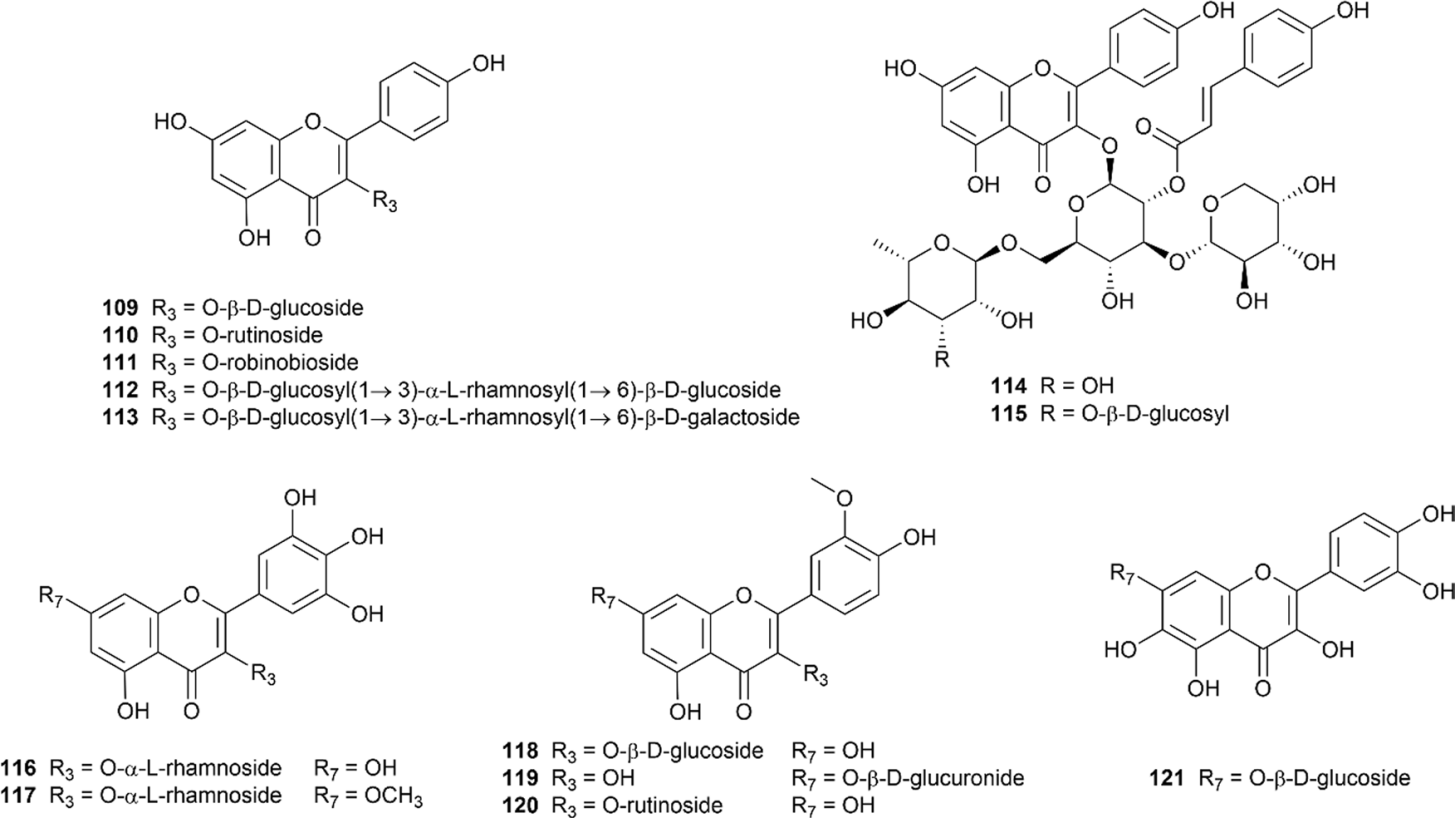
Chemical structures of retrieved flavonol derivatives

Flavonols are probably the most common flavonoid subclasses found across the plant kingdom (except for algae) [140]. They are characterized by the presence of hydroxy substituent at the C_3_ position in ring C of flavone structure (Fig. 5), also known as 3-hydroxy flavones. This subclass of flavonoids received considerable attention in the research of prominent natural substances for the simultaneous inhibitory effect on α-glucosidase and α-amylase, with 54 structures (**68–121**) taken into consideration in the present study (Additional file 1: Table S10). Quercetin **(68)**, which has been the subject of 75 articles, has garnered the most attention of all the flavonol aglycones, followed by kaempferol (**69)** and myricetin **(70)**.

Quercetin **(68)** is a natural polyphenol obtained in more than 50 plant species, as shown in the curated dataset. In terms of α-glucosidase inhibitory potential, quercetin showed superior efficacy compared to acarbose, as demonstrated by 45 out of 65 in vitro studies [41, 51, 52, 54, 61, 75, 76, 83, 93–95, 98, 101, 105, 114, 116, 141–169], making it the most well-examined flavonoid against α-glucosidase. It is worth mentioning that quercetin (**68**) was even used as a positive control in several studies. Otherwise, compared to the control drug in the same condition, this flavonol exhibited a weak α-amylase inhibitory effect [27, 33, 48, 117, 118, 139, 142, 158, 160, 165, 170–173], with the pIC_50_ being 4.07 (3.26–4.83). The mechanism underlying the inhibition of both enzymes remained controversial, which was documented to be in a mixed [141, 149, 172], non-competitive [142, 155, 166], or even competitive manner [48, 94, 114, 161, 172].

Kaempferol **(69)** is proven to be a strong α-glucosidase inhibitor, supported by at least 22 studies [41, 52, 61, 83, 86, 88, 94, 98, 101, 141, 144, 146, 150, 154, 159, 160, 169, 174–178]. Meanwhile, the subtle inhibitory ability against α-amylase promised antidiabetic efficacy while minimizing the adverse effects of kaempferol compared to acarbose. Concerning the inhibitory mechanism, kaempferol was reported to suppress α-glucosidase via a mixed-type manner while inhibiting α-amylase via a non-competitive mechanism [141, 142]. However, those were only two pieces of research evaluating the inhibitory mechanism of kaempferol, which needed further investigation to confirm.

Myricetin **(70)** displayed similar inhibitory features to those of the previously mentioned aglycones quercetin **(68)** and kaempferol **(69)**. As one of the major flavonols found in nature, myricetin is also well-known for its ability to inhibit α-glucosidase [49, 52, 61, 65, 75, 141, 143, 153, 165, 169, 171, 179–183]. The IC_50_ value on α-glucosidase of myricetin **(70)** ranged from 0.52 to 3000 µM, as reported in 19 included papers, in which 15 research revealed superior inhibitory effect compared to acarbose in the same experimental condition [49, 52, 61, 65, 75, 141, 143, 153, 165, 169, 171, 179–183]. Additionally, while α-glucosidase inhibitory mechanisms were discordantly suggested, the inhibition of α-amylase was stated competitively. Notably, myricetin **(70)** is the only flavonoid in our curated database whose inhibitory mechanism against α-amylase has been proved via X-ray crystallography [184].

A notable finding is that the α-glucosidase inhibitory activities of quercetin **(68)**, kaempferol **(69)**, and myricetin **(70)** increase with the number of hydroxyl groups attached to the B ring, as kaempferol **(69)** < quercetin **(68) < **myricetin **(70)** [61, 65, 93, 101, 139, 141, 171, 180]. This notion was consistently discussed in multiple articles, and the probable reason was due to their different capabilities to form additional hydrogen bonds with the amino acids residues in the active pocket of α-glucosidase [93, 101, 141].

Fisetin **(71)** and 3,7,4’-trihydroxyflavone **(72)** are other natural flavonol aglycones that lack the hydroxyl group at the C_5_-position of the A-ring present in compound **68–70 **(Fig. 5). Fisetin **(71)**, also known as 7,3’,4’-flavon-3-ol, exhibited a better inhibitory potency against α-glucosidase than acarbose in two studies by Yue et al. (2018) and Liu et al. (2022) [169, 185], whereas one paper by Jia et al. (2019) stated the contrary [65]. Only one included research explored fisetin **(71)** for its α-amylase inhibitory potential, which was found to be 20 times weaker than acarbose. Therefore, its dual inhibitory ability is prospective, yet further investigation should be conducted to confirm it. Compound **72**, 3,7,4’-trihydroxyflavone was reported to possess inhibitory activity against α-glucosidase, being six times more potent than acarbose [94]. This compound also poorly inhibited α-amylase in a competitive manner [139]. However, these claims were only supported by one study for each enzyme. Fisetin **(71)** and 3,7,4’-trihydroxyflavone **(72)** seemed to have an inferior α-glucosidase inhibitory effect than compound **68–70** as a result of the removal of –OH group from the 5-position, which weakened the interaction between flavonoid-enzyme [94].

Quercetagetin** (73)**, having the additional C_6_-OH in the A ring compared to quercetin **(68)**, speculated a marked affinity to targeted enzymes [186]. Concerning α-glucosidase inhibitory potential, two included studies showed opposed results in particular origin of tested enzymes [158, 186]. In a study by Wang et al. (2016) using *Saccharomyces cerevisiae*’s α-glucosidase, quercetagetin (**73**) exhibited a remarkable 4.5-fold greater effectiveness than acarbose [158]. However, when tested against human sucrase, this compound displayed 13-fold weaker inhibition compared to the positive control [186]. The three curated papers consistently confirmed the weak inhibitory potency of quercetagetin (**73**) against α-amylase [187–189].

Isorhamnetin **(74)** is also known as 3’-methoxy quercetin, a methoxyflavonol found in *Ginkgo biloba* leaf extract. As reported in 10 studies, isorhamnetin **(74)** demonstrated simultaneous inhibition against α-glucosidase and α-amylase. In comparison with acarbose, relevant research revealed isorhamnetin **(74) **with a superior pattern against both starch hydrolysis enzymes [41, 101, 146, 150, 169, 190, 191].

Kaempferide** (75),** the 4’-*O*-methyl derivative of kaempferol, has been investigated in five relevant studies for its inhibitory activity against α-glucosidase [33, 52, 62, 93, 98]. According to these studies, kaempferide **(75)** was 0.8 to 20 times more potent than acarbose [52, 62, 98]. However, a study by Tian et al. (2021) presented opposite results, claiming that kaempferide** (75) **was ten times weaker than acarbose [33]. This research also reported the α-amylase inhibitory activity of kaempferide **(75) **to be 15-fold inferior to the standard drug. Besides, it is worth mentioning that the methoxylation of C_4'_ weakened the α-glucosidase inhibitory potency compared to its unmethoxylated form (kaempferol, **69**) [33].

Quercetin-3-methyl ether **(76)** was found in five studies [33, 52, 62, 93, 98], out of which four reported weaker α-glucosidase inhibition compared to acarbose, with IC_50_ values ranging from 10.41 to 120.23 µM. Furthermore, compound **76** exhibited nine times weaker inhibition towards α-amylase as acarbose in the same experimental condition [33].

As a 2’-prenyl derivative of compound **76,** podoverine A **(77)** could inhibit both enzymes of interest, yet two times weaker than acarbose, reported in one study by Van Thanh et al. (2022) [81]. This study also extracted and evaluated the digestive enzyme inhibitory effects of 8,2’-diprenylquercetin 3-methyl ether **(78)**, and it was found to be relatively good as acarbose [81]. Solophenol D **(79)** is a 2’-geranyl derivative of quercetin. In a study by Uddin et al. (2022) [51], this compound displayed significant inhibitory effects against α-glucosidase and α-amylase, respectively, six times and four times more effective than acarbose.

As for the structure–activity relationship of compounds **74–79**, the majority of studies revealed decreased inhibitory activities against α-glucosidase of methoxyflavonols as compared to unmethoxylated forms [52, 98, 101, 105, 169]. On the other hand, some studies have argued that this phenomenon depended on the position of the substitutions [62, 93, 192]. While the impact of the methoxylation remains disputable, the prenylation of the flavonols appeared to increase the inhibitory potential against α-glucosidase [81, 193].

Compounds **80–96** are synthetic 5,7-dibromoflavonol derivatives reported by Ashraf et al. in 2020 [194]. Although achieving impressive IC_50_ values against both enzymes, these compounds are less potent than the standard drug acarbose, except for compounds **93** and **94**, which had the phenyl ring B replaced with the indole ring.

Next is a series of flavonoid glycosides **(97–115)**, each possessing a sugar residue at C_3_. Several SAR studies have shown that the attachment of sugar moieties diminished the α-glucosidase inhibitory effects of the flavonoids [41, 52, 61, 65, 75, 93, 101, 139, 141, 169, 171, 180]. Moreover, it was suggested that the 3-hydroxyl group in the C ring was essential for proper binding orientation [101].

Compounds **97–108** are natural *O*-glycoside quercetin derivatives, as displayed in Fig. 5. Isoquercetin **(97)** or quercetin-3-β-d-glucoside has been investigated in 26 research articles [41, 55, 61, 76, 101, 124, 145, 149, 154, 156, 166, 167, 169, 170, 189, 191, 195–206], making it the most extensively studied flavonol glycoside. The majority of included studies have proved the α-glucosidase inhibitory potential of isoquercetin **(97)** to be better than the standard drug (up to 32 more potent than acarbose) [41]. However, there was insufficient data on either the modes of inhibition or α-amylase inhibitory activity. Hyperoside (**98**, hyperin, or quercetin-3-β-d-galactoside) had similar patterns as **97** but was slightly inferior in disaccharide hydrolysis, demonstrated in 13 curated studies [53, 61, 156, 160, 164, 167, 169–171, 195, 207–209].

Quercitrin **(99)**, also known as quercetin-3-rhamnoside, appeared to potently inhibit α-glucosidase in a mixed manner [210], noticeably better than acarbose in more than 12 studies [41, 49, 55, 76, 108, 116, 152, 154, 167, 179, 211, 212]. However, only one research examined quercitrin as an α-amylase inhibitor, which showed quercitrin as roughly 30-fold stronger than acarbose [41]. Following this, quercetin-3-rutinoside or rutin **(100)** had excellent α-glucosidase inhibitory activity while marginally inhibiting α-amylase, which could be a promising antidiabetic agent.

Quercetin-3-*O*-(6’’-*O*-acetyl)-β-d-glucopyranoside **(101)** and quercetin-3-*O*-glucuronide **(102)** are moderate α-glucosidase and α-amylase inhibitors (comparing to acarbose) [145, 187–189, 191]. Avicularin **(103)** or quercetin-3-*O*-α-l-arabinofuranoside displayed similar features as **101** and **102** in most studies [157, 167, 169, 171, 179, 188, 200] yet showed strong inhibitory ability towards α-glucosidase in a report by Wang et al. (2018) [167].

Flavonol glycosides **104–106** are quercetin glycosides isolated from Lu’an GuaPian tea by Fang et al. in 2018 [160]. These compounds strongly inhibited α-glucosidase (three to five times better than acarbose) and α-amylase in the assays. In the same study, compounds **107–108** which are acylated quercetin glycoside derivatives were also reported for their dual target inhibition. These compounds modestly inhibited α-amylase and showed superior α-glucosidase inhibitory activities to acarbose.

Compounds **109–115** are kaempferol glycoside derivatives, in which the sugar moieties were attached at the OH 3-position. These derivatives demonstrated moderate to considerable inhibition against α-glucosidase in comparison with acarbose. The included studies also revealed that compounds **111–113** were weak to moderate α-amylase inhibitors. Kaempferol-3-*O*-β-d-glucopyranoside, also known as astragalin **(109)**, potently inhibited α-glucosidase as potent as acarbose, proven by at least eight studies [99, 124, 145, 155, 160, 197, 202, 203]. Two out of three studies examined the α-amylase inhibition of **109** as three times weaker than the control agent [160, 206]. Nicotiflorin **(110)** is a 3-rutinoside derivative, exhibiting controversial results in either inhibitory potential or inhibitory mechanisms. Concerning α-glucosidase inhibition, nicotiflorin appeared to have the IC_50_ ratio of compound-acarbose ranging from 0.1 to 8, supported in 12 separate studies [41, 61, 138, 144, 145, 160, 180, 197, 203, 213–215]. Only three papers examined the α-amylase inhibitory potential of **110**, revealing 0.32, 6, and 30 times as compared to that of acarbose [41, 160, 216]. Compounds **111–115,** which are kaempferol glycosides extracted from Lu’an GuaPian tea, share the same sugar residue as quercetin glycosides **104–108**, respectively [160]. Compounds **111–113** had weaker inhibitory activities towards two enzymes of interest than the corresponding quercetin glycosides but were still superior to acarbose. Conversely, compounds **114–115** were slightly better than their respective quercetin derivatives in the abilities to inhibit α-glucosidase and α-amylase [160].

The remaining compounds **(116–121)** are glycosides of other flavonols (myricetin, europentin, isorhamnetin, and quercetagetin). Most included papers in our database revealed that myricetin-3-rhamnoside (**116,** myricitrin) exhibited weaker inhibitory potent against two starch-hydrolyzing enzymes than acarbose and their corresponding aglycones myricetin **(70)** [61, 65, 165, 181]. The IC_50_ values against α-amylase of compound **116** lacked compromised results, which required more biological assays [49, 61, 65, 153, 165, 169, 181, 209, 217].

On the contrary, europetin-3-*O*-rhamnoside **(117)**, having an additional methyl group at position C-7, potently inhibited α-amylase eight times more effectively than the standard drug. This compound was also 20 times more potent than acarbose for α-glucosidase inhibition [153].

Isorhamnetin-3-*O*-glucoside **(118)** and isorhamnetin-7-*O*-β-D-glucopyranuronide **(119)** have displayed weaker inhibition of α-amylase and α-glucosidase than the positive control in in vitro studies [33, 91, 202]. Conversely, isorhamnetin-3-*O*-rutinoside **(120)** has shown IC_50_ values two to five times less than acarbose [41, 218, 219], indicating its superiority over other isorhamnetin glycoside derivatives **(118, 119)**. The last flavonol glycoside, quercetagetin-7-*O*-β-d-glucopyranoside **(121)**, marginally inhibited α-glucosidase in a mixed mechanism and had little effect on the α-amylase activity mentioned in a report [33].

#### Anthocyanidins and anthocyanins

**Fig. 6:**
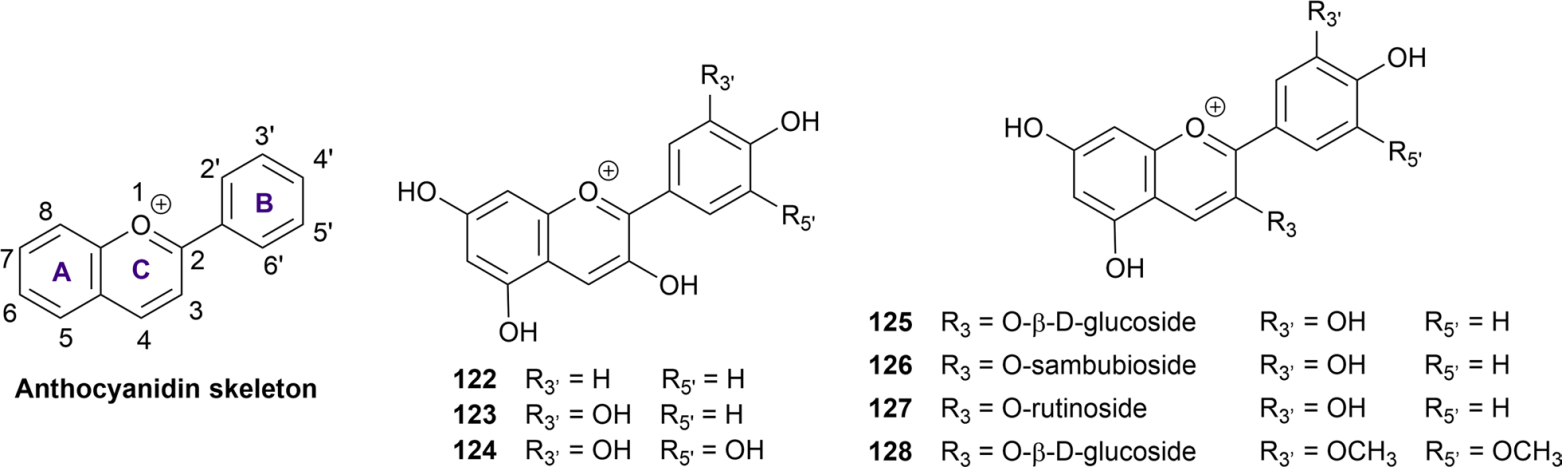
Chemical structures of retrieved anthocyanidin derivatives

Anthocyanidins and their glycoside forms (anthocyanins) are water-soluble natural pigments and are responsible for the characteristic color of many fruits and flowers. The general skeleton of anthocyanidins is the flavylium ion, with the oxygen atom in the C ring being cationized (Fig. 6). In this review, three anthocyanidins (**122–124**) and four anthocyanins (**125–128**) are included and will be introduced briefly for their activity against the two enzymes of interest (Additional file 1: Table S11).

Pelargonidin (**122**) was involved in at least two reports for its anti-α-glucosidase and α-amylase capabilities [118, 130]. The current evidence shows that pelargonidin could be a potential α-glucosidase and α-amylase inhibitor with the IC_50_ being 0.18 µM and 2.07 µM, respectively [130]. However, in a study reported by Xiao et al. [118], a different inhibitory pattern was observed, with the IC_50_ of this compound against α-amylase being 459.15 µM. Such conflict could stem from the difference in the method used in the in vitro experiments. Cyanidin (**126**) is a well-known anthocyanidin derived from red berries and was involved in at least four studies [118, 220–222]. Regarding the α-glucosidase inhibition, this compound was able to inhibit the enzyme far better than acarbose in the same condition, and the mechanism was reported to be in a non-competitive pattern [220–222]. In terms of α-amylase inhibition, the compound was regarded as a weak inhibitor of this enzyme. However, the retrieved IC_50_ values varied significantly between studies [118, 221, 222]. Delphinidin (**124**) was able to inhibit 10 to 14 times as well as acarbose in two studies against the α-glucosidase [130, 223]. The figure was 16 times when it comes to the α-amylase inhibition [130].

As for the anthocyanins, three cyanidin derivatives (**125–127**) and a malvidin derivative (**128**) were included. Some studies suggest that the introduction of sugar moiety may reduce the inhibitory activity toward both enzymes. Although the reported IC_50_ values of cyanidin-3-*O*-glucoside (**125**) and cyanidin-3-*O*-rutinoside (**127**) toward the two enzymes was still in the micromolar range, the activity of these compounds was weaker than that of acarbose in the same experimental conditions [65, 79, 209, 221, 222, 224, 225]. In a study reported by Ho et al. [222], the introduction of sambubioside (**126**), however, increases dramatically the inhibitory activity of this anthocyanin against α-glucosidase and α-amylase, with the respective IC_50_ values being 2.80 µM and 2.30 µM. In addition, malvidin-3-*O*-glucoside (**128**) was also reported to exhibit stronger inhibition toward α-glucosidase while the inhibitory activity of this compound was weaker toward α-amylase, compared to acarbose [224–226].

#### Aurone

**Fig. 7:**
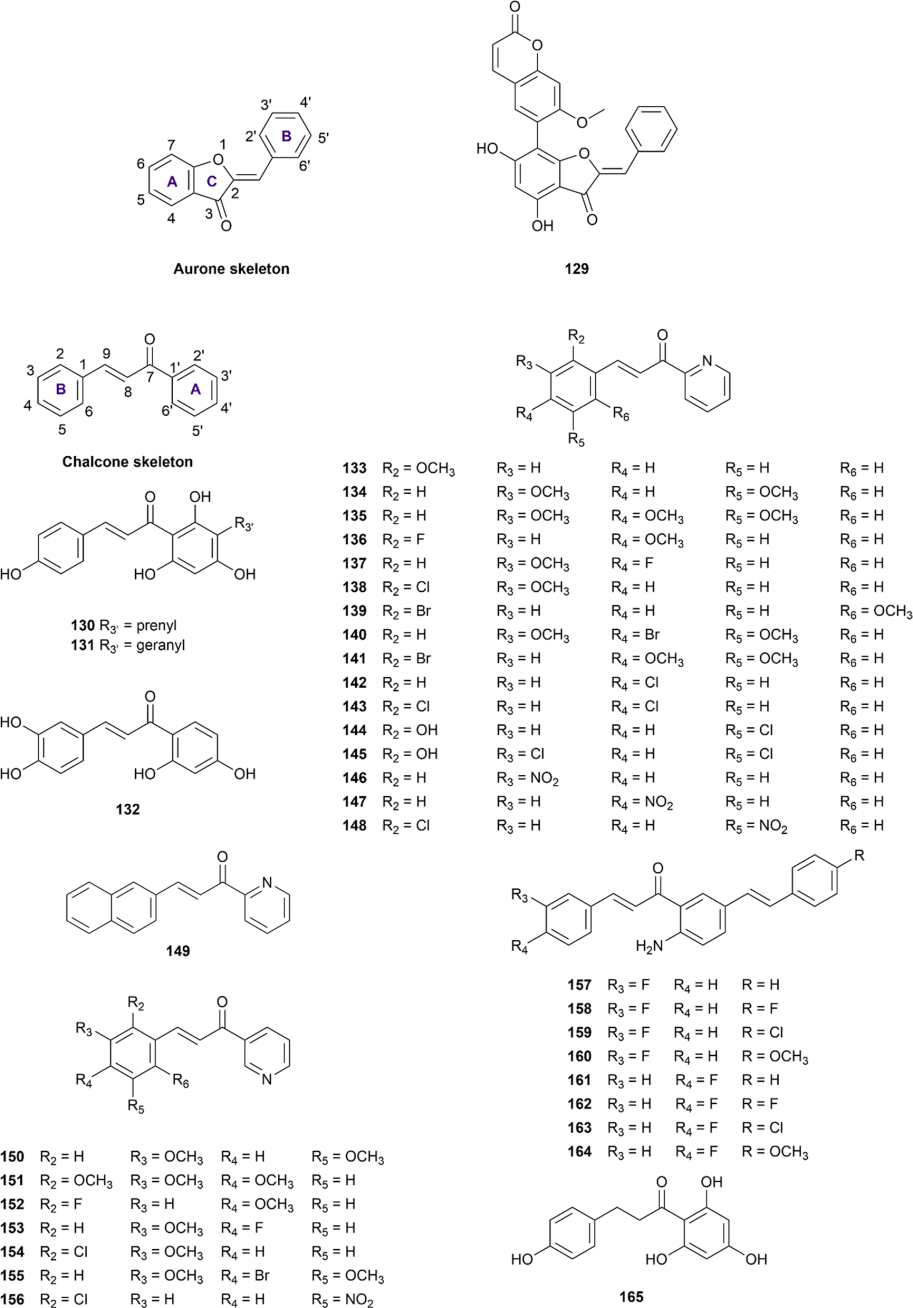
Chemical structures of retrieved aurone and chalcone derivatives

Regarding the aurone structure, although four studies have been included in our database, we are only able to retrieve one aurone which has concurrent data against the two enzymes of interest (Fig. 7). Compound **14a** ((*Z*)-6-(2-benzylidene-4,6-dihydroxy-3-oxo-2,3-dihydrobenzofuran-7-yl)-7-methoxy-2*H*-chromen-2-one, numbered **129**) is a semi-synthesized aurone-coumarin hybrid reported by Sun et al. [134]. This aurone was able to inhibit α-glucosidase and α-amylase with the respective IC_50_ values being 3.55 and 10.97 µM. The respective values for acarbose in the same condition were reported to be 224.7 µM and 2.72 µM, indicating that compound **129** is a potentially strong α-glucosidase inhibitor while exhibiting moderate inhibition toward α-amylase.

#### Chalcones

Chalcones share a common scaffold of 1,3-diaryl-2-propen-1-one, which exists in either *trans* (*E*) or *cis* (*Z*) isomers, with the *trans* (*E*) isomers being more stable and popular (Fig. 7) [227, 228]. In this review, besides the traditional chalcone structure, multiple chalcone mimics (**133–156**), in which the A ring and/or the B ring are substituted by other aromatic rings, are also included. Moreover, one naturally occurring dihydrochalcone called phloretin (**165**) is also included in the study. Additional file 1: Table S12 summarizes the main findings related to the potency of aurone and chalcone derivatives against both enzymes.

Desmethylxanthohumol (**130**) and 3’-geranylchalconaringenin (**131**) are two respective prenyl and geranyl derivatives of chalconaringenin which were isolated from hops (*Humulus lupulus*). In a study reported by Sun et al. (2018) [57], although chalconaringenin could not achieve a specific IC_50_ value toward α-amylase even at the concentration of 100 µM, the introduction of either prenyl or geranyl substituent significantly increased the inhibitory activity of this compound indicating the importance of these groups toward the inhibition manner of this chalcones. Moreover, his study also showed that the introduction of the geranyl group (**131**) increased the inhibitory potency by 20 times and 4 times, compared with desmethylxanthohumol (**123**), against α-glucosidase and α-amylase, respectively. As an effort to evaluate the anti-diabetic activities of the chalcone derivatives, 28 chalcones and 13 chalcone analogs have been synthesized and assessed for their IC_50_ value toward the two enzymes by Rocha et al. (2019) [229]. Butein (**132**) was the only and the most active inhibitor which had sufficient IC_50_ data for the two enzymes of interest.

Compounds **133–156** were 24 azachalcone derivatives synthesized by Salem et al. which show inhibition against both glucosidase and amylase [230]. In this study, the authors divided the synthesized chalcones into two categories namely categories A and B. The category “A” consists of the chalcone derivatives in which the A ring is a 2-pyridyl aromatic ring (compound **133–149**). This ring is the 3-pyridyl ring, when it comes to the “B” category (compound **150–156**) and these two categories are cordially playing a vital role in demonstrating the inhibition strength against both enzymes. However, the different substitutions at ring B are accountable for varying these activities. Among the synthesized compounds, compounds **133**, **135, 136, 146**, and **156** demonstrated excellent inhibition against both enzymes. However, in comparison with acarbose in the same experiment condition, these chalcone derivatives did not perform as well as the standard drug (IC_50_ values of 18.67 µM and 18.08 µM for α-glucosidase and α-amylase, correspondingly).

Compounds **157–164** are the seven 5-styryl-2-aminochalcone derivatives that were synthesized and reported by Mphahlele et al in 2021 [231]. These chalcone hybrids were able to inhibit both digestive hydrolysis enzymes with impressive IC_50_ values, ranging from 5.1 to 19.2 µM and 1.6 to 15.6 µM for α-glucosidase and α-amylase, respectively. Although having achieved such low IC_50_ values, these compounds were not able to surpass the inhibitory activity of acarbose in the same experiment settings, giving the IC_50_ of acarbose against α-glucosidase and α-amylase being 0.95 µM and 1.03 µM, respectively.

Phloretin (**165**) is a dihydrochalcone retrieved from either natural sources [55, 153] or a fully synthetic approach [65, 128]. This is the only dihydrochalcone that comes with sufficient data regarding its inhibitory activity against both enzymes of interest. However, the current evidence differs on whether this flavonoid can inhibit the two enzymes better than the standard drug acarbose or not. Thus, further validations are needed to confirm the potency of this compound.

#### Isoflavonoid

**Fig. 8:**
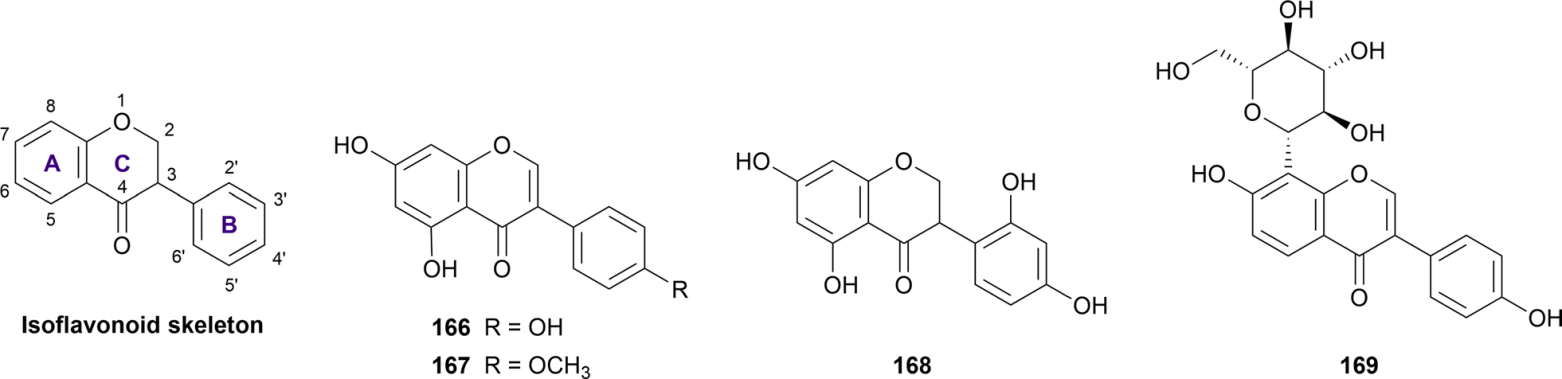
Chemical structures of retrieved isoflavonoid derivatives

Isoflavonoid is a subclass of flavonoid in which the B ring is attached to the C_3_ position instead of C_2_, forming the phenyl-3-chroman skeleton (Fig. 8). In our current review, four isoflavonoid derivatives were obtained and showed concurrent inhibitory activity against α-glucosidase and α-amylase, with genistein (**166**) being the most well-studied isoflavonoid (Additional file 1: Table S13).

Genistein (**166**) was found to be more potent than acarbose against α-glucosidase in seven out of eight studies with the inhibitory mechanism being non-competitive, mixed, or uncompetitive type [52, 65, 130, 137, 168, 232–234]. The inconsistency of the inhibitory mechanism could result from the difference in the α-glucosidase type used in these studies. Additionally, this isoflavonoid was also reported to be more potent than acarbose in the α-amylase inhibition assay in two references [130, 233]. On the other hand, the inhibitory potency of formononetin (**167**) towards α-glucosidase, is still controversial, with two out of three studies reporting it to be more effective than acarbose with micromolar potency [130, 235] while the result from Jia et al. showed a reverse pattern [65]. Nevertheless, this isoflavonoid exhibited outstanding inhibitory activity against α-amylase, in comparison with acarbose [130].

In a study by Ha et al. (2018), dalbergioidin (**168**) was extracted from *Desmodium heterophyllum* aerial parts and evaluated for its anti-diabetic properties [233]. According to the results, this substance was found to exhibit stronger inhibition against α-glucosidase but weaker inhibition against α-amylase, in comparison with acarbose in the same experimental conditions [233]. Puerarin (**169**) is the only isoflavonoid glycoside included in our current review. The inhibitory activity of puerarin towards α-glucosidase was found to be comparable to that of acarbose, with the IC_50_ values ranging from 75.60 to 524.08 µM [42, 62, 154, 234, 236] while its inhibitory activity against α-amylase was reported to be roughly 74 times lower than that of acarbose, reported in a study by Zhang et al. [111].

#### Oligomeric flavonoids

**Fig. 9:**
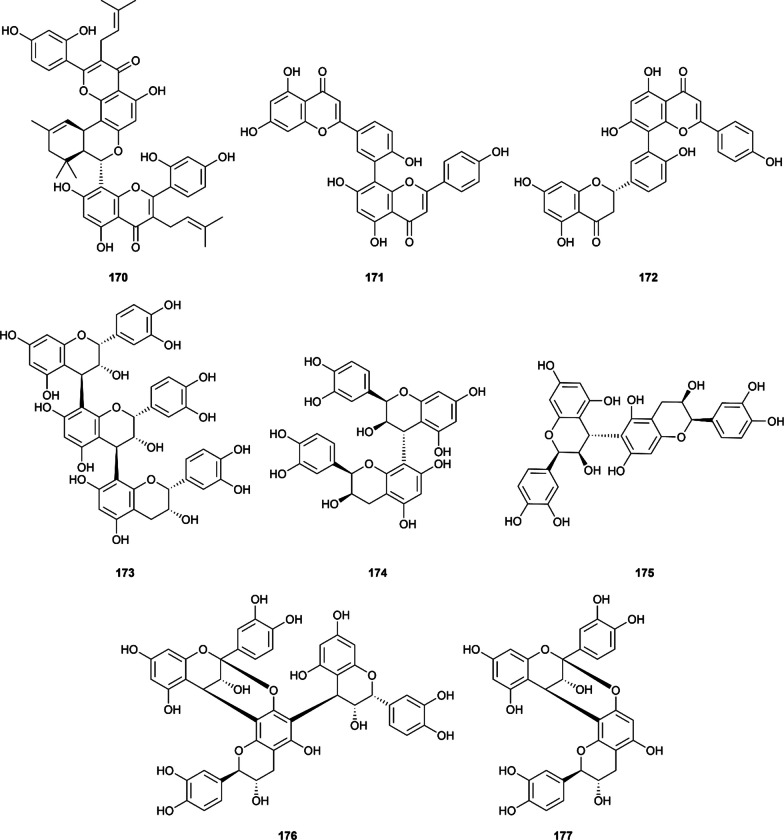
Chemical structures of retrieved oligomeric flavonoids

Oligomeric flavonoids or specifically biflavonoids have gained wide attention recently due to their health promotion effects and fascinating flavors with proanthocyanidin being the largest class of flavonoid polymer and found in various appealing-colored fruits and plants [237]. Three subgroups of proanthocyanidin (i.e., procyanidin, prodelphinidin, propelargonidin) differing in their monomers have been identified, yet their simultaneous inhibition against α-glucosidase and α-amylase received disproportionated concerns. In the current review, a total of eleven articles and eight oligomeric flavonoids are included **(**Fig. 9), with the most well-studied biflavonoid being amentoflavone (**171**). Six out of eight included structures are biflavonoids, demonstrating their potential as anti-diabetic therapeutic agents (Additional file 1: Table S14).

Kuwanon M (**170**) is the only biflavonoid extracted from the root bark of *Morus alba* L. which exhibited both α-glucosidase and α-amylase inhibition [74]. In his study, Zhao et al. reported the IC_50_ values of kuwanon M against α-glucosidase and α-amylase as 0.60 ± 0.09 µM and 1.22 ± 0.34 µM, respectively. In the same condition, acarbose only achieved the respective IC_50_ of 293.50 µM and 1.51 µM.

Amentoflavone (**171**) is the most well-studied biflavonoid included, which is formed by the condensation of two apigenin monomers via a C_3’_-C_8’’_ linkage. In the α-glucosidase inhibition assay, amentoflavone exhibited a stronger inhibition against α-glucosidase in 4 studies, with the IC_50_ ranging from 3.28 µM to 522.33 µM [34, 59, 76, 83, 101, 238]. Regarding the α-amylase inhibition, although achieving outstanding IC_50_ values below 100 µM, this compound is 2 times or 4 times less potent than the commercially available drug acarbose, reported by two publications [238, 239]. The results on 2,3-dihydroamentoflavone (**172**) also suggest a similar pattern [238].

In a study by Ho et al., the inhibitory activity of procyanidin C1 (**173**), procyanidin B2 (**174**), and procyanidin B5 (**175**) were also reported [222]. In this study, all these three oligomeric flavonoids outperformed acarbose in the both two α-glucosidase and α-amylase inhibition assay, with the IC_50_ values varied below 10 µM. These results indicate that proanthocyanidins could be promising effective and safe therapeutics in the future.

Lastly, in a report by Josim et al. (2022), the anti-α-glucosidase and anti-α-amylase properties of proanthocyanidins from *Ceriscoides campanulata* were documented [129]. In this study, compound **176** and procyanidin A1 (**177**) could inhibit α-glucosidase more effectively than acarbose (IC_50_ of 4.6 µM, 6.2 µM and 665 µM, respectively). The results on α-amylase inhibition suggested that compound **176** was also active against α-amylase with an impressive IC_50_ value (3.50 µM compared to 5.9 µM of acarbose). It could be hypothesized that the higher degree of oligomerization allowed this compound to bind and inhibit α-amylase more effectively, yet further evaluation is needed.

### Quality assessment

In this study, we employed a modified CONSORT checklist of Faggion et al. (2012) [240] for reporting in vitro studies. The checklist comprises 14 items, which are divided into six sections (Abstract, Introduction, Methods, Results, Discussion, and Other Information). However, due to the lack of information about sample size, randomization, blinding, and research protocol in most of the in vitro studies and the unnecessity of these items in the non-cellular enzymatic assay, we only include items 1–4, 10–13 for quality assessment in our review [241]. The detailed checklist and quality evaluation of included studies are available in Additional file 1: Table S5, with the overall characteristics being described in Additional file 1: Fig. S3.

As can be seen from Additional file 1: Fig. S3, all the studies satisfied at least five CONSORT items, with most of them could achieve up to eight over nine items. Specifically, it is understandable that most of the studies fulfilled items related to results presentation (Item 11) and provide adequate information for experiment reproduction (items 3, 4, 10). Nevertheless, only 75.9% of the studies indicated how the IC_50_ values were determined and 89.7% of the studies represented IC_50_ values using statistical methods. Moreover, only 18.8% of studies discussed their limitations, yet up to 90.6% of the studies provided information on the funding. Overall, these figures suggest that the included articles are of moderate to high quality and could be used in our systematic review.

## Discussions

### Structure and dual inhibitory activity on the two enzymes relationship

#### SAR and mechanism of α-glucosidase inhibition

**Fig. 10:**
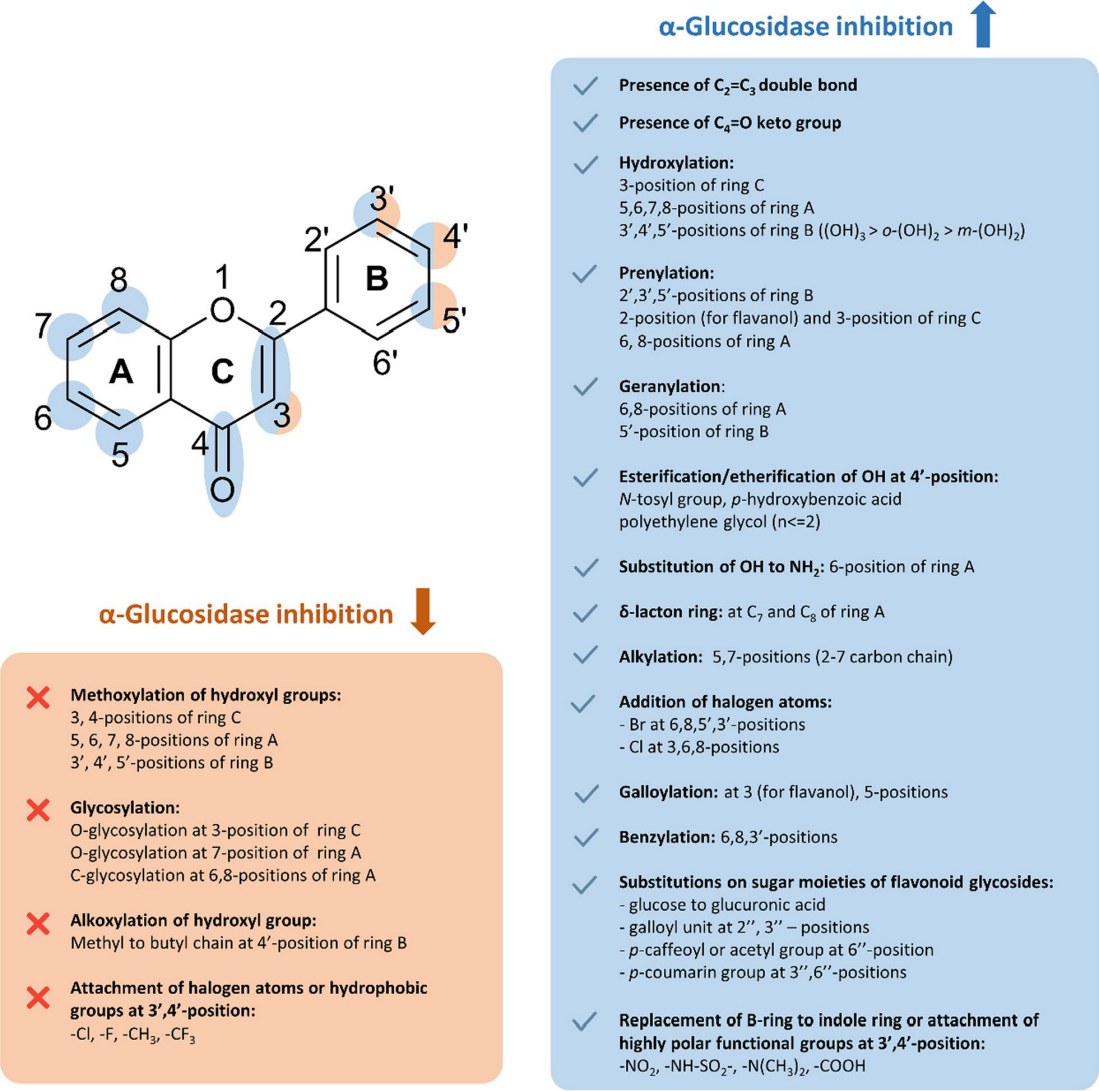
Structure–activity relationship of flavonoids against α-glucosidase

The abundance of evidence allowed us to outline and summarize the main characteristics required for the inhibition of flavonoids against α-glucosidase (Fig. 10). We observed that the decreasing order of the inhibitory activities of flavonoid subgroups against α-glucosidase could be demonstrated as (1) flavonol > flavone > flavanonol > flavanol > flavan; (2) flavonoids > flavonoid glycoside; and (3) the inhibitory potency of flavonoids is stronger than their corresponding chalcones or isoflavonoids. Nevertheless, the effect of substituents and sugar moieties should also be taken into account and a case-by-case comparison should be conducted to confirm these observations.

Concerning the flavonoid scaffold, generally, the chemical features that are favorable for the inhibition of flavonoids are (1) the presence of C_2_ = C_3_; (2) the hydroxyl group at the C_3_ position; and (3) the ketone group at the C_4_ position. These features help form a large conjugation π-system between the B-ring and the benzopyran system (A and C ring), thereby inducing the near-planarity structure and enhancing the activity of the flavonoids. Furthermore, the number and the position of hydroxyl groups attached to the flavonoids also highly affect the activity of flavonoids. An increase in the number of hydroxyl groups in either ring A (5,6,7,8-positions) and B (3’,4’,5’-positions) seems to be beneficial for the inhibitory activity and the replacement of these hydroxyl groups by alkyl or glycosyl groups generally decreases the activity of the flavonoids. However, the influence of hydroxyl groups also depends on the substitution position.

As regards the B-ring, compounds with *o*-dihydroxyl groups (catechol moiety) are more active than those with *m-*dihydroxyl groups (resorcinol moiety), and the addition of a third adjacent -OH group to form a pyrogallol moiety would significantly increase the activity of the flavonoids. Furthermore, the introduction of several highly polar functional groups such as –NO_2_, –NH-SO_2_–, –N(CH_3_)_2_, or –COOH at the 3’ or 4’-positions of the B-ring also yields stronger inhibitors while the introduction of hydrophobic or halogen atoms such as –CH_3_, –CF_3_, –Cl, –F results in a decrease in activity. Interestingly, although the free 4’-OH group is considered an important feature for α-glucosidase inhibition, several studies showed that the esterification of this OH group with the carboxylic group of *p*-hydroxybenzoic acid and the etherification of this OH group with a polyethylene glycol chain (up to dimer) or *N*-tosyl group seems to enhance the activity of the flavonoids.

As for the A ring, hydroxyl groups at the C_5_ or C_7_ position are abundantly presented in nature and usually listed as important features for α-glucosidase inhibition, but the alkylation of these hydroxyl groups with the alkyl chains (2–7 carbons) seems to benefit the activity while the methylation possibly exerts the opposite. Additionally, the introduction of the prenyl group and geranyl group at several positions is beneficial for the activity. Relatively, the induced effect of the geranyl group on the flavonoids’ activities is stronger than that of the prenyl group and the substitution at the 6-position affords a stronger inhibitor than that at the 8-position. Several attempts to attach the halogen atoms to the flavonoid scaffold have been reported, with the substitution of bromine atoms at 6,8,5’,3’-positions and the substitution of chlorine atoms at 3,6,8-positions resulting in strong inhibitors. Surprisingly, the replacement of the hydroxyl group at C_3_ with the chlorine atom does not significantly affect the inhibitory activity of the flavonoids. Lastly, the attachment of the hydroxybenzyl moiety to the 6,8,3’-positions and the formation of the fourth δ-lactone ring at C_7_ and C_8_ also enhance the inhibitory effect.

As previously mentioned, the glycosylation of flavonoids appears not to favor the inhibitory capability of the flavonoid. Several hypotheses for this phenomenon have been proposed, including the increase of steric hindrance, or an increase in molecular size, polarity, and the presence of a non-planar structure, all of which could affect the binding capability of the flavonoids. The impact of different sugar molecules on flavonoid glycosides is not well understood, and it may vary depending on the compound. Nevertheless, several naturally occurring substituents on sugar moiety that may recover the activity of flavonoid glycosides have been reported, including the oxidation from glucose to form glucuronic acid, or the addition of the galloyl units, *p-*caffeoyl, *p-*coumarin or acetyl groups at 2’’,3’’, or 6’’-positions of the sugar part.

The inhibitory mechanism of flavonoids towards α-glucosidase is a subject of ongoing debate. Although utilizing the same enzyme and substrate, different inhibitory mechanisms have been reported for the same flavonoid molecule, such as epicatechin (**4**), luteolin (**30**)**,** quercetin (**68**), isoquercetin (**105**), and genistein (**166**). These discrepancies could be due to variations in the assay protocols, such as differences in the concentration of enzyme and substrate, and incubation time used by various research groups. After the analysis of numerous studies, there is still no consensus on the inhibitory mechanism of flavonoids against α-glucosidases. Flavonoids could inhibit α-glucosidase competitively or non-competitively or even only when the enzyme–substrate complex has been formed (uncompetitively). Thus, a case-by-case evaluation is needed to confirm the inhibitory mechanism.

It is worth mentioning that despite nearly four decades of research on the anti-α-glucosidase activity of flavonoids (since 1986—the article is not included due to the absence of acarbose), no co-crystallized structure of flavonoids-α-glucosidase from either origin has been reported to the date the manuscript was written. While molecular modeling techniques, such as homology modeling, molecular docking, and molecular dynamics simulation, have been utilized to overcome this limitation, experimental three-dimensional structures are still needed to gain insights into how flavonoids inhibit α-glucosidase at an atomistic level.

#### SAR and mechanism of α-amylase inhibition

**Fig. 11:**
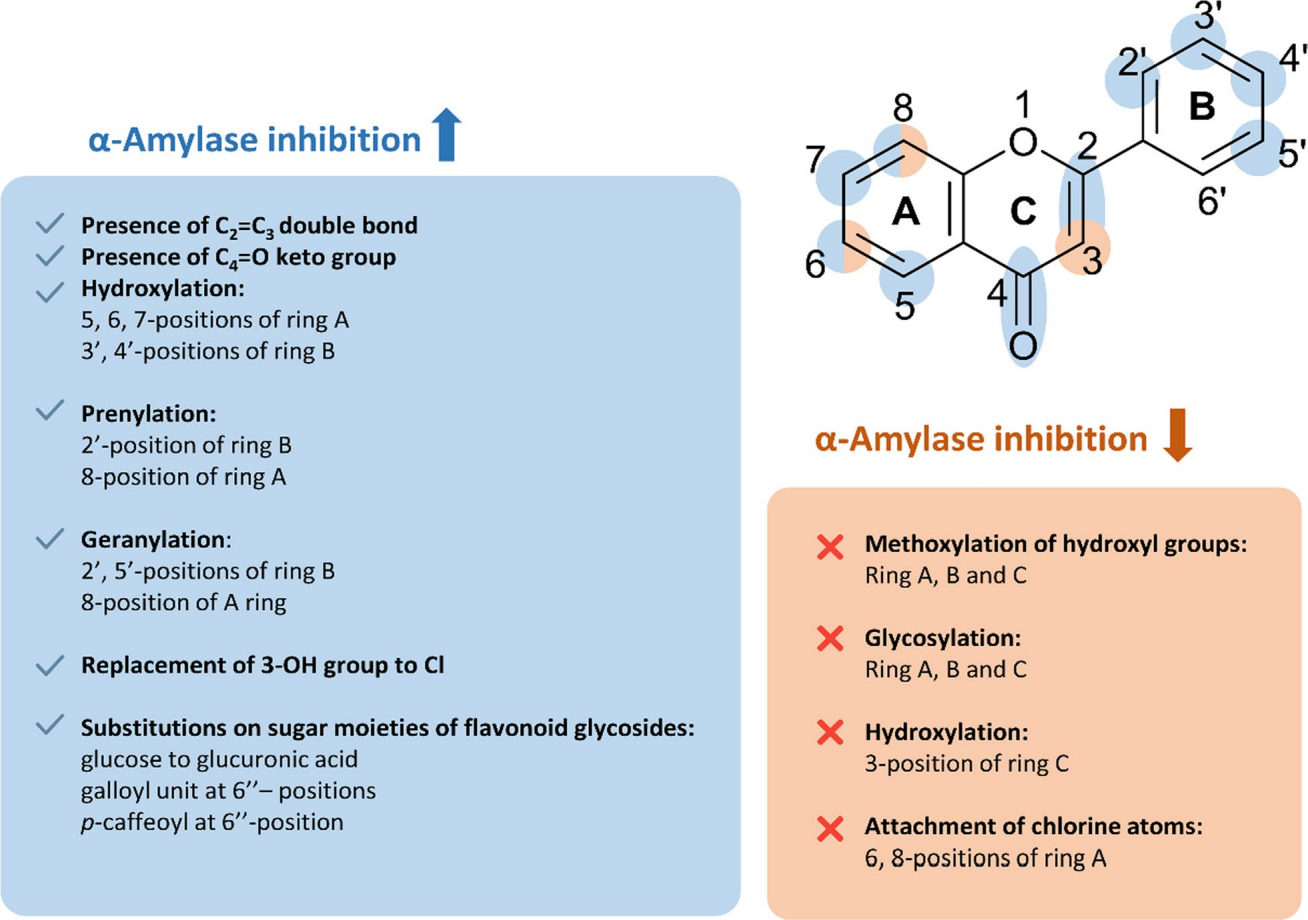
Structure–activity relationship of flavonoids against α-amylase

The evidence supporting the inhibition of flavonoids against α-amylase is not as abundant as for α-glucosidase, but it is still sufficient to establish a preliminary SAR for flavonoids against this digestive enzyme (Fig. 11). In general, the activity of aglycone flavonoids is stronger than their corresponding glycosides and isoflavonoids.

Several common features have been considered favorable for the inhibition against α-amylase, including (1) the double bond of C_2_ = C_3_; (2) the keto group at the C_4_-position; and (3) hydroxyl groups at 5,6,7,3’,4’-positions. Generally, the higher number of hydroxyl groups leads to a higher activity of flavonoids, yet the position at which the hydroxyl groups are introduced is also important. In fact, the hydroxyl group at C_3_-position, which is crucial for the inhibitory activity against α-glucosidase, appears to be detrimental to the activity against α-amylase and the replacement of this OH-group with a chlorine atom may increase the latter activity of the flavonoids. Similar to α-glucosidase inhibition, the catechol moiety at the B-ring is responsible for the strong α-amylase inhibitory activity while the addition of another adjacent OH-group at C_5’_ only enhances the effect modestly. Prenylation and geranylation at several specific positions also similarly enhance inhibitory activity. It is observed that the induced effect of the geranyl group is stronger than that of the prenyl group at the same position.

With respect to the reducing factors, methylation or glycosylation of hydroxyl groups is also found to weaken the inhibitory effects. The effect is more pronounced when it comes to crucial positions such as C_3_, C_4’_, and C_7_. Nonetheless, the recovery in the activity of flavonoid glycosides whose sugar moiety has been substituted with several polyphenol groups is also recorded. In addition, we also observe that the presence of chlorine substituent at either 6 or 8-positions does not favor the anti-α-amylase activity.

**Fig. 12:**
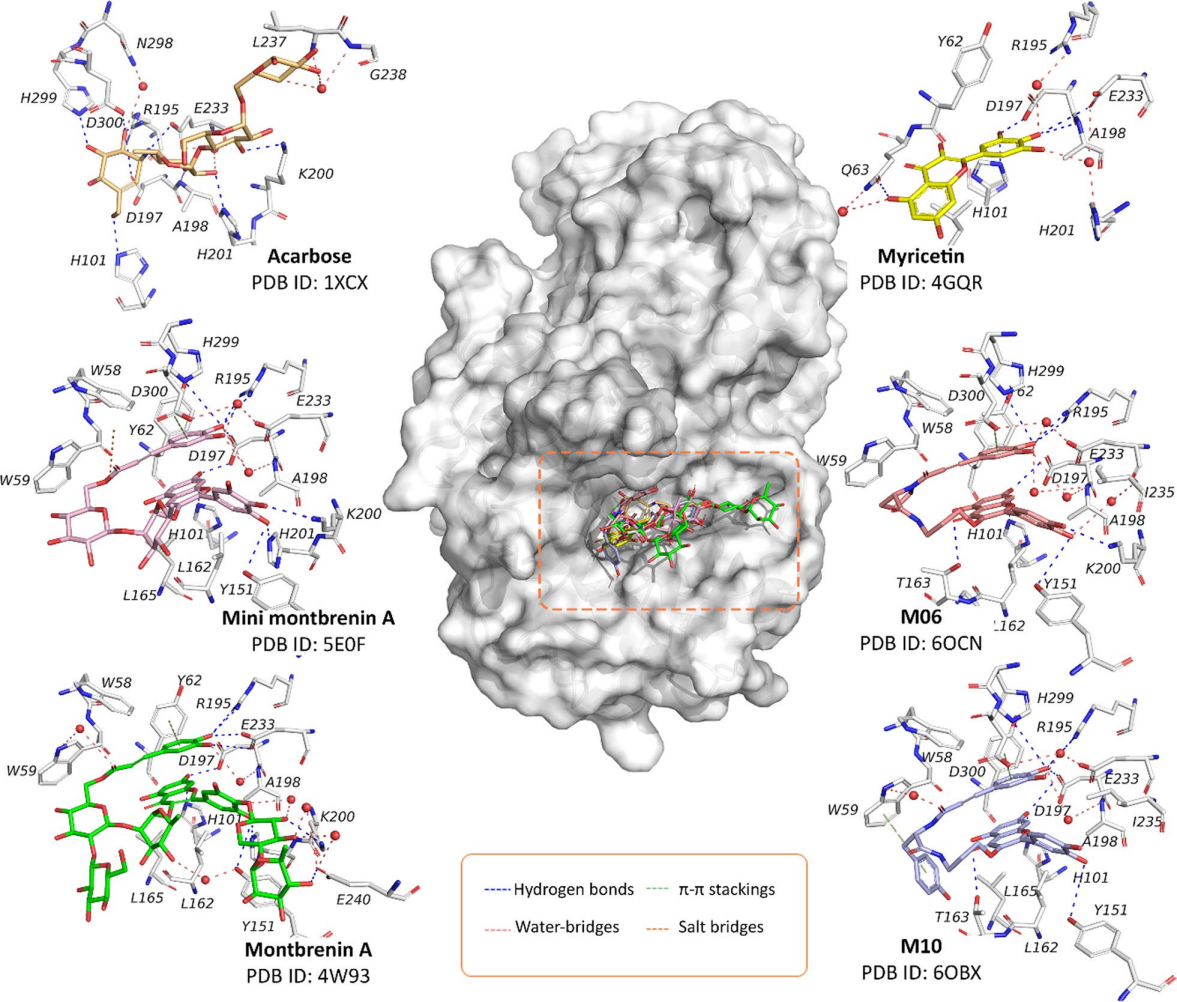
Interactions between the flavonoid derivatives with human pancreatic α-amylases retrieved from the Protein Data Bank as of April 2023. The small molecules with their corresponding colors and PDB IDs are: Acarbose (orange—PDB ID: 1XCX) [242]; Myricetin (yellow—PDB ID: 4GQR) [184]; Montbretin A (Green—PDB ID: 4W93) [243]; “Mini-montbretin A” (Pink—PDB ID: 5E0F) [243]; **M06** (Salmon—PDB ID: 6OCN) [244]; and **M10** (Blue—PDB ID: 6OBX) [244].

Similar to findings on α-glucosidase, the inhibitory mechanism of flavonoids against α-amylase exhibits a wide range of reported outcomes. Within our curated database, we have observed that flavonoids can inhibit α-amylase competitively, non-competitively, or through a combination of both mechanisms. However, no instances of uncompetitive inhibition have been documented. It is important to acknowledge that although certain compounds show consistent mechanisms of inhibition, there are still discrepancies within specific compounds. These variations may arise due to variations in assay protocols and detection methods employed across different studies. However, the structural information from our search on the Protein Data Bank as of April 2023 suggests a more homogenous result, leaning toward the competitive mechanism (Fig. 12). These structures provide valuable insights into the binding site and protein–ligand interactions that underlie the mechanism of action of flavonoids, facilitating future rational drug design efforts. In our manuscript, we hence provide a concise discussion of these protein–ligand interactions and their potential implications for drug discovery.

As can be seen from Fig. 12, all the available structures suggest that flavonoid derivatives occupied the same binding site of acarbose, which is also the active site responsible for the hydrolysis of starch [242]. However, upon taking a closer look at the interaction between the small molecules and the enzyme, there is a transition between the conformation of myricetin to other 3-*O*-glycosylated and 3-*O*-caffeate flavonoid derivatives. The myricetin-α-amylase complex shows that the B-ring headed toward the catalytic triad (Asp197, Glu233, and Asp300) and formed multiple hydrogen bonds and water-meditated bridges between the pyrogallol moiety and the residues in the cavity pocket. Additionally, the hydroxyl group at C_5_ of the A-ring could form hydrogen bonds with the Gln63 residue, thereby stabilizing the ligand in the binding pocket. These observations confirm the SAR that the OH at C_3_ is not necessary for the binding capability, but several features such as the hydroxyl groups at the B and the A-ring are required. However, in flavonoid glycosides and caffeate flavonoid derivatives, the aglycone part is oriented in the opposite direction, with the B-ring headed toward the solvent site and forming several hydrogen bonds with residues such as Tyr151 and Lys200. In this case, the hydroxyl group at the C_7_-position is responsible for the interaction with catalytic residues such as Asp197 and Glu233. The addition of the caffeate moiety (as in the case of **M06** and **M10**) allowed these flavonoids to form more hydrogen bonds with the enzyme residues and additional π–π contacts with the Tyr62, thereby inducing inhibitory activity. On the other hand, the addition of sugar moiety (as of montbrenin A and mini-montbrenin A) did not significantly increase the contact with the enzyme residues, which is consistent with our SAR observations. In summary, it could be postulated that the binding conformation of flavonoid aglycones within the α-amylase pocket is similar to that of myricetin, while the opposite conformation of the aglycone part is expected for flavonoid glycosides or caffeate flavonoids. Even so, as mentioned previously, multiple mechanisms of flavonoid inhibition towards α-amylase have been reported. Therefore, further validation is necessary to confirm the binding patterns of new flavonoids with α-amylase.

### Limitations

Our work has highlighted the concurrent α-glucosidase and α-amylase inhibitory effects of flavonoid compounds from 339 included studies, yet several limitations should be considered.

The inconsistent assay methods and enzyme origins are two limitations that could impact the interoperability of the data on α-glucosidase inhibition. This can lead to variations in IC_50_ values and inhibitory mechanisms being reported among studies, making it difficult to compare results. A lack of a common recommendation has hindered researchers in replicating experiments and verifying findings. In addition, it is interesting to note that although the ultimate therapeutic targets are human enzymes, yeast or rat proteins are often preferred in in vitro studies due to their availability and affordability. However, using yeast enzymes may not accurately reflect the inhibition of human MGAM and SI enzymes, which is an important consideration when designing experiments. Therefore, a consensual protocol is needed for in vitro assays using human-sourced enzymes to improve predictive capacity and reduce potential variations in results.

Recent efforts to develop quantitative methods for the inhibitory activity evaluation of flavonoids on human enzymes have been reported. For example, studies by Barber et al. (2021) [186] and Pyner et al. (2017) [245], employed human intestinal Caco-2 cells to extract human-sourced maltase and sucrase have been conducted. Additionally, although not being included in our literature database due to the absence of inhibitory data of acarbose, the study by Lim et al. in 2021 [246] presents a promising model for future evaluation of the anti-α-glucosidase activity. In this study, the researchers utilized human Ct-MGAM and Nt-MGAM expressed on the baculovirus Sf9 system and *Drosophila* S2 cells, respectively. This approach could improve the reliability of in vitro results thereby enhancing the success rate of drug candidates in subsequent evaluations. These studies provide valuable insights and could be served as guiding references for future research on evaluating the activity of flavonoids toward human-sourced α-glucosidases.

Furthermore, there is a gap in our understanding of the interaction between flavonoids and human MGAM or SI at an atomistic level, which could provide valuable insights into their inhibitory mechanism and be used for future rational drug design. Therefore, future studies should focus on filling these gaps to explore the mechanism of flavonoids' inhibitory effects on human α-glucosidases.

The results of α-amylase inhibition, in contrast, show less variation and support a competitive mechanism. This is likely because most research groups have conducted experiments using porcine α-amylase, which shares a high degree of sequence identity with human α-amylase. This enables more accurate extrapolation from the inhibition of flavonoids on porcine enzymes to human-sourced counterparts. It is worth noting that commercial human α-amylase is also commercially available, allowing for more effective and cost-efficient inhibition assays. Additionally, the availability of flavonoid-α-amylase complexes enables the identification of the core interactions involved in inhibition and confirms SAR observations, thus providing a valuable tool for drug optimization before any further investigation.

There were several limitations encountered during our review process. Firstly, our database was limited to papers published in English, which meant that we were unable to retrieve data from a number of studies in other languages such as Chinese, Japanese, Korean, Portuguese, and German. This language barrier prevented us from accessing potentially relevant research articles. Additionally, although had intended to cover studies in Vietnamese, the shortage of a Vietnamese journal database also hindered us from retrieving research articles in Vietnamese. Furthermore, our systematic review has taken a considerable period due to the extended data analysis process, initiated in August 2022 and culminated in May 2023. We can only accumulate data until August 21, 2022, and thus, some of the latest articles published after this date may not have been included in our analysis. Hence, future follow-up reviews may be necessary to update our findings with the latest research articles.

## Conclusions and future perspectives

Diabetes has become a challenging health problem with rapidly increasing incidents and mortality yearly. Especially, sustainable management of diabetes has faced the consistent development of drug resistance, which recognized a significant necessity for novel treatments with better pharmacological characteristics. In recent years, researchers have been increasingly turning to safe and effective phytochemicals, particularly flavonoids, because of their impressive ability to interfere with starch digestion.

The ongoing data on flavonoids in vitro inhibition on α-glucosidase and α-amylase have grown substantially. However, to the best of our knowledge, there has yet to be any exhaustive data collection efforts and discussion to provide this insight. Our review established a comprehensive overview of the inhibition potential of flavonoids against two starch-digestive enzymes, covering six databases and 974 standardized unique flavonoid structures, each with corresponding IC_50_ values for the test substance and acarbose. In addition to the inhibition data, we also recorded the origin of flavonoids, testing methods, and the underlying mechanism of inhibition. The curated database can lay a foundation for future ligand-based QSAR studies, facilitating natural products drug discovery efforts aimed at α-glucosidase inhibition while minimizing side effects related to α-amylase. A number of remarks have been discussed thoroughly, including structure characteristics that would impact the activity of interest. Additionally, considerable efforts have been made to curate and assess the molecular-level interactions between flavonoids and two enzymes. Subsequently, we wish to provide a balance of evidence for the advance in the discovery of new antidiabetic candidates.

On a side note, we identified exciting prospects that should be brought to the attention of investigators.

1. As for in vitro assays, the inhibitory effects of flavonoids varied significantly with respect to the methods, substrates, and enzyme origins, thus impeding the reproducibility of consistent results. Hence, future studies should focus on developing consensual protocols for in vitro enzyme activity evaluation and coming up with unambiguous instructions to improve the replication of results.
2. It should be noted that while porcine α-amylase could be used provisionally in replacement of human α-amylase in vitro due to its sequential and structural identity with human α-amylase, yeast or rat-sourced α-glucosidases diverged considerably from human MGAM and SI enzymes. Taking account of these distinctions, it is crucial to establish in vitro assays using human-sourced enzymes to improve the reliability of the data for successive stages on living models.
3. The molecular interactions between flavonoids and enzymes are of great importance in the drug development pipeline. Despite recent advances in scientific approaches and modeling techniques, little research has been reported about the mechanisms underlying the inhibitory effects of flavonoids on α-amylase and α-glucosidases. Thus, this area remained a research gap that required further investigation.

## Methods

This systematic review is conducted in accordance with The Preferred Reporting Items for Systematic Reviews and Meta-Analysis (PRISMA) statement [247]. The protocol of this review has been deposited on ResearchGate in August 2022 (https://doi.org/10.13140/RG.2.2.17980.31368/2). For details of the methodology, including the study protocol, eligibility criteria, information sources and search strategies, study selection, data collection process, outcomes and prioritization, quality assessment, and data processing, readers are encouraged to refer to the Detailed methodologies in Additional file 1.

### Supplementary Information

**Additional file 1. Detailed methodologies. Fig. S1.** Schematic illustration of starch hydrolysis pathway in humans and potential mechanisms of action of flavonoids as anti-diabetic agents. **Fig. S2.** PRISMA flow chart for the identification and screening process. **Fig. S3**. Result of quality assessment using the modified CONSORT checklist. **Table S1**. PRISMA checklist. **Table S2**. Details of search terms and the number of records curated in each database as of August 21, 2022. **Table S3**. Details of search terms and the number of complex structures examined on RCSB Protein Data Bank as of April 27 2023. **Table S4.** Detailed characteristics of included studies. **Table S5**. Quality assessment of included studies. **Table S6**. In vitro α-glucosidase and α-amylase inhibitory effects of retrieved flavan and flavanol derivatives. **Table S7**. In vitro α-glucosidase and α-amylase inhibitory effects of retrieved flavanone derivatives. **Table S8**. In vitro α-glucosidase and α-amylase inhibitory effects of retrieved flavanonol derivatives. **Table S9**. In vitro α-glucosidase and α-amylase inhibitory effects of retrieved flavone derivatives. **Table S10**. In vitro α-glucosidase and α-amylase inhibitory effects of retrieved flavonol derivatives. **Table S11**. In vitro α-glucosidase and α-amylase inhibitory effects of retrieved anthocyanidin derivatives. **Table S12**. In vitro α-glucosidase and α-amylase inhibitory effects of aurone and chalcone derivatives. **Table S13**. In vitro α-glucosidase and α-amylase inhibitory effects of retrieved isoflavonoids. **Table S14**. In vitro α-glucosidase and α-amylase inhibitory effects of retrieved oligomeric flavonoids.

## Data Availability

The code and the datasets used in this systematic review are provided publicly at https://github.com/MedChemUMP/FDIGA.
