# Supplementary material for: Flavonoids as dual-target inhibitors against α-glucosidase and α-amylase: a systematic review of in vitro studies
Source: Nat Prod Bioprospect. 2024 Jan 8;14(1):4. doi: 10.1007/s13659-023-00424-w (PMC10772047; doi:10.1007/s13659-023-00424-w)
Supplement: Supplementary file 1 — Additional file 1. Detailed methodologies. Fig. S1. Schematic illustration of starch hydrolysis pathway in humans and potential mechanisms of action of flavonoids as anti-diabetic agents. Fig. S2. PRISMA flow chart for the identification and screening process. Fig. S3. Result of quality assessment using the modified CONSORT checklist. Table S1. PRISMA checklist. Table S2. Details of search terms and the number of records curated in each database as of August 21, 2022. Table S3. Details of search terms and the number of complex structures examined on RCSB Protein Data Bank as of April 27 2023. Table S4. Detailed characteristics of included studies. Table S5. Quality assessment of included studies. Table S6. In vitro α-glucosidase and α-amylase inhibitory effects of retrieved flavan and flavanol derivatives. Table S7. In vitro α-glucosidase and α-amylase inhibitory effects of retrieved flavanone derivatives. Table S8. In vitro α-glucosidase and α-amylase inhibitory effects of retrieved flavanonol derivatives. Table S9. In vitro α-glucosidase and α-amylase inhibitory effects of retrieved flavone derivatives. Table S10. In vitro α-glucosidase and α-amylase inhibitory effects of retrieved flavonol derivatives. Table S11. In vitro α-glucosidase and α-amylase inhibitory effects of retrieved anthocyanidin derivatives. Table S12. In vitro α-glucosidase and α-amylase inhibitory effects of aurone and chalcone derivatives. Table S13. In vitro α-glucosidase and α-amylase inhibitory effects of retrieved isoflavonoids. Table S14. In vitro α-glucosidase and α-amylase inhibitory effects of retrieved oligomeric flavonoids. [file 13659_2023_424_MOESM1_ESM.docx]

**Supplementary information**

**Flavonoids as dual-target inhibitors against α-glucosidase and α-amylase:
a systematic review of *in vitro* studies**

Thua-Phong Lam^a,b,†^, Ngoc-Vi Nguyen Tran^a,b,†^, Long-Hung Dinh Pham^a,c^, Nghia Vo-Trong Lai^a^, Bao-Tran Ngoc Dang^a^, Ngoc-Lam Nguyen Truong^a^,
Song-Ky Nguyen-Vo^a^, Thuy-Linh Hoang^d^, Tan Thanh Mai^a,*^, Thanh-Dao Tran^a,*^

^a^ Faculty of Pharmacy, University of Medicine and Pharmacy at Ho Chi Minh City, 700000 Ho Chi Minh City, Vietnam

^b^ Faculty of Pharmacy, Uppsala University, 75105 Uppsala, Sweden

^c^ Department of Chemistry, Imperial College London, London W12 0BZ, United Kingdom

^d^ California Northstate University College of Pharmacy, 95757 California, USA

^†^ These authors contributed equally and are considered co-first authors.

^*^ Corresponding authors: Tan Thanh Mai, [mthtan@ump.edu.vn](mailto:mthtan@ump.edu.vn); Thanh-Dao Tran, [daott@ump.edu.vn](mailto:daott@ump.edu.vn);

**Availability of data:**

The code and the datasets used in this systematic review are provided publicly at: <https://github.com/MedChemUMP/FDIGA>

# Contents

[DETAILED METHODOLOGIES S3](#_Toc151636952)

[Protocol and registration S3](#_Toc151636953)

[Eligibility criteria S3](#_Toc151636954)

[Information sources and search strategies S3](#_Toc151636955)

[Study selection S3](#_Toc151636956)

[Data collection process and data items S4](#_Toc151636957)

[Outcomes and prioritization S4](#_Toc151636958)

[Quality assessment S4](#_Toc151636959)

[Data processing S4](#_Toc151636960)

[Figure S1. Schematic illustration of starch hydrolysis pathway in humans and potential mechanisms of action of flavonoids as anti-diabetic agents S5](#_Toc151636961)

[Figure S2. PRISMA flow chart for the identification and screening process. S6](#_Toc151636962)

[Figure S3. Result of quality assessment using the modified CONSORT checklist S7](#_Toc151636963)

[Table S1. PRISMA checklist S8](#_Toc151636964)

[Table S2. Details of search terms and the number of records curated in each database as of August 21, 2022 S11](#_Toc151636965)

[Table S3. Details of search terms and the number of complex structures examined on RCSB Protein Data Bank as of April 27 2023 S12](#_Toc151636966)

[Table S4. Detailed characteristics of included studies S13](#_Toc151636967)

[Table S5. Quality assessment of included studies S59](#_Toc151636968)

[Table S6. *In vitro* α-glucosidase and α-amylase inhibitory effects of retrieved flavan and flavanol derivatives. S70](#_Toc151636969)

[Table S7. *In vitro* α-glucosidase and α-amylase inhibitory effects of retrieved flavanone derivatives. S70](#_Toc151636970)

[Table S8. *In vitro* α-glucosidase and α-amylase inhibitory effects of retrieved flavanonol derivatives. S72](#_Toc151636971)

[Table S9. *In vitro* α-glucosidase and α-amylase inhibitory effects of retrieved flavone derivatives. S73](#_Toc151636972)

[Table S10. *In vitro* α-glucosidase and α-amylase inhibitory effects of retrieved flavonol derivatives. S77](#_Toc151636973)

[Table S11. *In vitro* α-glucosidase and α-amylase inhibitory effects of retrieved anthocyanidin derivatives. S84](#_Toc151636974)

[Table S12. *In vitro* α-glucosidase and α-amylase inhibitory effects of aurone and chalcone derivatives. S85](#_Toc151636975)

[Table S13. *In vitro* α-glucosidase and α-amylase inhibitory effects of retrieved isoflavonoids. S89](#_Toc151636976)

[Table S14. *In vitro* α-glucosidase and α-amylase inhibitory effects of retrieved oligomeric flavonoids. S89](#_Toc151636977)

[References S91](#_Toc151636978)

# DETAILED METHODOLOGIES

## Protocol and registration

This study aimed to provide a general overview of flavonoids that could inhibit both α-glucosidase and
α-amylase. Our study is conducted following The Preferred Reporting Items for Systematic Reviews and Meta-Analysis (PRISMA) statement ^1^. The protocol of this review was uploaded on ResearchGate in August 2022 (DOI: 10.13140/RG.2.2.17980.31368/2). As this study is only concerned with *in vitro* studies, we could not register our protocol on the International Prospective Register of Systematic Reviews (PROSPERO) server. The PRISMA checklist is available in **Table S1**.

## Eligibility criteria

A study must meet the following criteria to be included in this systematic review: (1) original *in vitro* studies reporting the inhibitory effects of purified flavonoids on α-amylase or α-glucosidase; (2) provide IC_50_ results of tested compounds with the concurrent positive control (3) be written in English or Vietnamese. For comparison purposes, we only included the studies which used acarbose as the positive control, as acarbose is the most common substance used in the literature. For exclusion criteria, case reports, reviews, systematic reviews, conference abstracts, and unpublished papers were excluded. We also eliminated pure *in silico*, *in vivo*, *ex vivo*, or clinical studies. Studies without concurrent positive samples, and those without a clear method description that could present risks of producing unreliable extracted data, were also excluded.

## Information sources and search strategies

An electronic search was conducted on six databases until 21 August 2022, including PubMed, Virtual Health Library (VHL), EMBASE, SCOPUS, Web of Science (WOS), and WHO Global Index Medicus (GIM). The search terms (alpha-glucosidase OR maltase OR sucrase OR alpha-amylase) AND (flavonoid OR flavonoids) were customized to fit the corresponding electronic databases. No time restriction, language restriction, and article type restriction were set at this stage. In addition to literature screening using the aforementioned databases, we also searched for structures of flavonoid-enzyme complexes on the RCSB Protein Data Bank (PDB) (<https://www.rcsb.org>). The search was conducted on April 27^th^, 2023, to systematically evaluate the interaction patterns between flavonoids and the two enzymes of interest. The PDB search terms were set to be the Enzyme Commission number (E.C. number) of the corresponding enzymes. The detailed search strategy is available in **Table S2** and **Table S3**.

## Study selection

During this research, we screened the papers by titles, abstracts, and full texts with the assistance of the online systematic review software Rayyan (<https://rayyan.ai>) ^2^. Our study selection process consists of three main stages, including duplicate and automatic removal, title/abstract screening, and full-text screening.

The study selection procedure began with eliminating duplicate papers using the Zotero and Rayyan programs, followed by splitting the initial database into three portions for the next stage. Six reviewers who worked in pairs of two (T.P.L. and S.K.N.V.; N.V.T.L. and B.T.N.D.; N.V.N.T. and N.L.N.T.) performed the title/abstract screening for matching articles. Subsequently, full-text screening was conducted to reach the final include or exclude decision. The previously indicated reviewers operated independently and were blinded throughout the screening process. A debate and consensus would be held if there was a dispute, and a third reviewer's viewpoint (T.D.T. and T.T.M.) would be sought to conclude. The summary of the screening results is graphically presented in a PRISMA flow chart (**Figure S2).** In addition, the reasons for full-text exclusion are illustrated in the flow chart.

## Data collection process and data items

The following information was collected from each study and stored in a Microsoft Excel workbook. The extracted data consisted of 3 parts: (1) Study determinants, including title, authors, and publication year; (2) Study design (assay protocol, types of enzymes), sample size, intervention, and comparator; (3) Outcomes of the study. Concerning primary outcomes, we searched for the α-amylase and/or α‑glucosidase inhibitors' chemical structures and their inhibitory efficiency.

## Outcomes and prioritization

According to the respective flavonoid subclasses, compounds and derivatives that have one or both inhibitory activities against α-glucosidase and α-amylase were classified. The structures of compounds were redrawn using ChemDraw software ^3^ and stored as Simplified molecular-input line-entry system (SMILES) strings in the same aforementioned workbook. The inhibitory activity outcomes of flavonoids against enzymes were presented as the half-maximal inhibitory concentration (IC_50_).

## Quality assessment

A modified version of the CONSORT checklist developed by Faggion ^4^ was used in the quality assessment process. The checklist consisted of 14 items concerning the assessment of the abstract, introduction, method, results, discussion, and other information to interpret the quality of the research studies. In this study, we only employed Items 1-4, and 10-13 for quality evaluation due to the lack of information on randomization, blinding, and sample size in most of the studies. The detailed checklist and quality evaluation of included studies are provided in **Table S5.**

## Data processing

As one natural product may have many synonymous names, it is insufficient to use the compounds’ names to organize extracted results from different studies. To overcome this situation, we used SMILES string representation to store the chemical information of the compounds. However, varying SMILES generation algorithms also lead to inconsistent results. To address this, the MolStandardize module in the Python RDKit library ^5^ was used to standardize the molecular SMILES structures, yielding RDKit canonical SMILES that allowed for deduplication and identification of compounds. Moreover, IC_50_ values in mass per volume (m/V) were converted to the standard molar concentration (C_M_/V). The ExactMolWt function from RDKit was employed to calculate the molecular weight of each compound and the acarbose control. The code and the original datasets used in this systematic review are available online at: <https://github.com/MedChemUMP/FDIGA>.


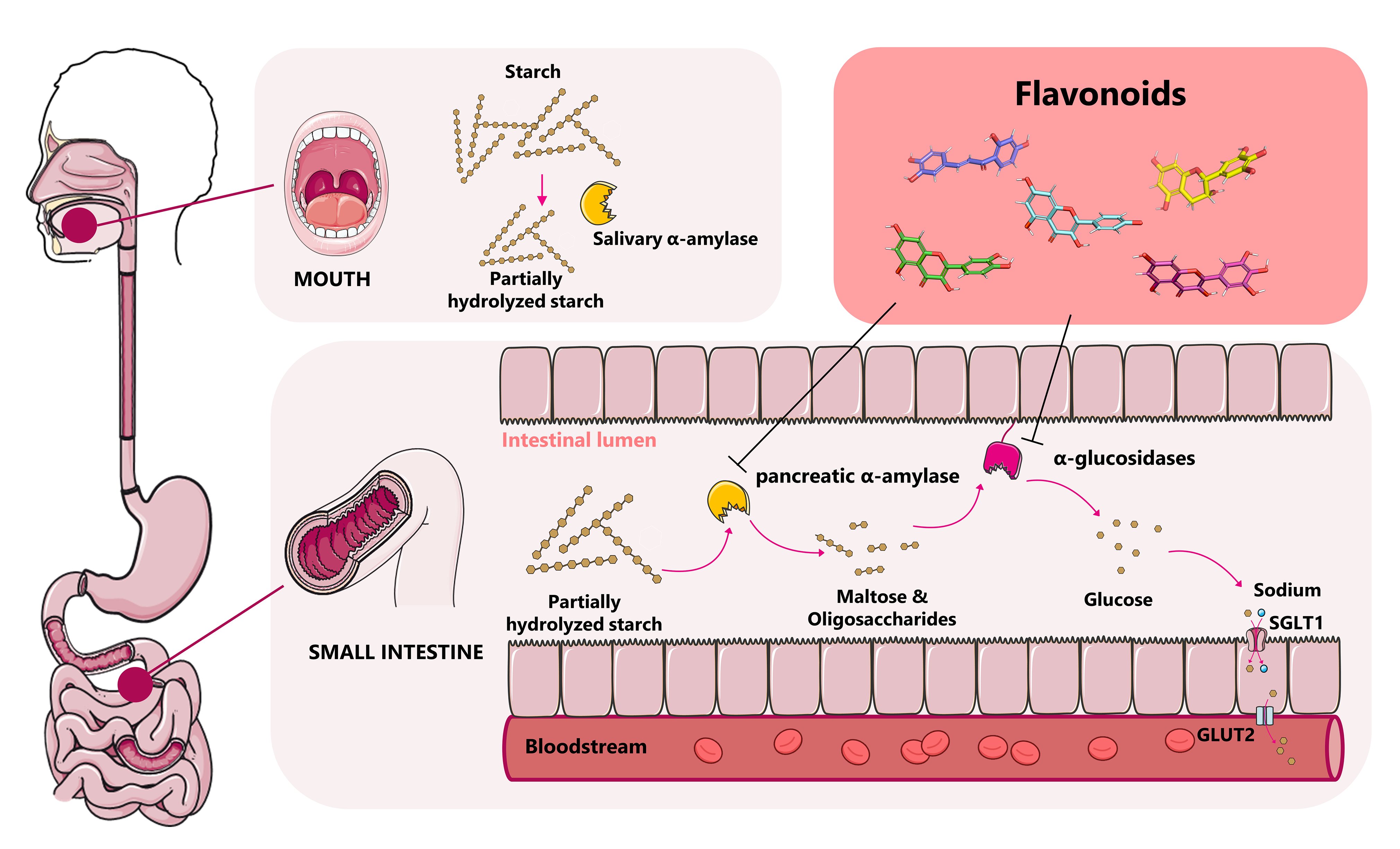


# Figure S1. Schematic illustration of starch hydrolysis pathway in humans and potential mechanisms of action of flavonoids as anti-diabetic agents. The figure was partly generated using Servier Medical Art, provided by Servier, licensed under a Creative Commons Attribution 3.0 unported license.


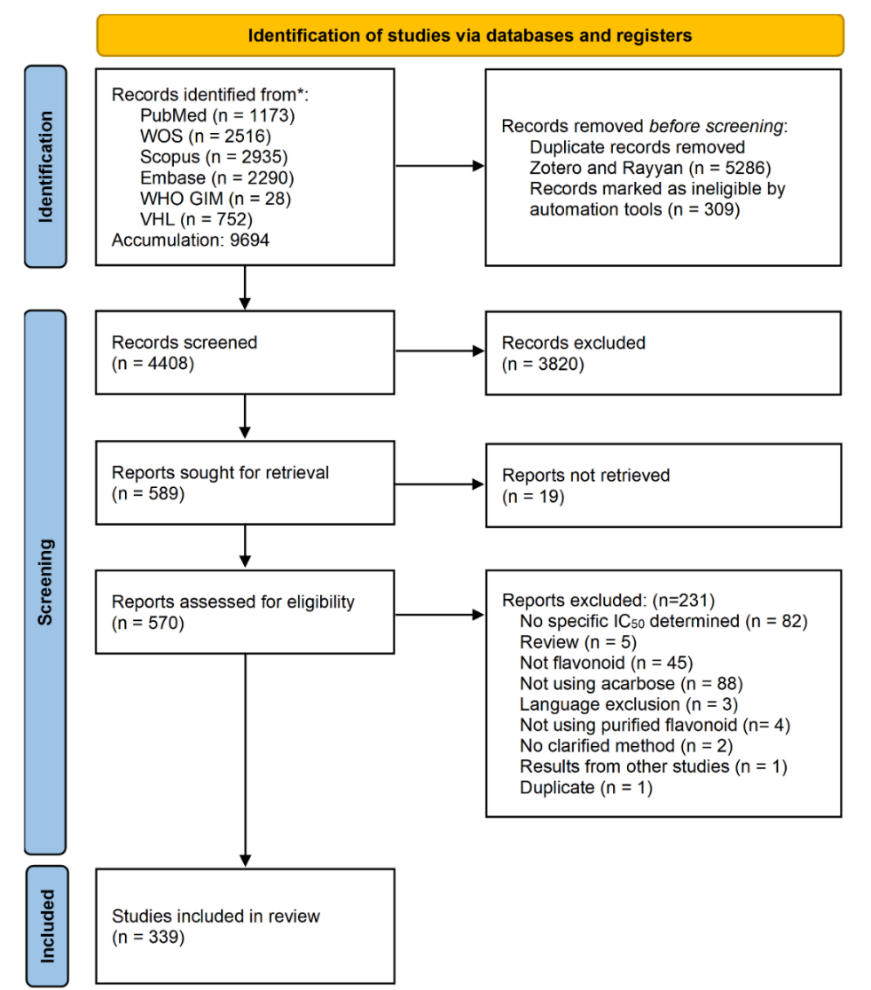


# Figure S2. PRISMA flow chart for the identification and screening process.


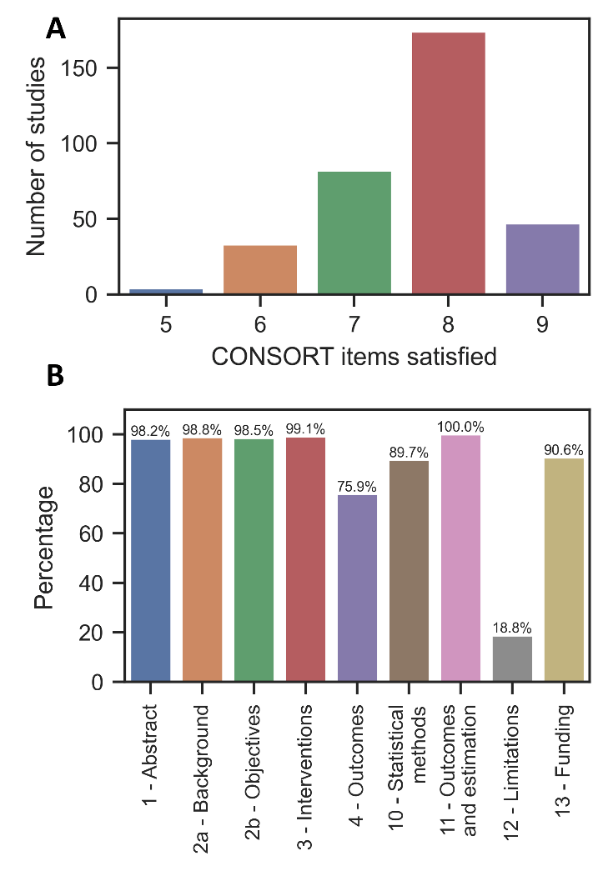


# Figure S3. Result of quality assessment using the modified CONSORT checklist. (A) Overall scores of the included studies and (B) the percentage of studies that satisfied each item in the checklist.

# Table S1. PRISMA checklist

| **Section and Topic** | **Item #** | **Checklist item** | **Location where item is reported** |
| --- | --- | --- | --- |
| **TITLE** | | |  |
| Title | 1 | Identify the report as a systematic review. | 1 |
| **ABSTRACT** | | |  |
| Abstract | 2 | See the PRISMA 2020 for Abstracts checklist. | 1 (See below) |
| **INTRODUCTION** | | |  |
| Rationale | 3 | Describe the rationale for the review in the context of existing knowledge. | 2-3 |
| Objectives | 4 | Provide an explicit statement of the objective(s) or question(s) the review addresses. | 3 |
| **METHODS** | | |  |
| Eligibility criteria | 5 | Specify the inclusion and exclusion criteria for the review and how studies were grouped for the syntheses. | SI (S3) |
| Information sources | 6 | Specify all databases, registers, websites, organisations, reference lists and other sources searched or consulted to identify studies. Specify the date when each source was last searched or consulted. | SI (S3) |
| Search strategy | 7 | Present the full search strategies for all databases, registers and websites, including any filters and limits used. | SI and Table S2 |
| Selection process | 8 | Specify the methods used to decide whether a study met the inclusion criteria of the review, including how many reviewers screened each record and each report retrieved, whether they worked independently, and if applicable, details of automation tools used in the process. | SI (S3) |
| Data collection process | 9 | Specify the methods used to collect data from reports, including how many reviewers collected data from each report, whether they worked independently, any processes for obtaining or confirming data from study investigators, and if applicable, details of automation tools used in the process. | SI (S3) |
| Data items | 10a | List and define all outcomes for which data were sought. Specify whether all results that were compatible with each outcome domain in each study were sought (e.g. for all measures, time points, analyses), and if not, the methods used to decide which results to collect. | SI (S4) |
|  | 10b | List and define all other variables for which data were sought (e.g. participant and intervention characteristics, funding sources). Describe any assumptions made about any missing or unclear information. | SI (S4) |
| Study risk of bias assessment | 11 | Specify the methods used to assess risk of bias in the included studies, including details of the tool(s) used, how many reviewers assessed each study and whether they worked independently, and if applicable, details of automation tools used in the process. | SI (S4) |
| Effect measures | 12 | Specify for each outcome the effect measure(s) (e.g. risk ratio, mean difference) used in the synthesis or presentation of results. | SI (S4) |
| Synthesis methods | 13a | Describe the processes used to decide which studies were eligible for each synthesis (e.g. tabulating the study intervention characteristics and comparing against the planned groups for each synthesis (item #5)). | SI (S4) |
|  | 13b | Describe any methods required to prepare the data for presentation or synthesis, such as handling of missing summary statistics, or data conversions. | SI (S4) |
|  | 13c | Describe any methods used to tabulate or visually display results of individual studies and syntheses. | SI (S4) |
|  | 13d | Describe any methods used to synthesize results and provide a rationale for the choice(s). If meta-analysis was performed, describe the model(s), method(s) to identify the presence and extent of statistical heterogeneity, and software package(s) used. | N/A |
|  | 13e | Describe any methods used to explore possible causes of heterogeneity among study results (e.g. subgroup analysis, meta-regression). | N/A |
|  | 13f | Describe any sensitivity analyses conducted to assess robustness of the synthesized results. | N/A |
| Reporting bias assessment | 14 | Describe any methods used to assess risk of bias due to missing results in a synthesis (arising from reporting biases). | N/A |
| Certainty assessment | 15 | Describe any methods used to assess certainty (or confidence) in the body of evidence for an outcome. | N/A |
| **RESULTS** | | |  |
| Study selection | 16a | Describe the results of the search and selection process, from the number of records identified in the search to the number of studies included in the review, ideally using a flow diagram. | Figure S2 |
|  | 16b | Cite studies that might appear to meet the inclusion criteria, but which were excluded, and explain why they were excluded. | N/A |
| Study characteristics | 17 | Cite each included study and present its characteristics. | Table S4 |
| Risk of bias in studies | 18 | Present assessments of risk of bias for each included study. | Table S5 |
| Results of individual studies | 19 | For all outcomes, present, for each study: (a) summary statistics for each group (where appropriate) and (b) an effect estimate and its precision (e.g. confidence/credible interval), ideally using structured tables or plots. | N/A |
| Results of syntheses | 20a | For each synthesis, briefly summarise the characteristics and risk of bias among contributing studies. | Table S6-S14 |
|  | 20b | Present results of all statistical syntheses conducted. If meta-analysis was done, present for each the summary estimate and its precision (e.g. confidence/credible interval) and measures of statistical heterogeneity. If comparing groups, describe the direction of the effect. | N/A |
|  | 20c | Present results of all investigations of possible causes of heterogeneity among study results. | N/A |
|  | 20d | Present results of all sensitivity analyses conducted to assess the robustness of the synthesized results. | N/A |
| Reporting biases | 21 | Present assessments of risk of bias due to missing results (arising from reporting biases) for each synthesis assessed. | N/A |
| Certainty of evidence | 22 | Present assessments of certainty (or confidence) in the body of evidence for each outcome assessed. | N/A |
| **DISCUSSION** | | |  |
| Discussion | 23a | Provide a general interpretation of the results in the context of other evidence. | 20-25 |
|  | 23b | Discuss any limitations of the evidence included in the review. | 24 |
|  | 23c | Discuss any limitations of the review processes used. | 25 |
|  | 23d | Discuss implications of the results for practice, policy, and future research. | 25 |
| **OTHER INFORMATION** | | |  |
| Registration and protocol | 24a | Provide registration information for the review, including register name and registration number, or state that the review was not registered. | SI (S3) |
|  | 24b | Indicate where the review protocol can be accessed, or state that a protocol was not prepared. | 26 |
|  | 24c | Describe and explain any amendments to information provided at registration or in the protocol. | N/A |
| Support | 25 | Describe sources of financial or non-financial support for the review, and the role of the funders or sponsors in the review. | 26 |
| Competing interests | 26 | Declare any competing interests of review authors. | 26 |
| Availability of data, code and other materials | 27 | Report which of the following are publicly available and where they can be found: template data collection forms; data extracted from included studies; data used for all analyses; analytic code; any other materials used in the review. | 26 |

**(PRISMA Abstract checklist)**

| **Section and Topic** | **Item #** | **Checklist item** | **Reported (Yes/No)** |
| --- | --- | --- | --- |
| **TITLE** | | |  |
| Title | 1 | Identify the report as a systematic review. | Yes |
| **BACKGROUND** | | |  |
| Objectives | 2 | Provide an explicit statement of the main objective(s) or question(s) the review addresses. | Yes |
| **METHODS** | | |  |
| Eligibility criteria | 3 | Specify the inclusion and exclusion criteria for the review. | Yes |
| Information sources | 4 | Specify the information sources (e.g. databases, registers) used to identify studies and the date when each was last searched. | Yes |
| Risk of bias | 5 | Specify the methods used to assess risk of bias in the included studies. | Yes |
| Synthesis of results | 6 | Specify the methods used to present and synthesise results. | No |
| **RESULTS** | | |  |
| Included studies | 7 | Give the total number of included studies and participants and summarise relevant characteristics of studies. | Yes |
| Synthesis of results | 8 | Present results for main outcomes, preferably indicating the number of included studies and participants for each. If meta-analysis was done, report the summary estimate and confidence/credible interval. If comparing groups, indicate the direction of the effect (i.e. which group is favoured). | Yes |
| **DISCUSSION** | | |  |
| Limitations of evidence | 9 | Provide a brief summary of the limitations of the evidence included in the review (e.g. study risk of bias, inconsistency and imprecision). | No |
| Interpretation | 10 | Provide a general interpretation of the results and important implications. | Yes |
| **OTHER** | | |  |
| Funding | 11 | Specify the primary source of funding for the review. | No |
| Registration | 12 | Provide the register name and registration number. | No |

# Table S2. Details of search terms and the number of records curated in each database as of August 21, 2022

|  | **Databases** | **Search Terms** | | **Results** |
| --- | --- | --- | --- | --- |
| **1** | **PubMed** | (("alpha-Glucosidases"[Mesh] OR Maltase OR sucrase OR "alpha-amylases"[Mesh] OR "alpha-amylase")) AND ((flavonoids[MeSH Terms]) OR flavonoid[Title/Abstract]) | | **1173** |
| **2** | **WOS** | TI=((alpha-glucosidase OR alpha-amylase OR maltase OR sucrase) AND (flavonoid OR flavonoids)) OR AB=((alpha-glucosidase OR alpha-amylase OR maltase OR sucrase) AND (flavonoid OR flavonoids)) | | **2516** |
| **3** | **SCOPUS** | TITLE-ABS-KEY(alpha-glucosidase OR alpha-amylase OR maltase OR sucrase) AND TITLE-ABS-KEY(flavonoid OR flavonoids) | | **2935** |
| **4** | **WHO GIM** | tw:((("alpha glucosidase") OR ("alpha glucosidases") OR (maltase) OR (maltases) OR (sucrase) OR (sucrases) OR ("alpha amylase") OR ("alpha amylases")) AND (mh:(flavonoid) OR flavonoid OR flavonoids)) | | **28** |
| **5** | **VHL** | (("alpha glucosidase") OR ("alpha glucosidases") OR (maltase) OR (maltases) OR (sucrase) OR (sucrases) OR ("alpha amylase") OR ("alpha amylases")) AND (mh:(flavonoid) OR flavonoid OR flavonoids) | | **752** |
| **6** | **EMBASE** | 1. exp alpha glucosidase/ | 10,327 | **2290** |
|  |  | 2. exp amylase/ | 42,376 |  |
|  |  | 3. maltase.mp. [mp=title, abstract, heading word, drug trade name, original title, device manufacturer, drug manufacturer, device trade name, keyword heading word, floating subheading word, candidate term word] | 2,928 |  |
|  |  | 4. sucrase.mp. [mp=title, abstract, heading word, drug trade name, original title, device manufacturer, drug manufacturer, device trade name, keyword heading word, floating subheading word, candidate term word] | 4,226 |  |
|  |  | 5. 1 or 2 or 3 or 4 | 54,381 |  |
|  |  | 6. exp flavonoid/ | 180,801 |  |
|  |  | 7. 5 and 6 | 2,290 |  |
| **Total** | | | | **9,694** |

# Table S3. Details of search terms and the number of complex structures examined on RCSB Protein Data Bank as of April 27 2023

|  | **Enzyme** | **E.C. number (Search term)** | **Number of structures queried** | **Structures that co-crystallized with flavonoid derivatives** |
| --- | --- | --- | --- | --- |
| **1** | **α-amylase** | 3.2.1.1 | 212 | 4GQR, 4W93, 5E0F, 6OCN, 6OBX |
| **2** | **maltase** | 3.2.1.20 | 80 | N/A |
| **3** | **α-gluco-amylase** | 3.2.1.3 | 46 | N/A |
| **4** | **isomaltase** | 3.2.1.10 | 16 | N/A |
| **5** | **sucrase** | 3.2.1.48 | 6 | N/A |

# Table S4. Detailed characteristics of included studies

| **ID** | **Author (Year)** | **Enzyme**  **(origin; assay method)** | **Compound origin** | **Compounds: subgroups of flavonoids;**  **Range of IC_50_:**  **Best compound: IC_50_ Mean ± SD**  **Positive control: IC_50_ Mean ± SD** |
| --- | --- | --- | --- | --- |
| 1 | Yang Yao *et al.* (2011)^6^ | α-glucosidase (Rat intestinal; Chromogenic method) | *Vigna angularis* | 2 flavonoids: flavone  IC_50_ range: 0.4-4.8 mg/mL  Vitexin: 0.4 mg/mL  Acarbose: 0.45 mg/mL |
| 2 | Van Thanh *et al.* (2022)^7^ | α-glucosidase (*Saccharomyces cerevisiae;* Chromogenic method)  α-amylase (*Aspergillus oryzae;* Iodinemetric method) | Dysosma difformis | 4 flavonoids: flavanonol; flavonol.  **AG:**  IC_50_ range: 1057-1962 µM  8,2'-diprenylquercetin 3-methyl ether: 1057 ± 82 µM  Acarbose: 1020 ± 85 µM  **AM:**  IC_50_ range: 857.8-1330 µM  8,2'-diprenylquercetin 3-methyl ether: 857.8 ± 78.3 µM  Acarbose: 623.9 ± 60.2 µM |
| 3 | El-Nashar, H. A. S. *et al.* (2022)^8^ | α-amylase (unstated*;* Reducing sugar method) | *Schinus polygama* | 1 flavonoid: oligomeric flavonoid  luteolin-(6🠖8'')-apigenin: 64.7 µg/mL  Acarbose: 34.71 µg/mL |
| 4 | Jia, C. C. *et al.* (2017)^9^ | α-glucosidase (*Saccharomyces cerevisiae;* Chromogenic method) | *Garcinia paucinervis* | 1 flavonoid: oligomeric flavonoid  Paucinervin K: 12.48 ± 4.60 µM  Acarbose: 2.88 ± 0.85 µM |
| 5 | Nguyen, Tan Phat *et al.* (2016)^10^ | α-glucosidase (*Saccharomyces cerevisiae;* Chromogenic method) | *Pandanus tectorius* | 2 flavonoids: flavone; flavanone.  IC_50_ range: 14.4-22.1 µM  Luteolin: 14.4 µM  Acarbose: 214.5 µM |
| 6 | Zhang, Y. *et al.* (2013)^11^ | α-glucosidase (*Saccharomyces cerevisiae;* Chromogenic method) | *Euonymus alatus* | 6 flavonoids: flavan-3-ol; flavonol; oligomeric  IC_50_ range: 23-119.1 µM  Kaempferitrin: 23 µM  Acarbose: 679.7 µM |
| 7 | Wu, S. *et al.* (2019)^12^ | α-glucosidase (*Saccharomyces cerevisiae;* Chromogenic method)  α-amylase (Porcine pancreatic*;* Chromogenic method) | *Punica granatum* (3)  Commercial (2) | 5 flavonoids: Flavone  **AG:**  IC_50_ range: 7.83-34.37 mM  Tricetin: 7.83 mM  Acarbose: 6.14 mM  **AM:**  IC_50_ range: 1.42-144.9 mM  Tricetin: 1.42 mM  Acarbose: 0.058 mM |
| 8 | Thuy, N. T. L. *et al.* (2019)^13^ | α-glucosidase (*Saccharomyces cerevisiae;* Chromogenic method) | *Lumnitzera littorea* | 10 flavonoids: Flavone, flavonol, flavanone.  IC_50_ range: 1.9-153.5 µg/mL  Naringenin: 1.9 µg/mL  Acarbose: 138.2 µg/mL |
| 9 | Nguyen , T. T. H. *et al.* (2021)^14^ | α-glucosidase (*Saccharomyces cerevisiae;* Chromogenic method) | *Garcinia mckeaniana* | 5 flavonoids: flavone, oligomeric, flavonol  IC_50_ range: 27.39-210.16 µM  Quercetin: 27.39 µM  Acarbose: 249 µM |
| 10 | Liu, Y. *et al.*  (2020)^15^ | α-glucosidase (Rat intestinal; Chromogenic method) | *Scutellaria barbata* | 1 flavonoid: flavone  Acacetin 7-*O*-β-D-galactopyranosyl(1→2)-β-D-glucopyranoside: 8.9 µM  Acarbose: 14.6 µM |
| 11 | Zhang, X. H. *et al.* (2020)^16^ | α-glucosidase (Rat intestinal; Chromogenic method) | *Galium verum* | 1 flavonoid: flavone  Apigenin 7-*O*-α-L-2,4-Di-*O*-acetylrhamnopyranosyl-(1→6)-β-D-glucopyranoside: 13.7 µM  Acarbose: 14.6 µM |
| 12 | Phuong, N. H. *et al.* (2017)^17^ | α-glucosidase (*Saccharomyces cerevisiae;* Chromogenic method) | *Lumnitzera racemosa* | 4 flavonoids: flavonol  IC_50_ range: 1.3-179.5 µM  Quercetin: 1.3 µM  Acarbose: 214.5 µM |
| 13 | Zhou, Q. *et al.*  (2022)^18^ | α-glucosidase (*Saccharomyces cerevisiae;* Chromogenic method) | *Ficus tikoua* | 8 flavonoids: flavonol; flavanone; flavanonol; isoflavonoid  IC_50_ range: 3.43 - 46.42 µM  Ficusin A: 3.43 ± 0.15 µM  Acarbose: 33.93 ± 0.02 µM |
| 14 | Helal, I. E. *et al.* (2022)^19^ | α-glucosidase (*Saccharomyces cerevisiae;* Chromogenic method) | *Dracaena cinnabari* | 5 flavonoids: flavan; flavanone; chalcone; dihydrochalcone  IC_50_ range: 203.6 – 423.8 µM  7-hydroxyflavanone: 203.6 ± 3.0µM  Acarbose: 47.3 ± 2.3 µM |
| 15 | Parveen, A. *et al.* (2020)^20^ | α-glucosidase (Unstated*;* Chromogenic method) | *Nigella sativa* | 5 flavonoids: flavonol  IC_50_ range: 214.5-360 µM  Kaempferol-3-rutinoside 214.5 µM  Acarbose: 127.9 µM |
| 16 | Li, K. *et al.*  (2020)^21^ | α-glucosidase (*Saccharomyces cerevisiae;* Chromogenic method) | *Panax ginseng* | 6 flavonoids: flavonol  IC_50_ range: 5.48-482.1 µM  Afzelin 5.48 ± 1.05 µM  Acarbose: 385.2 ± 18.6 µM |
| 17 | Kim, T. H. (2016)^22^ | α-glucosidase (*Saccharomyces cerevisiae;* Chromogenic method) | *Uncaria gambir* | 5 flavonoids: Flavonol; oligomeric, flavan-3-ol  IC_50_ range: 17.3 – 213.7 µM  Procyanidin B3: 17.3 ± 1.00 µM  Acarbose: 312.6 ± 3.8 µM |
| 18 | Rocha, S. *et al.* (2019)^23^ | α-glucosidase (*Saccharomyces cerevisiae;* Chromogenic method)  α-amylase (Porcine pancreatic*;* Chromogenic method) | Commercial/synthesize | **AG:**  3 flavonoids: chalcone  IC_50_ range: 21 – 87 µM  Butein: 21 ± 2 µM  Acarbose: 357 ± 25 µM  **AM:**  1 flavonoid: chalcone  Butein: 62 ± 4 µM  Acarbose: 1.1 ± 0.2 µM |
| 19 | Wan, C. *et al.*  (2013)^24^ | α-glucosidase (yeast*;* Chromogenic method) | *Vaccinium corymbosum* | 1 flavonoid: flavonol  Isorhamnetin-3-*O*-(6''-*O*-coumaroyl)-β-D-glucoside: 80.9 ± 11.3 µM  Acarbose: 200.7 ± 19.7 µM |
| 20 | Kashchenko, N. I. *et al.* (2018)^25^ | α-amylase (*Aspergillus niger;* Enzymatic method) | *Spirae* genus | 6 flavonoids: flavonol  IC_50_ range: 46.18 – 182.16 µg/ml  6"-*O*-caffeoylhyperoside: 46.18 ± 0.92 µg/ml  Acarbose: 154.35 ± 3.08 µg/ml |
| 21 | Lee, Shoei-Sheng *et al.*(2008)^26^ | α-glucosidase (*Bacillus stearothermophilus;* Chromogenic method) | *Machilus philippinensis* | 4 flavonoids: flavonol  IC_50_ range: 1.00 – 228.11 µM  kaempferol-3-*O*-a-L-(3’’-*E*,4’’-*Z*-di-p-coumaroyl)-rhamnopyranoside: 1.00 ± 0.01 µM  Acarbose: 0.046 ± 0.01 µM |
| 22 | Chang, C. C. *et al.* (2015)^27^ | α-glucosidase (*Bacillus stearothermophilus;* Chromogenic method) | *Tinospora crispa* | 10 flavonoids: flavone  IC_50_ range: 4.30 – 1525.7 µM  isovitexin 2"-(*E*)-p-coumarate: 4.30 ± 1.40 µM  Acarbose: 0.033 ± 0.006 µM |
| 23 | Habtemariam, Solomon (2011)^28^ | α-glucosidase (*Saccharomyces cerevisiae;* Chromogenic method) | Commercial | 3 flavonoids: flavonol  IC_50_ range: 19.36 – 42.59 µM  Kaempferol-3-*O*-rutinoside: 19.36 ± 2.43 µM  Acarbose: 177.5 ± 27.5 µM |
| 24 | Wang,H. *et al.* (2010)^29^ | α-glucosidase (rat sucrase*;* Enzymatic method)  α-amylase (porcine pancreatic*;* Chromogenic method) | *Psidium guajava* | **AG**:  6 flavonoids: flavonol  IC_50_ range: 3.0 – 7.5 mM  Myricetin: 3.0 ± 0.1 mM  Acarbose: 2.5 ± 0.1 mM  **AM:**  6 flavonoids: flavonol  IC50 range: 4.3 – 6.1 mM  Myricetin: 4.3 ± 0.3 mM  Acarbose: 2.3 ± 0.1 mM |
| 25 | Etsassala, N. G. E. R. *et al.* (2020)^30^ | α-glucosidase (*Saccharomyces cerevisiae;* Chromogenic method) | *Salvia aurita* | 1flavonoid: flavanone  4,7-dimethylapigenin ether: 28.7 ± 0.9 µg/mL  Acarbose: 610.4 ± 1.0 µg/mL |
| 26 | Hussain, Munawar *et al.* (2019)^31^ | α-glucosidase (*Saccharomyces cerevisiae;* Chromogenic method) | *Abutilon pakistanicum* | 3 flavonoids: flavonol; flavanonol  IC_50_ range: 16.3 – 435 µM  Compound **4**: 16.3 ± 0.82 µM  Acarbose: 780 ± 28 µM |
| 27 | Gao, H. *et al.*  (2005)^32^ | α-glucosidase (rat sucrase*;* Enzymatic method) | Synthesis | 9 flavonoids: flavone, flavonol, flavanone, isoflavonoid  IC_50_ range: 2.4 – 640 µM  Compound 16: 2.4 µM  Acarbose: 1.8 µM |
| 28 | Gutierrez-Gonzalez, Jose A. *et al.* (2021)^33^ | α-glucosidase (*Saccharomyces cerevisiae;* Chromogenic method) | *Ageratina grandifolia* | 3 flavonoids: flavonol  IC_50_ range: 0.79 – 401.5 µM  Compound 3: 0.79 ± 0.01 µM  Acarbose: 278.73 ± 12.5 µM |
| 29 | Escandon-Rivera, S. *et al.* (2012)^34^ | α-glucosidase (*Saccharomyces cerevisiae;* Chromogenic method) | *Brickellia cavanillesii* | 2 flavonoids: flavonol  IC_50_ range: 0.16 – 0.53 mM  Isorhamnetin: 0.16 mM  Acarbose: 0.34 mM |
| 30 | Kazemi, R. *et al.* (2022)^35^ | α-glucosidase (*Saccharomyces cerevisiae;* Chromogenic method) | *Marrubium astracanicum* | 3 flavonoids: flavone  IC_50_ range: 57.4 – 926.1 µM  apigenin-7-*O*-(3′′-(*E*)-p-coumaroyl)-β-D-glucoside: 57.4 ± 1.2 µM  Acarbose: 751.2 ± 0.4 µM |
| 31 | Ha, Kim-Ngoc *et al.* (2022)^36^ | α-glucosidase (*Saccharomyces cerevisiae;* Chromogenic method) | *Nervilia concolor (2)*  *Tecoma stans (1)* | 3 flavonoids: flavone, flavonol  IC_50_ range: 35.6 – 64.7 µM  Astragalin: 35.6 ± 2.5 µM  Acarbose: 332.5 µM |
| 32 | Matsui, T. *et al.* (2001)^37^ | α-glucosidase (Rat sucrase*;* Enzymatic method) | *Pharbitis nil (2)*  *Ipomoea batatas (2)* | 4 flavonoids: anthocyanidin  IC_50_ range: 60 – 200 µM  SOA4: 60 µM  Acarbose: 0.426 µM |
| 33 | Nguyen, T. N. T. *et al.* (2022)^38^ | α-glucosidase (*Saccharomyces cerevisiae;* Chromogenic method) | *Aquilaria crassna* | 2 flavonoids: flavone  IC_50_ range: 24 – 76.5 µM  Genkwanin 5-*O*-β-D-primeveroside: 24 ± 3.6 µM  Acarbose: 105.2 ± 11.7 µM |
| 34 | Joycharat, N. *et al.* (2018)^39^ | α-glucosidase (yeast*;* Chromogenic method) | *Albizia myriophylla* | 2 flavonoids: flavone; flavan-3,4-diol  IC_50_ range: 98.59 – 195.21 µg/mL  3,4,7,3'-Tetrahydroxyflavan: 98.59 µg/mL  Acarbose: 125.00 µg/mL |
| 35 | Vi, L. N. T. *et al.*  (2022)^40^ | α-glucosidase (Unstated*;* Chromogenic method) | *Ruellia tuberosa* | 1 flavonoid: flavone  Apigenin: 20.1 ± 0.3 µg/mL  Acarbose: 127.7 ± 0.2 µg/mL |
| 36 | Nguyen, P. T. M. *et al.* (2019)^41^ | α-glucosidase (*Saccharomyces cerevisiae;* Chromogenic method) | *Smilax glabra* | 1 flavonoid: flavanonol  Astilbin: 125 ± 3.11 µg/mL  Acarbose: 525 ± 1.72 µg/mL |
| 37 | Dej-Adisai, S. *et al.* (2021)^42^ | α-glucosidase (*Saccharomyces cerevisiae;* Chromogenic method) | *Bauhinia pulla* | 6 flavonoids: flavonol; flavone; chalcone  IC_50_ range: 5.41 – 1273.31 µg/mL  Quercetin: 5.41 µg/mL  Acarbose: 124.11 µg/mL |
| 38 | Tabussum, A. *et al.* (2013)^43^ | α-glucosidase (*Saccharomyces cerevisiae;* Chromogenic method) | *Chrozophora plicata* | 2 flavonoids: flavone  IC_50_ range: 111.23 – 287.12 µM  Plicatanoside: 111.23 ± 0.65 µM  Acarbose: 38.25 ± 0.12 µM |
| 39 | Lima Júnior, J. P. D. *et al.* (2021)^44^ | α-amylase (human salivary*;* Chromogenic method) | *Anacardium humile* | 2 flavonoids: flavonol, flavan-3-ol  IC_50_ range: 0.09 – 0.13 µg/mL  Catechin: 0.09 µg/mL  Acarbose: 0.08 µg/mL |
| 40 | Xie, H. *et al.*  (2020)^45^ | α-glucosidase (yeast*;* Chromogenic method) | *Elaeagnus angustifolia* | 2 flavonoids: oligomeric, flavonol  IC_50_ range: 4.4-30.5 µM  Angustifolinoid B: 4.4 µM  Acarbose: 65.8 µM |
| 41 | Homoki, J. R. *et al.* (2016)^46^ | α-amylase (human salivary*;* Chromogenic method) | Commercial | 4 flavonoids: anthocyanidin  IC_50_ range: 80-675 µM  Malvidin-3,5-diglycoside: 80 ± 10 µM  Acarbose: 0.5 µM |
| 42 | Swilam, N. *et al.* (2022)^47^ | α-amylase (unstated*;* Reducing sugar method) | *Ammannia baccifera* | 3 flavonoids: flavonol  IC_50_ range: 157.54 – 223.6 µg/mL  MGGG: 157.54 ± 5.9 µg/mL  Acarbose: 71.85 ± 3.70 µg/mL |
| 43 | Wang, Y. *et al.* (2013)^48^ | α-glucosidase (*Saccharomyces cerevisiae;* Chromogenic method) | *Morus alba* | 14 flavonoids: flavonol, flavanone, flavanonol, chalcone  IC_50_ range: 8.57 – 932.51 µM  Quercetin: 8.57 µM  Acarbose: 119.15 µM |
| 44 | Gulçin, I. *et al.* (2018)^49^ | α-glucosidase (*Saccharomyces cerevisiae;* Chromogenic method)  α-amylase (porcine pancreatic*;* Iodinemetric method) | Commercial | 4 flavonoids: dihydrochalcone; flavone  **AG:**  IC_50_ range: 324.28 – 679.41 nM  Eupatilin: 324.28 nM  Acarbose: 22.800 nM  **AM:**  IC_50_ range: 175.01 – 295.27 nM  Eupatorin: 175.01 nM  Acarbose: 10.000 nM |
| 45 | Junejo, J. A. *et al.* (2021)^50^ | α-glucosidase (Unstated*;* Chromogenic method)  α-amylase (Unstated*;* Reducing sugar method) | *Tetrastigma angustifolia* | 1 flavonoid: flavone  **AG:**  8-hydroxyapigenin 7-*O*-β-D-glucopyranoside: 58.05 µg/mL  Acarbose: 30.01 µg/mL  **AM:**  8-hydroxyapigenin 7-*O*-β-D-glucopyranoside: 51.45 µg/mL  Acarbose: 28.72 µg/mL |
| 46 | Varghese, G. K. *et al.* (2013)^51^ | α-glucosidase (*Saccharomyces cerevisiae;* Chromogenic method) | *Cassia alata* | 2 flavonoids: flavonol  IC_50_ range: 50 – 56.7 µM  Kaempferol 3-*O*-gentibioside: 50 ± 8.5 µM  Acarbose: 166.21 ± 19.06 µM |
| 47 | Zhu, G. *et al.*  (2019)^52^ | α-glucosidase (unstated*;* Chromogenic method) | *Psoralea corylifolia* | 7 flavonoids: aurone, flavanone, isoflavonoid  IC_50_ range: 28 – 179.4 µM  Bavachinone B: 28 µM  Acarbose: 214.8 µM |
| 48 | El Ridhasya, F. *et al.* (2020)^53^ | α-glucosidase (unstated*;* Chromogenic method) | *Helminthostachys zeylanica* | 2 flavonoids: flavone  IC­_50_ range: 138.21 – 273.13 µg/ml  Ugonin K: 138.21 ± 0.263 µg/ml  Acarbose: 19.73 ± 0.342 µg/ml |
| 49 | Ablat, A. *et al.* (2017)^54^ | α-glucosidase (unstated*;* Chromogenic method) | *Brucea javanica* | 1 flavonoid: flavone  Luteolin: 26.41 ± 0.04 µM  Acarbose: 145.83 ± 0.03 µM |
| 50 | Laishram, S. *et al.* (2015)^55^ | α-glucosidase (yeast*;* Chromogenic method)  α-amylase (porcine pancreatic*;* Reducing sugar method) | *Cycas pectinata* | 2 flavonoids: oligomeric  **AG:**  IC­_50_ range: 8.09 – 9.77 µM  Amentoflavone: 8.09 ± 0.023 µM  Acarbose: 540.36 ± 25.51 µM  **AM:**  IC­_50_ range: 39.69 – 73.6 µM  2,3-dihydroamentoflavone: 39.69 ± 0.39 µM  Acarbose: 17.9 ± 0.62 µM |
| 51 | Jo, Y. H. *et al.*  (2021)^56^ | α-glucosidase (*Saccharomyces cerevisiae;* Chromogenic method) | *Masclura tricuspidata* | 32 flavonoids: isoflavonoid  IC­_50_ range: 3.2 – 23.5 µM  millewanin G: 3.2 µM  Acarbose: 78.2 µM |
| 52 | Yoshikawa, M.  *et al.* (1998)^57^ | α-glucosidase (Rat maltase*;* Enzymatic method) | *Myrcia multiflora* | 4 flavonoids: flavanone, flavonol  IC­_50_ range: 240 – 600 µM  Desmanthin-1: 240 µM  Acarbose: 2 µM |
| 53 | Demir, Y. *et al.* (2019)^58^ | α-glucosidase (unstated*;* Chromogenic method)  α-amylase (unstated*;* Iodinemetric method) | Commercial | 5 flavonoids: isoflavonoid, flavone, anthocyanidin  **AG:**  IC­_50_ range: 165.51 – 506.20 nM  Genistein: 165.51 nM  Acarbose: 2.800 nM  **AM:**  IC­_50_ range: 601.56 – 2067.78 nM  Formononetin: 601.56 nM  Acarbose: 10.000 nM |
| 54 | Ahmed, S. *et al.* (2019)^59^ | α-glucosidase (unstated*;* Chromogenic method)  α-amylase (unstated*;* Reducing sugar method) | *Euphorbia schimperi* | 1 flavonoid: flavonol  **AG:**  Quercetin-3-*O*-glucuronide: 89.20 ± 9.20 µg/mL  Acarbose: 52.40 ± 4.60 µg/mL  **AM:**  Quercetin-3-*O*-glucuronide: 128.34 ± 12.30 µg/mL  Acarbose: 64.20 ± 5.60 µg/mL |
| 55 | Qun, Sun *et al.*  (2017)^60^ | α-glucosidase (*Saccharomyces cerevisiae*, Chromogenic method) | *Gnaphalium hypoleucum* | 3 flavonoids: flavone, flavanol  IC­_50_ range: 6.83 - 21.84 μM  Luteolin-4'-*O*-β-D-(6’’-E-caffeoyl)-glucopyranoside: 6.83 ± 0.54 μM  Acarbose: 7.69 ± 0.47 μM |
| 56 | Bui, T. T. *et al.*  (2022)^61^ | α-glucosidase (*Saccharomyces cerevisiae*, Chromogenic method) | *Bruguiera parviflora* | 5 flavonoids: flavanonol; flavonol  IC­_50_ range: 3.4 - 97.8 μg/mL  Quercetin: 3.4 ± 0.5 μg/mL  Acarbose: 127.7 ± 0.2 μg/mL |
| 57 | Hlila, Malek Besbes *et al.*  (2017)^62^ | α-glucosidase (*Aspergillus niger*, Chromogenic method) | *Scabiosa arenaria* | 2 flavonoids: flavone  IC­_50_ range: 0.0092 - 0.0140 mg/mL  Luteolin: 0.0092 mg/mL  Acarbose: 0.2800 mg/mL |
| 58 | Vu, N. K. *et al.*  (2020)^63^ | α-glucosidase (unstated, Chromogenic method) | *Allium cepa* | 13 flavonoids: flavonol, flavanonol, oligomeric  IC­_50_ range: 0.89 - 74.44 μM  1,3,11a-trihydroxy-9-(3,5,7-trihydroxy-4*H*-1-benzopyran-4-on-2-yl)-5a-[1,3,11a-trihydroxy-5a-(3,4-dihydroxyphenyl)-5,6,11-hexahydro-5,6,11-trioxanaphthacene-12-on-9-yl]-5,6,11-hexahydro-5,6,11-trioxanaphthacene-12-one (15): 0.89 ± 0.01 μM  Acarbose: 257.41 ± 0.57 μM |
| 59 | Fan, P. *et al.*  (2010)^64^ | α-glucosidase (*Saccharomyces cerevisiae*, Chromogenic method) | *Polygonum sachalinensis* | 1 flavonoid: flavonol  Quercetin-3-*O*-β-D-galactopyranoside: 0.201 mM  Acarbose: 0.375 mM |
| 60 | Choi, C. I. *et al.*  (2015)^65^ | α-glucosidase (unstated, Chromogenic method) | *Euonymus alatus* | 2 flavonoids: flavonol, flavanone  IC­_50_ range: 96.8 - 107.8 μM  Narigenin: 96.8 ± 6.2 μM  Acarbose: 310.2 ± 12.2 μM |
| 61 | Yue, Y. *et al.*  (2018)^66^ | α-glucosidase (*Saccharomyces cerevisiae*, Chromogenic method) | Commercial | 1 flavonoid: flavonol  Fisetin: 9.38 ± 0.35 μg/mL  Acarbose: 1.07 ± 0.15 mg/mL |
| 62 | Choi, C. I. *et al.*  (2016)^67^ | α-glucosidase (*Saccharomyces cerevisiae*, Chromogenic method) | *Lactuca indica* | 2 flavonoids: flavone  IC­_50_ range: 96.4 - 100.7 μM  Apigenin: 96.4 ± 0.1 μM  Acarbose: 310.2 ± 12.2 μM |
| 63 | Wan, C. *et al.*  (2012)^68^ | α-glucosidase (yeast, Chromogenic method) | Highbush blueberry flowers | 11 flavonoids: flavonol, flavan-3-ol  IC­_50_ range: 28.89 - 137.47 μM  Catechin-[8,7-*e*]-4β-(3,4-dihydroxy-phenyl)-dihydro-2(3*H*)-pyranone (21): 28.89 ± 2.48 μM  Acarbose: 200.68 ± 19.63 μM |
| 64 | Wang, Y. *et al.* (2021)^69^ | α-glucosidase (*Saccharomyces cerevisiae*, Chromogenic method) | *Livistona chinensis* | 5 flavonoids: flavan-3-ol, flavone  IC­_50_ range: 24.03 - 98.60 μM  (-)-epicatechin (4): 24.03 ± 0.38 μM  Acarbose: 61.34 ± 1.02 μM |
| 65 | Polbuppha, I. *et al.* (2017)^70^ | α-glucosidase (unstated, Chromogenic method) | *Maclura fruticosa* | 1 flavonoid: isoflavonoid  Wighteone: 0.02 ± 0.37 mM  Acarbose: 1.55 ± 0.39 mM |
| 66 | Supasuteekul, C. *et al.* (2016)^71^ | α-glucosidase (*Saccharomyces cerevisiae*, Chromogenic method) | *Garcinia gracilis* | 1 flavonoid: flavone  apigenin-8-*C*-α-L-rhamnopyranosyl-(1→2)-β-D-glucopyranoside: 0.56 ± 0.01 mM  Acarbose: 0.90 ± 0.06 mM |
| 67 | Zhao, J. Q. *et al.*  (2017)^72^ | α-glucosidase (*Saccharomyces cerevisiae*, Chromogenic method) | *Sibiraea laevigata* | 1 flavonoid: flavonol  kaempferol 3-*O*-β-D-(6"-*E*-p-coumaroyl)-glucopyranoside: 62.0 ± 9.8 μg/mL  Acarbose: 118.4 ± 12.7 μg/mL |
| 68 | Zhang, L. *et al.* (2016)^73^ | α-glucosidase (unstated, Chromogenic method) | *Ipomoea batatas* | 2 flavonoids: flavonol  IC­_50_ range: 22.38 – 432.04 μM  Quercetin-3-*O*-glucoside: 22.38 ± 1.73 μM  Acarbose: 168.95 ± 12.27 μM |
| 69 | Anh, L. T. T. *et al.*  (2022)^74^ | α-glucosidase (unstated, Chromogenic method) | *Polyscias guilfoylei* | 1 flavonoid: flavonol  Quercitrin: 2.0 μg/mL  Acarbose: 184.0 μg/mL |
| 70 | Sahnoun, M. *et al.* (2018)^75^ | α-amylase (human pancreatic; Reducing sugar method) | *Agave americana* | 1 flavonoid: flavone  Apigenin: 75.12 μM  Acarbose: 43 μM |
| 71 | Ma, J. *et al.*  (2017)^76^ | α-glucosidase (unstated, Chromogenic method) | *Aspergillus candidus* | 1 flavonoid: flavonol  Aspergivone B: 244 μg/mL  Acarbose: 457 μg/mL |
| 72 | Monteiro, A. D. O. *et al.* (2022)^77^ | α-glucosidase (unstated, Chromogenic method) | *Bauhinia pulchella* | 3 flavonoids: flavan, flavan-3-ol  IC­_50_ range: 0.51 - 3.67 μg/mL  (-)-fisetinidol: 0.51 μg/mL  Acarbose: 45.72 μg/mL |
| 73 | Song, M. *et al.*  (2022)^78^ | α-glucosidase (unstated, Chromogenic method) | *Cephalotaxus oliveri* | 6 flavonoids: oligomeric  IC­_50_ range: 1.84 - 51.16 μM  Umcephabiflovin (3): 1.84 ± 1.14 μM  Acarbose: 187.40 ± 14.81 μM |
| 74 | Tian, J. L. *et al.*  (2021)^79^ | α-glucosidase (unstated; Chromogenic method)  α-amylase (unstated; Reducing sugar method) | *Rubus corchorifolius* | 12 flavonoids: flavan-3-ol, flavone, flavonol, flavone, flavanone  **AG:**  IC­_50_ range: 4.96 - 25.41 μM  quercetagetin-7-*O*-β-D-glucopyranoside (4): 4.96 ± 0.54 μM  Acarbose: 1.93 ± 0.08 μM  **AM:**  IC­_50_ range: 8.04 - 31.46 μM  Quercetagetin-7-*O*-β-D-glucopyranoside (4): 8.04 ± 0.69 μM  Acarbose: 1.49 ± 0.03 μM |
| 75 | Jing, Pan *et al.*  (2018)^80^ | α-glucosidase (unstated, Chromogenic method) | *Mangifera indica* | 10 flavonoids: flavonol, flavone, aurone, flavanonol, oligomeric  IC­_50_ range: 92.15 - 977.34 μM  Quercetin: 92.15 ± 5.56 μM  Acarbose: 185.25 ± 6.00 μM |
| 76 | Nickavar, B. *et al.* (2013)^81^ | α-amylase (porcine pancreatic, reducing sugar method) | *Salvia virgata* | 1 flavonoid: flavone  Chrysoeriol: 1.27 mM  Acarbose: 0.049 mM |
| 77 | Li, S. *et al.*  (2022)^82^ | α-glucosidase (unstated, Chromogenic method) | *Cerasus humilis* | 6 flavonoids: flavonol, flavanone  IC­_50_ range: 36.17 - 157.74 μg/mL  Myrcetin: 36.17 μg/mL  Acarbose: 189.57 μg/mL |
| 78 | Renda, G. *et al.* (2017)^83^ | α-glucosidase (unstated, Chromogenic method) | *Alchemilla barbatiflora* | 1 flavonoid: oligomeric flavonoid  Procyanidin B3: 241.60 ± 4.21 μM  Acarbose: 57.97 ± 0.23 μM |
| 79 | Jibril, S. *et al.*  (2017)^84^ | α-glucosidase (unstated, Chromogenic method) | *Cassia sieberiana* | 3 flavonoids: flavonol, flavanonol  IC­_50_ range: 5.73 - 92.0 μM  Quercetin: 5.73 ± 3.6 μM  Acarbose: 14.12 ± 1.5 μM |
| 80 | Nickavar, B. *et al.* (2010)^85^ | α-amylase (Porcine pancreatic; reducing sugar method) | *Vaccinium arctostaphylos* | 1 flavonoid: anthocyanidin  Malvidin-3-*O*-β-glucoside: 0.329 mM  Acarbose: 0.033 mM |
| 81 | Khalid, M. F. *et al.* (2022)^86^ | α-glucosidase (unstated; Chromogenic method)  α-amylase (human pancreatic; reducing sugar method) | Commercial | 1 flavonoid: flavanonol  **AG:**  Taxifolin: 45.86 ± 3.78 μM  Acarbose: 4.6 ± 1.26 μM  **AM:**  Taxifolin: 31.26 ± 0.556 μM  Acarbose:  77.88 ± 0.277 μM |
| 82 | Zhou, H. *et al.* (2017)^87^ | α-glucosidase (unstated; Chromogenic method) | *Camellia sinensis* | 11 flavonoids: flavone, isoflavonoid, flavonol, oligomeric, flavan-3-ol  IC­_50_ range: 10.2 - 89.7 μM  Amelliaone A: 10.2 ±0.04 μM  Acarbose: 11.8 ± 0.08 μM |
| 83 | Ha, M. T. *et al.*  (2018)^88^ | α-glucosidase (yeast; Chromogenic method) | *Morus alba* | 8 flavonoids: flavone, chalcone  IC­_50_ range: 8.70 – 100.96 μM  5′-geranyl-5,7,2′,4′-tetrahydroxy-flavone: 8.70 μM  Acarbose: 203.97 ± 4.27 μM |
| 84 | Tajudeen Bale, A. *et al.* (2018)^89^ | α-amylase (Human pancreatic; Reducing sugar method) | Synthesis | 13 flavonoids: chalcone  IC­_50_ range: 1.25 - 2.26 μM  (10): 1.25 ±1.05μM  Acarbose: 1.04 ±0.3μM |
| 85 | Ali, M. *et al.* (2020)^90^ | α-amylase (Porcine pancreatic, Reducing sugar method) | Synthesis | 16 flavonoids: chalcone  IC­_50_ range: 1.47 - 2.81 μM  (*E*)-3-(4-(Dimethylamino)phenyl)-1-(naphthalene-2-yl)prop-2-en-1-one: 1.47 ± 0.481 μM  Acarbose: 1.34 ± 0.326 μM |
| 86 | Fidelis. Q. C. *et al.* (2019)^91^ | α-glucosidase (*Saccharomyces cerevisiae;* Chromogenic method) | *Ouratea hexasperma* | 2 flavonoids: flavone  IC­_50_ range: 20.9 - 69.6 μM  Luteolin: 20.9 ± 0.7μM  Acarbose: 749.0 ± 48.1μM |
| 87 | Tian, X. *et al.* (2020)^92^ | α-glucosidase (*Saccharomyces cerevisiae;* Chromogenic method) | *Paeonia ostii* | 4 flavonoids: flavone, flavonol  IC­_50_ range: 0.306 - 1.137 mM  Kaempferol (11): 0.306 ± 0.002 mM  Acarbose: 0.605 mM |
| 88 | Uddin, S. *et al.* (2022)^93^ | α-glucosidase (Unstated, Chromogenic method)  α-amylase (Human salivary, Chromogenic method) | Australian Honeybee Propolis  Commercial | 8 flavonoids: flavanone, flavonol  **AG**  IC_50_ range: 57.8 - 776.6 μM  Quercetin: 19.2 μM  Acarbose: 396.7 μM  **AM:**  IC_50_ range: 52.5 - 246.0 μM  Quercertin: 17.4 μM  Acarbose: 624.7 μM |
| 89 | Costa Silva, T. D. *et al.* (2019)^94^ | α-amylase (human salivary, Chromogenic method) | *Cassia bakeriana* | 1 flavonoid: flavonol  Kaempferol: 1.5 ± 0.14 μg/mL  Acarbose: 0.05 ± 0.01 μg/mL |
| 90 | Adhikari-Devkota, A. *et al.* (2019)^95^ | α-glucosidase (*Saccharomyces cerevisiae*; Chromogenic method) | *Satsuma mandarin* | 2 flavonoids: flavanone, flavonol  10.6 - 35.2 μM  Quercetin: 10.6 μM  Acarbose: 607.9 μM |
| 91 | Paul, Swastika *et al.* (2020)^96^ | α-glucosidase (*Saccharomyces cerevisiae*; Chromogenic method) | *Brassica rapa* var. *rapa* | 1 flavonoid: chalcone  Lidochalcone A: 62.4 ± 8.0 μM  Acarbose: 142 ± 0.02 μM |
| 92 | Jeong, S. Y. *et al.* (2015)^97^ | α-glucosidase (unstated, Chromogenic method) | *Euonymus alatus* | 2 flavonoids: flavan-3-ol, isoflavone  9.1 - 75.6 μM  Euonymalatus: 9.1 ± 0.5 μM  Acarbose: 124.2 ± 0.4 μM |
| 93 | Linh, N. T. T. *et al.*  (2022)^98^ | α-glucosidase (unstated; Chromogenic method) | *Impatiens chapaensis* | 1 flavonoid: flavanone  (±)-3’,5’,5,7-tetrahydroxyflavanone: 101.00 ± 9.01 μM; 28.91 ± 2.58 μg/mL  Acarbose: 227.14 ± 13.71 μM; 146.64 ± 8.85 μg/mL |
| 94 | Abdullah, Nur Hakimah *et al.* (2016)^99^ | α-glucosidase (*Saccharomyces cerevisiae*, Chromogenic method) | *Uncaria cordata* var. *ferruginea* | 1 flavonoid: flavonol  Quercetin: 556 μg/mL; 1.84 mM  Acarbose: 580 μg/mL; 0.89 mM |
| 95 | Lee, H. E. *et al.*  (2017)^100^ | α-glucosidase (*Saccharomyces cerevisiae*; Chromogenic method) | *Smilax china* | 4 flavonoids: flavonol, flavone  31.7 - 609 μM  Afzelin: 31.7 ± 1.6 μM  Acarbose: 172 ± 17 μM |
| 96 | Kuroda, M. *et al.* (2012)^101^ | α-glucosidase (Rat intestinal, enzymatic method) | *Scutellaria lateriflora* | 3 flavonoids: flavone  14.9 - 811 μM  Baicalein: 14.9 μM  Acarbose: 0.29 μM (maltase); 1.32 μM (sucrase) |
| 97 | Lam, S. H. *et al.*  (2019)^102^ | α-glucosidase  (*Bacillus stearothermophilus*; Chromogenic method) | *Bombax malabarica* | 1 flavonoid: flavan-3-ol  (-)-epicatechin 5-*O*-β-D-xylopyranoside (5): 345.4 μM  Acarbose: 0.04 μM |
| 98 | Huang, Q. *et al.* (2021)^103^ | α-glucosidase  (*Saccharomyces cerevisiae*; Chromogenic method) | *Paeonia delavayi* | 1 flavonoid: flavone  Luteolin: 94.6 μM  Acarbose: 123.0 μM |
| 99 | Xu, L. *et al.*  (2019)^104^ | α-glucosidase (unstated, Chromogenic method) | *Morus nigra* | 6 flavonoids: flavonol, flavanone  9.79 - 30.21 μM  Nigragenon E (2): 9.79 μM  Acarbose: 987.9 μM |
| 100 | Zeng, Y. R. *et al.* (2018)^105^ | α-glucosidase (unstated, Chromogenic method) | *Hypericum monogynum* | 1 flavonoid: flavanonol  Hypemonone E (5): 257.78 μg/ml  Acarbose: 150.47 μg/ml |
| 101 | Qurtam, A. A. *et al.* (2021)^106^ | α-glucosidase (*Saccharomyces cerevisiae;* Chromogenic method)  α-amylase (Porcine pancreatic, Turbidimetric method) | Commercial | 1 flavonoid: flavone  **AG**  Narirutin: 0.00091 mg/ml  Acarbose: 0.00035 mg/ml  **AM:**  Narirutin: 0.0066 mg/ml  Acarbose: 1.012 mg/ml |
| 102 | Sahnoun, M. *et al.* (2017)^107^ | α-glucosidase (Unstated, Chromogenic method)  α-amylase (Human pancreatic, Reducing sugar method) | Commercial | 5 flavonoids: flavone, flavanone  **AG**  IC_50_ range: 0.55 - 125.5 μM  Naringin: 0.55 ± 0.3 μM  Acarbose: 108.26 ± 0.4 μM  **AM:**  IC_50_ range: 1 – 50 μM  Poncirin: 1 ± 0.2 μM  Acarbose: 43 ± 0.3 μM |
| 103 | Li, Y. Q. *et al.*  (2009)^108^ | α-glucosidase (*Saccharomyces cerevisiae*, Chromogenic method) | Commercial | 3 flavonoids: flavonol  17 - 196 μM  Quercetin: 17 μM  Acarbose: 91 μM |
| 104 | Jia, Y. *et al.*  (2019)^109^ | α-glucosidase (*Saccharomyces cerevisiae*, Chromogenic method) | Commercial | 27 flavonoids: flavonol, flavone, flavanonol, dihydrochalcone, flavan-3-ol, , anthocyanidin  11.63 - 16244.26 μM  Myricetin: 11.63 ± 0.36 μM  Acarbose: 0.59 ± 0.14 μM |
| 105 | Wang, L. *et al.* (2022)^110^ | α-glucosidase (*Saccharomyces cerevisiae*; Chromogenic method)    α-amylase (porcine pancreatic; reducing sugar method) | Commercial | 2 flavonoids: flavonol  **AG**  IC_50_ range: 0.12 - 1.9 mg/ml  Quercetin: 0.12 mg/ml  Acarbose: 0.015 mg/ml  **AM: No specific IC_50_ mentioned** |
| 106 | Olennikov, D. N. *et al.* (2014)^111^ | α-amylase (*Aspergillus niger,* Enzymatic method) | *Calendula officinalis* | 6 flavonoids: flavonol  IC_50_ range: 1.79 - 89.85 μg/mL  Quercetin: 1.79 μg/mL  Acarbose: 9.54 μg/mL |
| 107 | Lyu, Q. *et al.*  (2019)^112^ | α-glucosidase (unstated, Chromogenic method) | *Litchi chinensis* | 4 flavonoids: flavonol  Quercetin: 1.68 ± 0.13 mg/ml  Acarbose: 2.44 ± 0.16 mg/ml |
| 108 | Li, Gang *et al.*  (2020)^113^ | α-glucosidase (unstated; Chromogenic method)  α-amylase (unstated; Iodinemetric method) | Synthesis | 2 flavonoid: flavone  **AG:**  Oroxin D: 180.4 ± 25.7 μM  Acarbose: 449.3 ± 38.9 μM  **AM:**  Oroxin C: 210.3 ± 19.1 μM  Acarbose: 579.2 ± 30.7 μM |
| 109 | Wang, X. L. *et al.* (2017)^114^ | α-glucosidase (*Saccharomyces cerevisiae*, pNPGalactose, Chromogenic method) | *Pueraria*  *lobata* | 5 flavonoids: isoflavonoid  IC_50_ range: 23.01 - 524.08 μM  Daidzein: 23.01 μM  Acarbose: 1998.79 μM |
| 110 | Mo, Q. G. *et al.*  (2021)^115^ | α-glucosidase (Rat intestinal, Chromogenic method) | *Ginkgo biloba* | 4 flavonoids: flavonol  IC_50_ range: 1.82 - 3.58 mM  Quercetin 3-*O*-(6-*O*-trans-p-feruloyl)- β-D-glucopyranosyl-(1→ 2)-α-L-rhamnopyranoside: 1.82 ± 0.09 mM  Acarbose: 1.21 ± 0.03 mM |
| 111 | Nazir, N. *et al.* (2020)^116^ | α-glucosidase (*Saccharomyces cerevisiae*, Chromogenic method)  α-amylase (Porcine pancreatic, Reducing sugar method) | *Elaeagnus umbellata* | 1 flavonoid: flavan-3-ol  **AG**  Catechin: 32 μg/mL  Acarbose: 26 μg/mL  **AM:**  Catechin: 38 μg/mL  Acarbose: 30 μg/mL |
| 112 | Oueslati, Mohamed Habib *et al.* (2020)^117^ | α-glucosidase (unstated; Chromogenic method) | *Lotus lanuginosus* | 3 flavonoids: flavonol  87.24 - 412 μM  Isorhamnetin 3‑*O*‑rutinoside: 87.24 ± 12.6 μM  Acarbose: 195.21 ± 21.46 μM |
| 113 | Li, Q. *et al.* (2015)^118^ | α-glucosidase (*Saccharomyces cerevisiae*; chromogenic method) | *Impatiens balsamina* | 7 flavonoids: flavonol, flavanonol  IC_50_ range: 1.65 – 24.9 μg/mL  Myricetin: 1.65 μg/mL  Acarbose: 3.36 μg/mL |
| 114 | Ashraf, J. *et al.* (2020)^119^ | α-glucosidase (unstated; chromogenic method)  α-amylase (porcine pancreatic; reducing sugar method) | Synthesis | 17 flavonoids: flavonol.  **AG:**  IC_50_ range: 70.57 – 322.98 μM  5,7-Dibromo-3-hydroxy-2-[1-[(4-methylphenyl)sulfonyl]-1*H*-indol-3-y]-4*H*-chromen-4-one: 70.57 ± 1.13 μM  Acarbose: 75.26 ± 0.15 μM  **AM**  IC_50_ range: 4.86 – 265.61 μM  5,7-Dibromo-3-hydroxy-2-[1-[(4-methylphenyl)sulfonyl]-1*H*-indol-3-yl]-4*H*-chromen-4-one: 4.86 ± 1.39 μM.  Acarbose: 5.02 ± 1.35 μM. |
| 115 | Yang, J. R. *et al.* (2015)^120^ | α-glucosidase (rat intestinal; enzymatic method) | *Scutellaria baicalensis* | 5 flavonoids: flavone.  IC_50_ range: 38.19 – 269.8 μM  Baicalein: 38.19 ± 1.82 μM  Acarbose: 3.5 μM |
| 116 | Nguyen, D. H. *et al.* (2020)^121^ | α-glucosidase (unstated; chromogenic method) | *Eclipta prostrata* L. | 5 flavonoids: flavone, isoflavonoid.  IC_50_ range: 23.8 – 59.5 μM  Pratensein: 23.8 ± 1.0 μM  Acarbose: 161.9 ± 1.4 μM |
| 117 | He, X. F. *et al.*  (2021)^122^ | α-glucosidase (unstated; chromogenic method) | *Alpinia katsumadai* | 26 flavonoids: chalcone  IC_50_ range: 3.1 – 29.5 μM  Katsumadainol A11: 3.1 ±0.2 μM  Acarbose: 170.9 ±3.2 μM |
| 118 | Bo-wei, Zhang *et al.* (2017)^123^ | α-glucosidase (rat intestinal; enzymatic method)  α-amylase (porcine pancreatic; reducing sugar method) | Commercial | 6 flavonoids: flavone, isoflavonoid, flavonol, flavan-3-ol  **AG:**  IC50 range: 74.1 – 339.4 μM  Baicalein: 74.1±5.6 μM  Acarbose: 0.4±0.1 μM  **AM:**  IC50 range: 146.8 – 446.4 μM  Apigenin: 146.8±7.1 μM  Acarbose: 5.3±3.1 μM |
| 119 | Lv, Q. *et al.*  (2019)^124^ | α-glucosidase (*Saccharomyces cerevisiae*; chromogenic method) | *Malus hupehensis* | 2 flavonoids: dihydrochalcone  IC_50_ range: 39.03 – 152.9 μg/mL  3-Hydroxyphloridzin: 39.03 μg/mL  Acarbose: 458.5 μg/mL |
| 120 | Sohretoglu, Didem *et al.* (2018)^125^ | α-glucosidase (*Saccharomyces cerevisiae*; chromogenic method) | *Geranium purpureum*  *Geranium stepporum* Davis*.* | 5 flavonoids: flavonol.  IC_50_ range: 0.97 – 77.42 μM  Quercetin 3-*O*-(3′′-*O*-galloyl)-β-galactopyranoside: 0.97 ± 0.02 μM.  Acarbose: 50.58 ± 0.25 μM. |
| 121 | Su, Z. R. *et al.*  (2015)^126^ | α-glucosidase (unstated; chromogenic method) | Semi-synthesis, Commercial | 12 flavonoids: flavone  IC_50_ range: 18.31 – 112.7 μM  8-((5-hydroxy-2-(4-hydroxyphenyl)-4-oxo-4*H*-chromen-7-yl)oxy)octanoic acid (3f): 18.31 μM  Acarbose: 222.3 μM |
| 122 | Ray, S. *et al.*  (2014)^127^ | α-glucosidase (yeast; chromogenic method)  α-amylase (porcine pancreatic; reducing sugar method) | *Camellia sinensis* L. | 4 flavonoids: flavan-3-ol  **AG:**  IC_50_ range: 2.77 – 9.12 μg/mL  Epigallocatechin gallate: 2.77 ± 0.36 μg/mL (6.06 μM)  Acarbose: 0.006 ± 00 μg/mL (9.16 nM)  **AM:**  IC_50_ range: 0.26 – 20.9 μg/mL  Theaflavin: 0.26 ± 0.02 μg/mL  Acarbose: 4.87 ± 0.14 μg/mL (7.54 μM) |
| 123 | Giang Thanh Thi, Ho *et al.* (2017)^128^ | α-glucosidase (*Saccharomyces cerevisiae*; chromogenic method)  α-amylase (porcine pancreatic; chromogenic method) | Commercial | 11 flavonoids: flavone, flavanone, flavan-3-ol  **AG:**  IC_50_ range: 2.6 – 25.2 μM  Quercetin: 2.6 ± 0.9 μM  Acarbose: 131.2±9.3 μM  **AM:**  IC_50_ range: 2.1 – 26.2 μM  Quercetin: 2.1 ± 0.5 μM  Acarbose: 113.5±4.6 μM |
| 124 | Zhou, Y. *et al.* (2021)^129^ | α-glucosidase (*Aspergillus niger*; chromogenic method)  α-amylase (porcine pancreatic; reducing sugar method) | Commercial | 1 flavonoid: flavone  **AG:**  IC50 range: 544 ± 9.01 μg/mL  Quercetin: 544 ± 9.01 μg/mL  Acarbose: 47.23 ± 0.39 μg/mL  **AM:**  IC50 range: 270 ± 3.1 μg/mL  Quercetin: 270 ± 3.1 μg/mL  Acarbose: 32.3 ± 0.29 μg/mL |
| 125 | Krishna, M. S. *et al.* (2015)^130^ | α-glucosidase (unstated; chromogenic method)  α-amylase (unstated; reducing sugar method) | *Piper longum* L. | 1 flavonoid: flavone  **AG:**  Apigenin 7,4'-dimethyl ether: 27.342 μg/mL  Acarbose: 175.35 μg/mL  **AM:**  Apigenin 7,4'-dimethyl ether: 98.143 μg/mL  Acarbose: 45.20 μg/mL |
| 126 | Su, J. *et al.* (2019)^131^ | α-amylase (unstated; reducing sugar method) | Commercial | 2 flavonoids: flavone, flavan-3-ol  IC50 range: 0.31 – 1.36 mg/mL  Epigallocatechin gallate: 0.31 mg/mL  Acarbose: 0.45 mg/mL |
| 127 | Nickavar, B. *et al.* (2011)^132^ | α-amylase (porcine pancreatic; reducing sugar method) | *Vaccinium arctostaphylos* | 1 flavonoid: flavone  IC50 range: 0.17 mM  Quercetin: 0.17 (0.16 – 0.17) mM  Acarbose: 0.033 (0.0031 – 0.0036) mM |
| 128 | Zhang, Y. *et al.* (2020)^133^ | α-amylase (porcine pancreatic; reducing sugar method) | *Sophora japonica* L. | 1 flavonoid: flavonol  IC50 range: 88.56±0.60 μg/mL  Kaempferol 3-*O*-α-L-rhamnopyranosyl-(1→6)-β-D-galactopyranosyl-7-*O*-α-L-rhamnopyranoside: 88.56±0.60 μg/mL  Acarbose: 48.757±4.02 μg/mL |
| 129 | Minh Anh Thu, Phan *et al.* (2013)^134^ | α-glucosidase (*Saccharomyces cerevisiae* ; chromogenic method)  α-amylase (porcine pancreatic; reducing sugar method) | commercial | 1 flavonoid: flavonol  **AG:**  IC50 range: 28.9 μM  Baohuoside I: 28.9 μM  Acarbose: 236 μM  **AM:** |
| 130 | Huang, D. *et al.* (2015)^135^ | α-amylase (unstated; reducing sugar method) | *Penthorum chinense* | 3 flavonoids: flavanone, chalcone  IC50 range: 0.03 – 0.14 μM  Pinocembrin-7-O-[3-O-galloyl-4'',6''-hexahydroxydiphenoyl]-β-D-glucose: 0.03 μM  Acarbose: 0.21 μM |
| 131 | Numonov, S. *et al.* (2017)^136^ | α-glucosidase (unstated; chromogenic method) | *Geranium collinum* | 4 flavonoids: flavone, flavan-3-ol  IC50 range: 2.62 – 42.44 μg/mL  Epicatechin: 2.62±0.12 μg/mL  Acarbose: 2.19±0.11 μg/mL |
| 132 | Dubey, K. *et al.* (2021)^137^ | α-glucosidase (unstated; chromogenic method)  α-amylase (unstated; reducing sugar method) | Commercial | 1 flavonoid: flavone  **AG:**  IC50 range: 410.30±0.01 μg/mL  Diosmin: 404.22±0.02 μg/mL  Acarbose: 428.97±0.04 μg/mL  **AM:**  IC50 range: 404.22±0.02 μg/mL  Diosmin: 404.22±0.02 μg/mL  Acarbose: 471.38±0.05 μg/mL |
| 133 | Li, M. *et al.* (2022)^138^ | α-glucosidase (*Saccharomyces cerevisiae*; chromogenic method) | Commercial | 3 flavonoid: flavone, flavonol  IC50 range: 49.31 – 173.16 μM  Luteolin: 49.31 ±8.86 μM  Acarbose: 0.80 ±0.03 μM |
| 134 | Tang, H. *et al.* (2020)^139^ | α-glucosidase (*Saccharomyces cerevisiae*; chromogenic method) | Commercial | 14 flavonoids: flavone, flavonol, isoflavonoid, flavanone, flavan-3-ol  IC50 range: 8.13 – 180.2 μM  Epigallocatechin gallate: 8.13 ± 0.16 μM  Acarbose: 425 ± 9.37 μM |
| 135 | Liumin, Wang *et al.* (2022)^140^ | α-glucosidase (*Saccharomyces cerevisiae*; chromogenic method) | *Humulus lupulus* | 4 flavonoids: chalcone, flavanone, flavan-3-ol  IC50 range: 40 – 50 μM  Isoxanthohumol: 40 ±1.0 μM  Acarbose: 58 ±3.7 μM |
| 136 | Jiang, Ping *et al.* (2021)^141^ | α-amylase (unstated; iodinemetric method) | *Taxus cuspidata* | 2 flavonoids: flavan-3-ol  IC50 range: 0.655 – 0.752 mg/mL  Catechin: 0.655 mg/mL  Acarbose: 0.456 mg/mL |
| 137 | Terfassi, S. *et al.* (2022)^142^ | α-glucosidase (unstated; chromogenic method) | *Helianthemum getulum* | 2 flavonoids: flavan-3-ol  IC50 range: 2.7 – 3.09 μM  dulcisflavan: 2.70±0.03 μM  Acarbose: 275.43±1.59 μM |
| 138 | Wang, X. *et al.* (2017)^143^ | α-glucosidase (rat intestinal; enzymatic method) | *Camellia sinensis* var. *assamica* | 11 flavonoid: flavan-3-ol  IC50 range: 32.5 – 107.1 μM  (–)-epigallocatechin-3-*O*-gallate: 32.5 μM  Acarbose: 0.97 μM |
| 139 | Dhameja, M. *et al.* (2022)^144^ | α-glucosidase (*Saccharomyces cerevisiae*; chromogenic method) | Synthesis | 19 flavonoids: flavone  IC50 range: 24.37 – 168.44 μM  *N*-(2,5-dichlorophenyl)-2-(4-((4-(4-oxo-4*H*-chromen-2-yl)phe-noxy)methyl)-1*H*-1,2,3-triazol-1-yl)acetamide: 24.37 ±0.55 μM  Acarbose: 844.81 ±0.69 μM |
| 140 | Dao, T. B. N. *et al.* (2021)^145^ | α-glucosidase (unstated; chromogenic method) | *Combretum quadrangulare,* Semi-synthesis | 10 flavonoids: flavonol, flavone  IC50 range: 30.5 – 282.0 μM  6-Bromokumatakenin: 30.5±1.9 μM  Acarbose: 332.5 μM |
| 141 | Li, R. *et al.* (2019)^146^ | α-glucosidase (unstated; chromogenic method) | *Hippophae rhamnoides* Commercial | 11 flavonoid: flavonol  IC50 range: 8.3 – 112.11 μM  Hippophin M: 8.3 μM  Acarbose: 1727.07 μM |
| 142 | Lin, H. C. *et al.* (2011)^147^ | α-glucosidase (*Bacillus stearothermophilus*;  chromogenic method) | *Machilus philippinensis* | 2 flavonoid: flavonol  IC50 range: 19.02 – 19.46 μM  kaempferol-3-*O*-α-L-arabinopyranoside: 19.02 ± 0.63 μM  Acarbose: 0.031 μM |
| 143 | Zhang, X. *et al.* (2013)^148^ | α-glucosidase (*Saccharomyces cerevisiae*; chromogenic method) | *Callistephus chinensis* | 8 flavonoids: flavone, flavonol, flavanone  IC50 range: 2.04 – 57.4 μg/mL  quercetin: 2.04 μg/mL  Acarbose: 2.24 μg/mL |
| 144 | Praparatana, R. *et al.* (2022)^149^ | α-glucosidase (*Saccharomyces cerevisiae*; chromogenic method) | *Bauhinia strychnifolia* Craib. Stem. | 2 flavonoids: flavone, flavan-3-ol  IC50 range: 6.26 – 25 μM  quercetin: 6.26 μM  Acarbose: 509.6 μM |
| 145 | Barber, E. *et al.* (2021)^150^ | α-glucosidase (human and rat intestinal; enzymatic method) | Commercial | 3 flavonoids: flavone, flavan-3-ol  IC50 range: 21.7 – 175.2 μM  Quercetagetin: 21.7±5.3 μM  Acarbose: 1.65±0.25 μM |
| 146 | Lo Piparo, E. *et al.* (2008)^151^ | α-amylase (human salivary; reducing sugar method) | Commercial | 7 flavonoids: flavonol, flavone  IC50 range: 9.64 – 48 μM  Scutellarein: 9.64±0.30 μM  Acarbose: 0.996±0.011 μM |
| 147 | Abbasi, B. *et al.* (2014)^152^ | α-glucosidase (unstated; chromogenic method) | *Rhynchosia pseudo-cajan* Cambess. | 6 flavonoids: flavonol, flavone, flavan-3-ol  IC50 range: 14.72 – 375.21 μM  Myricetin: 14.72 ±0.25 μM  Acarbose: 38.25±0.12 μM |
| 148 | Asghari, B. *et al.* (2015)^153^ | α-glucosidase (*Saccharomyces cerevisiae*; chromogenic method)  α-amylase (porcine pancreatic; reducing sugar method) | *Salvia chloroleuca* | 3 flavonoids: flavone  **AG:**  IC50 range: 14.7 – 18.3 μM  Luteolin 7-*O*-glucuronide: 14.7±2.1 μM  Acarbose: 16.1±0.8 μM  **AM:**  IC50 range: 61.5 – 81.7 μM  Luteolin 7-O-glucuronide: 61.5±1.4 μM  Acarbose: 53.4±3.1 μM |
| 149 | Devkota, H. P. *et al.* (2021)^154^ | α-glucosidase (*Saccharomyces cerevisiae*; chromogenic method) | *Lindera sericea var. sericea* | 5 flavonoids: flavone, flavan-3-ol, flavonol, flavanone  IC50 range: 3.0 – 59.3 μM  quercetin: 3.0 ± 0.26 μM  Acarbose: 331.6 ± 6.49 μM |
| 150 | Manaharan, T. et al. (2012)^155^ | α-glucosidase (*Saccharomyces cerevisiae*; chromogenic method)  α-amylase (porcine pancreatic; reducing sugar method) | *Syzygium aqueum* | 5 flavonoids: flavonol, dihydrochalcone  **AG:**  IC50 range: 1.9 – 27 μM  Europetin-3-*O*-rhamnoside: 1.9 ± 0.06 μM  Acarbose: 43 ± 1.6 μM  **AM:**  IC50 range: 1.9 – 31 μM  Myricetin-3-*O*-rhamnoside: 1.9 ± 0.02 μM  Acarbose: 19 ± 1.6 μM |
| 151 | Hong, H. C. *et al.* (2013)^156^ | α-glucosidase (*Saccharomyces cerevisiae*; chromogenic method) | *Morus atropurpurea* | 4 flavonoids: flavonol  IC50 range: 13.19 – 365.4 μM  Rutin: 13.19 ± 1.10 μM  Acarbose: 780.2 ± 1.04 μM |
| 152 | Lin, Y. S. *et al.* (2014)^157^ | α-glucosidase (*Bacillus stearothermophilus*; chromogenic method) | *Machilus konishii* | 3 flavonoids: flavonol  IC50 range: 30.3 – 112.8 μM  Kaempferol 3-*O*-(2-β-D-apiofuranosyl)-α-L-rhamnopyranoside: 30.3±6.1 μM  Acarbose: 0.040±0.001 μM |
| 153 | Akoro, S. M. *et al.* (2020)^158^ | α-amylase (unstated; reducing sugar method) | *Garcinia kola* | 3 flavonoids: flavanone  IC50 range: 9.9 – 15.3 μg/mL  3’’,4’,4’’’,5,5’’,7,7’’-hepta-hydroxy-3,8’’-biflavanone: 9.9 ± 0.2 μg/mL  Acarbose: 38.1 ± 8.3 μg/mL |
| 154 | Kiruthiga, N. *et al.* (2021)^159^ | α-amylase (unstated; reducing sugar method) | Synthesis | 13 flavonoids: flavanone  IC50 range: 54.8 – 125.4 μg/mL  7-hydroxy-2-(4-hydroxy-3-methoxyphenyl)-2,3-dihydro-4*H*-chromen-4-one: 54.8 ± 0.4 μg/mL  Acarbose: 21.2 ± 0.64 μg/mL |
| 155 | Shan-Shan, Zhang *et al.* (2022)^160^ | α-glucosidase (unstated, chromogenic method) | *Pueraria thomsonii* | 9 flavonoids: flavonol, isoflavonoid  IC50 range: 107 – 2206 μM  Puerarin: 107±27 μM  Acarbose: 785±1 μM |
| 156 | Sichaem, J. *et al.* (2020)^161^ | α-glucosidase (*Saccharomyces cerevisiae*; chromogenic method) | *Pterocarpus indicus* | 5 flavonoids: isoflavonoid  IC50 range: 39.8 – 288 μM  calycosin: 39.8 μM  Acarbose: 526 μM |
| 157 | Silva, E. L. *et al.* (2016)^162^ | α-glucosidase (*Saccharomyces cerevisiae*, chromogenic method) | *Eremanthus crotonoides* | 3 flavonoids: flavonol, flavone  IC50 range: 7.19 – 59.64 μM  Quercetin: 7.19±0.06 μM  Acarbose: 859.79±0.09 μM |
| 158 | Jeong, G. H. *et al.* (2017)^163^ | α-glucosidase (unstated, chromogenic method) | Semisynthesis | 3 flavonoids: flavanonol  IC50 range: 11.2 – 56.2 μM  Isoradiorutinol: 11.2±0.7 μM  Acarbose: 310.2±3.6 μM |
| 159 | Sengupta, S. *et al.* (2009)^164^ | α-glucosidase (rat intestinal and *Saccharomyces cerevisiae* and *Aspergillus niger*, enzymatic method) | *Tinospora cordifolia* | 1 flavonoid: flavone  IC50 range: 35μM  Saponarin: 35±1.95 μM  Acarbose: 1.7 μM |
| 160 | Zhang, Y. *et al.* (2022)^165^ | α-glucosidase (*Saccharomyces cerevisiae*, chromogenic method) | *Desmodium caudatum* | 5 flavonoids: flavonol, flavone, flavanone  IC50 range: 4.38 – 45.92 μM  8-Prenylquercetin: 4.38 ±0.22 μM  Acarbose: 330.10 ±10.47 μM |
| 161 | Deutschlander, M. S. *et al.* (2011)^166^ | α-glucosidase (*Saccharomyces cerevisiae*; chromogenic method) | *Euclea undulata* var*. myrtina* | 1 flavonoid: flavan-3-ol  epicatechin: 20.18 μM  Acarbose: 7.35 μM |
| 162 | Milella, L. *et al.* (2016)^167^ | α-glucosidase (*Saccharomyces cerevisiae*, chromogenic method)  α-amylase (porcine pancreatic; reducing sugar method) | *Arcytophyllum thymifolium* | **AG:**  1 flavonoid: flavanone  Rhamnetin: 73.9 ± 5.9 μM  Acarbose: 402.7 ± 15.5 μM  **AM:**  1 flavonoid: flavonol  (2*S*)-7-prenyloxyer-iodictyol: 73.9 ± 5.9 μM  Acarbose: 26.3 ± 1.2 μM |
| 163 | Jin, D. X. *et al.* (2021)^168^ | α-glucosidase (*Saccharomyces cerevisiae*; chromogenic method) | *Hypericum attenuatum* | 3 flavonoids: flavonol  IC50 range: 17.23 – 33.9 μM  guaijaverin: 17.23 ± 0.75 μM  Acarbose: 62.20 ± 1.05 μM |
| 164 | Chen, J. *et al.* (2016)^169^ | α-glucosidase (*Saccharomyces cerevisiae*; chromogenic method) | *Ampelopsis grossedentata (3)*  semi-synthesis (6) | 9 flavonoids: Flavanonol, flavonol  IC50 range: 9.3 – 837.7 μM  4′-*O*-(2-*N*,*N*-hydroxy ethyl-toluene-4-sulfonyl)ethylmyricetin: 9.3±0.4 μM  Acarbose: 720.3±57.5 μM |
| 165 | Proença, C. *et al.* (2017)^170^ | α-glucosidase (*Saccharomyces cerevisiae*; chromogenic method) | Commercial, synthesis | 21 flavonoids: flavone, flavonol  IC50 range: 7.6 – 96 μM  2-(3,4-dihydroxyphenyl)-3,7,8-trihydroxy-4*H*-chromen-4-one: 7.6 ± 0.4 μM  Acarbose: 607 ± 56 μM |
| 166 | Yan, J. *et al.* (2014)^171^ | α-glucosidase (*Saccharomyces cerevisiae*; chromogenic method) | Commercial | 1 flavonoid: flavone  IC50 range: (1.72±0.05)×10^−4^ M  2-(3,4-dihydroxyphenyl)-3,7,8-trihydroxy-4*H*-chromen-4-one: (1.72±0.05)×10^−4^ M  Acarbose: (1.08±0.04)×10^-4^ M |
| 167 | Kim, J. H. *et al.* (2017)^172^ | α-glucosidase (*Saccharomyces cerevisiae*; chromogenic method) | *Sophora flavescens* | 4 flavonoid: flavanone, flavanonol  IC50 range: 11.0 – 50.6 μM  (*S*)-2-(2,4-dihydroxyphenyl)-5,7-dihydroxy-8-((*S*)-5-methyl-2-(prop-1-en-2-yl)hex-4-en-1-yl)-6-(3-methylbut-2-en-1-yl)chroman-4-one: 11.0±0.3 μM  Acarbose: 119.2±3.7 μM |
| 168 | Liu, Y. *et al.* (2020)^173^ | α-glucosidase (*Saccharomyces cerevisiae*; chromogenic method) | *Morella rubra* | 3 flavonoid: flavonol  IC50 range: 33.2 – 65.36 μM  myricetin: 33.20±0.43 μM  Acarbose: 381.27±1.07 μM |
| 169 | Le, T. K. D. *et al.* (2022)^174^ | α-glucosidase (*Saccharomyces cerevisiae*, Chromogenic method) | *Knema globularia* | 12 flavonoids: Flavan-3-ol, Flavanonol, Flavanone, Isoflavonoid, Dihydrochalcone, Chalcone, oligomeric  IC_50_ range: 0.4 - 178.2 uM  15: 0.4 ± 0.1 uM  Acarbose: 93.6 ± 0.5 uM |
| \| 170 \| \| --- \| | Flores-Bocanegra, L. *et al.* (2015)^175^ | α-glucosidase (yeast; Chromogenic method) | *Vauquelinia corymbose* | 5 flavonoids: flavan-3-ol, flavonol  IC_50_ range: 0.03-1.60 mM  Quercetin-3-*O*-(6′′-benzoyl)-β-galactoside: 0.03 ± 0.006 mM  Acarbose: 0.05 mM |
| 171 | Tasnuva, S. T. (2019)^176^ | α-glucosidase (*Saccharomyces cerevisiae*, Chromogenic method) | *Mimosa pudica* | 2 flavonoids: flavonol  IC_50_ range: 75.16-481.17 μg/mL  Quercetin: 75.16±0.92 μg/mL  Acarbose: 351.02±1.46 ug/mL |
| 172 | Hou, Z. W. *et al.*  (2022)^177^ | α-glucosidase (*Saccharomyces cerevisiae*, Chromogenic method) | *Camellia sinensis* | 4 flavonoids: flavan-3-ol  IC_50_ range: 3.34 - 22.50 uM  1: 3.34±0.11 uM  Acarbose: 181.63±1.72 uM |
| 173 | Dendup, T. *et al.* (2014)^178^ | α-glucosidase (*Saccharomyces cerevisiae*, Chromogenic method) | *Mucuna pruriens* | 6 flavonoids: Isoflavonoid  IC_50_ range: 12.19 - 115.01 uM  parvisoflavone B: 12.19 ± 0.14 uM  Acarbose: 7.96 ± 1.68 uM |
| 174 | Suthiphasilp, V. *et al.* (2021)^179^ | α-glucosidase (unstated, Chromogenic method) | *Desmos dumosus* | 7 flavonoids: flavanone, flavone, chalcone  IC_50_ range: 5.3 - 154.4 uM  Matteuorien: 5.3 uM  Acarbose: 83.5 uM |
| 175 | Chen, Y. G. *et al.* (2013)^180^ | α-glucosidase (*Saccharomyces cerevisiae*, Chromogenic method) | *Microcos paniculata* | 3 flavonoids: flavone  IC_50_ range: 244.0 -275.4 uM  Vitexin: 244.0 uM  Acarbose: 1007 uM |
| 176 | Chen, J. G. *et al.* (2020)^181^ | α-glucosidase (*Saccharomyces cerevisiae*, Chromogenic method) | *Cinnamomum camphora* | 1 flavonoid: anthocyanidin  Cyanidin: 5.291 uM  Acarbose: 1644 uM |
| 177 | Didem  Söhretoglu. *et al.* (2017)^182^ | α-glucosidase (*Saccharomyces cerevisiae*, Chromogenic method) | *Potentilla astracanica* | 4 flavonoids: isoflavonoid  IC_50_ range: 1.47-56.05 μg/mL  genistein: 1.47±0.11 μg/mL  Acarbose: 31.92 ±0.17 ug/mL |
| 178 | Renda, G. *et al.* (2018)^183^ | α-glucosidase (*Saccharomyces cerevisiae*, Chromogenic method) | *Geranium asphodeloides* | 5 flavonoids: flavonol  IC_50_ range: 52.54 - 74.51 μM  2: 29.92 ± 1.07 μM  Acarbose: 57.49 ± 0.25 μM |
| 179 | Meesakul, P. *et al.* (2019)^184^ | α-glucosidase (*Saccharomyces cerevisiae*, Chromogenic method) | *Desmos cochinchinensis* | 7 flavonoids: flavone, flavanone  IC_50_ range: 0.2 - 158.2 μM  15: 0.2 μM  Acarbose: 170.7 μM |
| 180 | G. López-Angulo *et al.* (2022)^185^ | α-glucosidase (*Saccharomyces cerevisiae,* Chromogenic method) | *Echeveria subrigida* | 3 flavonoids: flavonol, anthocyanidin  IC_50_ range: 0.009-0.166 mg/mL  Proanthocyanidins: 0.009 mg/mL  Acarbose: 4.426 mg/mL |
| 181 | Ye, R. *et al.*  (2017)^186^ | α-glucosidase (Bacillus stearothermophilus*,* chromogenic method) | *Glycyrrhiza uralensis* | 7 flavonoids: flavonol, flavanone, flavanonol  IC_50_ range: 2.3-84.7 μg/mL  3: 2.3 μg/mL  Acarbose: 0.1 µg/mL |
| 182 | Duong, T. H. *et al.* (2021)^187^ | α-glucosidase (*Saccharomyces cerevisiae*, Chromogenic method) | *Artocarpus integer* | 6 flavonoids: chalcone, flavone, flavonol  IC_50_ range: 7.80 - 182 μM  3: 7.80 ± 0.1 μM  Acarbose: 332 ± 3.9 μM |
| 183 | Gabriel Monzón Daza *et al.* (2021)^188^ | α-glucosidase (rat intestinal, Chromogenic method)  α-amylase (porcine pancreatic, turbidimetric method) | *Passiflora ligularis* | 2 flavonoids: flavonol  **AG**  IC_50_ range: 15.4- 1830 μM  16: 15.4 ± 1.1 μM  Acarbose: 156.6 ± 1.1 μM  **AM**:  IC_50_ range: 31.0-33.4 μM  16: 31.0 ± 1.1 μM  Acarbose: 234.1 ± 15.9 μM |
| 184 | Assefa, S. T. *et al.* (2021)^189^ | α-glucosidase (yeast, Chromogenic method) | *Capsicum species* | 2 flavonoids: flavone  IC_50_ range: 7.6 -81 μM  Luteolin: 7.6 μM  Acarbose: 197 μM |
| 185 | Fang, H. L. *et al.* (2022)^190^ | α-glucosidase (*Saccharomyces cerevisiae*, Chromogenic method) | *Lithocarpus polystachyus* | 9 flavonoids: Flavonol, Dihydrochalcone, Flavanone, flavanonol  IC_50_ range: 6.14 - 22.01 mg/mL  Astilbin: 6.14 ± 1.21 mg/mL  Acarbose: 25.30 ± 1.07 mg/mL |
| 186 | Tao, Y. *et al.*  (2013)^191^ | α-glucosidase (yeast, Chromogenic method) | Tang-Zhi-Qing | 2 flavonoids: flavonol  IC_50_ range: 133.16-462.1 μM  Quercetin-3-O-ß-D-glucuronide: 133.16 μM  Acarbose: 465.40 μM |
| 187 | Vonia, S. *et al.* (2022)^192^ | α-glucosidase (*Saccharomyces cerevisiae*, Chromogenic method) | *Gymnanthemum amygdalinum* | 2 flavonoids: flavonol, flavone  IC_50_ range: 6.53- 38.95 µg/mL  Luteolin: 6.53 ± 0.16 µg/mL  Acarbose: 73.36 ± 3.05 µg/mL |
| 188 | Yoon, K. D. *et al.* (2020)^193^ | α-glucosidase (rat intestinal sucrase, Enzymatic method) | *Oryzae sativa* | 3 flavonoids: flavanonol, anthocyanidin  IC_50_ range: 540-1740 µM  C-3-G: (0.97 ± 0.09) mM  Acarbose: <50 µM |
| 189 | Su, H. *et al.* (2020)^194^ | α-glucosidase (*Saccharomyces cerevisiae*, Chromogenic method)  α-amylase (porcine pancreatic, reducing sugar method) | Rhizoma *Smilacis glabrae* | 1 flavonoid: flavononol  **AG**:  Taxifolin: 38 µg/mL  Acarbose: 917 µg/mL  **AM**:  Taxifolin: 1555 µg/mL  Acarbose: 135 µg/mL |
| 190 | Wang, W. *et al.* (2016)^195^ | α-glucosidase (*Saccharomyces cerevisiae*, Chromogenic method)  α-amylase (porcine pancreatic, reducing sugar method) | *Tagetes erecta* | 3 flavonoids: flavonol  **AG:**  IC_50_ range: 99.13-180.11 µM  Rutin: 99.13 ± 1.99µM  Acarbose: 810.85 ± 5.96 µM  **AM**  IC_50_ range: 0.043-0.138 µM  Rutin: 43.29 ± 1.73 µM  Acarbose: 5.80 ± 0.34 µM |
| 191 | Akkarachiyasit, S.  *et al.* (2011)^196^ | α-amylase (porcine pancreatic, Reducing sugar method) | synthesis | 1 flavonoid: anthocyanidin  Cyanidin-3-rutinoside: 24.4 ± 0.1 μM  Acarbose: 18.1 ± 0.1 μM |
| 192 | Hu, Y. C. *et al.* (2012)^197^ | α-amylase (porcine pancreatic, Chromogenic method) | *Cleistocalyx operculatus* | 1 flavonoid: chalcone  DMC: 43 uM  Acarbose: 1.64 uM |
| 193 | Lian-Xin. *et al.* (2019)^198^ | α-glucosidase (unstated, Chromogenic method) | commercial | 3 flavonoids: flavonol  IC50 range: 0.006-0.102 mg/mL  Quercetin: 0.006 mg/mL  Acarbose: 0.741 mg/mL |
| 194 | Liao, G. *et al.* (2020)^199^ | α-glucosidase (unstated, Chromogenic method) | synthesis | 1 flavonoid: flavone  2b: 60.1 ± 0.6 uM  Acarbose: 766.2 ± 37.8 uM |
| 195 | Yang, J. *et al.* (2021)^200^ | α-glucosidase (*Saccharomyces cerevisiae*, Chromogenic method)  α-amylase (porcine pancreatic, Chromogenic method) | commercial | 1 flavonoid: flavanonol  **AG:**  Silibinin: 11.54 ± 0.81 µM  Acarbose: 194.20 ± 0.30 µM  **AM:**  Silibinin: 59.11 ± 1.72 µM  Acarbose: 1.99 ± 0.02 µM |
| 196 | Kwon, R. H. *et al.* (2022)^201^ | α-glucosidase (*Saccharomyces cerevisiae*, Chromogenic method) | *Morus alba* | 4 flavonoids: flavone, flavanonol, oligomeric  IC_50_ range: 1.44 - 47.35 µM  7: 1.44 ± 0.11 μM  Acarbose: 350.9 ± 17.94 μM |
| 197 | Fang, Hua. *et al.* (2018)^202^ | α-glucosidase (*Saccharomyces cerevisiae*, Chromogenic method)  α-amylase (porcine pancreatic, Reducing sugar method) | Lu'an GuaPian | 15 flavonoids: flavonol  **AG:**  IC_50_ range: 14.08 - 106.65 µM  5: 14.08 ± 2.21 μM  Acarbose: 179.60 ± 25.76 μM  **AM:**  IC_50_ range: 0.09 - 12.66 µM  13: 0.09 ± 0.02 μM  Acarbose 2.78 ± 0.46 μM |
| 198 | Sheliya, M. A. *et al.* (2015)^203^ | α-glucosidase (unstated, Chromogenic method) | *Euphorbia hirta* | 4 flavonoids: flavonol  IC_50_ range: 22-182 µM  3: 22 µM  Acarbose: 92 µM |
| 199 | Pyner, A. *et al.* (2017)^204^ | α-glucosidase (rat and human, Enzymatic method) | commercial | 1 flavonoid: flavan-3-ol  EGCG: 14± 2.0 µM  Acarbose: 0.42 ± 0.02 µM |
| 200 | Tuan, N. N. *et al.* (2022)^205^ | α-glucosidase (*Saccharomyces cerevisiae*, Chromogenic method) | *Millettia speciosa* | 1 flavonoid: flavonol  Rutin: 2.2 ± 0.09 µg/mL  Acarbose: 169.8 ± 7.05 µg/mL |
| 201 | Sun, J. *et al.* (2018)^206^ | α-amylase (human salivary, reducing sugar method) | *Pomegranate rind* | 4 flavonoids: flavone, flavonol  IC_50_ range: 59.67- 265.65 µM  Luteolin: 59.67 µM  Acarbose: 11.26 µM |
| 202 | Luyen, N. T. *et al.* (2013)^207^ | α-glucosidase (*Saccharomyces cerevisiae*, Chromogenic method)  α-amylase (*Aspergillus oryzae*, Iodinemetric method) | *Chrysanthemum morifolium* | 3 flavonoids: flavanone, flavone  **AG:**  IC_50_ range: 362.5 - 451.8 µM  4: 362.5 ± 35.1 μM  Acarbose: 1907 ± 156 μM  **AM:**  IC_50_ range: 112.5 - 337.1 µM  4: 112.5 ± 15.7.1 μM  Acarbose: 732.4 ± 41.6 μM |
| 203 | Umamaheswari, S.  *et al.* (2019)^208^ | α-glucosidase (unstated, enzymatic method) | synthesis | 2 flavonoids: flavone  IC_50_ range: 0.47 – 46.37 µM/mL  2’,3’-dihydroxyflavone: 0.47 µM/mL  Acarbose: 23.84 µM/mL |
| 204 | Akkarachiyasit, S. *et al.* (2010)^209^ | α-glucosidase (Rat intestinal, Enzymatic method)  α-amylase (porcine pancreatic, Reducing sugar method) | Commercial | 3 Flavonoids: Anthocyanidin  **AG**:  IC_50_ range: 0.5- 1.42 mM  Cyanidin-3-galactoside: 0.50 ± 0.05 mM  Acarbose: 0.09 ± 0.04 mM  **AM**:  IC50 range: 0.3 - 0.38 mM  Cyanidin-3-glucoside: 0.30 ± 0.01 mM  Acarbose: 0.12 ± 0.04 mM |
| 205 | Han, L. *et al.* (2022)^210^ | α-glucosidase (*Saccharomyces cerevisiae*, Chromogenic method) | Commercial | 2 flavonoids: isoflavonoid  IC_50_ range: 39.45 - 174.04 µM  CA: 39.45 µM  Acarbose: 471.73 µM |
| 206 | Kim, J. H. *et al.* (2018)^211^ | α-glucosidase (*Saccharomyces cerevisiae*, Chromogenic method) | *Orostachys japonicus* | 2 flavonoids: flavan-3-ol  IC_50_ range: 0.6-3.1 µM  2: 0.6 ± 0.2 μM  Acarbose: 121 ± 2.7 μM |
| 207 | Xu, L. *et al.* (2019)^212^ | α-glucosidase (*Saccharomyces cerevisiae*, Chromogenic method) | commercial | 1 flavonoid: flavan-3-ol  EGCG: 19.5 ± 0.3 µM  Acarbose: 278.7 ± 1.1 µM |
| 208 | Sadeghi, M. *et al.* (2022)^213^ | α-glucosidase (*Saccharomyces cerevisiae*, Chromogenic method)  α-amylase (porcine pancreatic, reducing sugar method) | commercial | 2 flavonoids: flavonol, flavone  AG:  Nicotiflorin: 148 µg/mL  Acarbose: 1478 µg/mL  AM:  Swertisin: 1894 µg/mL  Acarbose: 137 µg/mL |
| 209 | Li, K. *et al.* (2018)^214^ | α-glucosidase (*Saccharomyces cerevisiae*, Chromogenic method)  α-amylase (porcine pancreatic, Chromogenic method) | *Scutellaria baicalensis* | 8 flavonoids: flavanone, flavone  **AG**  IC_50_ range: 231-2149 µg/mL  1: 231.13±5.35 μg/mL  Acarbose: 996.02±21.34 µg/mL  **AM**:  IC_50_ range: 287-2941 µg/mL  1: 287.53±5.39 µg/mL  Acarbose: 2941.25±62.12 µg/mL |
| 210 | Tundis, R. *et al.* (2007)^215^ | α-amylase (unstated, reducing sugar method) | *Salicornia herbacea* | 2 flavonoids: flavonol  IC_50_ range: 129-619 µM  Isorhamnetin-3-*O*-rut: 0.129 ± 0.7 mM  Acarbose: 0.025 ± 0.002 mM |
| 211 | Peng, X. *et al.* (2016)^216^ | α-glucosidase (*Saccharomyces cerevisiae*, Chromogenic method) | commercial | 1 flavonoid: flavonol  Kaempferol: 11.6 ± 0.4 μM  Acarbose: 209 ± 3 μM |
| 212 | Li, Zeng. *et al.*  (2016)^217^ | α-glucosidase (*Saccharomyces cerevisiae*, Chromogenic method) | commercial | 3 flavonoids: flavone, flavonol  IC_50_ range: 2.25 - 10.5 μM  C: 2.25 ± 0.05) × 10^-3 µg/mL  Acarbose: 0.304±0.004 µg/mL |
| 213 | Wu, X. *et al.*  (2019)^218^ | α-glucosidase (*Saccharomyces cerevisiae*, Chromogenic method)  α-amylase (human salivary, reducing sugar method) | commercial | 2 flavonoids: flavan-3-ol  **AG:**  IC_50_ range: 1.05-4.03 μg/ml  EGCG: 1.05 ± 0.02 μg/ml  Acarbose: 79.41 ± 0.19 μg/ml  **AM:**  IC_50_ range: 45.30- 137.15 μg/ml  ECG: 45.30 ± 0.22 μg/ml  Acarbose: 27.76 ± 0.19 μg/ml |
| 214 | Zeng, L. *et al.* (2016)^219^ | α-glucosidase (*Saccharomyces cerevisiae*, Chromogenic method) | commercial | 1 flavonoid: flavonol  Morin: 4.48 ± 0.04 µM  Acarbose: 402 ± 5 µM |
| 215 | Mengting, Ni. *et al.* (2020)^220^ | α-glucosidase (*Saccharomyces cerevisiae*, Chromogenic method) | commerical | 1 flavonoid: flavone  Vitexin: 52.80 ± 1.65 μM  Acarbose: 375 ± 12.5 μM |
| 216 | Dong, H. Q. *et al.* (2012)^221^ | α-glucosidase (unstated, Chromogenic method) | \| *Lithocarpus polystachyus* \| \| --- \| | 2 flavonoids: dihydrochalcone, flavonol  IC_50_ range: 0.37 - 0.45 mg/mL  Trilobatin: 0.37 mg/mL  Acarbose: 0.28 mg/mL |
| 217 | Uddin, Md Josim. *et al.* (2022)^222^ | α-glucosidase (*Saccharomyces cerevisiae*, Chromogenic method)  α-amylase (porcine pancreatic, Iodinemetric method) | *Ceriscoides campanulata* | 8 flavonoids: flavonol, oligomeric  **AG:**  IC_50_ range: 4.6-142 μM  16: 4.6 ± 0.1 μM  Acarbose: 665 ± 42 μM  **AM:**  IC_50_ range: 3.5-289 μM  16: 3.5 μM  Acarbose: 5.9 ± 0.1 μM |
| 218 | Zhao, L. *et al.* (2020)^223^ | α-glucosidase (*Saccharomyces cerevisiae*, Chromogenic method) | commercial | 3 flavonoids: oligomeric, flavan-3-ol  IC_50_ range: 25.28- 626.15 μg/mL  7: 25.28 ± 0.67 μg/mL  Acarbose: 376.28 ± 10.49 μg/mL |
| 219 | Imran, M. *et al.* (2021)^224^ | α-glucosidase (unstated, Chromogenic method) | \| *Abutilon pakistanicum* \| \| --- \| | 2 flavonoids: flavonol  IC_50_ range: 8.27-52.43 μg/mL  Abutilin C: 8.27 ± 0.03 μg/mL  Acarbose: 5.92 ± 0.20 µg/mL |
| 220 | Ha, L. M. *et al.* (2018)^225^ | α-glucosidase (yeast, Chromogenic method)  α-amylase (porcine pancreatic, Chromogenic method) | *Desmodium heterophyllum* | 4 flavonoids: isoflavonoid  **AG**  IC_50_ range: 257.3 - 879.4 μM  5: 257.3 ± 12.8 µM  Acarbose: 671.4 ± 32.7 µM  **AM**  IC_50_ range: 110.4 - 162.8 μM  5: 110.4 ± 7.2 µM  Acarbose: 89.3 ± 6.2 µM |
| 221 | Yi, J. *et al.* (2022)^226^ | α-glucosidase (*Saccharomyces cerevisiae*, Chromogenic method)  α-amylase (porcine pancreatic, Iodinemetric method) | *Dracaena angustifolia* | 2 flavonoids: flavan  **AG**: 5: (0.24±0.037).10^3^ µM  Acarbose: (4.07±0.516).10^3^ μM  **AM**: 6: (6.03±0.27).10^3^ µM  Acarbose: 10.25±0.01 μM |
| 222 | Ha, T. J. *et al.* (2018)^227^ | α-glucosidase (*Saccharomyces cerevisiae,* Chromogenic method) | *Vigna nakashimae* | 4 flavonoids: flavan, flavan-3-ol, oligomeric  IC_50_ range: 36.8 -150.3 µM  Gambiriin D: 36.8 ± 2.3 µM  Acarbose: 620.2 ± 7.2 µM |
| 223 | Costa *et al.* (2015)^228^ | α-glucosidase (*Saccharomyces cerevisiae*, Chromogenic method) | *Passiflora bogotensis* | 6 flavonoids: flavone  IC_50_ range: 19-35 µM  2: 19 ± 0.01 µM  Acarbose: 15 ± 0.01 µM |
| 224 | Park, Mi Jin *et al.* (2020)^229^ | α-glucosidase (unstated, Chromogenic method) | \| *Agrimonia pilosa* \| \| --- \| | 4 flavonoids: flavonol, flavone  IC_50_ range: 28.7- 117.6 μM  Vitexin: 28.7 ± 1.2 μM  Acarbose: 45.2 ± 1.2 µM |
| 225 | Marmouzi, I. *et al.* (2021)^230^ | α-amylase (unstated, reducing sugar method) | *Hemimycale columella* | 3 flavonoids: flavonol  IC_50_ range: 1137.88 – 2111.18 µM  Kaempferol 3-*O*-β-glucopyranoside: 1137.88 ± 9.6 µM  Acarbose: 357.57 ± 2.45 µM |
| 226 | Jiang, M. Y. *et al.* (2020)^231^ | α-glucosidase (unstated*,* chromogenic method) | *Moringa oleifera* | 8 flavonoids: flavonol, flavanone  IC_50_ range: 14.9 – 347.4 µM  Compound 11: 14.9 ± 3.2 µM  Acarbose: 253.6 ± 9.6 µM |
| 227 | Keskes, H. *et al.* (2017)^232^ | α-amylase (porcine pancreatic, chromogenic method) | *Juniperus phoenicea* | 1 oligomeric flavonoid  Amentoflavone: 20.4 ± 1.2 µg/ml  Acarbose: 14.9 ± 1.0 µg/ml |
| 228 | Astiti, M. A. *et al.* (2021)^233^ | α-glucosidase (*Saccharomyces cerevisiae,* chromogenic method) | *Coccinia grandis* | 6 flavonoids: flavonol  IC_50_ range: 195.4-455.6 µM  Kaempferol 3-*O*-robinobioside: 195.4 ± 18.13 µM  Acarbose: 2023.3 ± 17.34 µM |
| 229 | Wang, Y. L. *et al.* (2019)^234^ | α-glucosidase (unstated*,* chromogenic method) | *Viola philippica* | 1 flavonol  5-hydroxy-4′-methoxyflavone-7-*O*-rutinoside: 24.63 µg/mL  Acarbose: 0.012 µg/mL |
| 230 | Wu, Q. *et al.*  (2019)^235^ | α-glucosidase (yeast*,* chromogenic method)  α-amylase (porcine pancreatic, reducing sugar method) | Commercial | 1 flavan-3-ol  **AG:**  Catechin: 0.307 mg/mL  Acarbose: 0.211 mg/mL  **AM:**  Catechin: 0.533 mg/mL  Acarbose: 0.461 mg/mL |
| 231 | Matsui, T. *et al.* (2002)^236^ | α-glucosidase (rat intestinal sucrase*,* enzymatic method) | Commercial | 2 flavonoids: flavone, flavonol  IC_50_ range: 2.3 – 17.3 mM  Luteolin: 2.3 mM  Acarbose: 430 nM |
| 232 | Djeujo, F. M. *et al.* (2022)^237^ | α-glucosidase (*Saccharomyces cerevisiae,* chromogenic method) | Commercial | 1 flavonoids: flavone  Luteolin: 14 ± 10.5 µM  Acarbose: 805.4 ± 10.2 µM |
| 233 | Visvanathan, R. *et al.* (2021)^238^ | α-amylase (human salivary, reducing sugar method) | Commercial | 2 flavonoids: flavan-3-ol, flavonol  IC_50_ range: 20.41 – 30.15 µM  EGCG: 20.41 ± 0.25 µM  Acarbose: 2.08 ± 0.01 µM |
| 234 | Kim, J. H. *et al.* (2019)^239^ | α-glucosidase (*Saccharomyces cerevisiae,* chromogenic method) | Commercial | 2 anthocyanidins  IC_50_ range: 14.4 – 29.7 µM  Delphinidin: 14.4 ± 0.1 µM  Acarbose: 210.8 ± 4.6 µM |
| 235 | Wang, X. *et al.* (2022)^240^ | α-amylase (porcine pancreatic, chromogenic method) | Commercial | 4 flavonoids: flavone  IC_50_ range: 10.83 – 30.08 µM  Nepetin: 10.83 ± 0.49 µM  Acarbose: 1.16 ± 0.07 µM |
| 236 | Hyun, T. K. *et al.* (2014)^241^ | α-glucosidase (Bacillus stearothermophilus*,* chromogenic method) | Commercial | 3 flavonoids: flavonol  IC_50_ range: 1.0 – 84.1 µg/mL  Quercetin: 1.0 ± 0.1 µg/mL  Acarbose: 140.5 ± 0.5 µg/mL |
| 237 | Şöhretoğlu, D. *et al.* (2022)^242^ | α-glucosidase (*Saccharomyces cerevisiae,* chromogenic method) | *Ziziphus jujuba* | 1 oligomeric flavonoid  Procyanidin B4: 170.18 ± 5.60 µg/mL  Acarbose: 46.10 ± 2.30 µg/mL |
| 238 | Priscilla, D. H. *et al.* (2014)^243^ | α-glucosidase (rat and yeast*,* chromogenic method) | Commerical | 1 flavanone  Naringenin: 384 µM  Acarbose: 2.45 µM |
| 239 | Sun, H. *et al.* (2017)^244^ | α-glucosidase (*Saccharomyces cerevisiae,* chromogenic method)  α-amylase (porcine pancreatic, reducing sugar method) | *Humulus lupulus* | 6 flavonoids: chalcone, flavanone  **AG:**  IC_50_ range: 1.08 – 45.92 µM  3'-geranylchalconaringenin: 1.08 µM  Acarbose: 51.3 µM  **AM:**  IC_50_ range: 15.38-85.92 µM  8-geranylnaringenin: 15.38 µM  Acarbose: 2.21 µM |
| 240 | Tran, C. L. *et al.* (2021)^245^ | α-glucosidase (*Saccharomyces cerevisiae,* chromogenic method) | *Nervilia concolor* | 2 flavonoids: flavone; flavonol  IC_50_ range: 155 – 210 µg/mL  5,7-dimethoxy-4'-hydroxyflavone: 155 µg/mL  Acarbose: 214 µg/mL |
| 241 | Shi, Z. F. *et al.* (2016)^246^ | α-glucosidase (*Saccharomyces cerevisiae,* chromogenic method) | *Ficus hispida* | 5 isoflavonoids  IC_50_ range: 22.1 – 108.1 µM  3'-(3-methylbut-2-en-1-yl)biochanin A: 22.1 ± 7.6 µM  Acarbose: 347.1 ± 68.6 µM |
| 242 | Qin, N. B. *et al.* (2017)^247^ | α-glucosidase (*Saccharomyces cerevisiae,* chromogenic method) | *Silybum marianum* | 4 flavonoids: flavan-3-ol, flavanone, flavonol  IC_50_ range: 1.44 – 41.43 µM  naringenin 7-*O*-β-D-glucopyranoside: 1.44 ± 0.01 µM  Acarbose: 2.68 ± 0.07 µM |
| 243 | Li, N. *et al.* (2020)^248^ | α-glucosidase (unstated*,* chromogenic method) | *Cammelia sinensis* | 11 flavonoids: flavan-3-ol, oligomeric, flavonol  IC_50_ range: 0.954 – 6.024 µM  (-)-epicatechin 3-*O*-gallate: 0.954 ± 0.013 µM  Acarbose: 228.95 ± 0.38 µM |
| 244 | Dat, N. T. *et al.* (2016)^249^ | α-amylase (porcine pancreatic, Iodinemetric method) | *Sesamum indicum* | 2 flavonoids: flavonol, flavan-3-ol  Epigallocatechin: 303.9 µM  Acarbose: 124.0 µM |
| 245 | Do, L. M. T. *et al.* (2022)^250^ | a-glucosidase (*Saccharomyces cerevisiae*, chromogenic method) | *Melodorum fruticosum* | 5 flavonoids: flavone, flavanone  IC_50_ range: 2.59 - 192 µM  melodorone A: 2.59 ± 0.15 µM  Acarbose: 179 ± 6.02 µM |
| 246 | Janibekov, A. A. *et al.* (2018)^251^ | a-glucosidase (*Saccharomyces cerevisiae*, chromogenic method) | *Astragalus turkestanus*  *Astragalus xапthomeloides* | 2 flavonoids: flavonol  IC_50_ range: 50.31-79.6 µg/mL  Compound 3: 50.31 ± 4.98 µg/mL  Acarbose: 30.57 ± 3.27 µg/mL |
| 247 | Ranga Rao, R. *et al.* (2009)^252^ | a-glucosidase (rat intestinal, chromogenic method) | *Derris indica* | 2 flavonoids: flavone, chalcone  IC_50_ range: 103.5 - 335 µM  Pongamol: 103.5 µM  Acarbose: 24 µM |
| 248 | Zhang, L. *et al.* (2015)^253^ | α-glucosidase (yeast***,*** chromogenic method) | *Acer pseudoplatanus* | 13 flavonoids: flavan-3-ol; flavonol  IC_50_ range: 26.48 – 622.32 µM  Compound 7: 26.48 ± 0.08 µM  Acarbose: 218.38 ± 21.78 µM |
| 249 | Wang, M. *et al.* (2015)^254^ | a-glucosidase (*Saccharomyces cerevisiae*, chromogenic method) | *Morus laevigata* | 1 flavonoids: flavonol  laevigasin A: 17.95 ±1.97 µM  Acarbose: 347.12 ± 68.61 |
| 250 | He, X. F. *et al.* (2020)^255^ | α-glucosidase (*Saccharomyces cerevisiae* ***,*** chromogenic method) | *Amomum tsao-ko* | 19 flavonoids: flavan-3-ol  IC_50_ range: 5.6 – 108.5 µM  Tsaokoflavanol F: 5.6 ± 2.1 µM  Acarbose: 180 ± 1.7 µM |
| 251 | Ren, D. *et al.* (2018)^256^ | α-glucosidase (unstated***,*** chromogenic method) | *Cephalotaxus oliveri* | 9 flavonoids: oligomeric flavonoids, flavone, flavanone  IC_50_ range: 8.19 – 210.95 µM  Umcephabiflovin A: 8.19 ± 1.78 µM  Acarbose: 286.37 ± 10.36 µM |
| 252 | Nile, A. *et al.* (2021)^257^ | α-glucosidase (unstated***,*** chromogenic method) | *Allium cepa* | 5 flavonoids: flavonol  IC_50_ range: 13.6 - 35.6 µM  Spiraeoside: 13.6 ± 1.1 µM  Acarbose: 10.1 ± 0.6 µM |
| 253 | Nguyen, V. B. *et al.* (2018)^258^ | a-glucosidase (*Saccharomyces cerevisiae*, chromogenic method) | *Euonymus laxiflorus* | 2 flavonoids: flavan-3-ol  IC_50_ range: 0.113 - 11.9 µg/ml  (+)-Catechin: 0.113 ± 0.008 µg/ml  Acarbose: 1.345 ± 89µg/ml |
| 254 | Kong, F. *et al.* (2020)^259^ | a-glucosidase (unstated, chromogenic method) | Commercial | 1 flavonoid: flavanone  Naringin: 15.782 mg/ml  Acarbose: 1.034 mg/ml |
| 255 | Zhang, K. *et al.* (2020)^260^ | α-glucosidase (*Saccharomyces cerevisiae,* chromogenic method) | Commercial | 2 flavonoids: flavanone  IC_50_ range: 0.214-12.85 mg/ml  Naringenin: 0.214 ± 0.12 mg/ml  Acarbose: 1.308 ± 0.12 mg/ml. |
| 256 | Damsud, T. *et al.* (2014)^261^ | α-glucosidase (yeast and rat***,*** chromogenic method) | *Orthosiphon aristatus* | 2 flavonoids: flavone, flavonol  IC_50_ range: 0.75 – 6.34 mM  3,7,4'-tri-*O*-methylkaempferol: 0.75 mM  Acarbose: 480 µM |
| 257 | Xu, Y. *et al.*  (2018)^262^ | α-glucosidase (*Saccharomyces cerevisiae,* chromogenic method) | Berry fruits | 16 flavonoids: anthocyanidin  IC_50_ range: 1.69 – 1025.32 µM  Pelargonidin 3-rutinoside: 1.69 µM  Acarbose: 356.26 µM |
| 258 | Xu, L. *et al.*  (2020)^263^ | α-glucosidase (unstated***,*** chromogenic method) | *Morus nigra* | 9 flavonoids: flavonol  IC_50_ range: 1.43 – 20.84 µM  Sanggenol H: 1.43 µM  Acarbose: 987.9 µM |
| 259 | Tan, C. *et al.*  (2015)^264^ | α-glucosidase (yeast***,*** chromogenic method) | *Sarcopyramis nepalensis* | 7 flavonoids: flavonol  IC_50_ range: 0.69 – 2.09 mg/mL  Isoquercetin: 0.69 ± 0.09 mg/mL  Acarbose: 1.23 ± 0.12 mg/mL. |
| 260 | Ho, G. T. *et al.* (2017)^265^ | α-glucosidase (*Saccharomyces cerevisiae,* chromogenic method)  α-amylase (Porcine pancreatic, chromogenic method) | *Sambucus nigra* | 6 flavonoids: anthocyanidin  **AG:**  IC_50_ range: 2.8 – 11.9 µM  Cyandin-3-sambubioside: 2.8 ± 0.9 µM  Acarbose: 131.2 ± 19 µM  **AM:**  IC_50_ range: 2.3 – 16.2 µM  Cyanidin-3-sambubioside: 2.3 ± 0.5 µM  Acarbose: 113.5 ± 16 µM |
| 261 | Toh, Z. S. *et al.* (2015)^266^ | α-amylase (porcine pancreatic, Reducing sugar method | *Daemonorops draco*  Semi-synthesis | 3 flavonoids: Oligomeric  IC50 range: 12-27 µM  Galloylated dracoflavan B: 12 µM  Acarbose: 28 µM |
| 262 | Yang, K. *et al.* (2016)^267^ | α-glucosidase (*Saccharomyces cerevisiae,* chromogenic method) | *Fragaria ananassa* | 4 flavonoids: flavan-3-ol; flavonol  IC_50_ range: 65.22 - 537.43 µM  kaempferol 3-(6-methylglucuronide): 65.22 ± 2.70 µM  Acarbose: 619.94 ± 118.34 µM |
| 263 | Nina, N. *et al.* (2020)^268^ | α-glucosidase (*Saccharomyces cerevisiae,* chromogenic method) | *Ombrophytum subterraneum* | 3 flavonoids: flavanonol, flavanone  IC_50_ range: 4.85 – 9.58 µg/ml  3′,5,5′,7-tetrahydroxyflavanone 7-*O*-β-D-1→ 6diglucoside: 4.85 ± 0.34 µg/ml  Acarbose: 137.73 ± 1.31 µg/ml |
| 264 | Amin, A. *et al.* (2016)^269^ | α-glucosidase (*Saccharomyces cerevisiae,* chromogenic method) | *Nymphoides indica* | 2 flavonoids: flavone  IC_50_ range: 27-45 mM  3,7-di-*O*-methylquercetin: 27 mM  Acarbose: 260 µM |
| 265 | Sadasivam, M. *et al.* (2018)^270^ | α-amylase **(**unstated, reducing sugar method) | *Citrus hystrix* | 5 flavonoids: 4 flavones, 1 flavonol IC_50_ range: 19.017–35.511 μg/mL  5,6,4′-trihydroxypyranoflavone: 19.017±0.112 μg/mL  Acarbose: 16.194±0.187 μg/mL |
| 266 | Kim, T. H. *et al.* (2017)^271^ | α-glucosidase (*Saccharomyces cerevisiae,* chromogenic method) | Commercial | 1 flavonol  Quercetin: 158.7 ± 2.3 µM  Acarbose: 280.1 ± 3.2 µM |
| 267 | Jeong, G. H. *et al.* (2021)^272^ | α-glucosidase (*Saccharomyces cerevisiae,* chromogenic method)  α-amylase (Porcine pancreatic, chromogenic method) | Commercial | 1 flavan-3-ol  **AG:**  EGCG: 97.2 ± 1.2 µM  Acarbose: 169.0 ± 1.8 µM  **AM:**  EGCG: 75.5 ± 1.3 µM  Acarbose: 70.7 ± 1.4 µM |
| 268 | Li, F. *et al.* (2013)^273^ | α-glucosidase (unstated***,*** chromogenic method) | *Polygonum cuspidatum* | 1 flavonoid: flavanol  polyflavanostilbene A.17.7 μM  Acarbose: 385 μM |
| 269 | Xu, J. *et al.* (2018)^274^ | α-glucosidase (yeast, chromogenic method) | *Quercus liaotungensis* | 19 flavonoids: Flavonol, flavan-3-ol, flavanone  IC_50_ range: 0.52 – 69.38 µM  Kaempferol 3-(6-methylglucuronide): 0.52 ± 0.09 µM  Acarbose: 5.90 ± 0.98 µM |
| 270 | Tundis, R. *et al.* (2016)^275^ | α-glucosidase (*Saccharomyces cerevisiae,* enzymatic method)  α-amylase (porcine pancreatic, reducing sugar method) | *Poncirus trifoliata* | 6 flavanones  **AG:**  IC_50_ range: 4.2 – 64.58 µM  Didymin: 4.20 ± 0.6 µM  Acarbose: 54.99 ± 1.3 µM  **AM:**  IC_50_ range: 4.69 – 70.8 µM  Neoeriocitrin: 4.69 ± 0.9 µM  Acarbose: 77.45 ± 1.8 µM |
| 271 | Islam, Md Nurul *et al.* (2013)^276^ | α-glucosidase (yeast, chromogenic method) | *Artemisia capillaris* | 4 flavonoids: flavone, flavonol  IC_50_ range: 58.93 – 351.71 µM  Quercetin: 58.93 ± 6.69 µM  Acarbose: 130.52 ± 10.01 µM |
| 272 | Mohamed, E. A. *et al.* (2012)^277^ | α-glucosidase (unstated*,* chromogenic method)  α-amylase (unstated, reducing sugar method) | *Orthosiphon stamineus* | 1 flavone  **AG:**  Sinensetin: 0.66 ± 0.025 mg/mL  Acarbose: 1.93 ± 0.281 mg/mL  **AM:**  Sinensetin: 1.13 ± 0.026 mg/mL  Acarbose: 4.89 ± 0.397 mg/mL |
| 273 | Amin, S. *et al.* (2019)^278^ | α-glucosidase (*Saccharomyces cerevisiae,* chromogenic method) | *Dryopteris cycadina* | 3 flavonoids: flavonol, flavanonol, oligomeric  IC_50_ range: 133 – 298 µM  Compound 3: 133 ± 6.9 µM  Acarbose: 290 ± 0.54 µM |
| 274 | Quan, Y. S. *et al.* (2020)^279^ | α-glucosidase (*Saccharomyces cerevisiae,* chromogenic method) | *Hylotelephium erythrostictum* | 6 flavonoids: flavone, flavonol  IC_50_ range: 230.3 – 731.1 µM  Quercetin: 230.3 µM  Acarbose: 822.9 µM |
| 275 | Kim, T. *et al.* (2014)^280^ | α-glucosidase (unstated***,*** chromogenic method) | Commercial  Semi-synthesis | 4 flavonoids: flavan-3-ol, Oligomeric  IC_50_ range: 0.14-176.6 µM  catechin [6' –8]-catechin: 0.14 ± 0.2 µM  Acarbose: 300.1 ± 3.5 µM |
| 276 | Arumugam, B. *et al.* (2016)^281^ | α-glucosidase (*Saccharomyces cerevisiae,* chromogenic method)  α-amylase (Porcine pancreatic, reducing sugar method) | Commercial | 3 flavonoids: Flavonol  **AG:**  IC_50_ range: 14.52 – 78.54 µM  Myricetin: 14.52 ± 1.15 µM  Acarbose: 388.50 ± 1.03 µM  **AM:**  IC_50_ range: 147.30 – 862.90 µM  Myricetin: 147.30 ± 1.57 µM  Acarbose: 2.25 ± 1.03 µM |
| 277 | Kim, D. H. *et al.* (2017)^282^ | α-glucosidase (yeast***,*** chromogenic method) | *Epimedium koreanum* | 2 flavonoids: flavonol  IC_50_ range: 74.42 – 106.59 µM  Icaritin: 74.42 ± 0.01 µM  Acarbose: 101.16 ± 3.69 µM |
| 278 | Söhretoglu D. *et al.* (2018)^283^ | α-glucosidase (*Saccharomyces cerevisiae****,*** chromogenic method) | *Potentilla inclinata* | 5 flavonols  IC_50_ range: 26.31-231.92 µg/ml  Rutin: 26.31 ± 0.02 µg/ml  Acarbose: 31.92 ± 0.17 µg/ml |
| 279 | Han, L. *et al.* (2018)^284^ | α-glucosidase (*Saccharomyces cerevisiae,* chromogenic method) | Commercial | 1 flavonoid: oligomeric flavonoid  Proanthocyanidin B2: 0.23 ± 0.01 µg/mL  Acarbose: 807.08 ± 23.00 µg/mL |
| 280 | Lin, H. C. *et al.* (2010)^285^ | α-glucosidase (*Bacillus stearothermophilus*, chromogenic method) | *Machilus philippinensis* | 6 flavonoids: Oligomeric flavonoid  IC_50_ range: 3.5 - 92.9 µM  Compound 2: 3.5 ± 0.0 µM  Acarbose: 0.049 ± 0.003 µM |
| 281 | Sheikh, Y. *et al.* (2019)^286^ | α-glucosidase (yeast maltase*,* chromogenic method) | *Wendlandia glabrata* | 1 oligomeric flavonoid  Proanthocyanidin A2: 0.27 ± 0.01 µg/mL  Acarbose: 378.73 ± 0.08 µg/mL |
| 282 | Braham, Na *et al.* (2016)^287^ | α-glucosidase (unstated*,* chromogenic method) | *Agrimonia pilosa* | 5 flavonoids: flavone; flavonol  IC_50_ range: 11.2 – 103.3 µM  Quercetin-7-O-b-D-glucoside: 11.2 ± 0.2 µM  Acarbose: 124.2 ± 0.4 µM |
| 283 | Seong, S. H. *et al.* (2016)^288^ | α-glucosidase (yeast; Chromogenic method) | *Puraria lobata* | 8 flavonoids: isoflavonoid  IC_50_ range: 2.37 – 495.03 μM  Genistein: 2.37 ± 0.52 μM  Acarbose: 144.26 ± 10.49 μM |
| 284 | Jung, H. A. *et al.* (2017)^289^ | α-glucosidase (yeast; Chromogenic method) | *Prunus davidiana* | 1 flavonoid: flavanone  Prunin: 317 ± 2.12 μM  Acarbose: 187 ± 1.23 μM |
| 285 | Zhao, Bing Tian *et al.* (2016)^290^ | α-glucosidase (unstated; Chromogenic method) | *Smilax china* | 11 flavonoids: flavone, oligomeric, isoflavonoid, flavanone; flavonol  IC_50_ range: 7.99 – 195.55 μM  Quercetin-4'-*O*-β-D-glucoside: 7.99 ± 0.48 μM  Acarbose: 175.84 ± 1.69 μM |
| 286 | Yan, S. *et al.* (2016)^291^ | α-glucosidase (unstated; Chromogenic method) | *Morella rubra* | 4 flavonoids: anthocyanidin; flavonol  IC_50_ range: 418.8 – 1444.3 μg/mL  myricetin-3-*O*-rhamnoside: 418.8 μg/mL  Acarbose: 383.2 μg/mL |
| 287 | Zhao, Y. *et al.* (2018)^292^ | α-glucosidase (*Saccharomyces cerevisiae*; Chromogenic method)  α-amylase (Porcine pancreatic; Chromogenic method) | *Morus alba* | 9 flavonoids: flavanone; flavone; oligomeric  **AG:**IC_50_ range: 0.60 – 27.15 µM  Kuwanon M: 0.60 ± 0.09 µM  Acarbose: 293.50 ± 34.42 µM  **AM:**IC_50_ range: 1.22 – 69.38 µM  Kuwanon M: 1.22 ± 0.34 µM  Acarbose: 1.51 ± 0.16 µM |
| 288 | Gou, S. H. *et al.* (2016)^293^ | α-glucosidase (yeast; Chromogenic method) | *Glycyrrhiza uralensis* | 6 flavonoids: flavanone; flavanonol; chalcone; isoflavonoid  IC_50_ range: 10.845 - 44.384 μg/mL  Isolicoflavonol: 10.845 ± 0.980 μg/mL  Acarbose: 38.995 ± 1.324 μg/mL |
| 289 | Yang, H. *et al.* (2016)^294^ | α-glucosidase (*Saccharomyces cerevisiae*; MALDI-MS) | Commercial | 1 flavonoid: flavan-3-ol  EGCG: 0.19 ± 0.04 mM  Acarbose: 3.5 ± 0.5 mM |
| 290 | Qu, X. *et al.* (2022)^295^ | α-glucosidase (unstated; Chromogenic method) | *Potentilla bifurca* | 4 flavonoids: flavonol  IC_50_ range: 0.065 – 0.695 mM  QC: 0.065 mM  Acarbose: 4.509 mM |
| 291 | Wang, L. *et al.* (2018)^296^ | α-glucosidase (*Saccharomyces cerevisiae*; Chromogenic method) | *Psidium guajava* | 10 flavonoids: flavan-3-ol; oligomeric; flavonol  IC_50_ range: 4.51 – 58.19 µg/mL  Quercetin: 4.51 ± 0.71 µg/mL  Acarbose: 178.52 ± 27 1.37 µg/mL |
| 292 | Lianwu, Xie *et al.* (2021)^297^ | α-glucosidase (*Saccharomyces cerevisiae*; Chromogenic method) | *Buddleja Flos* | 11 flavonoids: flavonol; flavone  IC_50_ range: 5.11 – 249.37 μg/mL  Apigenin: 5.11 ± 0.85 μg/mL  Acarbose: 195.49 ± 10.05 μg/mL |
| 293 | Yang, Y. *et al.* (2015)^298^ | α-glucosidase (*Saccharomyces cerevisiae*; Chromogenic method) | *Phlomis tuberosa* | 2 flavonoids: flavone; flavonol  IC_50_ range: 0.428 – 0.562 mM  Luteolin: 0.428 ± 0.018 mM  Acarbose: 3.72 ± 0.113 mM |
| 294 | Li, Y. *et al.* (2014)^299^ | α-amylase (*Bacillus sp.*; Chromogenic method) | *Garcinia xanthochymus* | 3 flavonoids: oligomeric  IC_50_ range: 0.97 – 44.59 µg/mL  GB2a: 0.97±0.09 µg/mL  Acarbose: 9.04±1.58 µg/mL |
| 295 | Tao, Y. *et al.* (2013)^300^ | α-glucosidase (*Saccharomyces cerevisiae*; Chromogenic method) | *Morus alba* | 2 flavonoids: flavonol  IC_50_ range: 462.1 – 662.9 μM  Isoquercitrin: 462.1 μM  Acarbose: 465.4 μM |
| 296 | Li, H. *et al.* (2009)^301^ | α-glucosidase (yeast; Chromogenic method) | *Crataegus oxyacantha* | 6 flavonoids: flavone  IC_50_ range: 13.07 – 25.11 μM  Luteolin: 13.07 μM  Acarbose: 228.16 μM |
| 297 | Jiang, W. *et al.* (2015)^302^ | α-glucosidase (unstated; Chromogenic method) | Radix  Astragali | 2 flavonoids: isoflavonoid  IC_50_ range: 20 – 27 μM  Biochanin A: 20 μM  Acarbose: 382 μM |
| 298 | Nguyen, M. T. T. *et al.* (2012)^303^ | α-glucosidase (*Saccharomyces cerevisiae*; Chromogenic method) | *Oroxylum indicum* | 3 flavonoids: flavone  IC_50_ range: 2.13 – 27.91 μM  oroxyloside: 2.13 ± 0.12 μM  Acarbose: 241.85 ± 0.15 μM |
| 299 | Wu, B. *et al.* (2016)^304^ | α-glucosidase (*Saccharomyces cerevisiae*; Chromogenic method) | *Ginkgo Biloba* | 15 flavonoids: flavonol; oligomeric; flavone  IC_50_ range: 1.79 – 694.7 μM  Ginkgetin: 1.79 μM  Acarbose: 93.91 μM |
| 300 | Wang, Y. M. *et al.* (2017)^305^ | α-glucosidase (*Saccharomyces cerevisiae*; Chromogenic method) | *Asterothamnus centrali-asiaticus* | 4 flavonoids: flavone; flavanone  IC_50_ range: 38.9 – 299.7 μM  Apigenin: 38.9 μM  Acarbose: 183.6 μM |
| 301 | Yu, Zhang *et al.* (2015)^306^ | α-glucosidase (*Bacillus stearothermophilus;* Chromogenic method) | *Xanthoceras*  *sorbifolia* | 5 flavonoids: flavanonol; flavonol; oligomeric  IC_50_ range: 1.2 - 95.9 μg/mL  epigallocatechin-(4β→8,2β→*O*-7)-epicatechin: 1.2 μg/mL  Acarbose: 0.038 μg/mL |
| 302 | Xiao, Z. *et al.* (2022)^307^ | α-amylase (porcine pancreatic; Reducing sugar method) | Synthesis; commercial | 5 flavonoids: anthocyanidin, flavonol, flavone  IC_50_ range: 325.54 – 1480.26 μM  Cyanidin: 325.54 ± 31.09 μM  Acarbose: 5.65 ± 1.68 μM |
| 303 | Zhang, H. *et al.* (2013)^308^ | α-glucosidase (rat intestinal; enzymatic method) | *Arachis hypogaea* | 5 flavonoids: flavan-3-ol; oligomeric  IC_50_ range: 0.088 – 0.78 mg/mL  Epicatechin, epicatechin-(2β→*O*→7,4β→8)-[catechin-(6→4β)]-epicatechin: 0.088 mg/mL  Acarbose: 0.0054 mg/mL |
| 304 | Fan, Y. H. *et al.* (2019)^309^ | α-glucosidase (*Bacillus stearothermophilus;* Chromogenic method) | *Glycyrrhiza uralensis*; commercial | 8 flavonoids: flavanone; flavonol  IC_50_ range: 3.7 – 53.4 µg/mL  6-prenylquercetin: 3.7 µg/mL  Acarbose: 0.04 µg/mL |
| 305 | Ajish, K. R. *et al.*  (2015)^310^ | α-glucosidase (unstated; Chromogenic method) | *Zingiber zerumbet* | 4 flavonoids: flavonol  IC_50_ range: 7.88 – 81.16 µM  kaempferol-3-*O*-methylether: 7.88 ± 0.06 µM  Acarbose: 47.2 ± 0.95 µM |
| 306 | Boonsombat, J. *et al.* (2017)^311^ | α-glucosidase (*Saccharomyces cerevisiae*; Chromogenic method) | *Butea superba* | 1 flavonoid: isoflavonoid  Biochanin A: 12.35 ± 0.36 μM  Acarbose: 6.54 ± 0.04 μM |
| 307 | Sun, H. *et al.*  (2018)^312^ | α-glucosidase (*Saccharomyces cerevisiae*; Chromogenic method)  α-amylase (porcine pancreatic; Chromogenic method) | Synthesis | 22 flavonoids: flavone; flavonol; aurone  **AG:**  IC_50_ range: 1.47 – 22.73 μM  5a: 1.47 ± 0.07 μM  Acarbose: 224.70 ± 14.14 μM  **AM:**  IC_50_ range: 6.89 – 10.97 μM  5a: 6.89 ± 1.17 μM  Acarbose: 2.72 ± 0.30 μM |
| 308 | Hari Babu, T. *et al.* (2008)^313^ | α-glucosidase (yeast and rat; Chromogenic method) | *Oroxylum indicum* | 7 flavonoids: flavone  IC_50_ range: 35.43 – 375.55 μM  1b: 35.43 μM  Acarbose: 6.38 μM |
| 309 | Kumar, G. S. *et al.* (2010)^314^ | α-glucosidase (unstated; Chromogenic method) | *Oroxylum indicum* | 6 flavonoids: flavone  IC_50_ range: 25.78 – 512.58 μM  Baicalein: 25.78 μM  Acarbose: 18.63 μM |
| 310 | Cheng, N. *et al.* (2014)^315^ | α-glucosidase (*Saccharomyces cerevisiae*; Chromogenic method) | Commercial; synthesis | 16 flavonoids: flavone  IC_50_ range: 2.406 – 77.730 μM  7-(hexyloxy)-2-(3-(hexyloxy)-4-methoxyphenyl)-5-hydroxy-4*H*-chromen-4-one: 2.406 ± 0.101 μM  Acarbose: 563.601 ± 40.492 μM |
| 311 | Tang, C. *et al.* (2012)^316^ | α-glucosidase (*Saccharomyces cerevisiae*; Chromogenic method) | Synthesis | 4 flavonoids: chalcone  IC_50_ range: 47.5 – 272.1 μM  2b: 47.5 ± 4.1 μM  Acarbose: 362.3 ± 7.6 μM |
| 312 | Saleem, F. *et al.* (2021)^317^ | α-glucosidase (*Saccharomyces cerevisiae*; Chromogenic method)  α-amylase (*Aspergillus oryzae*; Reducing sugar method) | Synthesis | 24 flavonoids: chalcone  **AG:**  IC_50_ range: 26.08 – 89.71 µM  (*E*)-3-(2-Methoxyphenyl)-1-(pyridin-2-yl)prop-2-en-1-one: 26.08 ± 0.43 µM  Acarbose: 18.67 ± 0.09 µM  **AM:**  IC_50_ range: 23.08 – 89.71 µM  (*E*)-3-(2-Methoxyphenyl)-1-(pyridin-2-yl)prop-2-en-1-one: 23.08 ± 0.03 µM  Acarbose: 18.08 ± 0.07 µM |
| 313 | Saidi, I. *et al.* (2022)^318^ | α-amylase (unstated; Reducing sugar method) | Synthesis | 15 flavonoids: flavonol  IC_50_ range: 16.20 – 33.26 µM  6-bromo-3-((3-(4-fluorophenyl)isoxazol-5-yl)methoxy)-2-(4- (trifluoromethyl)phenyl)-4*H*-chromen-4-one: 16.20 ± 0.31 µM  Acarbose: 15.74 ± 0.21 µM |
| 314 | Imran, S. *et al.* (2015)^319^ | α-glucosidase (*Saccharomyces cerevisiae*; Chromogenic method) | Synthesis | 33 flavonoids: chalcone; flavone  IC_50_ range: 15.4 - 840 μM  (*E*)-4-(4-oxo-4*H*-chromen-2-yl)-*N'* -(2,4,6- trihydroxybenzylidene)benzohydrazide: 15.4 ± 0.22 μM  Acarbose: 860.23 ± 6.10 μM |
| 315 | Zhang, Y. *et al.* (2021)^320^ | α-glucosidase (*Saccharomyces cerevisiae*; Chromogenic method) | *Humulus lupulus*; Synthesis | 14 flavonoids: chalcone; oligomeric  IC_50_ range: 0.12 – 22.42 μM  Compound 5d: 0.12 ± 0.02 μM  Acarbose: 188.57 ± 5.52 μM |
| 316 | Liu, J. *et al.* (2014)^321^ | α-glucosidase (*Saccharomyces cerevisiae*; Chromogenic method)  α-amylase (porcine pancreatic; Chromogenic method) | commercial | 1 flavonoid: flavan-3-ol  **AG:**  Catechin: 0.16 mg/mL  Acarbose: 4.67 mg/mL  **AM:**  Catechin: 20.56 mg/mL  Acarbose: 3.78 mg/mL |
| 317 | Chinthala, Y. *et al.* (2015)^322^ | α-glucosidase (rat intestinal; Chromogenic method) | Synthesis | 4 flavonoids: chalcone  IC_50_ range: 67.77 – 127.9 μM  Compound **4m**: 67.77 μM  Acarbose: 23.87 μM |
| 318 | Sun, H. *et al.* (2015)^323^ | α-glucosidase (*Saccharomyces cerevisiae*; Chromogenic method) | Synthesis; commercial | 11 flavonoids: Chalcone; Isoflavonoid; Flavonol  IC_50_ range: 0.9 – 50.05 μM  16: 0.9 μM  Acarbose: 51.32 μM |
| 319 | Imran, S. *et al.* (2016)^324^ | α-glucosidase (*Saccharomyces cerevisiae*; Chromogenic method) | Synthesis | 13 flavonoids: chalcone; flavone  IC_50_ range: 1.26 – 88.66 μM  2-(3-((2,5-bis(trifluoromethyl)benzyl)oxy)phenyl)- 4*H*-chromen-4-one: 1.26 ± 0.01 μM  Acarbose: 38.25 ± 0.12 μM |
| 320 | Mphahlele, M.  *et al.* (2021)^325^ | α-glucosidase (*Saccharomyces cerevisiae*; Chromogenic method)  α-amylase (Human salivary; Chromogenic method) | Synthesis | 8 flavonoids: chalcone  **AG:**  IC_50_ range: 5.1 – 30.7 µM  Compound 3e: 5.1 ± 0.61 µM  Acarbose: 0.95 ± 0.28 µM  **AM:**  IC_50_ range: 1.7 – 19.3 µM  Compound **3e:** 1.7 ± 0.25 µM  Acarbose: 1.03 ± 0.05 µM |
| 321 | Wu, C. *et al.* (2012)^326^ | α-glucosidase (yeast; Chromogenic method) | *Belamcanda chinensis* | 5 flavonoids: flavone; isoflavonoid  IC_50_ range: 74-333 µg/mL  Genistein: 74 µg/mL  Acarbose: 271 µg/mL |
| 322 | He, X. F. *et al.* (2021)^327^ | α-glucosidase (unstated; Chromogenic method) | *Amomum tsao-ko* | 14 flavonoids: flavan-3-ol; oligomeric  IC_50_ range: 3.73 – 693.5 µM  Proanthocyanidin A-2: 3.73 ± 0.88 µM  Acarbose: 193.77 ± 6.23 µM |
| 323 | Habtemariam, S. *et al.* (2012)^328^ | α-glucosidase (*Saccharomyces cerevisiae*; Chromogenic method) | Commercial;  *Erythrina*  *abyssinica* | 2 flavonoids: flavanone  IC_50_ range: 57.5 – 62.5 µM  Eriodictyol: 57.5 ± 13.15 µM  Acarbose: 190.6 ± 16.05 µM |
| 324 | Taslimi, P. *et al.* (2017)^329^ | α-glucosidase (unstated; Chromogenic method) | Commercial | 3 flavonoids: flavone; flavanone  IC_50_ range: 12.64 – 814.33 nM  Naringin: 12.64 nM  Acarbose: 22800 nM |
| 325 | Zhang, L. L. *et al.* (2019)^330^ | α-glucosidase (*Saccharomyces cerevisiae*; Chromogenic method) | Synthesis | 2 flavonoids: Flavan-3-ol  IC_50_ range: 0.95 – 1.12 μM  (-)-Epicatechin: 0.95 ± 0.02 μM  Acarbose: 1250.11 ± 35.63 μM |
| 326 | Chen, Y. *et al.* (2021)^331^ | α-amylase (porcine pancreatic; Reducing sugar method) | Commercial | 3 flavonoids: flavan-3-ol  IC_50_ range: 172.21 – 732.15 μg/mL  ECG: 172.21 ± 0.22 μg/mL  Acarbose: 42.87 ± 0.42 μg/mL |
| 327 | Mai, N. T. T. *et al.* (2012)^332^ | α-glucosidase (*Saccharomyces cerevisiae*; Chromogenic method) | *Artocarpus altilis* | 3 flavonoids: aurone  IC_50_ range: 4.9 – 5.4 µM  Altilisin H: 4.9 ± 0.1 µM  Acarbose: 241.8 ± 0.2 µM |
| 328 | Yuca, H. *et al.* (2021)^333^ | α-glucosidase (*Saccharomyces cerevisiae*; Chromogenic method) | *Elaeagnus angustifolia* | 1 flavonoid: flavonol  Trans-tiliroside: 2128 ± 63 μM  Acarbose: 6561 ± 207 μM |
| 329 | Algethami, F. K. *et al.* (2021)^334^ | α-amylase (*Aspergillus oryzae*; Reducing sugar method) | Synthesis | 15 flavonoids: flavonol  IC_50_ range: 12.6 – 27.6 μM  Compound 3b:12.6 ± 0.2 μM  Acarbose: 12.4 ± 0.1 μM |
| 330 | Srisurichan, S. *et al.* (2015)^335^ | α-glucosidase (*Saccharomyces cerevisiae*; Chromogenic method) | *Holarrhena curtisii* | 1 flavonoid: flavan-3-ol  (-)-gallocatechin: 397.8 μM  Acarbose: 884.6 μM |
| 331 | Liu, Y. *et al.* (2022)^336^ | α-glucosidase (*Saccharomyces cerevisiae*; Chromogenic method) | *Morella rubra* | 18 flavonoids: flavonol  IC_50_ range: 1.32 – 1195.95 μM  Myricetin-3-*O*-(2″-*O*-galloyl)-α-L-rhamnoside: 1.32 ± 0.17 μM  Acarbose: 369.15 ± 6.18 μM |
| 332 | Fei, W. T. *et al.* (2020)^337^ | α-glucosidase (unstated; Chromogenic method) | *Psoralea corylifolia* | 5 flavonoids: flavanone; chalcone; isoflavonoid  IC_50_ range: 53.1 – 149.3 μM  4',1”-dihydroxy-3'-methoxy-6,7-furanflavanone: 53.1 μM  Acarbose: 214.5 μM |
| 333 | El-Hawary, S. S. *et al.* (2021)^338^ | α-glucosidase (*Saccharomyces cerevisiae*; Chromogenic method) | *Gymnocarpos decandrus* | 2 flavonoids: flavone; flavonol  IC_50_ range: 69.18 – 293.3 μg/mL  Quercetin: 69.18 μg/mL  Acarbose: 30.57 μg/mL |
| 334 | Islam, M. N. *et al.* (2014)^339^ | α-glucosidase (yeast; Chromogenic method) | *Artemisia capillaris* | 1 flavonoid: flavone  Vicenin 2: 270.53 ± 6.23 μM  Acarbose: 320.33 ± 4.61 μM |
| 335 | Luyen, N. T. *et al.* (2019)^340^ | α-glucosidase (*Saccharomyces cerevisiae*; Chromogenic method) | *Wedelia trilobata* | 1 flavonoid: flavone  5,7,4′- trihydroxyflavone: 27.54 ± 1.12 μM/mL  Acarbose: 450.56 ± 2.31 μM/mL |
| 336 | Liu, M. *et al.* (2014)^341^ | α-glucosidase (*Saccharomyces cerevisiae*; Chromogenic method) | *Humulus lupulus* | 1 flavonoid: chalcone  Xanthohumol: 8.8 μM  Acarbose: 26.5 μM |
| 337 | Tan, C. *et al.* (2013)^342^ | α-glucosidase (yeast; Chromogenic method) | *Gynura medica* | 4 flavonoids: flavonol  IC_50_ range: 0.10 – 1.67 mg/mL  Rutin: 0.10 ± 0.01 mg/mL  Acarbose: 0.99 ± 0.02 mg/mL |
| 338 | Gong, T. *et al.* (2020)^343^ | α-glucosidase (unstated; Chromogenic method) | *Malus domestica* | 1 flavonoid: chalcone; flavan-3-ol  IC_50_ range: 2.10 – 3.57 mg/mL  Phlorizin: 2.10 ± 0.12 mg/mL  Acarbose: 0.76 μg/mL |
| 339 | Carina Proença, C. *et al.* (2019)^344^ | α-amylase (porcine pancreatic; Chromogenic method) | Commercial, synthesis | 13 flavonoids: flavonol, flavone  IC_50_ range: 44 – 192 μM  D11: 44 ± 3 μM  Acarbose: 1.3 ± 0.2 μM |

*AG: α-glucosidase; AM: α-amylase

# Table S5. Quality assessment of included studies

| **Study  ID** | **Authors (year)** | **Abstract** | **Introduction** | | **Methods** | | | | **Results** | **Discussion** | | **Other  information** |
| --- | --- | --- | --- | --- | --- | --- | --- | --- | --- | --- | --- | --- |
|  |  | **1 Abstract** | **2a Background** | **2b Hypothesis** | | **3 Intervention** | **4 Outcomes** | **10 Statistical methods** | **11 Outcomes and estimation** | **12 Limitations** | **13 Funding** | |
| 1 | Yang Yao *et al.* (2011)^6^ | Yes | Yes | Yes | | Yes | Yes | Yes | Yes | No | Yes | |
| 2 | Van Thanh *et al.* (2022)^7^ | Yes | Yes | No | | Yes | No | Yes | Yes | No | Yes | |
| 3 | El-Nashar, H. A. S. *et al.* (2022)^8^ | No | Yes | Yes | | Yes | Yes | Yes | Yes | No | No | |
| 4 | Jia, C. C. *et al.* (2017)^9^ | Yes | Yes | Yes | | Yes | Yes | Yes | Yes | No | Yes | |
| 5 | Nguyen, Tan Phat *et al.* (2016)^10^ | Yes | Yes | Yes | | Yes | No | No | Yes | No | Yes | |
| 6 | Zhang, Y. *et al.* (2013)^11^ | Yes | Yes | Yes | | Yes | Yes | Yes | Yes | No | Yes | |
| 7 | Wu, S. *et al.* (2019)^12^ | No | Yes | Yes | | Yes | Yes | Yes | Yes | No | Yes | |
| 8 | Thuy, N. T. L. *et al.* (2019)^13^ | Yes | Yes | Yes | | Yes | Yes | Yes | Yes | No | Yes | |
| 9 | Nguyen, T. T. H. *et al.* (2021)^14^ | Yes | Yes | Yes | | Yes | Yes | Yes | Yes | No | Yes | |
| 10 | Liu, Y. *et al.* (2020)^15^ | Yes | No | Yes | | Yes | No | No | Yes | No | Yes | |
| 11 | Zhang, X. H. *et al.* (2020)^16^ | Yes | No | Yes | | Yes | No | No | Yes | No | Yes | |
| 12 | Phuong, N. H. *et al.* (2017)^17^ | No | Yes | Yes | | Yes | Yes | Yes | Yes | Yes | Yes | |
| 13 | Zhou, Q. *et al.* (2022)^18^ | Yes | Yes | Yes | | Yes | Yes | Yes | Yes | Yes | Yes | |
| 14 | Helal, I. E. *et al.* (2022)^19^ | Yes | Yes | Yes | | Yes | Yes | Yes | Yes | Yes | No | |
| 15 | Parveen, A. *et al.* (2020)^20^ | Yes | Yes | Yes | | Yes | Yes | Yes | Yes | Yes | Yes | |
| 16 | Li, K. *et al.* (2020)^21^ | Yes | Yes | Yes | | Yes | Yes | Yes | Yes | No | Yes | |
| 17 | Kim, T. H. (2016)^22^ | Yes | Yes | Yes | | Yes | Yes | Yes | Yes | No | No | |
| 18 | Rocha, S. *et al.* (2019)^23^ | Yes | Yes | Yes | | Yes | Yes | Yes | Yes | Yes | Yes | |
| 19 | Wan, C. *et al.* (2013)^24^ | Yes | Yes | Yes | | Yes | Yes | Yes | Yes | No | No | |
| 20 | Kashchenko, N. I. *et al.* (2018)^25^ | Yes | Yes | Yes | | Yes | No | Yes | Yes | No | Yes | |
| 21 | Lee, Shoei-Sheng *et al.*(2008)^26^ | Yes | Yes | Yes | | Yes | Yes | Yes | Yes | Yes | Yes | |
| 22 | Chang, C. C. *et al.* (2015)^27^ | Yes | Yes | Yes | | Yes | Yes | Yes | Yes | Yes | Yes | |
| 23 | Habtemariam, Solomon (2011)^28^ | Yes | Yes | Yes | | Yes | No | Yes | Yes | Yes | Yes | |
| 24 | Wang,H. *et al.* (2010)^29^ | Yes | Yes | Yes | | Yes | Yes | Yes | Yes | Yes | Yes | |
| 25 | Etsassala, N. G. E. R. *et al.* (2020)^30^ | Yes | Yes | Yes | | Yes | Yes | Yes | Yes | Yes | Yes | |
| 26 | Hussain, Munawar *et al.* (2019)^31^ | Yes | Yes | Yes | | Yes | No | Yes | Yes | No | Yes | |
| 27 | Gao, H. *et al.* (2005)^32^ | Yes | Yes | Yes | | Yes | Yes | No | Yes | No | No | |
| 28 | Gutierrez-Gonzalez, Jose A. *et al.* (2021)^33^ | Yes | Yes | Yes | | Yes | Yes | Yes | Yes | No | No | |
| 29 | Escandon-Rivera, S. *et al.* (2012)^34^ | Yes | Yes | Yes | | Yes | Yes | Yes | Yes | No | Yes | |
| 30 | Kazemi, R. *et al.* (2022)^35^ | Yes | Yes | Yes | | Yes | Yes | Yes | Yes | No | Yes | |
| 31 | Ha, Kim-Ngoc *et al.* (2022)^36^ | Yes | Yes | Yes | | Yes | No | Yes | Yes | No | No | |
| 32 | Matsui, T. *et al.* (2001)^37^ | Yes | Yes | Yes | | Yes | Yes | No | Yes | Yes | No | |
| 33 | Nguyen, T. N. T. *et al.* (2022)^38^ | Yes | Yes | Yes | | Yes | Yes | Yes | Yes | No | Yes | |
| 34 | Joycharat, N. *et al.* (2018)^39^ | Yes | Yes | Yes | | Yes | Yes | Yes | Yes | No | Yes | |
| 35 | Vi, L. N. T. *et al.* (2022)^40^ | Yes | Yes | Yes | | Yes | Yes | Yes | Yes | No | Yes | |
| 36 | Nguyen, P. T. M. *et al.* (2019)^41^ | Yes | Yes | Yes | | Yes | Yes | Yes | Yes | No | Yes | |
| 37 | Dej-Adisai, S. *et al.* (2021)^42^ | Yes | Yes | Yes | | Yes | Yes | No | Yes | No | Yes | |
| 38 | Tabussum, A. *et al.* (2013)^43^ | Yes | Yes | Yes | | Yes | Yes | Yes | Yes | No | Yes | |
| 39 | Lima Júnior, J. P. D. *et al.* (2021)^44^ | Yes | Yes | Yes | | Yes | Yes | Yes | Yes | Yes | Yes | |
| 40 | Xie, H. *et al.* (2020)^45^ | Yes | Yes | Yes | | Yes | Yes | No | Yes | No | Yes | |
| 41 | Homoki, J. R. *et al.* (2016)^46^ | Yes | Yes | Yes | | Yes | No | Yes | Yes | Yes | Yes | |
| 42 | Swilam, N. *et al.* (2022)^47^ | Yes | Yes | Yes | | Yes | Yes | Yes | Yes | Yes | Yes | |
| 43 | Wang, Y. *et al.* (2013)^48^ | Yes | Yes | Yes | | Yes | Yes | Yes | Yes | Yes | No | |
| 44 | Gulçin, I. *et al.* (2018)^49^ | Yes | Yes | Yes | | Yes | Yes | Yes | Yes | No | Yes | |
| 45 | Junejo, J. A. *et al.* (2021)^50^ | Yes | Yes | Yes | | Yes | Yes | Yes | Yes | Yes | Yes | |
| 46 | Varghese, G. K. *et al.* (2013)^51^ | Yes | Yes | Yes | | Yes | No | Yes | Yes | Yes | Yes | |
| 47 | Zhu, G. *et al.* (2019)^52^ | Yes | Yes | Yes | | Yes | Yes | Yes | Yes | No | No | |
| 48 | El Ridhasya, F. *et al.* (2020)^53^ | Yes | Yes | Yes | | Yes | No | Yes | Yes | No | Yes | |
| 49 | Ablat, A. *et al.* (2017)^54^ | Yes | Yes | Yes | | Yes | Yes | Yes | Yes | Yes | Yes | |
| 50 | Laishram, S. *et al.* (2015)^55^ | Yes | Yes | Yes | | Yes | No | Yes | Yes | No | Yes | |
| 51 | Jo, Y. H. *et al.* (2021)^56^ | Yes | Yes | Yes | | Yes | No | Yes | Yes | No | Yes | |
| 52 | Yoshikawa, M. *et al.* (1998)^57^ | Yes | Yes | Yes | | Yes | No | Yes | Yes | No | No | |
| 53 | Demir, Y. *et al.* (2019)^58^ | Yes | Yes | Yes | | Yes | Yes | No | Yes | No | No | |
| 54 | Ahmed, S. *et al.* (2019)^59^ | Yes | Yes | Yes | | Yes | No | Yes | Yes | No | Yes | |
| 55 | Qun, Sun *et al.* (2017)^60^ | Yes | Yes | Yes | | Yes | Yes | Yes | Yes | No | Yes | |
| 56 | Bui, T. T. *et al.* (2022)^61^ | Yes | Yes | Yes | | Yes | Yes | Yes | Yes | No | Yes | |
| 57 | Hlila, Malek Besbes *et al.* (2017)^62^ | Yes | Yes | Yes | | Yes | Yes | Yes | Yes | No | Yes | |
| 58 | Vu, N. K. *et al.* (2020)^63^ | Yes | Yes | Yes | | Yes | No | Yes | Yes | No | Yes | |
| 59 | Fan, P. *et al.* (2010)^64^ | Yes | Yes | Yes | | Yes | Yes | Yes | Yes | No | Yes | |
| 60 | Choi, C. I. *et al.* (2015)^65^ | Yes | Yes | Yes | | Yes | Yes | Yes | Yes | Yes | Yes | |
| 61 | Yue, Y. *et al.* (2018)^66^ | Yes | Yes | Yes | | Yes | Yes | Yes | Yes | Yes | Yes | |
| 62 | Choi, C. I. *et al.* (2016)^67^ | Yes | Yes | Yes | | Yes | Yes | Yes | Yes | No | Yes | |
| 63 | Wan, C. *et al.* (2012)^68^ | Yes | Yes | Yes | | Yes | Yes | Yes | Yes | No | No | |
| 64 | Wang, Y. *et al.* (2021)^69^ | Yes | Yes | Yes | | Yes | Yes | Yes | Yes | No | No | |
| 65 | Polbuppha, I. *et al.* (2017)^70^ | Yes | Yes | Yes | | Yes | Yes | Yes | Yes | No | Yes | |
| 66 | Supasuteekul, C.*et al.* (2016)^71^ | Yes | Yes | Yes | | Yes | Yes | Yes | Yes | No | Yes | |
| 67 | Zhao, J. Q. *et al.* (2017)^72^ | Yes | Yes | Yes | | Yes | Yes | Yes | Yes | No | Yes | |
| 68 | Zhang, L. *et al.* (2016)^73^ | Yes | Yes | Yes | | Yes | Yes | Yes | Yes | Yes | Yes | |
| 69 | Anh, L. T. T. *et al.* (2022)^74^ | Yes | Yes | Yes | | Yes | Yes | Yes | Yes | No | Yes | |
| 70 | Sahnoun, M. *et al.* (2018)^75^ | Yes | Yes | Yes | | Yes | Yes | Yes | Yes | No | Yes | |
| 71 | Ma, J. *et al.* (2017)^76^ | Yes | Yes | Yes | | Yes | No | No | Yes | No | Yes | |
| 72 | Monteiro, A. D. O. *et al.* (2022)^77^ | Yes | Yes | Yes | | Yes | No | No | Yes | No | Yes | |
| 73 | Song, M. *et al.* (2022)^78^ | Yes | Yes | Yes | | Yes | Yes | Yes | Yes | No | Yes | |
| 74 | Tian, J. L. *et al.* (2021)^79^ | Yes | Yes | Yes | | Yes | Yes | Yes | Yes | Yes | Yes | |
| 75 | Jing, Pan *et al.* (2018)^80^ | Yes | Yes | Yes | | Yes | Yes | Yes | Yes | No | Yes | |
| 76 | Nickavar, B. *et al.* (2013)^81^ | Yes | Yes | Yes | | Yes | Yes | Yes | Yes | No | No | |
| 77 | Li, S. *et al.* (2022)^82^ | Yes | Yes | Yes | | Yes | Yes | Yes | Yes | Yes | Yes | |
| 78 | Renda, G. *et al.* (2017)^83^ | Yes | Yes | Yes | | Yes | No | Yes | Yes | No | Yes | |
| 79 | Jibril, S. *et al.* (2017)^84^ | Yes | Yes | Yes | | Yes | Yes | Yes | Yes | No | Yes | |
| 80 | Nickavar, B. *et al.* (2010)^85^ | Yes | Yes | Yes | | Yes | Yes | Yes | Yes | No | No | |
| 81 | Khalid, M. F. *et al.* (2022)^86^ | Yes | Yes | Yes | | Yes | Yes | Yes | Yes | Yes | Yes | |
| 82 | Zhou, H. *et al.* (2017)^87^ | Yes | Yes | Yes | | Yes | Yes | Yes | Yes | Yes | Yes | |
| 83 | Ha, M. T. *et al.* (2018)^88^ | Yes | Yes | Yes | | Yes | No | Yes | Yes | Yes | Yes | |
| 84 | Tajudeen Bale, A. *et al.* (2018)^89^ | Yes | Yes | Yes | | Yes | Yes | Yes | Yes | Yes | Yes | |
| 85 | Ali, M. *et al.* (2020)^90^ | Yes | Yes | Yes | | Yes | Yes | Yes | Yes | Yes | Yes | |
| 86 | Fidelis. Q. C. *et al.* (2019)^91^ | Yes | Yes | Yes | | Yes | Yes | Yes | Yes | No | No | |
| 87 | Tian, X. *et al.* (2020)^92^ | Yes | Yes | Yes | | Yes | Yes | Yes | Yes | No | Yes | |
| 88 | Uddin, S. *et al.* (2022)^93^ | Yes | Yes | Yes | | Yes | Yes | Yes | Yes | Yes | Yes | |
| 89 | Costa Silva, T. D. *et al.* (2019)^94^ | Yes | Yes | Yes | | Yes | Yes | Yes | Yes | Yes | Yes | |
| 90 | Adhikari-Devkota, A. *et al.* (2019)^95^ | Yes | Yes | Yes | | Yes | Yes | No | Yes | No | Yes | |
| 91 | Paul, Swastika *et al.* (2020)^96^ | Yes | Yes | Yes | | Yes | No | No | Yes | No | Yes | |
| 92 | Jeong, S. Y. *et al.* (2015)^97^ | Yes | Yes | Yes | | Yes | Yes | Yes | Yes | No | Yes | |
| 93 | Linh, N. T. T. *et al.* (2022)^98^ | Yes | Yes | Yes | | Yes | Yes | Yes | Yes | No | Yes | |
| 94 | Abdullah, Nur Hakimah *et al.* (2016)^99^ | Yes | Yes | Yes | | Yes | Yes | Yes | Yes | No | Yes | |
| 95 | Lee, H. E. *et al.* (2017)^100^ | Yes | Yes | Yes | | Yes | Yes | Yes | Yes | No | Yes | |
| 96 | Kuroda, M. *et al.* (2012)^101^ | Yes | Yes | Yes | | Yes | No | No | Yes | No | No | |
| 97 | Lam, S. H. *et al.* (2019)^102^ | Yes | Yes | Yes | | Yes | Yes | Yes | Yes | No | Yes | |
| 98 | Huang, Q. *et al.* (2021)^103^ | Yes | Yes | Yes | | Yes | Yes | Yes | Yes | Yes | Yes | |
| 99 | Xu, L. *et al.* (2019)^104^ | Yes | Yes | Yes | | Yes | Yes | Yes | Yes | No | Yes | |
| 100 | Zeng, Y. R. *et al.* (2018)^105^ | Yes | Yes | Yes | | Yes | Yes | Yes | Yes | Yes | Yes | |
| 101 | Qurtam, A. A. *et al.* (2021)^106^ | Yes | Yes | Yes | | Yes | No | Yes | Yes | No | Yes | |
| 102 | Sahnoun, M. *et al.* (2017)^107^ | Yes | Yes | Yes | | Yes | Yes | Yes | Yes | No | Yes | |
| 103 | Li, Y. Q. *et al.* (2009)^108^ | Yes | Yes | Yes | | Yes | No | No | Yes | Yes | Yes | |
| 104 | Jia, Y. *et al.* (2019)^109^ | Yes | Yes | Yes | | Yes | Yes | Yes | Yes | No | Yes | |
| 105 | Wang, L. *et al.* (2022)^110^ | Yes | Yes | Yes | | Yes | Yes | Yes | Yes | No | Yes | |
| 106 | Olennikov, D. N. *et al.* (2014)^111^ | No | Yes | Yes | | Yes | Yes | Yes | Yes | No | Yes | |
| 107 | Lyu, Q. *et al.* (2019)^112^ | Yes | Yes | Yes | | Yes | Yes | Yes | Yes | No | Yes | |
| 108 | Li, Gang *et al.* (2020)^113^ | Yes | Yes | Yes | | Yes | No | Yes | Yes | No | Yes | |
| 109 | Wang, X. L. *et al.* (2017)^114^ | Yes | Yes | Yes | | Yes | Yes | Yes | Yes | No | No | |
| 110 | Mo, Q. G. *et al.* (2021)^115^ | Yes | Yes | Yes | | Yes | Yes | Yes | Yes | No | Yes | |
| 111 | Nazir, N. *et al.* (2020)^116^ | Yes | Yes | Yes | | Yes | Yes | Yes | Yes | Yes | Yes | |
| 112 | Oueslati, Mohamed Habib *et al.* (2020)^117^ | Yes | Yes | Yes | | Yes | Yes | Yes | Yes | No | Yes | |
| 113 | Li, Q. *et al.* (2015)^118^ | Yes | Yes | Yes | | Yes | Yes | Yes | Yes | No | Yes | |
| 114 | Ashraf, J. *et al.* (2020)^119^ | Yes | Yes | Yes | | Yes | Yes | Yes | Yes | No | Yes | |
| 115 | Yang, J. R. *et al.* (2015)^120^ | Yes | Yes | Yes | | Yes | Yes | Yes | Yes | No | Yes | |
| 116 | Nguyen, D. H. *et al.* (2020)^121^ | Yes | Yes | Yes | | Yes | Yes | Yes | Yes | No | Yes | |
| 117 | He, X. F. *et al.* (2021)^122^ | Yes | Yes | Yes | | Yes | No | Yes | Yes | No | Yes | |
| 118 | Bo-wei, Zhang *et al.* (2017)^123^ | Yes | Yes | Yes | | Yes | Yes | Yes | Yes | No | Yes | |
| 119 | Lv, Q. *et al.* (2019)^124^ | Yes | Yes | Yes | | Yes | Yes | No | Yes | No | Yes | |
| 120 | Sohretoglu, Didem *et al.* (2018)^125^ | Yes | Yes | Yes | | Yes | Yes | Yes | Yes | No | Yes | |
| 121 | Su, Z. R. *et al.* (2015)^126^ | Yes | Yes | Yes | | Yes | Yes | Yes | Yes | No | Yes | |
| 122 | Ray, S. *et al.*(2014)^127^ | Yes | Yes | Yes | | Yes | Yes | Yes | Yes | Yes | Yes | |
| 123 | Giang Thanh Thi, Ho *et al.* (2017)^128^ | Yes | Yes | Yes | | Yes | No | Yes | Yes | Yes | Yes | |
| 124 | Zhou, Y. *et al.* (2021)^129^ | Yes | Yes | Yes | | Yes | Yes | Yes | Yes | Yes | Yes | |
| 125 | Krishna, M. S. *et al.* (2015)^130^ | Yes | Yes | Yes | | Yes | Yes | Yes | Yes | Yes | Yes | |
| 126 | Su, J. *et al.* (2019)^131^ | Yes | Yes | Yes | | Yes | Yes | Yes | Yes | No | Yes | |
| 127 | Nickavar, B. *et al.* (2011)^132^ | Yes | Yes | Yes | | Yes | Yes | Yes | Yes | No | No | |
| 128 | Zhang, Y. *et al.* (2020)^133^ | Yes | Yes | Yes | | Yes | Yes | Yes | Yes | No | Yes | |
| 129 | Minh Anh Thu, Phan *et al.* (2013)^134^ | Yes | Yes | Yes | | Yes | Yes | Yes | Yes | No | Yes | |
| 130 | Huang, D. *et al.* (2015)^135^ | Yes | Yes | Yes | | Yes | Yes | Yes | Yes | Yes | Yes | |
| 131 | Numonov, S. *et al.* (2017)^136^ | Yes | Yes | Yes | | Yes | Yes | Yes | Yes | Yes | Yes | |
| 132 | Dubey, K. *et al.* (2021)^137^ | Yes | Yes | Yes | | Yes | Yes | Yes | Yes | Yes | Yes | |
| 133 | Li, M. *et al.* (2022)^138^ | Yes | Yes | Yes | | Yes | No | Yes | Yes | Yes | Yes | |
| 134 | Tang, H. *et al.* (2020)^139^ | Yes | Yes | Yes | | Yes | Yes | Yes | Yes | No | Yes | |
| 135 | Liumin, Wang *et al.* (2022)^140^ | Yes | Yes | Yes | | Yes | Yes | Yes | Yes | No | Yes | |
| 136 | Jiang, Ping *et al.* (2021)^141^ | Yes | Yes | Yes | | Yes | No | Yes | Yes | Yes | Yes | |
| 137 | Terfassi, S. *et al.* (2022)^142^ | Yes | Yes | Yes | | Yes | Yes | Yes | Yes | Yes | Yes | |
| 138 | Wang, X. *et al.* (2017)^143^ | Yes | Yes | Yes | | Yes | Yes | Yes | Yes | No | Yes | |
| 139 | Dhameja, M. *et al.* (2022)^144^ | Yes | Yes | Yes | | Yes | Yes | Yes | Yes | No | Yes | |
| 140 | Dao, T. B. N. *et al.* (2021)^145^ | Yes | Yes | Yes | | Yes | Yes | Yes | Yes | No | Yes | |
| 141 | Li, R. *et al.* (2019)^146^ | Yes | Yes | Yes | | Yes | Yes | Yes | Yes | No | Yes | |
| 142 | Lin, H. C. *et al.* (2011)^147^ | Yes | Yes | Yes | | Yes | No | Yes | Yes | No | Yes | |
| 143 | Zhang, X. *et al.* (2013)^148^ | Yes | Yes | Yes | | Yes | Yes | Yes | Yes | No | Yes | |
| 144 | Praparatana, R. *et al.* (2022)^149^ | Yes | Yes | Yes | | Yes | Yes | Yes | Yes | No | Yes | |
| 145 | Barber, E. *et al.* (2021)^150^ | Yes | Yes | Yes | | Yes | Yes | Yes | Yes | No | Yes | |
| 146 | Lo Piparo, E. *et al.* (2008)^151^ | Yes | Yes | Yes | | Yes | No | Yes | Yes | No | Yes | |
| 147 | Abbasi, B. *et al.* (2014)^152^ | Yes | Yes | Yes | | Yes | Yes | Yes | Yes | No | Yes | |
| 148 | Asghari, B. *et al.* (2015)^153^ | Yes | Yes | Yes | | Yes | Yes | Yes | Yes | No | Yes | |
| 149 | Devkota, H. P. *et al.* (2021)^154^ | Yes | Yes | Yes | | Yes | No | Yes | Yes | Yes | Yes | |
| 150 | Manaharan, T. et al. (2012)^155^ | Yes | Yes | Yes | | Yes | Yes | Yes | Yes | No | Yes | |
| 151 | Hong, H. C. *et al.* (2013)^156^ | Yes | Yes | Yes | | Yes | Yes | Yes | Yes | No | Yes | |
| 152 | Lin, Y. S. *et al.* (2014)^157^ | Yes | No | Yes | | Yes | Yes | Yes | Yes | No | Yes | |
| 153 | Akoro, S. M. *et al.* (2020)^158^ | Yes | Yes | Yes | | Yes | Yes | Yes | Yes | No | Yes | |
| 154 | Kiruthiga, N. *et al.* (2021)^159^ | Yes | Yes | Yes | | Yes | Yes | Yes | Yes | No | Yes | |
| 155 | Shan-Shan, Zhang *et al.* (2022)^160^ | Yes | Yes | Yes | | Yes | Yes | Yes | Yes | No | Yes | |
| 156 | Sichaem, J. *et al.* (2020)^161^ | Yes | Yes | Yes | | Yes | Yes | No | Yes | No | Yes | |
| 157 | Silva, E. L. *et al.* (2016)^162^ | Yes | Yes | Yes | | Yes | Yes | Yes | Yes | No | Yes | |
| 158 | Jeong, G. H. *et al.* (2017)^163^ | Yes | Yes | Yes | | Yes | No | Yes | Yes | No | No | |
| 159 | Sengupta, S. *et al.* (2009)^164^ | Yes | Yes | Yes | | Yes | No | Yes | Yes | No | No | |
| 160 | Zhang, Y. *et al.* (2022)^165^ | Yes | Yes | Yes | | Yes | No | Yes | Yes | No | Yes | |
| 161 | Deutschlander, M. S. *et al.* (2011)^166^ | Yes | Yes | Yes | | Yes | No | Yes | Yes | No | No | |
| 162 | Milella, L. *et al.* (2016)^167^ | Yes | Yes | Yes | | Yes | Yes | Yes | Yes | No | Yes | |
| 163 | Jin, D. X. *et al.* (2021)^168^ | Yes | Yes | Yes | | Yes | Yes | Yes | Yes | No | Yes | |
| 164 | Chen, J. *et al.* (2016)^169^ | Yes | Yes | Yes | | Yes | Yes | Yes | Yes | Yes | Yes | |
| 165 | Proença, C. *et al.* (2017)^170^ | Yes | Yes | Yes | | Yes | No | Yes | Yes | No | Yes | |
| 166 | Yan, J. *et al.* (2014)^171^ | Yes | Yes | Yes | | Yes | Yes | Yes | Yes | No | Yes | |
| 167 | Kim, J. H. *et al.* (2017)^172^ | Yes | Yes | Yes | | Yes | No | Yes | Yes | No | Yes | |
| 168 | Liu, Y. *et al.* (2020)^173^ | Yes | Yes | Yes | | Yes | Yes | Yes | Yes | No | Yes | |
| 169 | Le, T. K. D. *et al.* (2022)^174^ | Yes | Yes | Yes | | Yes | Yes | Yes | Yes | No | Yes | |
| 170 | Flores-Bocanegra, L. *et al.* (2015)^175^ | Yes | Yes | Yes | | Yes | Yes | Yes | Yes | No | Yes | |
| 171 | Tasnuva, S. T. (2019)^176^ | Yes | Yes | Yes | | Yes | Yes | Yes | Yes | No | Yes | |
| 172 | Hou, Z. W. *et al.* (2022)^177^ | Yes | Yes | Yes | | Yes | Yes | Yes | Yes | No | Yes | |
| 173 | Dendup, T. *et al.* (2014)^178^ | Yes | Yes | Yes | | Yes | No | Yes | Yes | No | Yes | |
| 174 | Suthiphasilp, V. *et al.* (2021)^179^ | Yes | Yes | Yes | | Yes | No | No | Yes | No | Yes | |
| 175 | Chen, Y. G. *et al.* (2013)^180^ | Yes | Yes | Yes | | Yes | Yes | Yes | Yes | No | Yes | |
| 176 | Chen, J. G. *et al.* (2020)^181^ | Yes | Yes | Yes | | Yes | Yes | Yes | Yes | Yes | Yes | |
| 177 | Didem Söhretoglu. *et al.* (2017)^182^ | Yes | Yes | Yes | | Yes | Yes | Yes | Yes | No | No | |
| 178 | Renda, G. *et al.* (2018)^183^ | Yes | Yes | Yes | | Yes | Yes | Yes | Yes | Yes | Yes | |
| 179 | Meesakul, P. *et al.* (2019)^184^ | Yes | Yes | Yes | | Yes | No | No | Yes | No | Yes | |
| 180 | G. López-Angulo *et al.* (2022)^185^ | Yes | Yes | Yes | | Yes | Yes | Yes | Yes | Yes | Yes | |
| 181 | Ye, R. *et al.* (2017)^186^ | Yes | Yes | Yes | | Yes | Yes | Yes | Yes | No | Yes | |
| 182 | Duong, T. H. *et al.* (2021)^187^ | Yes | Yes | Yes | | Yes | Yes | Yes | Yes | No | Yes | |
| 183 | Gabriel Monzón Daza *et al.* (2021)^188^ | Yes | Yes | Yes | | Yes | Yes | Yes | Yes | No | Yes | |
| 184 | Assefa, S. T. *et al.* (2021)^189^ | Yes | Yes | Yes | | Yes | No | Yes | Yes | No | Yes | |
| 185 | Fang, H. L. *et al.* (2022)^190^ | Yes | Yes | Yes | | Yes | Yes | Yes | Yes | No | Yes | |
| 186 | Tao, Y. *et al.* (2013)^191^ | Yes | Yes | Yes | | Yes | No | No | Yes | No | Yes | |
| 187 | Vonia, S. *et al.* (2022)^192^ | Yes | Yes | Yes | | Yes | Yes | Yes | Yes | No | Yes | |
| 188 | Yoon, K. D. *et al.* (2020)^193^ | Yes | Yes | Yes | | Yes | Yes | Yes | Yes | No | Yes | |
| 189 | Su, H. *et al.* (2020)^194^ | Yes | Yes | Yes | | Yes | Yes | Yes | Yes | No | Yes | |
| 190 | Wang, W. *et al.* (2016)^195^ | Yes | Yes | Yes | | Yes | Yes | Yes | Yes | No | Yes | |
| 191 | Akkarachiyasit, S. *et al.* (2011)^196^ | Yes | Yes | Yes | | Yes | Yes | Yes | Yes | No | Yes | |
| 192 | Hu, Y. C. *et al.* (2012)^197^ | Yes | Yes | Yes | | Yes | Yes | Yes | Yes | Yes | Yes | |
| 193 | Lian-Xin. *et al.* (2019)^198^ | Yes | Yes | Yes | | Yes | Yes | Yes | Yes | Yes | Yes | |
| 194 | Liao, G. *et al.* (2020)^199^ | Yes | Yes | Yes | | Yes | Yes | Yes | Yes | No | Yes | |
| 195 | Yang, J. *et al.* (2021)^200^ | No | Yes | Yes | | Yes | Yes | Yes | Yes | Yes | Yes | |
| 196 | Kwon, R. H. *et al.* (2022)^201^ | Yes | Yes | Yes | | Yes | No | Yes | Yes | Yes | Yes | |
| 197 | Fang, Hua. *et al.* (2018)^202^ | Yes | Yes | Yes | | Yes | Yes | Yes | Yes | Yes | Yes | |
| 198 | Sheliya, M. A. *et al.* (2015)^203^ | Yes | Yes | Yes | | Yes | Yes | Yes | Yes | No | Yes | |
| 199 | Pyner, A. *et al.* (2017)^204^ | Yes | Yes | Yes | | Yes | Yes | Yes | Yes | Yes | Yes | |
| 200 | Tuan, N. N. *et al.* (2022)^205^ | Yes | Yes | Yes | | Yes | Yes | Yes | Yes | Yes | Yes | |
| 201 | Sun, J. *et al.* (2018)^206^ | Yes | Yes | Yes | | Yes | Yes | Yes | Yes | No | Yes | |
| 202 | Luyen, N. T. *et al.* (2013)^207^ | Yes | Yes | Yes | | Yes | No | Yes | Yes | No | Yes | |
| 203 | Umamaheswari, S. *et al.* (2019)^208^ | Yes | Yes | Yes | | Yes | Yes | Yes | Yes | No | Yes | |
| 204 | Akkarachiyasit, S. *et al.* (2010)^209^ | Yes | Yes | Yes | | Yes | No | Yes | Yes | No | Yes | |
| 205 | Han, L. *et al.* (2022)^210^ | Yes | Yes | Yes | | Yes | No | No | Yes | No | Yes | |
| 206 | Kim, J. H. *et al.* (2018)^211^ | Yes | Yes | Yes | | Yes | No | Yes | Yes | No | Yes | |
| 207 | Xu, L. *et al.* (2019)^212^ | Yes | Yes | Yes | | Yes | Yes | Yes | Yes | No | Yes | |
| 208 | Sadeghi, M. *et al.* (2022)^213^ | Yes | Yes | Yes | | Yes | Yes | Yes | Yes | No | Yes | |
| 209 | Li, K. *et al.* (2018)^214^ | Yes | Yes | Yes | | Yes | Yes | Yes | Yes | No | Yes | |
| 210 | Tundis, R. *et al.* (2007)^215^ | Yes | Yes | Yes | | Yes | Yes | Yes | Yes | No | Yes | |
| 211 | Peng, X. *et al.* (2016)^216^ | Yes | Yes | Yes | | Yes | No | Yes | Yes | No | Yes | |
| 212 | Li, Zeng. *et al.* (2016)^217^ | Yes | Yes | Yes | | Yes | Yes | Yes | Yes | No | Yes | |
| 213 | Wu, X. *et al.* (2019)^218^ | Yes | Yes | Yes | | Yes | Yes | Yes | Yes | No | Yes | |
| 214 | Zeng, L. *et al.* (2016)^219^ | Yes | Yes | Yes | | Yes | Yes | Yes | Yes | Yes | Yes | |
| 215 | Mengting, Ni. *et al.* (2020)^220^ | Yes | Yes | Yes | | Yes | Yes | Yes | Yes | Yes | Yes | |
| 216 | Dong, H. Q. *et al.* (2012)^221^ | Yes | Yes | Yes | | Yes | Yes | Yes | Yes | No | Yes | |
| 217 | Uddin, Md Josim. *et al.* (2022)^222^ | Yes | Yes | Yes | | Yes | No | Yes | Yes | No | Yes | |
| 218 | Zhao, L. *et al.* (2020)^223^ | Yes | Yes | Yes | | Yes | Yes | Yes | Yes | No | Yes | |
| 219 | Imran, M. *et al.* (2021)^224^ | Yes | Yes | Yes | | Yes | Yes | Yes | Yes | No | Yes | |
| 220 | Ha, L. M. *et al.* (2018)^225^ | Yes | Yes | No | | Yes | No | Yes | Yes | No | Yes | |
| 221 | Yi, J. *et al.* (2022)^226^ | Yes | Yes | Yes | | Yes | Yes | Yes | Yes | No | Yes | |
| 222 | Ha, T. J. *et al.* (2018)^227^ | Yes | Yes | Yes | | Yes | Yes | Yes | Yes | No | Yes | |
| 223 | Costa *et al.* (2015)^228^ | Yes | Yes | Yes | | Yes | Yes | Yes | Yes | No | Yes | |
| 224 | Park, Mi Jin *et al.* (2020)^229^ | Yes | Yes | Yes | | Yes | Yes | Yes | Yes | No | Yes | |
| 225 | Marmouzi, I. *et al.* (2021)^230^ | Yes | Yes | Yes | | Yes | No | Yes | Yes | No | No | |
| 226 | Jiang, M. Y. *et al.* (2020)^231^ | Yes | Yes | Yes | | Yes | No | Yes | Yes | No | Yes | |
| 227 | Keskes, H. *et al.* (2017)^232^ | Yes | Yes | Yes | | Yes | Yes | Yes | Yes | No | Yes | |
| 228 | Astiti, M. A. *et al.* (2021)^233^ | Yes | Yes | Yes | | Yes | Yes | Yes | Yes | No | Yes | |
| 229 | Wang, Y. L. *et al.* (2019)^234^ | Yes | Yes | Yes | | Yes | Yes | No | Yes | No | Yes | |
| 230 | Wu, Q. *et al.* (2019)^235^ | Yes | Yes | Yes | | Yes | Yes | Yes | Yes | No | Yes | |
| 231 | Matsui, T. *et al.* (2002)^236^ | Yes | Yes | Yes | | Yes | No | No | Yes | No | Yes | |
| 232 | Djeujo, F. M. *et al.* (2022)^237^ | Yes | Yes | Yes | | Yes | Yes | Yes | Yes | No | Yes | |
| 233 | Visvanathan, R. *et al.* (2021)^238^ | Yes | Yes | Yes | | Yes | Yes | Yes | Yes | Yes | Yes | |
| 234 | Kim, J. H. *et al.* (2019)^239^ | Yes | Yes | Yes | | Yes | Yes | Yes | Yes | No | Yes | |
| 235 | Wang, X. *et al.* (2022)^240^ | Yes | Yes | Yes | | Yes | Yes | Yes | Yes | No | Yes | |
| 236 | Hyun, T. K. *et al.* (2014)^241^ | Yes | Yes | Yes | | Yes | Yes | Yes | Yes | No | Yes | |
| 237 | Şöhretoğlu, D. *et al.* (2022)^242^ | Yes | Yes | Yes | | Yes | Yes | Yes | Yes | No | Yes | |
| 238 | Priscilla, D. H. *et al.* (2014)^243^ | Yes | Yes | Yes | | Yes | Yes | Yes | Yes | No | Yes | |
| 239 | Sun, H. *et al.* (2017)^244^ | Yes | Yes | Yes | | Yes | No | Yes | Yes | No | Yes | |
| 240 | Tran, C. L. *et al.* (2021)^245^ | Yes | Yes | Yes | | Yes | Yes | Yes | Yes | No | No | |
| 241 | Shi, Z. F. *et al.* (2016)^246^ | Yes | Yes | Yes | | Yes | Yes | Yes | Yes | No | No | |
| 242 | Qin, N. B. *et al.* (2017)^247^ | Yes | Yes | Yes | | Yes | Yes | Yes | Yes | No | Yes | |
| 243 | Li, N. *et al.* (2020)^248^ | Yes | Yes | Yes | | Yes | Yes | Yes | Yes | No | Yes | |
| 244 | Dat, N. T. *et al.* (2016)^249^ | Yes | Yes | Yes | | Yes | No | No | Yes | No | Yes | |
| 245 | Do, L. M. T. *et al.* (2022)^250^ | Yes | Yes | Yes | | Yes | Yes | Yes | Yes | No | Yes | |
| 246 | Janibekov, A. A. *et al.* (2018)^251^ | Yes | Yes | Yes | | Yes | Yes | Yes | Yes | No | No | |
| 247 | Ranga Rao, R. *et al.* (2009)^252^ | Yes | Yes | Yes | | Yes | Yes | Yes | Yes | No | Yes | |
| 248 | Zhang, L. *et al.* (2015)^253^ | Yes | Yes | Yes | | Yes | Yes | Yes | Yes | No | Yes | |
| 249 | Wang, M. *et al.* (2015)^254^ | Yes | No | Yes | | Yes | Yes | Yes | Yes | No | Yes | |
| 250 | He, X. F. *et al.* (2020)^255^ | Yes | Yes | Yes | | Yes | No | Yes | Yes | No | Yes | |
| 251 | Ren, D. *et al.* (2018)^256^ | No | Yes | No | | Yes | Yes | Yes | Yes | No | Yes | |
| 252 | Nile, A. *et al.* (2021)^257^ | Yes | Yes | Yes | | No | No | Yes | Yes | No | Yes | |
| 253 | Nguyen, V. B. *et al.* (2018)^258^ | Yes | Yes | Yes | | Yes | Yes | Yes | Yes | No | No | |
| 254 | Kong, F. *et al.* (2020)^259^ | Yes | Yes | Yes | | Yes | Yes | Yes | Yes | No | Yes | |
| 255 | Zhang, K. *et al.* (2020)^260^ | Yes | Yes | Yes | | Yes | Yes | Yes | Yes | No | Yes | |
| 256 | Damsud, T. *et al.* (2014)^261^ | Yes | Yes | Yes | | Yes | Yes | No | Yes | No | Yes | |
| 257 | Xu, Y. *et al.* (2018)^262^ | Yes | Yes | Yes | | Yes | Yes | No | Yes | No | Yes | |
| 258 | Xu, L. *et al.* (2020)^263^ | Yes | Yes | Yes | | Yes | Yes | Yes | Yes | No | Yes | |
| 259 | Tan, C. *et al.* (2015)^264^ | Yes | Yes | Yes | | Yes | Yes | Yes | Yes | No | No | |
| 260 | Ho, G. T. *et al.* (2017)^265^ | Yes | Yes | Yes | | Yes | No | Yes | Yes | No | No | |
| 261 | Toh, Z. S. *et al.* (2015)^266^ | Yes | Yes | Yes | | Yes | No | No | Yes | No | Yes | |
| 262 | Yang, K. *et al.* (2016)^267^ | Yes | Yes | Yes | | Yes | Yes | Yes | Yes | Yes | Yes | |
| 263 | Nina, N. *et al.* (2020)^268^ | Yes | Yes | Yes | | Yes | No | Yes | Yes | No | Yes | |
| 264 | Amin, A. *et al.* (2016)^269^ | Yes | Yes | Yes | | Yes | Yes | No | Yes | No | Yes | |
| 265 | Sadasivam, M. *et al.* (2018)^270^ | Yes | Yes | Yes | | Yes | No | Yes | Yes | No | Yes | |
| 266 | Kim, T. H. *et al.* (2017)^271^ | Yes | Yes | Yes | | Yes | Yes | Yes | Yes | No | Yes | |
| 267 | Jeong, G. H. *et al.* (2021)^272^ | Yes | Yes | Yes | | Yes | Yes | Yes | Yes | No | Yes | |
| 268 | Li, F. *et al.* (2013)^273^ | Yes | Yes | Yes | | Yes | Yes | Yes | Yes | No | Yes | |
| 269 | Xu, J. *et al.* (2018)^274^ | Yes | Yes | Yes | | Yes | No | Yes | Yes | No | Yes | |
| 270 | Tundis, R. *et al.* (2016)^275^ | Yes | Yes | Yes | | Yes | Yes | Yes | Yes | No | Yes | |
| 271 | Islam, Md Nurul *et al.* (2013)^276^ | Yes | Yes | Yes | | Yes | Yes | Yes | Yes | No | Yes | |
| 272 | Mohamed, E. A. *et al.* (2012)^277^ | Yes | Yes | Yes | | Yes | No | Yes | Yes | No | Yes | |
| 273 | Amin, S. *et al.* (2019)^278^ | Yes | Yes | Yes | | Yes | Yes | Yes | Yes | Yes | Yes | |
| 274 | Quan, Y. S. *et al.* (2020)^279^ | Yes | Yes | Yes | | Yes | Yes | Yes | Yes | No | Yes | |
| 275 | Kim, T. *et al.* (2014)^280^ | Yes | Yes | Yes | | Yes | No | Yes | Yes | No | Yes | |
| 276 | Arumugam, B. *et al.* (2016)^281^ | Yes | Yes | Yes | | Yes | No | Yes | Yes | No | Yes | |
| 277 | Kim, D. H. *et al.* (2017)^282^ | Yes | Yes | Yes | | Yes | Yes | Yes | Yes | No | Yes | |
| 278 | Söhretoglu D. *et al.* (2018)^283^ | Yes | Yes | Yes | | Yes | Yes | Yes | Yes | No | Yes | |
| 279 | Han, L. *et al.* (2018)^284^ | Yes | Yes | Yes | | Yes | Yes | Yes | Yes | No | Yes | |
| 280 | Lin, H. C. *et al.* (2010)^285^ | Yes | Yes | Yes | | Yes | Yes | Yes | Yes | No | Yes | |
| 281 | Sheikh, Y. *et al.* (2019)^286^ | Yes | Yes | Yes | | Yes | Yes | Yes | Yes | No | Yes | |
| 282 | Braham, Na *et al.* (2016)^287^ | Yes | Yes | Yes | | Yes | Yes | Yes | Yes | No | Yes | |
| 283 | Seong, S. H. *et al.* (2016)^288^ | Yes | Yes | Yes | | Yes | No | Yes | Yes | No | Yes | |
| 284 | Jung, H. A. *et al.* (2017)^289^ | Yes | Yes | Yes | | Yes | No | Yes | Yes | No | Yes | |
| 285 | Zhao, Bing Tian *et al.* (2016)^290^ | Yes | Yes | Yes | | Yes | Yes | Yes | Yes | No | Yes | |
| 286 | Yan, S. *et al.* (2016)^291^ | Yes | Yes | Yes | | Yes | Yes | Yes | Yes | No | Yes | |
| 287 | Zhao, Y. *et al.* (2018)^292^ | Yes | Yes | Yes | | Yes | Yes | Yes | Yes | No | Yes | |
| 288 | Gou, S. H. *et al.* (2016)^293^ | Yes | Yes | Yes | | No | No | Yes | Yes | No | Yes | |
| 289 | Yang, H. *et al.* (2016)^294^ | Yes | Yes | Yes | | Yes | Yes | Yes | Yes | No | Yes | |
| 290 | Qu, X. *et al.* (2022)^295^ | Yes | Yes | Yes | | Yes | Yes | Yes | Yes | No | Yes | |
| 291 | Wang, L. *et al.* (2018)^296^ | Yes | Yes | Yes | | Yes | Yes | Yes | Yes | No | Yes | |
| 292 | Lianwu, Xie *et al.* (2021)^297^ | Yes | Yes | Yes | | Yes | Yes | Yes | Yes | No | Yes | |
| 293 | Yang, Y. *et al.* (2015)^298^ | Yes | Yes | Yes | | Yes | Yes | Yes | Yes | No | Yes | |
| 294 | Li, Y. *et al.* (2014)^299^ | Yes | Yes | Yes | | Yes | Yes | Yes | Yes | No | Yes | |
| 295 | Tao, Y. *et al.* (2013)^300^ | Yes | Yes | Yes | | Yes | Yes | No | Yes | No | Yes | |
| 296 | Li, H. *et al.* (2009)^301^ | Yes | Yes | Yes | | Yes | Yes | Yes | Yes | No | Yes | |
| 297 | Jiang, W. *et al.* (2015)^302^ | Yes | Yes | Yes | | Yes | Yes | Yes | Yes | Yes | Yes | |
| 298 | Nguyen, M. T. T. *et al.* (2012)^303^ | Yes | Yes | Yes | | Yes | No | Yes | Yes | No | Yes | |
| 299 | Wu, B. *et al.* (2016)^304^ | Yes | Yes | Yes | | Yes | Yes | Yes | Yes | No | Yes | |
| 300 | Wang, Y. M. *et al.* (2017)^305^ | Yes | Yes | Yes | | Yes | No | No | Yes | No | Yes | |
| 301 | Yu, Zhang *et al.* (2015)^306^ | Yes | Yes | Yes | | Yes | Yes | No | Yes | No | Yes | |
| 302 | Xiao, Z. *et al.* (2022)^307^ | Yes | Yes | Yes | | Yes | Yes | Yes | Yes | No | Yes | |
| 303 | Zhang, H. *et al.* (2013)^308^ | Yes | Yes | Yes | | Yes | Yes | No | Yes | No | Yes | |
| 304 | Fan, Y. H. *et al.* (2019)^309^ | Yes | Yes | Yes | | Yes | No | No | Yes | No | Yes | |
| 305 | Ajish, K. R. *et al.*(2015)^310^ | Yes | Yes | Yes | | Yes | Yes | Yes | Yes | No | Yes | |
| 306 | Boonsombat, J. *et al.* (2017)^311^ | Yes | Yes | Yes | | Yes | Yes | Yes | Yes | Yes | Yes | |
| 307 | Sun, H. *et al.* (2018)^312^ | Yes | Yes | Yes | | Yes | No | Yes | Yes | No | Yes | |
| 308 | Hari Babu, T. *et al.* (2008)^313^ | Yes | Yes | Yes | | No | No | No | Yes | No | Yes | |
| 309 | Kumar, G. S. *et al.* (2010)^314^ | Yes | Yes | No | | Yes | No | Yes | Yes | No | Yes | |
| 310 | Cheng, N. *et al.* (2014)^315^ | Yes | Yes | Yes | | Yes | Yes | Yes | Yes | No | Yes | |
| 311 | Tang, C. *et al.* (2012)^316^ | Yes | Yes | Yes | | Yes | No | Yes | Yes | No | Yes | |
| 312 | Saleem, F. *et al.* (2021)^317^ | Yes | Yes | Yes | | Yes | Yes | Yes | Yes | No | Yes | |
| 313 | Saidi, I. *et al.* (2022)^318^ | Yes | Yes | Yes | | Yes | Yes | Yes | Yes | No | Yes | |
| 314 | Imran, S. *et al.* (2015)^319^ | Yes | Yes | Yes | | Yes | Yes | Yes | Yes | No | Yes | |
| 315 | Zhang, Y. *et al.* (2021)^320^ | Yes | Yes | Yes | | Yes | Yes | Yes | Yes | No | Yes | |
| 316 | Liu, J. *et al.* (2014)^321^ | Yes | Yes | Yes | | Yes | Yes | Yes | Yes | No | Yes | |
| 317 | Chinthala, Y. *et al.* (2015)^322^ | Yes | Yes | No | | Yes | Yes | No | Yes | No | Yes | |
| 318 | Sun, H. *et al.* (2015)^323^ | Yes | Yes | Yes | | Yes | No | Yes | Yes | No | Yes | |
| 319 | Imran, S. *et al.* (2016)^324^ | Yes | Yes | Yes | | Yes | Yes | Yes | Yes | No | Yes | |
| 320 | Mphahlele, M. *et al.* (2021)^325^ | Yes | Yes | Yes | | Yes | Yes | Yes | Yes | No | Yes | |
| 321 | Wu, C. *et al.* (2012)^326^ | Yes | Yes | Yes | | Yes | Yes | Yes | Yes | No | Yes | |
| 322 | He, X. F. *et al.* (2021)^327^ | Yes | Yes | Yes | | Yes | Yes | Yes | Yes | No | Yes | |
| 323 | Habtemariam, S. *et al.* (2012)^328^ | Yes | Yes | Yes | | Yes | No | Yes | Yes | No | Yes | |
| 324 | Taslimi, P. *et al.* (2017)^329^ | Yes | Yes | Yes | | Yes | No | No | Yes | No | Yes | |
| 325 | Zhang, L. L. *et al.* (2019)^330^ | Yes | Yes | Yes | | Yes | Yes | Yes | Yes | No | Yes | |
| 326 | Chen, Y. *et al.* (2021)^331^ | Yes | Yes | Yes | | Yes | Yes | Yes | Yes | No | Yes | |
| 327 | Mai, N. T. T. *et al.* (2012)^332^ | Yes | Yes | Yes | | Yes | No | Yes | Yes | No | Yes | |
| 328 | Yuca, H. *et al.* (2021)^333^ | Yes | Yes | Yes | | Yes | Yes | Yes | Yes | No | Yes | |
| 329 | Algethami, F. K. *et al.* (2021)^334^ | Yes | Yes | Yes | | Yes | Yes | Yes | Yes | No | Yes | |
| 330 | Srisurichan, S. *et al.* (2015)^335^ | Yes | Yes | Yes | | Yes | Yes | Yes | Yes | No | Yes | |
| 331 | Liu, Y. *et al.* (2022)^336^ | Yes | Yes | Yes | | Yes | Yes | Yes | Yes | No | Yes | |
| 332 | Fei, W. T. *et al.* (2020)^337^ | Yes | Yes | Yes | | Yes | No | Yes | Yes | No | Yes | |
| 333 | El-Hawary, S. S. *et al.* (2021)^338^ | Yes | Yes | Yes | | Yes | Yes | Yes | Yes | No | Yes | |
| 334 | Islam, M. N. *et al.* (2014)^339^ | Yes | Yes | Yes | | Yes | Yes | Yes | Yes | No | Yes | |
| 335 | Luyen, N. T. *et al.* (2019)^340^ | Yes | Yes | Yes | | Yes | Yes | Yes | Yes | No | Yes | |
| 336 | Liu, M. *et al.* (2014)^341^ | Yes | Yes | Yes | | Yes | Yes | Yes | Yes | Yes | Yes | |
| 337 | Tan, C. *et al.* (2013)^342^ | Yes | Yes | Yes | | Yes | Yes | Yes | Yes | No | Yes | |
| 338 | Gong, T. *et al.* (2020)^343^ | Yes | Yes | Yes | | Yes | Yes | Yes | Yes | No | Yes | |
| 339 | Carina Proença, C. *et al.* (2019)^344^ | Yes | Yes | Yes | | Yes | No | Yes | Yes | No | Yes | |

# Table S6. *In vitro* α-glucosidase and α-amylase inhibitory effects of retrieved flavan and flavanol derivatives.

| **No** | **Name** | **α-glucosidase** | **α-amylase** |
| --- | --- | --- | --- |
| **1** | (2*S*)-4′-Hydroxy-5,7-dimethoxy-8-methylflavan | 1 study ^226^  pIC_50 flavonoid_: 3.43  pIC_50_ _acarbose_: 2.39  Mechanism: mixed ^226^ | 1 study ^226^  pIC_50 flavonoid_: 2.21  pIC_50_ _acarbose_: 4.98  Mechanism: mixed ^226^ |
| **2** | (+) Catechin | 18 studies ^11,22,69,109,116,123,128,136,152,223,227,235,258,274,280,321,327,330^  pIC_50 flavonoid_: 3.94 (3.42 – 4.28)  pIC_50_ _acarbose_: 3.61 (3.21 – 4.41)  Mechanism: competitive ^330^ | 6 studies ^44,116,128,141,235,321^  pIC_50 flavonoid_: 3.31 (2.67 - 4.61)  pIC_50_ _acarbose_: 3.55 (3.15 - 4.24) |
| **3** | (-) Catechin | 1 study ^127^  pIC_50 flavonoid_: 4.90  pIC_50_ _acarbose_: 8.04 | 1 study ^127^  pIC_50 flavonoid_: 5.12  pIC_50_ _acarbose_: 5.61 |
| **4** | (-) Epicatechin | 14 studies ^69,79,87,128,136,154,166,175,223,296,308,327,330,343^  pIC_50 flavonoid_: 4.47 (3.52 - 4.69)  pIC_50_ _acarbose_: 4.05 (3.5 - 5.12)  Mechanism: competitive,^330^ mixed ^343^ | 3 studies ^79,128,141^  pIC_50 flavonoid_: 4.50 (3.54 - 4.76)  pIC_50_ _acarbose_: 3.95 (3.55 - 4.89) |
| **5** | (-) Epigallocatechin | 2 studies ^136,143^  pIC_50 flavonoid_: 5.27 (4.78 - 5.64)  pIC_50_ _acarbose_: 5.74 (5.61 - 5.88) | 1 study ^249^  pIC_50 flavonoid_: 3.52  pIC_50_ _acarbose_: 3.91 |
| **6** | (-) ECG | 4 studies ^143,211,218,248^  pIC_50 flavonoid_: 5.27 (4.78 – 5.64)  pIC_50_ _acarbose_: 3.91 (3.84 – 4.44)  Mechanism: non-competitive ^218^ | 2 studies ^218,331^  pIC_50 flavonoid_: 3.70 (3.55 - 3.84)  pIC_50_ _acarbose_: 4.27 (4.22 - 4.32)  Mechanism: mixed ^218,331^ |
| **7** | (-) EGCG | 12 studies ^87,109,127,139,143,150,204,212,218,248,272,294^  pIC_50 flavonoid_: 4.66 (4.01 - 5.12)  pIC_50_ _acarbose_: 4.42 (3.62 - 6.02)  Mechanism: non-competitive ^212^ | 6 studies ^127,131,238,272,331^  pIC_50 flavonoid_: 3.82 (3.26 - 4.29)  pIC_50_ _acarbose_: 4.27 (4.16 – 4.93)  Mechanism: competitive,^131^  non-competitive^331^ |
| **8** | Theaflavin | 1 study ^127^  pIC_50 flavonoid_: 4.79  pIC_50_ _acarbose_: 8.03 | 1 study ^127^  pIC_50 flavonoid_: 5.12  pIC_50_ _acarbose_: 6.34 |

# Table S7. *In vitro* α-glucosidase and α-amylase inhibitory effects of retrieved flavanone derivatives.

| **No** | **Name** | **α-glucosidase** | **α-amylase** |
| --- | --- | --- | --- |
| **9** | Naringenin | 16 studies ^13,65,79,93,128,139,148,165,190,243,244,247,256,260,274,290^  pIC_50 flavonoid_: 4.35 (3.78 - 4.77)  pIC_50_ _acarbose_: 3.82 (3.5 - 4.61)  Mechanism: competitive ^243^ | 3 studies ^79,93,128^  pIC_50 flavonoid_: 4.66 (4.29 - 4.93)  pIC_50_ _acarbose_: 3.95 (3.57 - 4.89) |
| **10** | 8-Geranylnaringenin | 1 study ^244^  pIC_50 flavonoid_: 5.42  pIC_50_ _acarbose_: 4.29 | 1 study ^244^  pIC_50 flavonoid_: 4.81  pIC_50_ _acarbose_: 5.66 |
| **11** | Eriodictyol | 4 studies ^10,18,109,328^  pIC_50 flavonoid_: 4.36 (3.93 - 4.53)  pIC_50_ _acarbose_: 4.09 (3.71 - 4.91) | 1 study ^207^  pIC_50 flavonoid_: 3.50  pIC_50_ _acarbose_: 3.14 |
| **12** | Propolin D  (Nymphaeol B) | 1 study ^93^  pIC_50 flavonoid_: 3.60  pIC_50_ _acarbose_: 3.40 | 1 study ^93^  pIC_50 flavonoid_: 3.69  pIC_50_ _acarbose_: 3.20 |
| **13** | Propolin H  (3’-Geranylnaringenin) | 1 study ^93^  pIC_50 flavonoid_: 3.75  pIC_50_ _acarbose_: 3.40 | 1 study ^93^  pIC_50 flavonoid_: 4.28  pIC_50_ _acarbose_: 3.20 |
| **14** | Propolin F  (Isonymphaeol B) | 1 study ^93^  pIC_50 flavonoid_: 3.53  pIC_50_ _acarbose_: 3.40 | 1 study ^93^  pIC_50 flavonoid_: 3.87  pIC_50_ _acarbose_: 3.20 |
| **15** | Propolin C  (Nymphaeol A) | 1 study ^93^  pIC_50 flavonoid_: 3.38  pIC_50_ _acarbose_: 3.40 | 1 study ^93^  pIC_50 flavonoid_: 3.69  pIC_50_ _acarbose_: 3.20 |
| **16** | Propolin G  (Nymphaeol C) | 1 study ^93^  pIC_50 flavonoid_: 3.11  pIC_50_ _acarbose_: 3.40 | 1 study ^93^  pIC_50 flavonoid_: 3.61  pIC_50_ _acarbose_: 3.20 |
| **17** | Isocarthamidin-7-*O*-glucuronide | 1 study ^214^  pIC_50 flavonoid_: 2.33  pIC_50_ _acarbose_: 2.81 | 1 study ^214^  pIC_50 flavonoid_: 2.20  pIC_50_ _acarbose_: 2.81 |
| **18** | Naringin | 6 studies ^107,259,260,274,275,329^  pIC_50 flavonoid_: 4.57 (2.28 - 5.94)  pIC_50_ _acarbose_: 4.11 (3.09 - 4.55) | 2 studies ^107,275^  pIC_50 flavonoid_: 4.77 (4.6 - 4.93)  pIC_50_ _acarbose_: 4.24 (4.17 - 4.3)  Mechanism: competitive ^107^ |
| **19** | Narirutin | 2 studies ^106,275^  pIC_50 flavonoid_: 5.32 (5.08 - 5.56)  pIC_50_ _acarbose_: 5.26 (4.76 - 5.76) | 2 studies ^106,275^  pIC_50 flavonoid_: 4.55 (4.35 - 4.75)  pIC_50_ _acarbose_: 3.46 (3.13 - 3.78) |
| **20** | Poncirin | 2 studies ^107,275^  pIC_50 flavonoid_: 4.05 (3.97 - 4.12)  pIC_50_ _acarbose_: 4.11 (4.04 - 4.19) | 2 studies ^107,275^  pIC_50 flavonoid_: 5.20 (4.81 - 5.6)  pIC_50_ _acarbose_: 4.24 (4.17 - 4.3) |
| **21** | Hesperidin | 4 studies ^107,190,275,329^  pIC_50 flavonoid_: 4.69 (4.42 - 5.56)  pIC_50_ _acarbose_: 4.34 (4.19 - 4.47) | 2 studies ^107,275^  pIC_50 flavonoid_: 4.9 (4.74 - 5.06)  pIC_50_ _acarbose_: 4.24 (4.17 - 4.3) |
| **22** | Kuwanon L | 1 study ^292^  pIC_50 flavonoid_: 4.96  pIC_50_ _acarbose_: 3.53 | 1 study ^292^  pIC_50 flavonoid_: 4.57  pIC_50_ _acarbose_: 5.82 |
| **23** | Sanggenon G | 1 study ^292^  pIC_50 flavonoid_: 5.53  pIC_50_ _acarbose_: 3.53 | 1 study ^292^  pIC_50 flavonoid_: 5.12  pIC_50_ _acarbose_: 5.82 |

# Table S8. *In vitro* α-glucosidase and α-amylase inhibitory effects of retrieved flavanonol derivatives.

| **No** | **Name** | **α-glucosidase** | **α-amylase** |
| --- | --- | --- | --- |
| **24** | Taxifolin | 6 studies ^61,80,86,109,193,194^  pIC_50 flavonoid_: 3.35 (2.82 - 3.85)  pIC_50_ _acarbose_: 4.01 (3.71 - 5.07)  Mechanism: competitive ^194^ | 2 studies ^86,194^  pIC_50 flavonoid_: 3.40 (2.84 - 3.95)  pIC_50_ _acarbose_: 3.89 (3.79 – 4.00)  Mechanism: competitive ^194^ |
| **25** | Silibinin | 1 study ^200^  pIC_50 flavonoid_: 4.94  pIC_50_ _acarbose_: 3.71  Mechanism: non-competitive ^200^ | 1 study ^200^  pIC_50 flavonoid_: 4.23  pIC_50_ _acarbose_: 5.70  Mechanism: non-competitive ^200^ |
| **26** | Dysosmaflavanone | 1 study ^7^  pIC_50 flavonoid_: 2.86  pIC_50_ _acarbose_: 2.99 | 1 study ^7^  pIC_50 flavonoid_: 2.88  pIC_50_ _acarbose_: 3.20 |

# Table S9. *In vitro* α-glucosidase and α-amylase inhibitory effects of retrieved flavone derivatives.

| **No** | **Name** | **α-glucosidase** | **α-amylase** |
| --- | --- | --- | --- |
| **27** | Chrysin | 8 studies ^120,184,214,250,313-315,329^  pIC_50 flavonoid_: 4.14 (3.7 - 5.34)  pIC_50_ _acarbose_: 4.2 (3.62 - 4.85) | 1 study ^214^  pIC_50 flavonoid_: 2.75  pIC_50_ _acarbose_: 2.98 |
| **28** | Apigenin | 30 studies ^12,14,27,40,67,79,87,91,92,109,121,126,138,145,148,152,165,170,187,189,214,240,256,279,297,301,304,305,315,340^  pIC_50 flavonoid_: 4.14 (3.88 - 4.58)  pIC_50_ _acarbose_: 3.57 (3.31 - 3.97) | 7 studies ^12,75,79,123,214,240^  pIC_50 flavonoid_: 3.91 (3.4 - 4.39)  pIC_50_ _acarbose_: 5.28 (4.3 - 5.86)  Mechanism: competitive,^75^  non-competitive^240^ |
| **29** | 5-Deoxyluteolin | 3 studies ^42,79,170^  pIC_50 flavonoid_: 4.02 (3.35 - 4.31)  pIC_50_ _acarbose_: 3.72 (3.47 - 4.72) | 1 study ^79^  pIC_50 flavonoid_: 4.79  pIC_50_ _acarbose_: 5.83 |
| **30** | Luteolin | 31 studies ^10,12,27,42,54,62,67,79,91,92,101,103,109,121,123,138,139,145,148,162,170,171,189,192,229,236,237,297,298,301,315^  pIC_50 flavonoid_: 4.22 (3.90 - 4.67)  pIC_50_ _acarbose_: 3.70 (3.30 - 5.59)  Mechanism: competitive,^103^  non-competitive ^62,171,237^ | 6 studies ^79,151,206,307^  pIC_50 flavonoid_: 4.16 (3.27 – 4.60)  pIC_50_ _acarbose_: 5.53 (5.02 - 5.87)  Mechanism: non-competitive ^206^ |
| **31** | Chrysoeriol | 3 studies ^13,36,170^  pIC_50 flavonoid_: 4.19 (3.97 - 4.22)  pIC_50_ _acarbose_: 3.48 (3.35 - 3.57) | 2 studies ^81,344^  pIC_50 flavonoid_: 3.31 (3.10 - 3.51)  pIC_50_ _acarbose_: 5.10 (4.7 - 5.49) |
| **32** | Tricetin | 1 study ^12^  pIC_50 flavonoid_: 2.11  pIC_50_ _acarbose_: 2.21 | 1 study ^12^  pIC_50 flavonoid_: 2.85  pIC_50_ _acarbose_: 4.24 |
| **33** | Baicalein | 10 studies ^32,101,109,120,123,139,170,214,313,314^  pIC_50 flavonoid_: 4.21 (3.78 - 4.4)  pIC_50_ _acarbose_: 5.33 (3.71 - 5.85)  Mechanism: mixed ^139^ | 2 studies ^123,214^  pIC_50 flavonoid_: 3.13 (3.02 - 3.24)  pIC_50_ _acarbose_: 4.13 (3.55 - 4.7) |
| **34** | Hispidulin | 2 studies ^297,303^  pIC_50 flavonoid_: 4.73 (4.52 - 4.94)  pIC_50_ _acarbose_: 3.57 (3.54 - 3.59) | 1 study ^240^  pIC_50 flavonoid_: 4.52  pIC_50_ _acarbose_: 5.94  Mechanism: competitive ^240^ |
| **35** | Sinensetin | 1 study ^277^  pIC_50 flavonoid_: 2.75  pIC_50_ _acarbose_: 2.52 | 1 study ^277^  pIC_50 flavonoid_: 2.52  pIC_50_ _acarbose_: 2.12 |
| **36** | Eupatorin | 1 study ^49^  pIC_50 flavonoid_: 6.44  pIC_50_ _acarbose_: 4.64 | 1 study ^49^  pIC_50 flavonoid_: 6.76  pIC_50_ _acarbose_: 5.00 |
| **37** | Eupatillin | 1 study ^49^  pIC_50 flavonoid_: 6.49  pIC_50_ _acarbose_: 4.64 | 1 study ^49^  pIC_50 flavonoid_: 6.61  pIC_50_ _acarbose_: 5.00 |
| **38** | 5,7,3′,5′-Tetrahydroxy-6,4′-dimethoxyflavone | 1 study ^222^  pIC_50 flavonoid_: 4.23  pIC_50_ _acarbose_: 3.18 | 1 study ^222^  pIC_50 flavonoid_: 3.58  pIC_50_ _acarbose_: 5.23 |
| **39** | 5,3′,5′-Trihydroxy-6,7,4′-trimethoxyflavone | 1 study ^222^  pIC_50 flavonoid_: 4.11  pIC_50_ _acarbose_: 3.18 | 1 study ^222^  pIC_50 flavonoid_: 3.54  pIC_50_ _acarbose_: 5.23 |
| **40** | 5,7,5′-Trihydroxy-6,3′,4′-trimethoxy-flavone | 1 study ^222^  pIC_50 flavonoid_: 4.31  pIC_50_ _acarbose_: 3.18 | 1 study ^222^  pIC_50 flavonoid_: 3.92  pIC_50_ _acarbose_: 5.23 |
| **41** | 5-Hydroxy-6,7,3′,4′,5′-pentamethoxyflavone | 1 study ^222^  pIC_50 flavonoid_: 4.11  pIC_50_ _acarbose_: 3.18 | 1 study ^222^  pIC_50 flavonoid_: 3.41  pIC_50_ _acarbose_: 5.23 |
| **42** | Tangeretin | 2 studies ^58,107^  pIC_50 flavonoid_: 5.2 (4.52 - 5.87)  pIC_50_ _acarbose_: 4.76 (4.36 - 5.16) | 3 studies ^58,107,270^  pIC_50 flavonoid_: 4.83 (4.43 - 5.5)  pIC_50_ _acarbose_: 4.6 (4.48 - 4.8) |
| **43** | Nobiletin | 1 study ^107^  pIC_50 flavonoid_: 5.38  pIC_50_ _acarbose_: 3.97 | 2 studies ^107,270^  pIC_50 flavonoid_: 4.22 (4.18 - 4.26)  pIC_50_ _acarbose_: 4.48 (4.42 - 4.54) |
| **44** | Gardenin A | 1 study ^49^  pIC_50 flavonoid_: 6.43  pIC_50_ _acarbose_: 4.64 | 1 study ^49^  pIC_50 flavonoid_: 6.62  pIC_50_ _acarbose_: 5.00 |
| **45** | Kuwanon T | 1 study ^292^  pIC_50 flavonoid_: 4.98  pIC_50_ _acarbose_: 3.53 | 1 study ^292^  pIC_50 flavonoid_: 4.75  pIC_50_ _acarbose_: 5.82 |
| **46** | Kuwanon C | 3 studies ^88,201,292^  pIC_50 flavonoid_: 4.83 (4.7 - 4.88)  pIC_50_ _acarbose_: 3.53 (3.49 - 3.61)  Mechanism: mixed^201^ | 1 study ^292^  pIC_50 flavonoid_: 4.16  pIC_50_ _acarbose_: 5.82 |
| **47** | Morusin | 1 study ^292^  pIC_50 flavonoid_: 4.69  pIC_50_ _acarbose_: 3.53 | 1 study ^292^  pIC_50 flavonoid_: 4.76  pIC_50_ _acarbose_: 5.82 |
| **48** | Morusinol | 2 studies ^88,292^  pIC_50 flavonoid_: 4.35 (4.17 - 4.53)  pIC_50_ _acarbose_: 3.61 (3.57 - 3.65) | 1 study ^292^  pIC_50 flavonoid_: 4.29  pIC_50_ _acarbose_: 5.82 |
| **49** | Kuwanon G (Moracenin B) | 2 studies ^201,292^  pIC_50 flavonoid_: 5.70 (5.63 - 5.77)  pIC_50_ _acarbose_: 3.49 (3.47 - 3.51)  Mechanism: mixed ^201^ | 1 study ^292^  pIC_50 flavonoid_: 5.55  pIC_50_ _acarbose_: 5.82 |
| **50** | Moracenin A | 1 study ^292^  pIC_50 flavonoid_: 6.09  pIC_50_ _acarbose_: 3.53 | 1 study ^292^  pIC_50 flavonoid_: 5.71  pIC_50_ _acarbose_: 5.82 |
| **51** | 8-(7-Methoxycoumarin-6-yl)luteolin | 1 study ^312^  pIC_50 flavonoid_: 5.83  pIC_50_ _acarbose_: 3.65  Mechanism: mixed ^312^ | 1 study ^312^  pIC_50 flavonoid_: 5.16  pIC_50_ _acarbose_: 5.57 |
| **52** | Chrysin-7-*O*-β-D-glucuronide | 1 study ^214^  pIC_50 flavonoid_: 2.85  pIC_50_ _acarbose_: 2.81 | 1 study ^214^  pIC_50 flavonoid_: 2.64  pIC_50_ _acarbose_: 2.98 |
| **53** | Apigenin-7-*O*-β-D- glucuronide | 2 studies ^79,214^  pIC_50 flavonoid_: 3.89 (3.4 - 4.38)  pIC_50_ _acarbose_: 4.26 (3.54 - 4.99) | 2 studies ^79,214^  pIC_50 flavonoid_: 3.91 (3.37 - 4.45)  pIC_50_ _acarbose_: 4.40 (3.69 - 5.11) |
| **54** | Acacetin-7-*O*-β-D-glucoside | 1 study ^207^  pIC_50 flavonoid_: 3.35  pIC_50_ _acarbose_: 2.72 | 1 study ^207^  pIC_50 flavonoid_: 3.47  pIC_50_ _acarbose_: 3.14 |
| **55** | Luteolin-7-*O*-β-D-glucuronide | 1 study ^153^  pIC_50 flavonoid_: 4.83  pIC_50_ _acarbose_: 4.79 | 1 study ^153^  pIC_50 flavonoid_: 4.21  pIC_50_ _acarbose_: 4.27 |
| **56** | Luteolin-7-*O*-β-D-glucoside | 5 studies ^62,109,121,153,297^  pIC_50 flavonoid_: 4.23 (3.37 - 4.51)  pIC_50_ _acarbose_: 3.79 (3.52 - 4.79)  Mechanism: non-competitive^62^ | 1 study ^153^  pIC_50 flavonoid_: 4.09  pIC_50_ _acarbose_: 4.27 |
| **57** | Diosmin | 1 study ^137^  pIC_50 flavonoid_: 3.17  pIC_50_ _acarbose_: 3.18 | 1 study ^137^  pIC_50 flavonoid_: 3.18  pIC_50_ _acarbose_: 3.14 |
| **58** | 8-Hydroxyapigenin-7-*O*-β-D-glucoside | 1 study ^50^  pIC_50 flavonoid_: 3.89  pIC_50_ _acarbose_: 4.33 | 1 study ^50^  pIC_50 flavonoid_: 3.94  pIC_50_ _acarbose_: 4.35 |
| **59** | Baicalin | 3 studies ^101,120,214^  pIC_50 flavonoid_: 4.01 (3.44 - 4.22)  pIC_50_ _acarbose_: 5.46 (4.13 - 5.67) | 1 study ^214^  pIC_50 flavonoid_: 2.83  pIC_50_ _acarbose_: 2.98 |
| **60** | Scutellarin | 1 study ^214^  pIC_50 flavonoid_: 3.17  pIC_50_ _acarbose_: 2.81 | 1 study ^214^  pIC_50 flavonoid_: 3.10  pIC_50_ _acarbose_: 2.98 |
| **61** | Swertisin | 1 study ^326^  pIC_50 flavonoid_: 3.57  pIC_50_ _acarbose_: 3.47 | 1 study ^213^  pIC_50 flavonoid_: 2.37  pIC_50_ _acarbose_: 3.67  Mechanism: competitive^213^ |
| **62** | 3,8-Dichloro-2-(3,4-dihydroxyphenyl)-5,7-dihydroxy-4*H*-chromen-4-one | 1 study ^170^  pIC_50 flavonoid_: 4.37  pIC_50_ _acarbose_: 3.22 | 1 study ^344^  pIC_50 flavonoid_: 4.09  pIC_50_ _acarbose_: 5.89 |
| **63** | 3-Chloro-2-(3,4-dihydroxyphenyl)-5,7-dihydroxy-4*H*-chromen-4-one | 1 study ^170^  pIC_50 flavonoid_: 4.68  pIC_50_ _acarbose_: 3.22  Mechanism: non-competitive^170^ | 1 study ^344^  pIC_50 flavonoid_: 4.36  pIC_50_ _acarbose_: 5.89  Mechanism: competitive^344^ |
| **64** | 6,8-Dichloro-2-(3,4-dihydroxyphenyl)-5,7-dihydroxy-4*H*-chromen-4-one | 1 study ^170^  pIC_50 flavonoid_: 4.47  pIC_50_ _acarbose_: 3.22 | 1 study ^344^  pIC_50 flavonoid_: 4.01  pIC_50_ _acarbose_: 5.89 |
| **65** | 8-Chloro-2-(3,4-dihydroxyphenyl)-5,7-dihydroxy-4*H*-chromen-4-one | 1 study ^170^  pIC_50 flavonoid_: 4.26  pIC_50_ _acarbose_: 3.22 | 1 study ^344^  pIC_50 flavonoid_: 3.76  pIC_50_ _acarbose_: 5.89 |
| **66** | 2-(3,4-Dihydroxyphenyl)-5-hydroxy-4*H*-chromen-4-one | 1 study ^170^  pIC_50 flavonoid_: 4.02  pIC_50_ _acarbose_: 3.22 | 1 study ^344^  pIC_50 flavonoid_: 3.88  pIC_50_ _acarbose_: 5.89 |
| **67** | 2-(3,4-Dihydroxyphenyl)-5-hydroxy-4*H*-chromen-4-one | 1 study ^170^  pIC_50 flavonoid_: 4.18  pIC_50_ _acarbose_: 3.22 | 1 study ^344^  pIC_50 flavonoid_: 3.83  pIC_50_ _acarbose_: 5.89  Mechanism: competitive ^344^ |

# Table S10. *In vitro* α-glucosidase and α-amylase inhibitory effects of retrieved flavonol derivatives.

| **No** | **Name** | **α-glucosidase** | **α-amylase** |
| --- | --- | --- | --- |
| **68** | Quercetin | 67 studies ^13,14,17,28,29,34,42,48,61,63,79,80,84,87,93,95,99,108-110,112,118,123,125,128,129,136,139,148-150,152,154,155,157,160,162,165,168,170,173,175,176,186,187,195,198,202,229,241,248,253,257,264,271,274,276,279,281,295,296,304,309,323,336,338,342^  pIC_50 flavonoid_: 4.54 (3.87 - 4.96)  pIC_50_ _acarbose_: 3.67 (3.34 - 4.36)  Mechanism: mixed,^108,129,173^  non-competitive,^168,295^  competitive ^170,229,241^ | 18 studies ^25,29,44,79,111,128,129,131,132,151,155,195,202,206,281,307^  pIC_50 flavonoid_: 4.07 (3.26 - 4.83)  pIC_50_ _acarbose_: 4.89 (4.03 - 5.53)  Mechanism: non-competitive,^206^ competitive ^129,131^ |
| **69** | Kaempferol | 34 studies ^7,14,18,28,29,51,61,63,67,79,84,92,112,118,125,128,139,146,148,152,160,170,173,198,202,216,236,247,274,279,304,310,336^  pIC_50 flavonoid_: 4.24 (3.98 - 4.71)  pIC_50_ _acarbose_: 3.72 (3.26 - 4.46)  Mechanism: mixed ^173^ | 9 studies ^7,29,84,94,128,202,206,307^  pIC_50 flavonoid:_ 3.93 (2.9 - 4.92)  pIC_50_ _acarbose_: 5.25 (3.95 - 5.83)  Mechanism: non-competitive ^206^ |
| **70** | Myricetin | 19 studies ^13,17,29,61,82,109,118,139,152,155,169,173,217,241,274,281,304,306,336^  pIC_50 flavonoid_: 4.48 (3.75 - 4.97)  pIC_50_ _acarbose_: 3.67 (3.43 - 4.39)  Mechanism: competitive,^139,241^  mixed ^173^ | 5 studies ^29,151,155,281^  pIC_50 flavonoid_: 3.97 (3.33 - 4.52)  pIC_50_ _acarbose_: 5.46 (4.72 - 5.89)  Mechanism: competitive ^345^ |
| **71** | Fisetin | 3 studies ^66,109,336^  pIC_50 flavonoid_: 4.33 (4.20 - 4.41)  pIC_50_ _acarbose_: 3.43 (3.11 - 4.83)  Mechanism: mixed ^66^ | 1 study ^151^  pIC_50 flavonoid_: 4.71  pIC_50_ _acarbose_: 6.00 |
| **72** | 3,7,4'-Trihydroxyflavone | 1 study ^170^  pIC_50 flavonoid_: 4.02  pIC_50_ _acarbose_: 3.22 | 1 study ^344^  pIC_50 flavonoid_: 4.23  pIC_50_ _acarbose_: 5.89  Mechanism: Competitive ^344^ |
| **73** | Quercetagetin | 2 studies ^150,195^  pIC_50 flavonoid_: 4.20 (3.97 - 4.43)  pIC_50_ _acarbose_: 4.44 (3.76 - 5.11)  Mechanism: non-competitive ^195^ | 3 studies ^151,195,238^  pIC_50 flavonoid_: 4.52 (4.19 - 4.76)  pIC_50_ _acarbose_: 5.68 (5.46 - 5.84)  Mechanism: non-competitive ^195^ |
| **74** | Isorhamnetin | 9 studies ^34,63,109,112,128,264,276,304,336^  pIC_50 flavonoid_: 3.85 (3.50 - 4.35)  pIC_50_ _acarbose_: 3.59 (3.43 - 3.88)  Mechanism: mixed ^34^ | 2 studies ^111,128^  pIC_50 flavonoid_: 5.10 (5.09 - 5.11)  pIC_50_ _acarbose_: 4.39 (4.17 - 4.61) |
| **75** | Kaempferide | 5 studies ^79,139,152,279,290^  pIC_50 flavonoid_: 4.32 (3.93 - 4.69)  pIC_50_ _acarbose_: 3.75 (3.37 - 4.42) | 1 study ^79^  pIC_50 flavonoid_: 4.64  pIC_50_ _acarbose_: 5.83 |
| **76** | Quercetin-3-methylether | 5 studies ^79,162,186,192,309^  pIC_50 flavonoid_: 4.11 (4.08 - 4.69)  pIC_50_ _acarbose_: 5.71 (3.95 - 6.81) | 1 study ^79^  pIC_50 flavonoid_: 4.87  pIC_50_ _acarbose_: 5.83 |
| **77** | Podoverine A | 1 study ^7^  pIC_50 flavonoid_: 2.71  pIC_50_ _acarbose_: 2.99 | 1 study ^7^  pIC_50 flavonoid_: 2.89  pIC_50_ _acarbose_: 3.20 |
| **78** | 8,2'-Diprenylquercetin 3-methyl ether | 1 study ^7^  pIC_50 flavonoid_: 2.98  pIC_50_ _acarbose_: 2.99 | 1 study ^7^  pIC_50 flavonoid_: 3.07  pIC_50_ _acarbose_: 3.20 |
| **79** | Solophenol D | 1 study ^93^  pIC_50 flavonoid_: 4.24  pIC_50_ _acarbose_: 3.40 | 1 study ^93^  pIC_50 flavonoid_: 3.85  pIC_50_ _acarbose_: 3.20 |
| **80** | 5,7-Dibromo-2-(2,4-dimethylphenyl)-3-hydroxy-4*H*-chromen-4-one | 1 study ^119^  pIC_50 flavonoid_: 3.59  pIC_50_ _acarbose_: 4.12 | 1 study ^119^  pIC_50 flavonoid_: 5.12  pIC_50_ _acarbose_: 5.30 |
| **81** | 5,7-Dibromo-3-hydroxy-2-(4-methylphenyl)-4*H*-chromen-4-one | 1 study ^119^  pIC_50 flavonoid_: 3.82  pIC_50_ _acarbose_: 4.12 | 1 study ^119^  pIC_50 flavonoid_: 3.78  pIC_50_ _acarbose_: 5.30 |
| **82** | 5,7-Dibromo-2-[4-(dimethylamino)phenyl]-3-hydroxy-4*H*-chromen-4-one | 1 study ^119^  pIC_50 flavonoid_: 4.14  pIC_50_ _acarbose_: 4.12 | 1 study ^119^  pIC_50 flavonoid_: 3.93  pIC_50_ _acarbose_: 5.30 |
| **83** | 5,7-Dibromo-3-hydroxy-2-(4-methoxyphenyl)-4*H*-chromen-4-one | 1 study ^119^  pIC_50 flavonoid_: 3.67  pIC_50_ _acarbose_: 4.12 | 1 study ^119^  pIC_50 flavonoid_: 4.40  pIC_50_ _acarbose_: 5.30 |
| **84** | 5,7-Dibromo-2-(3,4-dimethoxyphenyl)-3-hydroxy-4*H*-chromen-4-one | 1 study ^119^  pIC_50 flavonoid_: 3.58  pIC_50_ _acarbose_: 4.12 | 1 study ^119^  pIC_50 flavonoid_: 5.23  pIC_50_ _acarbose_: 5.30 |
| **85** | 4-(5,7-Dibromo-3-hydroxy-4-oxo-4*H*-chromen-2-yl) benzoic acid | 1 study ^119^  pIC_50 flavonoid_: 3.52  pIC_50_ _acarbose_: 4.12 | 1 study ^119^  pIC_50 flavonoid_: 4.82  pIC_50_ _acarbose_: 5.30 |
| **86** | 5,7-Dibromo-3-hydroxy-2-(4-nitrophenyl)-4*H*-chromen-4-one | 1 study ^119^  pIC_50 flavonoid_: 4.10  pIC_50_ _acarbose_: 4.12 | 1 study ^119^  pIC_50 flavonoid_: 3.84  pIC_50_ _acarbose_: 5.30 |
| **87** | 5,7-Dibromo-3-hydroxy-2-[4-(trifluoromethyl)phenyl]-4*H*-chromen-4-one | 1 study ^119^  pIC_50 flavonoid_: 3.96  pIC_50_ _acarbose_: 4.12 | 1 study ^119^  pIC_50 flavonoid_: 4.54  pIC_50_ _acarbose_: 5.30 |
| **88** | 5,7-Dibromo-2-(4-chlorophenyl)-3-hydroxy-4*H*-chromen-4-one ( | 1 study ^119^  pIC_50 flavonoid_: 3.53  pIC_50_ _acarbose_: 4.12 | 1 study ^119^  pIC_50 flavonoid_: 3.88  pIC_50_ _acarbose_: 5.30 |
| **89** | 5,7-Dibromo-2-(4-fluorophenyl)-3-hydroxy-4*H*-chromen-4-one | 1 study ^119^  pIC_50 flavonoid_: 3.49  pIC_50_ _acarbose_: 4.12 | 1 study ^119^  pIC_50 flavonoid_: 3.65  pIC_50_ _acarbose_: 5.30 |
| **90** | 5,7-Dibromo-3-hydroxy-2-(4-morpholinophenyl)-4*H*-chromen-4-one | 1 study ^119^  pIC_50 flavonoid_: 3.92  pIC_50_ _acarbose_: 4.12 | 1 study ^119^  pIC_50 flavonoid_: 4.56  pIC_50_ _acarbose_: 5.30 |
| **91** | 5,7-Dibromo-3-hydroxy-2-(3-nitrophenyl)-4*H*-chromen-4-one | 1 study ^119^  pIC_50 flavonoid_: 3.66  pIC_50_ _acarbose_: 4.12 | 1 study ^119^  pIC_50 flavonoid_: 5.17  pIC_50_ _acarbose_: 5.30 |
| **92** | 5,7-Dibromo-3-hydroxy-2-(naphthalen-1-yl)-4*H*-chromen-4-one | 1 study ^119^  pIC_50 flavonoid_: 3.55  pIC_50_ _acarbose_: 4.12 | 1 study ^119^  pIC_50 flavonoid_: 3.58  pIC_50_ _acarbose_: 5.30 |
| **93** | 5,7-Dibromo-3-hydroxy-2-[1-[(4-methylphenyl)sulfonyl]-1*H*-indol-3-yl]-4*H*-chromen-4-one | 1 study ^119^  pIC_50 flavonoid_: 4.15  pIC_50_ _acarbose_: 4.12 | 1 study ^119^  pIC_50 flavonoid_: 5.31  pIC_50_ _acarbose_: 5.30 |
| **94** | 5,7-Dibromo-3-hydroxy-2-(1*H*-indol-3-yl)-4*H*-chromen-4-one | 1 study ^119^  pIC_50 flavonoid_: 4.14  pIC_50_ _acarbose_: 4.12 | 1 study ^119^  pIC_50 flavonoid_: 5.30  pIC_50_ _acarbose_: 5.30 |
| **95** | 5,7-Dibromo-2-(furan-2-yl)-3-hydroxy-4*H*-chromen-4-one | 1 study ^119^  pIC_50 flavonoid_: 3.82  pIC_50_ _acarbose_: 4.12 | 1 study ^119^  pIC_50 flavonoid_: 4.65  pIC_50_ _acarbose_: 5.30 |
| **96** | 5,7-Dibromo-3-hydroxy-2-(thiophen-2-yl)-4*H*-chromen-4-one | 1 study ^119^  pIC_50 flavonoid_: 3.58  pIC_50_ _acarbose_: 4.12 | 1 study ^119^  pIC_50 flavonoid_: 4.09  pIC_50_ _acarbose_: 5.30 |
| **97** | Isoquercetrin (Isoquercetin; Quercetin-3-β-D-glucoside) | 25 studies ^36,48,68,73,80,108,128,156,160,175,185,188,190,191,253,257,264,274,283,287,295,296,300,304,336^  pIC_50 flavonoid_: 3.78 (3.34 - 4.27)  pIC_50_ _acarbose_: 3.70 (3.33 - 3.92)  Mechanism: mixed,^108^  non-competitive,^283^ competitive ^295^ | 5 studies ^25,111,128,188,230^  pIC_50 flavonoid_: 4.48 (3.49 - 4.51)  pIC_50_ _acarbose_: 3.63 (3.62 - 3.95) |
| **98** | Hyperoside (Hyperin; Quercetin-3-β-D-galactoside) | 12 studies ^11,29,64,68,148,175,202,274,276,291,296,336^  pIC_50 flavonoid_: 3.90 (3.28 - 4.22)  pIC_50_ _acarbose_: 3.50 (3.28 - 3.78) | 3 studies ^25,29,202^  pIC_50 flavonoid_: 3.50 (2.86 - 4.89)  pIC_50_ _acarbose_: 3.62 (3.13 - 4.59) |
| **99** | Quercitrin  (Quercetin-3-rhamnoside) | 21 studies ^13,26,42,74,80,82,100,128,154,160,183,190,203,253,257,274,291,296,298,304,336^  pIC_50 flavonoid_: 3.96 (3.6 - 4.41)  pIC_50_ _acarbose_: 3.72 (3.53 - 4.04)  Mechanism: mixed ^74,203^ | 1 study ^128^  pIC_50 flavonoid_: 5.46  pIC_50_ _acarbose_: 3.95 |
| **100** | Rutin (Quercetin-3-rutinoside) | 20 studies ^20,48,61,108-110,118,128,156,160,195,198,205,221,233,241,274,283,290,342^  pIC_50 flavonoid_: 3.78 (3.69 - 4.42)  pIC_50_ _acarbose_: 3.73 (3.11 - 4.11)  Mechanism: mixed ^108,241^ | 5 studies ^47,111,128,195,206^  pIC_50 flavonoid_: 3.83 (3.58 - 4.36)  pIC_50_ _acarbose_: 4.83 (3.95 - 4.95)  Mechanism: non-competitive ^206^ |
| **101** | Quercetin-3-*O*-(6''-*O*-acetyl)-β-D-glucopyranoside | 1 study ^48^  pIC_50 flavonoid_: 3.51  pIC_50_ _acarbose_: 3.92 | 1 study ^111^  pIC_50 flavonoid_: 4.19  pIC_50_ _acarbose_: 4.83 |
| **102** | Quercetin-3-*O*-glucuronide | 3 studies ^59,183,191^  pIC_50 flavonoid_: 3.88 (3.8 - 4.04)  pIC_50_ _acarbose_: 4.09 (3.71 - 4.17)  Mechanism: competitive ^183^ | 1 study ^59^  pIC_50 flavonoid_: 3.57  pIC_50_ _acarbose_: 4.00 |
| **103** | Avicularin (Quercetin-3-*O*-α-L-arabinofuranoside) | 7 studies ^29,82,176,183,253,296,336^  pIC_50 flavonoid_: 3.38 (3.15 - 4.04)  pIC_50_ _acarbose_: 3.53 (3.35 - 3.61)  Mechanism: Mixed ^183^ | 1 study ^29^  pIC_50 flavonoid_: 2.23  pIC_50_ _acarbose_: 2.64 |
| **104** | Quercetin-3-*O*-[α-L-rhamnopyranosyl-(1⭢6)]-β-D-galactopyranose | 1 study ^202^  pIC_50 flavonoid_: 4.43  pIC_50_ _acarbose_: 3.75 | 1 study ^202^  pIC_50 flavonoid_: 6.30  pIC_50_ _acarbose_: 5.56 |
| **105** | Quercetin-3-*O*-[glucopyranosyl-(1⭢3)][α-L-rhamnopyranosyl-(1⭢6)]-β-D-glucopyranose | 1 study ^202^  pIC_50 flavonoid_: 4.18  pIC_50_ _acarbose_: 3.75 | 1 study ^202^  pIC_50 flavonoid_: 5.80  pIC_50_ _acarbose_: 5.56 |
| **106** | Quercetin-3-*O*-[glucopyranosyl-(1🠖3)][α-L-rhamnopyranosyl-(1🠖6)]-β-D-galactopyranose | 1 study ^202^  pIC_50 flavonoid_: 4.17  pIC_50_ _acarbose_: 3.75 | 1 study ^202^  pIC_50 flavonoid_: 5.88  pIC_50_ _acarbose_: 5.56 |
| **107** | Quercetin 3-*O*-[(*E*)-p-coumaroyl-(1⭢2)][α-L-arabinopyranosyl-(1⭢3)][ α-L-rhamnopyranosyl (1⭢6)]-β-D-glucopyranoside | 1 study ^202^  pIC_50 flavonoid_: 4.09  pIC_50_ _acarbose_: 3.75 | 1 study ^202^  pIC_50 flavonoid_: 4.99  pIC_50_ _acarbose_: 5.56 |
| **108** | Quercetin 3-*O*-[(*E*)-p-coumaroyl-(1⭢2)][α-L-arabinopyranosyl-(1⭢3)][β-D-glucopyranosyl (1⭢3)-α-L-rhamnopyranosyl (1⭢6)]-β-D-glucopyranoside | 1 study ^202^  pIC_50 flavonoid_: 3.97  pIC_50_ _acarbose_: 3.75 | 1 study ^202^  pIC_50 flavonoid_: 5.52  pIC_50_ _acarbose_: 5.56 |
| **109** | Astragalin  (Kaempferol-3-*O*-β-D-glucopyranoside) | 11 studies ^36,48,156,168,188,202,264,283,297,300,342^  pIC_50 flavonoid_: 3.38 (2.96 - 4.42)  pIC_50_ _acarbose_: 3.52 (3.22 - 3.86)  Mechanism: non-competitive ^168,283^ | 3 studies ^188,202,230^  pIC_50 flavonoid_: 4.48 (3.71 - 4.8)  pIC_50_ _acarbose_: 3.63 (3.54 - 4.59) |
| **110** | Kaempferol-3-rutinoside  (Nicotiflorin) | 12 studies ^20,28,48,118,128,156,202,213,233,274,283,342^  pIC_50 flavonoid_: 3.71 (3.56 - 4.47)  pIC_50_ _acarbose_: 3.82 (3.03 - 4.02)  Mechanism: competitive,^213^  uncompetitive ^283^ | 3 studies ^47,128,202^  pIC_50 flavonoid_: 4.72 (4.09 - 5.88)  pIC_50_ _acarbose_: 3.95 (3.95 - 4.75) |
| **111** | Kaempferol-3-*O*-[α-L-rhamnopyranosyl-(1⭢6)]-β-D-galactopyranoside | 1 study ^202^  pIC_50 flavonoid_: 4.42  pIC_50_ _acarbose_: 3.75 | 1 study ^202^  pIC_50 flavonoid_: 5.58  pIC_50_ _acarbose_: 5.56 |
| **112** | Kaempferol-3-*O*-[glucopyranosyl-(1⭢3)][α-L-rhamnopyranosyl-(1⭢6)]-β-D-glucopyranoside | 1 study ^202^  pIC_50 flavonoid_: 4.15  pIC_50_ _acarbose_: 3.75 | 1 study ^202^  pIC_50 flavonoid_: 5.52  pIC_50_ _acarbose_: 5.56 |
| **113** | Kaempferol-3-*O*-[glucopyranosyl-(1🠖3)][α-L-rhamnopyranosyl-(1🠖6)]-β-D-galactopyranoside | 1 study ^202^  pIC_50 flavonoid_: 4.13  pIC_50_ _acarbose_: 3.75 | 1 study ^202^  pIC_50 flavonoid_: 4.90  pIC_50_ _acarbose_: 5.56 |
| **114** | Kaempferol 3-*O*-[(*E*)-p-coumaroyl-(1⭢2)][α-L-arabinopyranosyl-(1⭢3)][ α-L-rhamnopyranosyl (1⭢6)]-β-D-glucopyranoside | 1 study ^202^  pIC_50 flavonoid_: 4.19  pIC_50_ _acarbose_: 3.75 | 1 study ^202^  pIC_50 flavonoid_: 4.95  pIC_50_ _acarbose_: 5.56 |
| **115** | Camellikaempferoside C | 1 study ^202^  pIC_50 flavonoid_: 4.10  pIC_50_ _acarbose_: 3.75 | 1 study ^202^  pIC_50 flavonoid_: 4.92  pIC_50_ _acarbose_: 5.56 |
| **116** | Myricitrin  (Myricetin-3-rhamnoside) | 9 studies ^13,57,109,155,169,274,281,291,336^  pIC_50 flavonoid_: 3.48 (3.11 - 3.77)  pIC_50_ _acarbose_: 3.67 (3.23 - 5.23) | 2 studies ^155,281^  pIC_50 flavonoid_: 4.23 (3.48 - 4.97)  pIC_50_ _acarbose_: 5.09 (4.91 - 5.27) |
| **117** | Europetin-3-*O*-rhamnoside | 1 study ^155^  pIC_50 flavonoid_: 5.72  pIC_50_ _acarbose_: 4.37 | 1 study ^155^  pIC_50 flavonoid_: 5.64  pIC_50_ _acarbose_: 4.72 |
| **118** | Isorhamnetin-3-*O*-glucoside | 3 studies ^138,185,264^  pIC_50 flavonoid_: 3.46 (3.02 - 3.66)  pIC_50_ _acarbose_: 2.72 (2.44 - 4.41) | 3 studies ^111,215,230^  pIC_50 flavonoid_: 3.21 (2.94 - 3.76)  pIC_50_ _acarbose_: 4.6 (4.02 - 4.72) |
| **119** | Isorhamnetin-7-*O*-β-D-glucopyranuronide | 1 study ^79^  pIC_50 flavonoid_: 4.93  pIC_50_ _acarbose_: 5.71 | 1 study ^79^  pIC_50 flavonoid_: 4.79  pIC_50_ _acarbose_: 5.83 |
| **120** | Isorhamnetin-3-*O*-rutinoside | 4 studies ^109,117,128,180^  pIC_50 flavonoid_: 3.81 (3.49 - 4.19)  pIC_50_ _acarbose_: 3.8 (3.53 - 4.47) | 2 studies ^128,215^  pIC_50 flavonoid_: 4.24 (4.06 - 4.41)  pIC_50_ _acarbose_: 4.27 (4.11 - 4.44) |
| **121** | Quercetagetin-7-*O*-β-D-glucopyranoside | 2 studies ^33,79^  pIC_50 flavonoid_: 4.35 (3.87 - 4.83)  pIC_50_ _acarbose_: 4.63 (4.09 - 5.17)  Mechanism: mixed ^79^ | 1 study ^79^  pIC_50 flavonoid_: 5.09  pIC_50_ _acarbose_: 5.83  Mechanism: mixed ^79^ |

# Table S11. *In vitro* α-glucosidase and α-amylase inhibitory effects of retrieved anthocyanidin derivatives.

| **No** | **Name** | **α-glucosidase** | **α-amylase** |
| --- | --- | --- | --- |
| **122** | Pelargonidin | 1 study ^58^  pIC_50 flavonoid_: 6.76  pIC_50_ _acarbose_: 5.55 | 2 studies ^58,307^  pIC_50 flavonoid_: 4.51 (3.92 - 5.1)  pIC_50_ _acarbose_: 5.12 (5.06 - 5.19) |
| **123** | Cyanidin | 3 studies ^181,209,265^  pIC_50 flavonoid_: 5.28 (5.01 - 5.56)  pIC_50_ _acarbose_: 3.88 (3.33 - 5.46)  Mechanism: non-competitive ^181^ | 3 studies ^209,265,307^  pIC_50 flavonoid_: 4.79 (4.14 - 5.61)  pIC_50_ _acarbose_: 5.25 (4.60 - 6.08) |
| **124** | Delphinidin | 2 studies ^58,239^  pIC_50 flavonoid_: 5.71 (5.27 - 6.14)  pIC_50_ _acarbose_: 4.61 (4.15 - 5.08)  Mechanism: mixed ^239^ | 1 study ^58^  pIC_50 flavonoid_: 6.22  pIC_50_ _acarbose_: 5.00 |
| **125** | Cyanidin-3-*O*-glucoside | 6 studies ^109,193,209,262,265,291^  pIC_50 flavonoid_: 4.06 (3.24 - 5.03)  pIC_50_ _acarbose_: 4.09 (3.56 - 5.75) | 3 studies ^46,209,265^  pIC_50 flavonoid_: 5.43 (4.59 - 5.98)  pIC_50_ _acarbose_: 6.30 (5.12 - 6.61)  Mechanism: competitive ^46^ |
| **126** | Cyanidin-3-*O*-sambubioside | 1 study ^265^  pIC_50 flavonoid_: 5.55  pIC_50_ _acarbose_: 3.88 | 1 study ^265^  pIC_50 flavonoid_: 5.64  pIC_50_ _acarbose_: 3.95 |
| **127** | Cyanidin-3-*O*-rutinoside (Antirrhinin) | 1 study ^262^  pIC_50 flavonoid_: 3.22  pIC_50_ _acarbose_: 3.45 | 2 studies ^46,209^  pIC_50 flavonoid_: 4.16 (3.93 - 4.38)  pIC_50_ _acarbose_: 5.52 (5.13 - 5.91)  Mechanism: competitive,^46^ non-competitive ^209^ |
| **128** | Malvidin-3-*O*-glucoside | 1 study ^262^  pIC_50 flavonoid_: 3.93  pIC_50_ _acarbose_: 3.45 | 2 studies ^46,85^  pIC_50 flavonoid_: 3.33 (3.25 - 3.4)  pIC_50_ _acarbose_: 5.39 (4.94 - 5.85)  Mechanism: competitive ^46^ |

# Table S12. *In vitro* α-glucosidase and α-amylase inhibitory effects of aurone and chalcone derivatives.

| **No** | **Name** | **α-glucosidase** | **α-amylase** |
| --- | --- | --- | --- |
| **129** | *(Z)*-6-(2-Benzylidene-4,6-dihydroxy-3-oxo-2,3-dihydrobenzofuran-7-yl)-7-methoxy-2*H*-chromen-2-one | 1 study ^312^  pIC_50 flavonoid_: 5.45  pIC_50_ _acarbose_: 3.65  Mechanism: mixed ^312^ | 1 study ^312^  pIC_50 flavonoid_: 4.96  pIC_50_ _acarbose_: 5.57 |
| **130** | Desmethylxanthohumol (3'-prenylchalconarigenin) | 2 studies ^244,320^  pIC_50 flavonoid_: 4.65 (4.65 - 4.65)  pIC_50_ _acarbose_: 4.01 (3.87 - 4.15) | 1 study ^244^  pIC_50 flavonoid_: 4.07  pIC_50_ _acarbose_: 5.66 |
| **131** | 3'-Geranylchalconaringenin | 1 study ^244^  pIC_50 flavonoid_: 5.97  pIC_50_ _acarbose_: 4.29  Mechanism: competitive ^244^ | 1 study ^244^  pIC_50 flavonoid_: 4.69  pIC_50_ _acarbose_: 5.66 |
| **132** | Butein | 1 study ^23^  pIC_50 flavonoid_: 4.68  pIC_50_ _acarbose_: 3.45  Mechanism: competitive ^23^ | 1 study ^23^  pIC_50 flavonoid_: 4.21  pIC_50_ _acarbose_: 5.96  Mechanism: competitive ^23^ |
| **133** | *(E)*-3-(2-Methoxyphenyl)-1-(pyridin-2-yl)prop-2-en-1-one | 1 study ^317^  pIC_50 flavonoid_: 4.58  pIC_50_ _acarbose_: 4.73  Mechanism: competitive ^317^ | 1 study ^317^  pIC_50 flavonoid_: 4.64  pIC_50_ _acarbose_: 4.74  Mechanism: competitive ^317^ |
| **134** | *(E)*-3-(3,5-Dimethoxyphenyl)-1-(pyridin-2-yl)prop-2-en-1-one | 1 study ^317^  pIC_50 flavonoid_: 4.19  pIC_50_ _acarbose_: 4.73 | 1 study ^317^  pIC_50 flavonoid_: 4.21  pIC_50_ _acarbose_: 4.74 |
| **135** | *(E)*-1-(Pyridin-2-yl)-3-(2,3,4-trimethoxyphenyl)prop-2-en-1-one | 1 study ^317^  pIC_50 flavonoid_: 4.56  pIC_50_ _acarbose_: 4.73  Mechanism: competitive ^317^ | 1 study ^317^  pIC_50 flavonoid_: 4.61  pIC_50_ _acarbose_: 4.74  Mechanism: competitive ^317^ |
| **136** | *(E)*-3-(2-Fluoro-4-methoxyphenyl)-1-(pyridin-2-yl)prop-2-en-1-one | 1 study ^317^  pIC_50 flavonoid_: 4.56  pIC_50_ _acarbose_: 4.73  Mechanism: competitive ^317^ | 1 study ^317^  pIC_50 flavonoid_: 4.60  pIC_50_ _acarbose_: 4.74  Mechanism: competitive ^317^ |
| **137** | *(E)*-3-(4-Fluoro-3-methoxyphenyl)-1-(pyridin-2-yl)prop-2-en-1-one | 1 study ^317^  pIC_50 flavonoid_: 4.32  pIC_50_ _acarbose_: 4.73 | 1 study ^317^  pIC_50 flavonoid_: 4.35  pIC_50_ _acarbose_: 4.74 |
| **138** | *(E)*-3-(2-Chloro-3-methoxyphenyl)-1-(pyridin-2-yl)prop-2-en-1-one | 1 study ^317^  pIC_50 flavonoid_: 4.41  pIC_50_ _acarbose_: 4.73 | 1 study ^317^  pIC_50 flavonoid_: 4.42  pIC_50_ _acarbose_: 4.74 |
| **139** | *(E)*-3-(2-Bromo-6-methoxyphenyl)-1-(pyridin-2-yl)prop-2-en-1-one | 1 study ^317^  pIC_50 flavonoid_: 4.17  pIC_50_ _acarbose_: 4.73 | 1 study ^317^  pIC_50 flavonoid_: 4.18  pIC_50_ _acarbose_: 4.74 |
| **140** | *(E)*-3-(4-Bromo-3,5-dimethoxyphenyl)-1-(pyridin-2-yl)prop-2-en-1- one | 1 study ^317^  pIC_50 flavonoid_: 4.31  pIC_50_ _acarbose_: 4.73 | 1 study ^317^  pIC_50 flavonoid_: 4.32  pIC_50_ _acarbose_: 4.74 |
| **141** | *(E)*-3-(2-Bromo-4,5-dimethoxyphenyl)-1-(pyridin-2-yl)prop-2-en-1- one | 1 study ^317^  pIC_50 flavonoid_: 4.19  pIC_50_ _acarbose_: 4.73 | 1 study ^317^  pIC_50 flavonoid_: 4.20  pIC_50_ _acarbose_: 4.74 |
| **142** | *(E)*-3-(4-Chlorophenyl)-1-(pyridin-2-yl)prop-2-en-1-one | 1 study ^317^  pIC_50 flavonoid_: 4.32  pIC_50_ _acarbose_: 4.73 | 1 study ^317^  pIC_50 flavonoid_: 4.35  pIC_50_ _acarbose_: 4.74 |
| **143** | *(E)*-3-(2,4-Dichlorophenyl)-1-(pyridin-2-yl)prop-2-en-1-one | 1 study ^317^  pIC_50 flavonoid_: 4.43  pIC_50_ _acarbose_: 4.73 | 1 study ^317^  pIC_50 flavonoid_: 4.46  pIC_50_ _acarbose_: 4.74 |
| **144** | *(E)*-3-(5-Chloro-2-hydroxyphenyl)-1-(pyridin-2-yl)prop-2-en-1- one | 1 study ^317^  pIC_50 flavonoid_: 4.39  pIC_50_ _acarbose_: 4.73 | 1 study ^317^  pIC_50 flavonoid_: 4.40  pIC_50_ _acarbose_: 4.74 |
| **145** | *(E)*-3-(3,5-Dichloro-2-hydroxyphenyl)-1-(pyridin-2-yl)prop-2-en-1-one | 1 study ^317^  pIC_50 flavonoid_: 4.06  pIC_50_ _acarbose_: 4.73 | 1 study ^317^  pIC_50 flavonoid_: 4.05  pIC_50_ _acarbose_: 4.74 |
| **146** | *(E)*-3-(3-Nitrophenyl)-1-(pyridin-2-yl)prop-2-en-1-one | 1 study ^317^  pIC_50 flavonoid_: 4.54  pIC_50_ _acarbose_: 4.73  Mechanism: competitive ^317^ | 1 study ^317^  pIC_50 flavonoid_: 4.56  pIC_50_ _acarbose_: 4.74  Mechanism: competitive ^317^ |
| **147** | *(E)*-3-(4-Nitrophenyl)-1-(pyridin-2-yl)prop-2-en-1-one | 1 study ^317^  pIC_50 flavonoid_: 4.10  pIC_50_ _acarbose_: 4.73 | 1 study ^317^  pIC_50 flavonoid_: 4.11  pIC_50_ _acarbose_: 4.74 |
| **148** | *(E)*-3-(2-Chloro-5-nitrophenyl)-1-(pyridin-2-yl)prop-2-en-1-one | 1 study ^317^  pIC_50 flavonoid_: 4.27  pIC_50_ _acarbose_: 4.73 | 1 study ^317^  pIC_50 flavonoid_: 4.28  pIC_50_ _acarbose_: 4.74 |
| **149** | *(E)*-3-(Naphthalen-2-yl)-1-(pyridin-2-yl)prop-2-en-1-one | 1 study ^317^  pIC_50 flavonoid_: 4.12  pIC_50_ _acarbose_: 4.73 | 1 study ^317^  pIC_50 flavonoid_: 4.11  pIC_50_ _acarbose_: 4.74 |
| **150** | *(E)*-3-(3,5-Dimethoxyphenyl)-1-(pyridin-3-yl)prop-2-en-1-one | 1 study ^317^  pIC_50 flavonoid_: 4.05  pIC_50_ _acarbose_: 4.73 | 1 study ^317^  pIC_50 flavonoid_: 4.06  pIC_50_ _acarbose_: 4.74 |
| **151** | *(E)*-1-(Pyridin-3-yl)-3-(2,3,4-trimethoxyphenyl)prop-2-en-1-one | 1 study ^317^  pIC_50 flavonoid_: 4.07  pIC_50_ _acarbose_: 4.73 | 1 study ^317^  pIC_50 flavonoid_: 4.05  pIC_50_ _acarbose_: 4.74 |
| **152** | *(E)*-3-(2-Fluoro-4-methoxyphenyl)-1-(pyridin-3-yl)prop-2-en-1- one | 1 study ^317^  pIC_50 flavonoid_: 4.34  pIC_50_ _acarbose_: 4.73 | 1 study ^317^  pIC_50 flavonoid_: 4.37  pIC_50_ _acarbose_: 4.74 |
| **153** | *(E)*-3-(4-Fluoro-3-methoxyphenyl)-1-(pyridin-3-yl)prop-2-en-1- one | 1 study ^317^  pIC_50 flavonoid_: 4.05  pIC_50_ _acarbose_: 4.73 | 1 study ^317^  pIC_50 flavonoid_: 4.06  pIC_50_ _acarbose_: 4.74 |
| **154** | *(E)*-3-(2-Chloro-3-methoxyphenyl)-1-(pyridin-3-yl)prop-2-en-1- one | 1 study ^317^  pIC_50 flavonoid_: 4.09  pIC_50_ _acarbose_: 4.73 | 1 study ^317^  pIC_50 flavonoid_: 4.09  pIC_50_ _acarbose_: 4.74 |
| **155** | *(E)*-3-(4-Bromo-3,5-dimethoxyphenyl)-1-(pyridin-3-yl)prop-2-en-1-one | 1 study ^317^  pIC_50 flavonoid_: 4.06  pIC_50_ _acarbose_: 4.73 | 1 study ^317^  pIC_50 flavonoid_: 4.07  pIC_50_ _acarbose_: 4.74 |
| **156** | *(E)*-3-(2-Chloro-5-nitrophenyl)-1-(pyridin-3-yl)prop-2-en-1-one | 1 study ^317^  pIC_50 flavonoid_: 4.55  pIC_50_ _acarbose_: 4.73  Mechanism: competitive ^317^ | 1 study ^317^  pIC_50 flavonoid_: 4.57  pIC_50_ _acarbose_: 4.74  Mechanism: competitive ^317^ |
| **157** | *(E)*-1-(2-Amino-5-(*(E)*-styryl)phenyl)-3-(3-fluorophenyl)prop-2-en-1-one | 1 study ^325^  pIC_50 flavonoid_: 4.75  pIC_50_ _acarbose_: 6.02 | 1 study ^325^  pIC_50 flavonoid_: 4.97  pIC_50_ _acarbose_: 5.99 |
| **158** | *(E)*-1-(2-Amino-5-(*(E)*-4-fluorostyryl)phenyl)-3-(3-fluorophenyl)prop-2-en-1-one | 1 study ^325^  pIC_50 flavonoid_: 5.21  pIC_50_ _acarbose_: 6.02 | 1 study ^325^  pIC_50 flavonoid_: 4.81  pIC_50_ _acarbose_: 5.99 |
| **159** | *(E)*-1-(2-Amino-5-(*(E)*-4-Chlorostyryl)phenyl)-3-(3-fluorophenyl)prop-2-en-1-one | 1 study ^325^  pIC_50 flavonoid_: 4.90  pIC_50_ _acarbose_: 6.02 | 1 study ^325^  pIC_50 flavonoid_: 5.62  pIC_50_ _acarbose_: 5.99 |
| **160** | *(E)*-1-(2-Amino-5-(*(E)*-4-methoxystyryl)phenyl)-3-(3-fluorophenyl)prop-2-en-1-one | 1 study ^325^  pIC_50 flavonoid_: 5.03  pIC_50_ _acarbose_: 6.02 | 1 study ^325^  pIC_50 flavonoid_: 5.15  pIC_50_ _acarbose_: 5.99 |
| **161** | *(E)*-1-(2-Amino-5-(*(E)*-styryl)phenyl)-3-(4-fluorophenyl)prop-2-en-1-one | 1 study ^325^  pIC_50 flavonoid_: 5.29  pIC_50_ _acarbose_: 6.02  Mechanism: non-competitive ^325^ | 1 study ^325^  pIC_50 flavonoid_: 5.80  pIC_50_ _acarbose_: 5.99 |
| **162** | *(E)*-1-(2-Amino-5-(*(E)*-4-fluorostyryl)phenyl)-3-(4-fluorophenyl)prop-2-en-1-one | 1 study ^325^  pIC_50 flavonoid_: 5.16  pIC_50_ _acarbose_: 6.02 | 1 study ^325^  pIC_50 flavonoid_: 5.02  pIC_50_ _acarbose_: 5.99 |
| **163** | *(E)*-1-(2-Amino-5-(*(E)*-4-chlorostyryl)phenyl)-3-(4-fluorophenyl)prop-2-en-1-one | 1 study ^325^  pIC_50 flavonoid_: 4.72  pIC_50_ _acarbose_: 6.02 | 1 study ^325^  pIC_50 flavonoid_: 5.77  pIC_50_ _acarbose_: 5.99 |
| **164** | *(E)*-1-(2-Amino-5-(*(E)*-4-methoxystyryl)phenyl)-3-(4-fluorophenyl)prop-2-en-1-one | 1 study ^325^  pIC_50 flavonoid_: 4.98  pIC_50_ _acarbose_: 6.02 | 1 study ^325^  pIC_50 flavonoid_: 5.12  pIC_50_ _acarbose_: 5.99 |
| **165** | Phloretin | 4 studies ^49,109,155,190^  pIC_50 flavonoid_: 4.45 (4.11 - 5.07)  pIC_50_ _acarbose_: 4.52 (4.4 - 5.04)  Mechanism: non-competitive ^190^ | 2 studies ^49,155^  pIC_50 flavonoid_: 5.52 (5.01 - 6.02)  pIC_50_ _acarbose_: 4.86 (4.79 - 4.93) |

# Table S13. *In vitro* α-glucosidase and α-amylase inhibitory effects of retrieved isoflavonoids.

| **No** | **Name** | **α-glucosidase** | **α-amylase** |
| --- | --- | --- | --- |
| **166** | Genistein | 8 studies ^58,109,139,182,225,288,323,326^  pIC_50 flavonoid_: 4.41 (3.58 - 5.48)  pIC_50_ _acarbose_: 4.07 (3.45 - 4.63)  Mechanism: mixed,^139^ non-competitive,^288,326^ uncompetitive ^182^ | 2 studies ^58,225^  pIC_50 flavonoid_: 4.91 (4.43 - 5.38)  pIC_50_ _acarbose_: 4.52 (4.29 - 4.76) |
| **167** | Formononetin | 3 studies ^58,109,302^  pIC_50 flavonoid_: 4.57 (3.85 - 5.43)  pIC_50_ _acarbose_: 5.55 (4.49 - 5.89) | 1 study ^58^  pIC_50 flavonoid_: 6.21  pIC_50_ _acarbose_: 5.00 |
| **168** | Dalbergioidin | 1 study ^225^  pIC_50 flavonoid_: 3.38  pIC_50_ _acarbose_: 3.17 | 1 study ^225^  pIC_50 flavonoid_: 3.79  pIC_50_ _acarbose_: 4.05 |
| **169** | Puerarin | 5 studies ^97,143,160,288,290^  pIC_50 flavonoid_: 3.83 (3.71 - 3.97)  pIC_50_ _acarbose_: 3.75 (3.11 - 3.84) | 1 study ^123^  pIC_50 flavonoid_: 3.40  pIC_50_ _acarbose_: 5.28 |

# Table S14. *In vitro* α-glucosidase and α-amylase inhibitory effects of retrieved oligomeric flavonoids.

| **No** | **Name** | **α-glucosidase** | **α-amylase** |
| --- | --- | --- | --- |
| **170** | Kuwanon M | 1 study ^292^  pIC_50 flavonoid_: 6.22  pIC_50_ _acarbose_: 3.53 | 1 study ^292^  pIC_50 flavonoid_: 5.91  pIC_50_ _acarbose_: 5.82 |
| **171** | Amentoflavone | 6 studies ^14,55,80,87,256,304^  pIC_50 flavonoid_: 4.57 (4.13 - 4.98)  pIC_50_ _acarbose_: 3.67 (3.56 - 3.95) | 2 studies ^55,232^  pIC_50 flavonoid_: 4.28 (4.21 - 4.35)  pIC_50_ _acarbose_: 4.69 (4.66 - 4.72) |
| **172** | 2,3-Dihydroamentoflavone | 1 study ^55^  pIC_50 flavonoid_: 5.01  pIC_50_ _acarbose_: 3.27 | 1 study ^55^  pIC_50 flavonoid_: 4.40  pIC_50_ _acarbose_: 4.75 |
| **173** | Procyanidin C1 | 1 study ^265^  pIC_50 flavonoid_: 5.28  pIC_50_ _acarbose_: 3.88 | 1 study ^265^  pIC_50 flavonoid_: 5.59  pIC_50_ _acarbose_: 3.95 |
| **174** | Procyanidin B2 | 2 studies ^265,284^  pIC_50 flavonoid_: 5.72 (5.38 - 6.06)  pIC_50_ _acarbose_: 3.39 (3.15 - 3.64)  Mechanism: mixed ^284^ | 1 study ^265^  pIC_50 flavonoid_: 5.18  pIC_50_ _acarbose_: 3.95 |
| **175** | Procyanidin B5 | 1 study ^265^  pIC_50 flavonoid_: 4.92  pIC_50_ _acarbose_: 3.88 | 1 study ^265^  pIC_50 flavonoid_: 5.15  pIC_50_ _acarbose_: 3.95 |
| **176** | Epicatechin-(2β-*O*-7,4β -8)-[catechin-(6-4β)]-epicatechin | 1 study ^222^  pIC_50 flavonoid_: 5.34  pIC_50_ _acarbose_: 3.18 | 1 study ^222^  pIC_50 flavonoid_: 5.46  pIC_50_ _acarbose_: 5.23 |
| **177** | Procyanidin A1 | 1 study ^222^  pIC_50 flavonoid_: 5.21  pIC_50_ _acarbose_: 3.18 | 1 study ^222^  pIC_50 flavonoid_: 5.08  pIC_50_ _acarbose_: 5.23 |

# References

1. Page MJ, McKenzie JE, Bossuyt PM, et al. The PRISMA 2020 statement: an updated guideline for reporting systematic reviews. *BMJ*. 2021;372:n71. doi:<https://doi.org/10.1136/bmj.n71>

2. Ouzzani M, Hammady H, Fedorowicz Z, Elmagarmid A. Rayyan—a web and mobile app for systematic reviews. *Syst Rev*. 2016;5(1):210. doi:<https://doi.org/10.1186/s13643-016-0384-4>

3. Perkin Elmer. ChemDraw - The gold standard for communicating chemistry research. Accessed Dec 13 2022, <https://perkinelmerinformatics.com/products/research/chemdraw/>

4. Faggion CM, Jr. Guidelines for reporting pre-clinical in vitro studies on dental materials. *J Evid Based Dent Pract*. 2012;12(4):182-9. doi:<https://doi.org/10.1016/j.jebdp.2012.10.001>

5. Landrum G. RDKit: open-source cheminformatics software. Accessed Oct 09, 2022, <http://www.rdkit.org>

6. Yao Y, Cheng X, Wang L, Wang S, Ren G. A determination of potential α-glucosidase inhibitors from Azuki Beans (*Vigna angularis*). *Int J Mol Sci*. 2011;12(10):6445-51. doi:<https://doi.org/10.3390/ijms12106445>

7. Van Thanh B, Van Anh NT, Ha CTT, et al. A new 2,3-dioxygenated flavanone and other constituents from *Dysosma difformis*. *Rec Nat Prod*. 2022;16(1):92-97. doi:<https://doi.org/10.25135/rnp.256.21.03.2017>

8. El-Nashar HAS, Mostafa NM, Eldahshan OA, Singab ANB. A new antidiabetic and anti-inflammatory biflavonoid from *Schinus polygama* (Cav.) Cabrera leaves. *Nat Prod Res*. 2022;36(5):1182-1190. doi:<https://doi.org/10.1080/14786419.2020.1864365>

9. Jia C, Han T, Xu J, et al. A new biflavonoid and a new triterpene from the leaves of *Garcinia paucinervis* and their biological activities. *J Nat Med*. 2017;71(4):642-649. doi:<https://doi.org/10.1007/s11418-017-1092-7>

10. Nguyen TP, Le TD, Minh PN, et al. A new dihydrofurocoumarin from the fruits of *Pandanus tectorius* Parkinson ex Du Roi. *Nat Prod Res*. 2016;30(21):2389-95. doi:<https://doi.org/10.1080/14786419.2016.1188095>

11. Zhang Y, Xiao G, Sun L, Wang Y, Wang Y, Wang Y. A new flavan-3-ol lactone and other constituents from *Euonymus alatus* with inhibitory activities on a-glucosidase and differentiation of 3T3-L1 cells. *Nat Prod Res*. 2013;27(17):1513-1520. doi:<https://doi.org/10.1080/14786419.2012.725400>

12. Wu S, Tian L. A new flavone glucoside together with known ellagitannins and flavones with anti-diabetic and anti-obesity activities from the flowers of pomegranate (*Punica granatum*). *Nat Prod Res*. 2019;33(2):252-257. doi:<https://doi.org/10.1080/14786419.2018.1446009>

13. Thuy NTL, Thuy PT, Tung BT, et al. A new flavone glycoside from *Lumnitzera littorea* with in vitro α-glucosidase inhibitory activity. *Nat Prod Commun*. 2019;doi:<https://doi.org/10.1177/1934578X19851361>

14. Nguyen TTH, Nguyen VT, Van Cuong P, et al. A new flavonoid from the leaves of *Garcinia mckeaniana* Craib and α-glucosidase and acetylcholinesterase inhibitory activities. *Nat Prod Res*. 2021;36(19):5074-5080. doi:<https://doi.org/10.1080/14786419.2021.1916019>

15. Liu Y, Huang X-H, Chen J, Shao J-H, Zhao C-C. A new flavonoid glycoside from *Scutellaria barbata*. *Chem Nat Compd*. 2020;56(6):1016-1018. doi:<https://doi.org/10.1007/s10600-020-03217-6>

16. Zhang X-H, Shen J, Zhao C-C, Shao J-H. A new flavonoid glycoside with α-glucosidase inhibitory activity from *Galium verum*. *Chem Nat Compd*. 2020;56(1):67-69. doi:<https://doi.org/10.1007/s10600-020-02945-z>

17. Phuong NH, Thuy NTL, Duc NT, Tuyet NTA, Mai NTT, Phung NKP. A new glycoside and in vitro evalution of alpha-glucosidase inhibitory activity of constituents of the mangrove *Lumnitzera racemosa*. *Nat Prod Commun*. 2017;12(11):1751-1754. doi:<https://doi.org/10.1177/1934578x1701201125>

18. Zhou Q, Lei X, Niu J, Chen Y, Shen X, Zhang N. A new hemiacetal chromone racemate and α-glucosidase inhibitors from *Ficus tikoua* Bur. *Nat Prod Res*. 2022;doi:<https://doi.org/10.1080/14786419.2022.2068544>

19. Helal IE, Elsbaey M, Zaghloul AM, Mansour E-SS. A new homoisoflavan from *Dracaena cinnabari* Balf. f. resin: α-glucosidase and COX-II inhibitory activity. *Nat Prod Res*. 2022;36(5):1224-1229. doi:<https://doi.org/10.1080/14786419.2020.1869229>

20. Parveen A, Farooq MA, Kyunn WW. A new oleanane type saponin from the aerial parts of *Nigella sativa* with anti-oxidant and anti-diabetic potential. *Molecules*. 2020;25(9):2171. doi:<https://doi.org/10.3390/molecules25092171>

21. Li K, Li S, Xu F, Cao G, Gong X. A novel acylated quercetin glycoside and compounds of inhibitory effects on α-glucosidase from *Panax ginseng* flower buds. *Nat Prod Res*. 2020;34(18):2559-2565. doi:<https://doi.org/10.1080/14786419.2018.1543685>

22. Kim TH. A novel alpha-glucosidase inhibitory constituent from *Uncaria gambir*. *J Nat Med*. 2016;70(4):811-815. doi:<https://doi.org/10.1007/s11418-016-1014-0>

23. Rocha S, Sousa A, Ribeiro D, et al. A study towards drug discovery for the management of type 2 diabetes mellitus through inhibition of the carbohydrate-hydrolyzing enzymes α-amylase and α-glucosidase by chalcone derivatives. *Food Funct*. 2019;10(9):5510-5520. doi:<https://doi.org/10.1039/c9fo01298b>

24. Wan C, Zhou S. Acylated flavonoid from *Vaccinium corymbosum* (Ericaceae) flowers with yeast α-glucosidase inhibitory activity. *Trop J Pharm Res*. 2013;12(4):549-552. doi:<https://doi.org/10.4314/tjpr.v12i4.16>

25. Kashchenko NI, Chirikova NK, Olennikov DN. Acylated flavonoids from *Spiraea* genus as inhibitors of α-amylase. *Russ J Bioorganic Chem*. 2018;44(7):876-886. doi:<https://doi.org/10.1134/S1068162018070051>

26. Lee S-S, Lin H-C, Chen C-K. Acylated flavonol monorhamnosides, alpha-glucosidase inhibitors, from *Machilus philippinensis*. *Phytochemistry*. 2008;69(12):2347-53. doi:<https://doi.org/10.1016/j.phytochem.2008.06.006>

27. Chang CC, Ho SL, Lee SS. Acylated glucosylflavones as α-glucosidase inhibitors from *Tinospora crispa* leaf. *Bioorg Med Chem*. 2015;23(13):3388-96. doi:<https://doi.org/10.1016/j.bmc.2015.04.053>

28. Habtemariam S. Alpha-glucosidase inhibitory activity of kaempferol-3-O-rutinoside. *Nat Prod Commun*. 2011;6(2):201-203. doi:<https://doi.org/10.1177/1934578x1100600211>

29. Wang H, Du YJ, Song HC. Alpha-glucosidase and alpha-amylase inhibitory activities of Guava leaves. *Food Chem*. 2010;123(1):6-13. doi:<https://doi.org/10.1016/j.foodchem.2010.03.088>

30. Etsassala N, Badmus JA, Marnewick JL, Iwuoha EI, Nchu F, Hussein AA. Alpha-glucosidase and alpha-amylase inhibitory activities, molecular docking, and antioxidant capacities of *Salvia aurita* Constituents. *Antioxidants*. 2020;9(11):1149. doi:<https://doi.org/10.3390/antiox9111149>

31. Hussain M, Ahmed Z, Khan S, et al. α-Glucosidase inhibition and docking studies of 5-deoxyflavonols and dihydroflavonols isolated from *Abutilon pakistanicum*. *Curr Org Chem*. 2019;23:1857 - 1866. doi:<https://doi.org/10.2174/1385272823666191001224741>

32. Gao H, Kawabata J. Alpha-glucosidase inhibition of 6-hydroxyflavones. Part 3: Synthesis and evaluation of 2,3,4-trihydroxybenzoyl-containing flavonoid analogs and 6-aminoflavones as alpha-glucosidase inhibitors. *Bioorg Med Chem*. 2005;13(5):1661-1671. doi:<https://doi.org/10.1016/j.bmc.2004.12.010>

33. Gutierrez-Gonzalez JA, Perez-Vasquez A, Torres-Colin R, Rangel-Grimaldo M, Rebollar-Ramos D, Mata R. Alpha-glucosidase inhibitors from *Ageratina grandifolia*. *J Nat Prod*. 2021;84(5):1573-1578. doi:<https://doi.org/10.1021/acs.jnatprod.1c00105>

34. Escandon-Rivera S, Gonzalez-Andrade M, Bye R, Linares E, Navarrete A, Mata R. Alpha-glucosidase inhibitors from *Brickellia cavanillesii*. *J Nat Prod*. 2012;75(5):968-974. doi:<https://doi.org/10.1021/np300204p>

35. Kazemi R, Delnavazi M-R, Parsa-Khankandi H, et al. α-Glucosidase inhibitors from *Marrubium astracanicum*: Isolation and molecular docking. *Rev Bras Farmacogn*. 2022;32(4):618-626. doi:<https://doi.org/10.1007/s43450-022-00287-1>

36. Ha K-N, Nguyen T-V-A, Mai D-T, et al. Alpha-glucosidase inhibitors from *Nervilia concolor*, *Tecoma stans*, and *Bouea macrophylla*. *Saudi J Biol Sci*. 2022;29(2):1029-1042. doi:<https://doi.org/10.1016/j.sjbs.2021.09.070>

37. Matsui T, Ueda T, Oki T, Sugita K, Terahara N, Matsumoto K. Alpha-glucosidase inhibitory action of natural acylated anthocyanins. 1. Survey of natural pigments with potent inhibitory activity. *J Agric Food Chem*. 2001;49(4):1948-51. doi:<https://doi.org/10.1021/jf001251u>

38. Nguyen TNT, Le TD, Nguyen PL, et al. α-Glucosidase inhibitory activity and quantitative contribution of phenolic compounds from Vietnamese *Aquilaria crassna* leaves. *Nat Prod Commun*. 2022;17(3):1934578X221080326. doi:<https://doi.org/10.1177/1934578X221080326>

39. Joycharat N, Issarachote P, Sontimuang C, Voravuthikunchai SP. Alpha-glucosidase inhibitory activity of ethanol extract, fractions and purified compounds from the wood of *Albizia myriophylla*. *Nat Prod Res*. 2018;32(11):1291-1294. doi:<https://doi.org/10.1080/14786419.2017.1333990>

40. Vi LNT, Tuan NN, Hung QT, et al. Alpha-glucosidase inhibitory activity of extracts and compounds from the leaves of *Ruellia tuberosa* L. *Nat Prod J*. 2022;12(5):63-68. doi:<https://doi.org/10.2174/2210315511666210218214955>

41. Nguyena P, Ngo Q, Nguyen T, Maccarone A, Pyne S. Alpha-glucosidase inhibitory activity of the extracts and major phytochemical components of *Smilax glabra* Roxb. *Nat Prod J*. 2020;10(1):26-32. doi:<https://doi.org/10.2174/2210315509666190124111435>

42. Dej-Adisai S, Rais IR, Wattanapiromsakul C, Pitakbut T. Alpha-glucosidase inhibitory assay-screened isolation and molecular docking model from *Bauhinia pulla* active compounds. *Molecules*. 2021;26(19):5970. doi:<https://doi.org/10.3390/molecules26195970>

43. Tabussum A, Riaz N, Saleem M, et al. α-Glucosidase inhibitory constituents from *Chrozophora plicata*. *Phytochem Lett*. 2013;6(4):614-619. doi:<https://doi.org/10.1016/j.phytol.2013.08.005>

44. Lima Júnior JPD, Franco RR, Saraiva AL, Moraes IB, Espindola FS. *Anacardium humile* St. Hil as a novel source of antioxidant, antiglycation and α-amylase inhibitors molecules with potential for management of oxidative stress and diabetes. *J Ethnopharmacol*. 2021;268:113667. doi:<https://doi.org/10.1016/j.jep.2020.113667>

45. Xie H, Chen X, Li M, Aisa HA, Yuan T. Angustifolinoid B, a flavonoid glycoside dimer with cyclobutane from *Elaeagnus angustifolia* flowers. *Tetrahedron Lett*. 2020;61(23):151946. doi:<https://doi.org/10.1016/j.tetlet.2020.151946>

46. Homoki JR, Nemes A, Fazekas E, et al. Anthocyanin composition, antioxidant efficiency, and α-amylase inhibitor activity of different Hungarian sour cherry varieties (*Prunus cerasus* L.). *Food Chem*. 2016;194:222-9. doi:<https://doi.org/10.1016/j.foodchem.2015.07.130>

47. Swilam N, Nawwar MAM, Radwan RA, Mostafa ES. Antidiabetic activity and in silico molecular docking of polyphenols from *Ammannia baccifera* L. subsp. *Aegyptiaca* (Willd.) Koehne Waste: Structure elucidation of undescribed acylated flavonol diglucoside. *Plants*. 2022;11(3):452. doi:<https://doi.org/10.3390/plants11030452>

48. Wang Y, Xiang L, Wang C, Tang C, He X. Antidiabetic and antioxidant effects and phytochemicals of Mulberry fruit (*Morus alba* L.) polyphenol enhanced extract. *PLoS One*. 2013;8(7):e71144. doi:<https://doi.org/10.1371/journal.pone.0071144>

49. Gulcin I, Taslimi P, Aygun A, et al. Antidiabetic and antiparasitic potentials: Inhibition effects of some natural antioxidant compounds on alpha-glycosidase, alpha-amylase and human glutathione S-transferase enzymes. *Int J Biol Macromol*. 2018;119:741-746. doi:<https://doi.org/10.1016/j.ijbiomac.2018.08.001>

50. Junejo JA, Zaman K, Rudrapal M, Celik I, Attah EI. Antidiabetic bioactive compounds from *Tetrastigma angustifolia* (Roxb.) Deb and *Oxalis debilis* Kunth.: Validation of ethnomedicinal claim by in vitro and in silico studies. *S Afr J Bot*. 2021;143:164-175. doi:<https://doi.org/10.1016/j.sajb.2021.07.023>

51. Varghese GK, Bose LV, Habtemariam S. Antidiabetic components of *Cassia alata* leaves: Identification through alpha-glucosidase inhibition studies. *Pharm Biol*. 2013;51(3):345-349. doi:<https://doi.org/10.3109/13880209.2012.729066>

52. Zhu G, Luo Y, Xu X, Zhang H, Zhu M. Anti-diabetic compounds from the seeds of *Psoralea corylifolia*. *Fitoterapia*. 2019;139:104373. doi:<https://doi.org/10.1016/j.fitote.2019.104373>

53. Ridhasya F, Rahim N, Almurdani M, Hendra R, Teruna H. Antidiabetic constituents from *Helminthostachys zeylanica* (L) Hook (Ophioglossaceae). *Pharmacogn J*. 2020;12:223-226. doi:<https://doi.org/10.5530/pj.2020.12.33>

54. Ablat A, Halabi MF, Mohamad J, et al. Antidiabetic effects of *Brucea javanica* seeds in type 2 diabetic rats. *BMC Complement Altern Med*. 2017;17(1):94. doi:<https://doi.org/10.1186/s12906-017-1610-x>

55. Laishram S, Sheikh Y, Moirangthem DS, et al. Anti-diabetic molecules from *Cycas pectinata* Griff. traditionally used by the Maiba-Maibi. *Phytomedicine*. 2015;22(1):23-6. doi:<https://doi.org/10.1016/j.phymed.2014.10.007>

56. Jo YH, Lee S, Yeon SW, et al. Anti-diabetic potential of *Masclura tricuspidata* leaves: Prenylated isoflavonoids with α-glucosidase inhibitory and anti-glycation activity. *Bioorg Chem*. 2021;114:105098. doi:<https://doi.org/10.1016/j.bioorg.2021.105098>

57. Yoshikawa M, Shimada H, Nishida N, et al. Antidiabetic principles of natural medicines. lI. Aldose reductase and α-glucosidase inhibitors from brazilian natural medicine, the leaves of *Myrcia multiflora* DC. (Myrtaceae): Structures of myrciacitrins I and II and myrciaphenones A and B. *Chem Pharm Bull (Tokyo)*. 1998;46(1):113-119. doi:<https://doi.org/10.1248/cpb.46.113>

58. Demir Y, Durmaz L, Taslimi P, Gulçin İ. Antidiabetic properties of dietary phenolic compounds: Inhibition effects on α-amylase, aldose reductase, and α-glycosidase. *Biotechnol Appl Biochem*. 2019;66(5):781-786. doi:<https://doi.org/10.1002/bab.1781>

59. Ahmed S, Al-Rehaily AJ, Alam P, et al. Antidiabetic, antioxidant, molecular docking and HPTLC analysis of miquelianin isolated from *Euphorbia schimperi* C. Presl. *Saudi Pharm J*. 2019;27(5):655-663. doi:<https://doi.org/10.1016/j.jsps.2019.03.008>

60. Sun Q, Xu N-Y, Li Q-R, et al. Antiglycemic and anticomplementary potential of an edible plant *Gnaphalium hypoleucum* DC. *J Funct Foods*. 2017;38:321-328. doi:<https://doi.org/10.1016/j.jff.2017.09.006>

61. Bui TT, Nguyen KPT, Nguyen PPK, Le DT, Nguyen TLT. Anti-inflammatory and α-glucosidase inhibitory activities of chemical constituents from *Bruguiera parviflora* leaves. *J Chem*. 2022;doi:<https://doi.org/10.1155/2022/3049994>

62. Hlila MB, Majouli K, Ben Jannet H, Mastouri M, Aouni M, Selmi B. Antioxidant and anti alpha-glucosidase of luteolin and luteolin 7-O-glucoside isolated from *Scabiosa arenaria* Forssk. *J Coast Life Med*. 2017;5(7):317-320. doi:<https://doi.org/10.12980/jclm.5.2017J7-66>

63. Vu NK, Kim CS, Ha MT, et al. Antioxidant and antidiabetic activities of flavonoid derivatives from the outer skins of *Allium cepa* L. *J Agric Food Chem*. 2020;68(33):8797-8811. doi:<https://doi.org/10.1021/acs.jafc.0c02122>

64. Fan P, Terrier L, Hay AE, Marston A, Hostettmann K. Antioxidant and enzyme inhibition activities and chemical profiles of *Polygonum sachalinensis* F.Schmidt ex Maxim (Polygonaceae). *Fitoterapia*. 2010;81(2):124-131. doi:<https://doi.org/10.1016/j.fitote.2009.08.019>

65. Choi CI, Lee SR, Kim KH. Antioxidant and α-glucosidase inhibitory activities of constituents from *Euonymus alatus* twigs. *Ind Crops Prod*. 2015;76:1055-1060. doi:<https://doi.org/10.1016/j.indcrop.2015.08.031>

66. Yue Y, Chen Y, Geng S, Liang G, Liu B. Antioxidant and α-glucosidase inhibitory activities of fisetin. *Nat Prod Commun*. 2018;13(11):1489-1492. doi:<https://doi.org/10.1177/1934578x1801301119>

67. Choi CI, Eom HJ, Kim KH. Antioxidant and α-glucosidase inhibitory phenolic constituents of *Lactuca indica* L. *Russ J Bioorganic Chem*. 2016;42(3):310-315. doi:<https://doi.org/10.1134/S1068162016030079>

68. Wan C, Yuan T, Cirello AL, Seeram NP. Antioxidant and α-glucosidase inhibitory phenolics isolated from highbush blueberry flowers. *Food Chem*. 2012;135(3):1929-1937. doi:<https://doi.org/10.1016/j.foodchem.2012.06.056>

69. Wang Y, Zhai J, Yang D, et al. Antioxidant, anti-inflammatory, and antidiabetic activities of bioactive compounds from the fruits of *Livistona chinensis* based on network pharmacology prediction. *Oxid Med Cell Longev*. 2021;2021:7807046. doi:<https://doi.org/10.1155/2021/7807046>

70. Polbuppha I, Maneerat W, Sripisut T, et al. Antioxidant, cytotoxic and α-glucosidase inhibitory activities of compounds isolated from the twig extracts of *Maclura fruticosa*. *Nat Prod Commun*. 2017;12(7):1934578X1701200718. doi:<https://doi.org/10.1177/1934578X1701200718>

71. Supasuteekul C, Nonthitipong W, Tadtong S, Likhitwitayawuid K, Tengamnuay P, Sritularak B. Antioxidant, DNA damage protective, neuroprotective, and α-glucosidase inhibitory activities of a flavonoid glycoside from leaves of *Garcinia gracilis*. *Rev Bras Farmacogn*. 2016;26(3):312-320. doi:<https://doi.org/10.1016/j.bjp.2016.01.007>

72. Zhao J-Q, Wang Y-M, Yang Y-L, et al. Antioxidants and α-glucosidase inhibitors from “Liucha” (young leaves and shoots of *Sibiraea laevigata*). *Food Chem*. 2017;230:117-124. doi:<https://doi.org/10.1016/j.foodchem.2017.03.024>

73. Zhang L, Tu ZC, Yuan T, Wang H, Xie X, Fu ZF. Antioxidants and α-glucosidase inhibitors from *Ipomoea batatas* leaves identified by bioassay-guided approach and structure-activity relationships. *Food Chem*. 2016;208:61-7. doi:<https://doi.org/10.1016/j.foodchem.2016.03.079>

74. Anh LTT, Son NT, Van Tuyen N, et al. Antioxidative and α-glucosidase inhibitory constituents of *Polyscias guilfoylei*: experimental and computational assessments. *Mol Divers*. 2022;26(1):229-243. doi:<https://doi.org/10.1007/s11030-021-10206-6>

75. Sahnoun M, Saibi W, Brini F, Bejar S. Apigenin isolated from *A. americana* encodes Human and *Aspergillus oryzae* S2 α-amylase inhibitions: credible approach for antifungal and antidiabetic therapies. *J Food Sci Technol*. 2018;55(4):1489-1498. doi:<https://doi.org/10.1007/s13197-018-3065-6>

76. Ma J, Zhang X-L, Wang Y, Zheng J-Y, Wang C-Y, Shao C-L. Aspergivones A and B, two new flavones isolated from a gorgonian-derived *Aspergillus candidus* fungus. *Nat Prod Res*. 2017;31(1):32-36. doi:<https://doi.org/10.1080/14786419.2016.1207073>

77. Monteiro AO, Carvalho JL, da Silva HC, et al. *Bauhinia pulchella*: chemical constituents, antioxidant and alpha-glucosidase inhibitory activities. *Nat Prod Res*. 2022;36(6):1604-1609. doi:<https://doi.org/10.1080/14786419.2021.1887176>

78. Song M, Xiao T, Wu Q-S, et al. Biflavonoids from the twigs and leaves of *Cephalotaxus oliveri* Mast. and their α-glucosidase inhibitory activity. *Nat Prod Res*. 2022;36(12):3085-3094. doi:<https://doi.org/10.1080/14786419.2021.1958328>

79. Tian JL, Si X, Wang YH, et al. Bioactive flavonoids from *Rubus corchorifolius* inhibit α-glucosidase and α-amylase to improve postprandial hyperglycemia. *Food Chem*. 2021;341:128149. doi:<https://doi.org/10.1016/j.foodchem.2020.128149>

80. Jing P, Xiaomin Y, Shujuan Z, et al. Bioactive phenolics from mango leaves (*Mangifera indica* L.). *Ind Crops Prod*. 2018;111:400-406. doi:<https://doi.org/10.1016/j.indcrop.2017.10.057>

81. Nickavar B, Abolhasani L. Bioactivity-guided separation of an α-amylase inhibitor flavonoid from *Salvia virgata*. *Iran J Pharm Res*. 2013;12(1):57-61.

82. Li S, Wang R, Hu X, Li C, Wang L. Bio-affinity ultra-filtration combined with HPLC-ESI-qTOF-MS/MS for screening potential α-glucosidase inhibitors from *Cerasus humilis* (Bge.) Sok. leaf-tea and in silico analysis. *Food Chem*. 2022;373:131528. doi:<https://doi.org/10.1016/j.foodchem.2021.131528>

83. Renda G, Özel A, Barut B, et al. Bioassay guided isolation of active compounds from *Alchemilla barbatiflora* Juz. *Rec Nat Prod*. 2017;12:76-85. doi:<https://doi.org/10.25135/rnp.07.17.07.117>

84. Jibril S, Sirat HM, Basar N. Bioassay-guided isolation of antioxidants and alpha-glucosidase inhibitors from the root of *Cassia sieberiana* D.C. (Fabaceae). *Rec Nat Prod*. 2017;11(4):406-410.

85. Nickavar B, Amin G. Bioassay-guided separation of an alpha-amylase inhibitor anthocyanin from *Vaccinium arctostaphylos* berries. *Z Naturforsch C J Biosci*. 2010;65(9):567-70. doi:<https://doi.org/10.1515/znc-2010-9-1006>

86. Khalid MF, Rehman K, Irshad K, Chohan TA, Akash MSH. Biochemical investigation of inibitory activities of plant-derived bioactive compounds against carbohydrate and glucagon-like peptide-1 metabolizing enzymes. *Dose-Response*. 2022;(2)doi:<https://doi.org/10.1177/15593258221093275>

87. Zhou H, Li HM, Du YM, et al. C-geranylated flavanones from YingDe black tea and their antioxidant and α-glucosidase inhibition activities. *Food Chem*. 2017;235:227-233. doi:<https://doi.org/10.1016/j.foodchem.2017.05.034>

88. Ha MT, Seong SH, Nguyen TD, et al. Chalcone derivatives from the root bark of *Morus alba* L. act as inhibitors of PTP1B and α-glucosidase. *Phytochemistry*. 2018;155:114-125. doi:<https://doi.org/10.1016/j.phytochem.2018.08.001>

89. Tajudeen Bale A, Mohammed Khan K, Salar U, et al. Chalcones and bis-chalcones: As potential α-amylase inhibitors; synthesis, in vitro screening, and molecular modelling studies. *Bioorg Chem*. 2018;79:179-189. doi:<https://doi.org/10.1016/j.bioorg.2018.05.003>

90. Ali M, Khan M, Zaman K, et al. Chalcones: As potent α-amylase enzyme inhibitors; synthesis, in vitro, and in silico studies. *Med Chem*. 2020;17(8):903-912. doi:<https://doi.org/10.2174/1573406416666200611103039>

91. Fidelis QC, Faraone I, Russo D, et al. Chemical and biological insights of *Ouratea hexasperma* (A. St.-Hil.) Baill.: a source of bioactive compounds with multifunctional properties. *Nat Prod Res*. 2019;33(10):1500-1503. doi:<https://doi.org/10.1080/14786419.2017.1419227>

92. Tian X, Guo S, Zhang S, et al. Chemical characterization of main bioactive constituents in *Paeonia ostii* seed meal and GC-MS analysis of seed oil. *J Food Biochem*. 2020;44(1):e13088. doi:<https://doi.org/10.1111/jfbc.13088>

93. Uddin S, Brooks PR, Tran TD. Chemical characterization, α-glucosidase, α-amylase and lipase inhibitory properties of the Australian honey bee propolis. *Foods*. 2022;11(13):1964. doi:<https://doi.org/10.3390/foods11131964>

94. Costa Silva TD, Justino AB, Prado DG, et al. Chemical composition, antioxidant activity and inhibitory capacity of α-amylase, α-glucosidase, lipase and non-enzymatic glycation, in vitro, of the leaves of *Cassia bakeriana* Craib. *Ind Crops Prod*. 2019;140:111641. doi:<https://doi.org/10.1016/j.indcrop.2019.111641>

95. Adhikari-Devkota A, Elbashir SMI, Watanabe T, Devkota HP. Chemical constituents from the flowers of *Satsuma mandarin* and their free radical scavenging and alpha-glucosidase inhibitory activities. *Nat Prod Res*. 2019;33(11):1670-1673. doi:<https://doi.org/10.1080/14786419.2018.1425856>

96. Paul S, Zhang X, Yang Y, Geng C. Chemical constituents from Turnip and their effects on alpha-glucosidase. *Phyton - Int J Exp Bot*. 2020;89(1):131-136. doi:<https://doi.org/10.32604/phyton.2020.08328>

97. Jeong SY, Nguyen PH, Zhao BT, et al. Chemical constituents of *Euonymus alatus* (Thunb.) Sieb. and their PTP1B and alpha-glucosidase inhibitory activities. *Phytother Res*. 2015;29(10):1540-1548. doi:<https://doi.org/10.1002/ptr.5411>

98. Linh NTT, Thuy TT, Tam NT, et al. Chemical constituents of *Impatiens chapaensis* Tard. and their α-glucosidase inhibition activities. *Nat Prod Res*. 2022;36(12):3229-3233. doi:<https://doi.org/10.1080/14786419.2021.1956923>

99. Abdullah NH, Salim F, Ahmad R. Chemical constituents of Malaysian *U. cordata* var. *ferruginea* and their in vitro alpha-glucosidase inhibitory activities. *Molecules*. 2016;21(5):525. doi:<https://doi.org/10.3390/molecules21050525>

100. Lee HE, Kim JA, Whang WK. Chemical constituents of *Smilax china* L. stems and their inhibitory activities against glycation, aldose reductase, alpha-glucosidase, and lipase. *Molecules*. 2017;22(3):451. doi:<https://doi.org/10.3390/molecules22030451>

101. Kuroda M, Iwabuchi K, Mimaki Y. Chemical constituents of the aerial parts of *Scutellaria lateriflora* and their alpha-glucosidase inhibitory activities. *Nat Prod Commun*. 2012;7(4):471-474. doi:<https://doi.org/10.1177/1934578x1200700413>

102. Lam S-H, Chen J-M, Tsai S-F, Lee S-S. Chemical investigation on the root bark of *Bombax malabarica*. *Fitoterapia*. 2019;139:104376. doi:<https://doi.org/10.1016/j.fitote.2019.104376>

103. Huang Q, Chen JJ, Pan Y, et al. Chemical profiling and antidiabetic potency of *Paeonia delavayi*: Comparison between different parts and constituents. *J Pharm Biomed Anal*. 2021;198:113998. doi:<https://doi.org/10.1016/j.jpba.2021.113998>

104. Xu L, Huang T, Huang C, Wu C, Jia A, Hu X. Chiral separation, absolute configuration, and bioactivity of two pairs of flavonoid enantiomers from *Morus nigra*. *Phytochemistry*. 2019;163:33-37. doi:<https://doi.org/10.1016/j.phytochem.2019.03.029>

105. Zeng Y-R, Wang L-P, Hu Z-X, et al. Chromanopyrones and a flavone from *Hypericum monogynum*. *Fitoterapia*. 2018;125:59-64. doi:<https://doi.org/10.1016/j.fitote.2017.12.013>

106. Qurtam AA, Mechchate H, Es-Safi I, et al. Citrus flavanone narirutin, in vitro and in silico mechanistic antidiabetic potential. *Pharmaceutics*. 2021;13(11):1818. doi:<https://doi.org/10.3390/pharmaceutics13111818>

107. Sahnoun M, Trabelsi S, Bejar S. Citrus flavonoids collectively dominate the α-amylase and α-glucosidase inhibitions. *Biologia*. 2017;72(7):764-773. doi:<https://doi.org/10.1515/biolog-2017-0091>

108. Li YQ, Zhou FC, Gao F, Bian JS, Shan F. Comparative evaluation of quercetin, isoquercetin and rutin as inhibitors of alpha-glucosidase. *J Agric Food Chem*. 2009;57(24):11463-11468. doi:<https://doi.org/10.1021/jf903083h>

109. Jia Y, Ma Y, Cheng G, Zhang Y, Cai S. Comparative study of dietary flavonoids with different structures as alpha-glucosidase inhibitors and insulin sensitizers. *J Agric Food Chem*. 2019;67(37):10521-10533. doi:<https://doi.org/10.1021/acs.jafc.9b04943>

110. Wang L, Wang L, Wang T, et al. Comparison of quercetin and rutin inhibitory influence on *Tartary buckwheat* starch digestion in vitro and their differences in binding sites with the digestive enzyme. *Food Chem*. 2022;367:130762. doi:<https://doi.org/10.1016/j.foodchem.2021.130762>

111. Olennikov DN, Kashchenko NI. Componential profile and amylase inhibiting activity of phenolic compounds from *Calendula officinalis* L. leaves. *Sci World J*. 2014;2014:654193. doi:<https://doi.org/10.1155/2014/654193>

112. Lyu Q, Kuo T-H, Sun C, Chen K, Hsu C-C, Li X. Comprehensive structural characterization of phenolics in litchi pulp using tandem mass spectral molecular networking. *Food Chem*. 2019;282:9-17. doi:<https://doi.org/10.1016/j.foodchem.2019.01.001>

113. Li G, Wang G, Tong Y, et al. Concise synthesis and antidiabetic activity of natural flavonoid glycosides, oroxins C and D, isolated from the seeds of *Oroxylum indium*. *J Chem Res*. 2020;45doi:<https://doi.org/10.1177/1747519820927966>

114. Wang XL, Jiao FR, Yu M, et al. Constituents with potent alpha-glucosidase inhibitory activity from *Pueraria lobata* (Willd.) ohwi. *Bioorg Med Chem Lett*. 2017;27(9):1993-1998. doi:<https://doi.org/10.1016/j.bmcl.2017.03.013>

115. Mo Q-G, Zhou G, Zhu W-D, Ge L-L, Wang Y-W. Coumaroyl and feruloyl flavonoid glycosides from the male flowers of *Ginkgo biloba* L. and their inhibitory activity against α-glucosidase. *Nat Prod Res*. 2022;36(17):4365-4372. doi:<https://doi.org/10.1080/14786419.2021.1993216>

116. Nazir N, Zahoor M, Ullah R, Ezzeldin E, Mostafa GAE. Curative effect of catechin isolated from *Elaeagnus umbellata* Thunb. berries for diabetes and related complications in streptozotocin-induced diabetic rats model. *Molecules*. 2020;26(1):137. doi:<https://doi.org/10.3390/molecules26010137>

117. Oueslati MH, Bouajila J, Guetat A, Al-Gamdi F, Hichri F. Cytotoxic, alpha-glucosidase, and antioxidant activities of flavonoid glycosides isolated from flowers of *Lotus lanuginosus* Vent. (Fabaceae). *Pharmacogn Mag*. 2020;16(68):22-27. doi:<https://doi.org/10.4103/pm.pm_232_19>

118. Li Q, Zhang X, Cao J, et al. Depside derivatives with anti-hepatic fibrosis and anti-diabetic activities from *Impatiens balsamina* L. flowers. *Fitoterapia*. 2015;105:234-239. doi:<https://doi.org/10.1016/j.fitote.2015.07.007>

119. Ashraf J, Mughal EU, Sadiq A, et al. Design and synthesis of new flavonols as dual ɑ-amylase and ɑ-glucosidase inhibitors: Structure-activity relationship, drug-likeness, in vitro and in silico studies. *J Mol Struct*. 2020;1218:128458. doi:<https://doi.org/10.1016/j.molstruc.2020.128458>

120. Yang JR, Luo JG, Kong LY. Determination of α-glucosidase inhibitors from *ScutScutellaria baicalensis* using liquid chromatography with quadrupole time of flight tandem mass spectrometry coupled with centrifugal ultrafiltration. *Chin J Nat Med*. 2015;13(3):208-214. doi:<https://doi.org/10.1016/S1875-5364(15)30006-6>

121. Nguyen DH, Le DD, Ma ES, Min BS, Woo MH. Development and validation of an HPLC-PDA method for quantitation of ten marker compounds from *Eclipta prostrata* (L.) and evaluation of their protein tyrosine phosphatase 1B, alpha-glucosidase, and acetylcholinesterase inhibitory activities. *Nat Prod Sci*. 2020;26(4):326-333. doi:<https://doi.org/10.20307/nps.2020.26.4.326>

122. He X-F, Chen J-J, Li T-Z, Hu J, Zhang X-M, Geng C-A. Diarylheptanoid-chalcone hybrids with PTP1B and α-glucosidase dual inhibition from *Alpinia katsumadai*. *Bioorg Chem*. 2021;108:104683. doi:<https://doi.org/10.1016/j.bioorg.2021.104683>

123. Bo-wei Z, Xia L, Wen-long S, et al. Dietary flavonoids and acarbose synergistically inhibit alpha-glucosidase and lower postprandial blood glucose. *J Agric Food Chem*. 2017;65(38):8319-8330. doi:<https://doi.org/10.1021/acs.jafc.7b02531>

124. Lv Q, Lin Y, Tan Z, et al. Dihydrochalcone-derived polyphenols from tea crab apple (*Malus hupehensis*) and their inhibitory effects on α-glucosidase in vitro. *Food Funct*. 2019;10(5):2881-2887. doi:<https://doi.org/10.1039/c9fo00229d>

125. Sohretoglu D, Sari S, Barut B, Ozel A. Discovery of potent alpha-glucosidase inhibitor flavonols: Insights into mechanism of action through inhibition kinetics and docking simulations. *Bioorg Chem*. 2018;79:257-264. doi:<https://doi.org/10.1016/j.bioorg.2018.05.010>

126. Su ZR, Fan SY, Shi WG, Zhong BH. Discovery of xanthine oxidase inhibitors and/or α-glucosidase inhibitors by carboxyalkyl derivatization based on the flavonoid of apigenin. *Bioorg Med Chem Lett*. 2015;25(14):2778-81. doi:<https://doi.org/10.1016/j.bmcl.2015.05.016>

127. Ray S, Samanta T, Mitra A, De B. Effect of extracts and components of black tea on the activity of beta-glucuronidase, lipase, alpha-amylase, alpha-glucosidase: An in vitro study. *Curr Nutr Food Sci*. 2014;10(3):181-186. doi:<https://doi.org/10.2174/1573401310666140529205646>

128. Giang Thanh Thi H, Kase ET, Wangensteen H, Barsett H. Effect of phenolic compounds from Elderflowers on glucose- and fatty acid uptake in human myotubes and HepG2-cells. *Molecules*. 2017;22(1):90. doi:<https://doi.org/10.3390/molecules22010090>

129. Zhou Y, Jiang Q, Ma S, Zhou X. Effect of quercetin on the in vitro *Tartary buckwheat* starch digestibility. *Int J Biol Macromol*. 2021;183:818-830. doi:<https://doi.org/10.1016/j.ijbiomac.2021.05.013>

130. Krishna MS, Joy B, Sundaresan A. Effect on oxidative stress, glucose uptake level and lipid droplet content by Apigenin 7, 4'-dimethyl ether isolated from *Piper longum* L. *J Food Sci Technol*. 2015;52(6):3561-70. doi:<https://doi.org/10.1007/s13197-014-1387-6>

131. Su J, Tang Z. Effects of (-)-epigallocatechin gallate and quercetin on the activity and structure of alpha-amylase. *Trop J Pharm Res*. 2019;18(3):585-590. doi:<https://doi.org/10.4314/tjpr.v18i3.20>

132. Nickavar B, Amin G. Enzyme assay guided isolation of an α-amylase inhibitor flavonoid from *Vaccinium arctostaphylos* leaves. *Iran J Pharm Res*. 2011;10(4):849-53.

133. Zhang Y, Gu D, He S, et al. Enzyme reaction-guided identification of active components from the flowers of: *Sophora japonica* var. *violacea*. *Food Funct*. 2020;11(5):4356-4362. doi:<https://doi.org/10.1039/d0fo00625d>

134. Phan MAT, Wang J, Tang J, Lee YZ, Ng K. Evaluation of α-glucosidase inhibition potential of some flavonoids from *Epimedium brevicornum*. *LWT*. 2013;53(2):492-498. doi:<https://doi.org/10.1016/j.lwt.2013.04.002>

135. Huang D, Jiang Y, Chen W, Yao F, Huang G, Sun L. Evaluation of hypoglycemic effects of polyphenols and extracts from *Penthorum chinense*. *J Ethnopharmacol*. 2015;163:256-263. doi:<https://doi.org/10.1016/j.jep.2015.01.014>

136. Numonov S, Edirs S, Bobakulov K, et al. Evaluation of the antidiabetic activity and chemical composition of *Geranium collinum* root extracts—computational and experimental investigations. *Molecules*. 2017;22(6):983. doi:<https://doi.org/10.3390/molecules22060983>

137. Dubey K, Dubey R, Gupta R, Gupta A. Exploration of diosmin to control diabetes and its complications-an in vitro and in silico approach. *Curr Comput Aided Drug Des*. 2021;17(2):307-313. doi:<https://doi.org/10.2174/1573409916666200324135734>

138. Li M, Bao X, Zhang X, et al. Exploring the phytochemicals and inhibitory effects against α-glucosidase and dipeptidyl peptidase-IV in Chinese pickled chili pepper: Insights into mechanisms by molecular docking analysis. *LWT*. 2022;162:113467. doi:<https://doi.org/10.1016/j.lwt.2022.113467>

139. Tang H, Huang L, Sun C, Zhao D. Exploring the structure-activity relationship and interaction mechanism of flavonoids and alpha-glucosidase based on experimental analysis and molecular docking studies. *Food Funct*. 2020;11(4):3332-3350. doi:<https://doi.org/10.1039/c9fo02806d>

140. Limin W, Yunsen Z, Johnpaul IA, et al. Exploring two types of prenylated bitter compounds from hop plant (*Humulus lupulus* L.) against alpha-glucosidase in vitro and in silico. *Food Chem*. 2022;370:130979-130979. doi:<https://doi.org/10.1016/j.foodchem.2021.130979>

141. Jiang P, Zhao Y, Xiong J, et al. Extraction, purification, and biological activities of flavonoids from branches and leaves of *Taxus cuspidata* S. et Z. *BioResources*. 2021;16(2):2655-2682. doi:<https://doi.org/10.15376/biores.16.2.2655-2682>

142. Terfassi S, Dauvergne X, Cérantola S, et al. First report on phytochemical investigation, antioxidant and antidiabetic activities of *Helianthemum getulum*. *Nat Prod Res*. 2022;36(11):2806-2813. doi:<https://doi.org/10.1080/14786419.2021.1928664>

143. Wang X, Liu Q, Zhu H, et al. Flavanols from the *Camellia sinensis* var. *assamica* and their hypoglycemic and hypolipidemic activities. *Acta Pharm Sin B*. 2017;7(3):342-346. doi:<https://doi.org/10.1016/j.apsb.2016.12.007>

144. Dhameja M, Kumar H, Kurella S, Uma A, Gupta P. Flavone-1,2,3-triazole derivatives as potential α-glucosidase inhibitors: Synthesis, enzyme inhibition, kinetic analysis and molecular docking study. *Bioorg Chem*. 2022;127:106028. doi:<https://doi.org/10.1016/j.bioorg.2022.106028>

145. Dao TBN, Nguyen TMT, Nguyen VQ, et al. Flavones from *Combretum quadrangulare* growing in Vietnam and their alpha-glucosidase inhibitory activity. *Molecules*. 2021;26(9):2531. doi:<https://doi.org/10.3390/molecules26092531>

146. Li R, Wang Q, Zhao M, Yang P, Hu X, Ouyang D. Flavonoid glycosides from seeds of *Hippophae rhamnoides* subsp. *Sinensis* with alpha-glucosidase inhibition activity. *Fitoterapia*. 2019;137:104248. doi:<https://doi.org/10.1016/j.fitote.2019.104248>

147. Lin H-C, Tsai S-F, Lee S-S. Flavonoid glycosides from the leaves of *Machilus philippinensis*. *J Chin Chem Soc*. 2011;58(4):555-562. doi:<https://doi.org/10.1002/jccs.201190020>

148. Zhang X, Liu Z, Bi X, Liu J, Li W, Zhao Y. Flavonoids and its derivatives from *Callistephus chinensis* flowers and their inhibitory activities against α-glucosidase. *EXCLI J*. 2013;12:956-966.

149. Praparatana R, Maliyam P, Barrows LR, Puttarak P. Flavonoids and phenols, the potential anti-diabetic compounds from *Bauhinia strychnifolia* Craib. stem. *Molecules*. 2022;27(8):2393. doi:<https://doi.org/10.3390/molecules27082393>

150. Barber E, Houghton MJ, Williamson G. Flavonoids as human intestinal α-glucosidase inhibitors. *Foods*. 2021;10(8):1939. doi:<https://doi.org/10.3390/foods10081939>

151. Lo Piparo E, Scheib H, Frei N, Williamson G, Grigorov M, Chou CJ. Flavonoids for controlling starch digestion: Structural requirements for inhibiting human alpha-amylase. *J Med Chem*. 2008;51(12):3555-3561. doi:<https://doi.org/10.1021/jm800115x>

152. Abbasi MA, Hussain G, Aziz ur R, Ahmad VU. Flavonoids from *Rhynchosia pseudo-cajan* as suitable alpha-glucosidase inhibitors and free radical scavengers. *Int Res J Pharm*. 2014;5(8):636-641. doi:<https://doi.org/10.7897/2230-8407.0508130>

153. Asghari B, Salehi P, Sonboli A, Ebrahimi SN. Flavonoids from *Salvia chloroleuca* with alpha-amylase and alpha-glucosidase inhibitory effect. *Iran J Pharm Res*. 2015;14(2):609-615.

154. Devkota HP, Kurizaki A, Tsushiro K, et al. Flavonoids from the leaves and twigs of *Lindera sericea* (Seibold et Zucc.) Blume var. *sericea* (Lauraceae) from Japan and their bioactivities. *Funct Foods Health Dis*. 2021;11(1):34-43. doi:<https://doi.org/10.31989/ffhd.v11i1.769>

155. Manaharan T, Appleton D, Cheng HM, Palanisamy UD. Flavonoids isolated from *Syzygium aqueum* leaf extract as potential antihyperglycaemic agents. *Food Chem*. 2012;132(4):1802-1807. doi:<https://doi.org/10.1016/j.foodchem.2011.11.147>

156. Hong HC, Li SL, Zhang XQ, Ye WC, Zhang QW. Flavonoids with alpha-glucosidase inhibitory activities and their contents in the leaves of *Morus atropurpurea*. *Chin Med*. 2013;8(1):19. doi:<https://doi.org/10.1186/1749-8546-8-19>

157. Lin YS, Lee SS. Flavonol glycosides with α-Glucosidase inhibitory activities and new flavone C-diosides from the leaves of *Machilus konishii*. *Helv Chim Acta*. 2014;97(12):1672-1682. doi:<https://doi.org/10.1002/hlca.201400081>

158. Akoro SM, Aiyelaagbe OO, Onocha PA, Gloer JB. Gakolanone: a new benzophenone derivative from *Garcinia kola* Heckel stem-bark. *Nat Prod Res*. 2020;34(2):241-250. doi:<https://doi.org/10.1080/14786419.2018.1528583>

159. Kiruthiga N, Saravanan G, Selvinthanuja C, Srinivasan K, Sivakumar T. Glycolytic inhibition and antidiabetic activity on synthesised flavanone scaffolds with computer aided drug design tool. *Lett Drug Des Discov*. 2021;18(6):574-592. doi:<https://doi.org/10.2174/1570180817999201209204523>

160. Shan-Shan Z, Niu-Niu Z, Sen G, et al. Glycosides and flavonoids from the extract of *Pueraria thomsonii* Benth leaf alleviate type 2 diabetes in high-fat diet plus streptozotocin-induced mice by modulating the gut microbiota. *Food Funct*. 2022;13(7):3931-3945. doi:<https://doi.org/10.1039/d1fo04170c>

161. Sichaem J, Tip-pyang S, Lugsanangarm K, Jutakanoke R. Highly potent glucosidase inhibitors from *Pterocarpus indicus* and molecular docking studies. *Songklanakarin J Sci Technol*. 2020;42(2):359-364. doi:<https://doi.org/10.4172/2169-0138-C3-029>

162. Silva EL, Lobo JFR, Vinther JM, Borges RM, Staerk D. High-resolution alpha-glucosidase inhibition profiling combined with HPLC-HRMS-SPE-NMR for identification of anti-diabetic compounds in *Eremanthus crotonoides* (Asteraceae). *Molecules*. 2016;21(6):782. doi:<https://doi.org/10.3390/molecules21060782>

163. Jeong GH, Kim TH. Hydroxymethylation of rutin induced by radiolysis as novel α-glucosidase inhibitors. *Chem Pharm Bull (Tokyo)*. 2017;65(7):678-682. doi:<https://doi.org/10.1248/cpb.c17-00190>

164. Sengupta S, Mukherjee A, Goswami R, Basu S. Hypoglycemic activity of the antioxidant saponarin, characterized as alpha-glucosidase inhibitor present in *Tinospora cordifolia*. *J Enzyme Inhib Med Chem*. 2009;24(3):684-90. doi:<https://doi.org/10.1080/14756360802333075>

165. Zhang Y, Xiao Z, Zhang X, Sun H. Hypoglycemic and hypolipidemic dual activities of extracts and flavonoids from Desmodium caudatum and an efficient synthesis of the most potent 8-prenylquercetin. *Fitoterapia*. 2022;156:105083. doi:<https://doi.org/10.1016/j.fitote.2021.105083>

166. Deutschlander MS, Lall N, Van De Venter M, Hussein AA. Hypoglycemic evaluation of a new triterpene and other compounds isolated from *Euclea undulata* Thunb. var. *Myrtina* (Ebenaceae) root bark. *J Ethnopharmacol*. 2011;133(3):1091-1095. doi:<https://doi.org/10.1016/j.jep.2010.11.038>

167. Milella L, Milazzo S, De Leo M, et al. α-glucosidase and α-amylase Inhibitors from *Arcytophyllum thymifolium*. *J Nat Prod*. 2016;79(8):2104-2112. doi:<https://doi.org/10.1021/acs.jnatprod.6b00484>

168. Jin DX, He JF, Zhang KQ, Luo XG, Zhang TC. α-Glucosidase inhibition action of major flavonoids identified from *Hypericum attenuatum* Choisy and their synergistic effects. *Chem Biodivers*. 2021;18(10):e2100244. doi:<https://doi.org/10.1002/cbdv.202100244>

169. Chen J, Wu Y, Zou J, Gao K. α-Glucosidase inhibition and antihyperglycemic activity of flavonoids from *Ampelopsis grossedentata* and the flavonoid derivatives. *Bioorg Med Chem*. 2016;24(7):1488-94. doi:<https://doi.org/10.1016/j.bmc.2016.02.018>

170. Proença C, Freitas M, Ribeiro D, et al. α-Glucosidase inhibition by flavonoids: an in vitro and in silico structure-activity relationship study. *J Enzyme Inhib Med Chem*. 2017;32(1):1216-1228. doi:<https://doi.org/10.1080/14756366.2017.1368503>

171. Yan J, Zhang G, Pan J, Wang Y. α-Glucosidase inhibition by luteolin: kinetics, interaction and molecular docking. *Int J Biol Macromol*. 2014;64:213-23. doi:<https://doi.org/10.1016/j.ijbiomac.2013.12.007>

172. Kim JH, Cho CW, Kim HY, et al. α-Glucosidase inhibition by prenylated and lavandulyl compounds from *Sophora flavescens* roots and in silico analysis. *Int J Biol Macromol*. 2017;102:960-969. doi:<https://doi.org/10.1016/j.ijbiomac.2017.04.092>

173. Liu Y, Zhan L, Xu C, et al. α-Glucosidase inhibitors from Chinese bayberry (*Morella rubra* Sieb. et Zucc.) fruit: Molecular docking and interaction mechanism of flavonols with different B-ring hydroxylations. *RSC Adv*. 2020;10(49):29347-29361. doi:<https://doi.org/10.1039/d0ra05015f>

174. Le T-K-D, Danova A, Aree T, et al. α-Glucosidase inhibitors from the stems of *Knema globularia*. *J Nat Prod*. 2022;85(4):776-786. doi:<https://doi.org/10.1021/acs.jnatprod.1c00765>

175. Flores-Bocanegra L, Pérez-Vásquez A, Torres-Piedra M, Bye R, Linares E, Mata R. α-Glucosidase inhibitors from *Vauquelinia corymbosa*. *Molecules*. 2015;20(8):15330-15342. doi:<https://doi.org/10.3390/molecules200815330>

176. Tasnuva ST, Qamar UA, Ghafoor K, et al. α-glucosidase inhibitors isolated from *Mimosa pudica* L. *Nat Prod Res*. 2019;33(10):1495-1499. doi:<https://doi.org/10.1080/14786419.2017.1419224>

177. Hou Z-W, Chen C-H, Ke J-P, et al. α-glucosidase inhibitory activities and the interaction mechanism of novel spiro-flavoalkaloids from YingDe green tea. *J Agric Food Chem*. 2022;70(1):136-148. doi:<https://doi.org/10.1021/acs.jafc.1c06106>

178. Dendup T, Prachyawarakorn V, Pansanit A, Mahidol C, Ruchirawat S, Kittakoop P. α-Glucosidase inhibitory activities of isoflavanones, isoflavones, and pterocarpans from *Mucuna pruriens*. *Planta Med*. 2014;80(7):604-8. doi:<https://doi.org/10.1055/s-0034-1368427>

179. Suthiphasilp V, Maneerat T, Andersen RJ, Patrick BO, Pyne SG, Laphookhieo S. a-glucosidase inhibitory activity of compounds isolated from the twig and leaf extracts of *Desmos dumosus*. *Heliyon*. 2021;7(2):e06180. doi:<https://doi.org/10.1016/j.heliyon.2021.e06180>

180. Chen YG, Li P, Li P, et al. α-glucosidase inhibitory effect and simultaneous quantification of three major flavonoid glycosides in *Microctis folium*. *Molecules*. 2013;18(4):4221-4232. doi:<https://doi.org/10.3390/molecules18044221>

181. Chen JG, Wu SF, Zhang QF, Yin ZP, Zhang L. α-Glucosidase inhibitory effect of anthocyanins from *Cinnamomum camphora* fruit: Inhibition kinetics and mechanistic insights through in vitro and in silico studies. *Int J Biol Macromol*. 2020;143:696-703. doi:<https://doi.org/10.1016/j.ijbiomac.2019.09.091>

182. Şöhretoğlu D, Sari S, Özel A, Barut B. α-Glucosidase inhibitory effect of *Potentilla astracanica* and some isoflavones: Inhibition kinetics and mechanistic insights through in vitro and in silico studies. *Int J Biol Macromol*. 2017;105:1062-1070. doi:<https://doi.org/10.1016/j.ijbiomac.2017.07.132>

183. Renda G, Sari S, Barut B, et al. α-Glucosidase inhibitory effects of polyphenols from *Geranium asphodeloides*: Inhibition kinetics and mechanistic insights through in vitro and in silico studies. *Bioorg Chem*. 2018;81:545-552. doi:<https://doi.org/10.1016/j.bioorg.2018.09.009>

184. Meesakul P, Richardson C, Pyne SG, Laphookhieo S. α-Glucosidase inhibitory flavonoids and oxepinones from the leaf and twig extracts of *Desmos cochinchinensis*. *J Nat Prod*. 2019;82(4):741-747. doi:<https://doi.org/10.1021/acs.jnatprod.8b00581>

185. López-Angulo G, Miranda-Soto V, López-Valenzuela JA, et al. α-Glucosidase inhibitory phenolics from *Echeveria subrigida* (B. L. Rob & Seaton) leaves. *Nat Prod Res*. 2022;36(4):1058-1061. doi:<https://doi.org/10.1080/14786419.2020.1844695>

186. Ye R, Fan YH, Ma CM. Identification and enrichment of alpha-glucosidase-inhibiting dihydrostilbene and flavonoids from *Glycyrrhiza uralensis* leaves. *J Agric Food Chem*. 2017;65(2):510-515. doi:<https://doi.org/10.1021/acs.jafc.6b04155>

187. Duong TH, Nguyen HT, Nguyen CH, et al. Identification of highly potent alpha-glucosidase inhibitors from *Artocarpus integer* and molecular docking studies. *Chem Biodivers*. 2021;18(12):e2100499. doi:<https://doi.org/10.1002/cbdv.202100499>

188. Monzón Daza G, Meneses Macías C, Forero AM, et al. Identification of α-amylase and α-glucosidase inhibitors and ligularoside A, a new triterpenoid saponin from *Passiflora ligularis* Juss (Sweet Granadilla) Leaves, by a nuclear magnetic resonance-based metabolomic study. *J Agric Food Chem*. 2021;69(9):2919-2931. doi:<https://doi.org/10.1021/acs.jafc.0c07850>

189. Assefa ST, Yang EY, Asamenew G, Kim HW, Cho MC, Lee J. Identification of α-glucosidase inhibitors from leaf extract of pepper (*Capsicum* spp.) through metabolomic analysis. *Metabolites*. 2021;11(10):649. doi:<https://doi.org/10.3390/metabo11100649>

190. Fang HL, Liu ML, Li SY, et al. Identification, potency evaluation, and mechanism clarification of alpha-glucosidase inhibitors from tender leaves of *Lithocarpus polystachyus* Rehd. *Food Chem*. 2022;371:131128. doi:<https://doi.org/10.1016/j.foodchem.2021.131128>

191. Tao Y, Chen Z, Zhang Y, Wang Y, Cheng Y. Immobilized magnetic beads based multi-target affinity selection coupled with high performance liquid chromatography-mass spectrometry for screening anti-diabetic compounds from a Chinese medicine "Tang-Zhi-Qing". *J Pharm Biomed Anal*. 2013;78:190-201. doi:<https://doi.org/10.1016/j.jpba.2013.02.024>

192. Vonia S, Hartati R, Insanu M. In vitro alpha-glucosidase inhibitory activity and the isolation of luteolin from the flower of *Gymnanthemum amygdalinum* (Delile) Sch. Bip ex Walp. *Molecules*. 2022;27(7):2132. doi:<https://doi.org/10.3390/molecules27072132>

193. Yoon KD, Lee JY, Kim TY, et al. In vitro and in vivo anti-hyperglycemic activities of taxifolin and its derivatives isolated from pigmented rice (*Oryzae sativa* L. cv. *Superhongmi*). *J Agric Food Chem*. 2020;68(3):742-750. doi:<https://doi.org/10.1021/acs.jafc.9b04962>

194. Su H, Ruan YT, Li Y, Chen JG, Yin ZP, Zhang QF. In vitro and in vivo inhibitory activity of taxifolin on three digestive enzymes. *Int J Biol Macromol*. 2020;150:31-37. doi:<https://doi.org/10.1016/j.ijbiomac.2020.02.027>

195. Wang W, Xu H, Chen H, Tai K, Liu F, Gao Y. In vitro antioxidant, anti-diabetic and antilipemic potentials of quercetagetin extracted from marigold (*Tagetes erecta* L.) inflorescence residues. *J Food Sci Technol*. 2016;53(6):2614-24. doi:<https://doi.org/10.1007/s13197-016-2228-6>

196. Akkarachiyasit S, Yibchok-Anun S, Wacharasindhu S, Adisakwattana S. In vitro inhibitory effects of cyandin-3-rutinoside on pancreatic α-amylase and its combined effect with acarbose. *Molecules*. 2011;16(3):2075-83. doi:<https://doi.org/10.3390/molecules16032075>

197. Hu YC, Luo YD, Li L, Joshi MK, Lu YH. In vitro investigation of 2',4'-dihydroxy-6'-methoxy-3',5'-dimethylchalcone for glycemic control. *J Agric Food Chem*. 2012;60(42):10683-8. doi:<https://doi.org/10.1021/jf303078r>

198. Lian-Xin P, Li-Juan W, Qian Y, et al. In vitro potential of flavonoids from tartary buckwheat on antioxidants activity and starch digestibility. *Int J Food Sci Technol*. 2019;54(6):2209-2218. doi:<https://doi.org/10.1111/ijfs.14131>

199. Liao G, Fan J, Ludwig-Radtke L, Backhaus K, Li S-M. Increasing structural diversity of natural products by Michael addition with ortho-quinone methide as the acceptor. *J Org Chem*. 2020;85(2):1298-1307. doi:<https://doi.org/10.1021/acs.joc.9b02971>

200. Yang J, Li H, Wang X, Zhang C, Feng G, Peng X. Inhibition mechanism of alpha-amylase/alpha-glucosidase by silibinin, its synergism with acarbose, and the effect of milk proteins. *J Agric Food Chem*. 2021;69(36):10515-10526. doi:<https://doi.org/10.1021/acs.jafc.1c01765>

201. Kwon RH, Thaku N, Timalsina B, Park SE, Choi JS, Jung HA. Inhibition mechanism of components isolated from *Morus alba* branches on diabetes and diabetic complications via experimental and molecular docking analyses. *Antioxidants*. 2022;11(2):383. doi:<https://doi.org/10.3390/antiox11020383>

202. Fang H, Peng Z, Hao-Yue W, Gang-Xiu C, Zhong-Wen X, Guan-Hu B. Inhibition of alpha-glucosidase and alpha-amylase by flavonoid glycosides from Lu'an GuaPian tea: molecular docking and interaction mechanism. *Food Funct*. 2018;9(8):4173-4183. doi:<https://doi.org/10.1039/c8fo00562a>

203. Sheliya MA, Rayhana B, Ali A, et al. Inhibition of alpha-glucosidase by new prenylated flavonoids from *Euphorbia hirta* L. herb. *J Ethnopharmacol*. 2015;176:1-8. doi:<https://doi.org/10.1016/j.jep.2015.10.018>

204. Pyner A, Nyambe-Silavwe H, Williamson G. Inhibition of human and rat sucrase and maltase activities to assess antiglycemic potential: Optimization of the assay using acarbose and polyphenols. *J Agric Food Chem*. 2017;65(39):8643-8651. doi:<https://doi.org/10.1021/acs.jafc.7b03678>

205. Tuan NN, Thi HN, My CLT, et al. Inhibition of α-glucosidase, acetylcholinesterase, and nitric oxide production by phytochemicals isolated from *Millettia speciosa*—In vitro and molecular docking studies. *Plants*. 2022;11(3):388. doi:<https://doi.org/10.3390/plants11030388>

206. Sun J, Dong S, Wu Y, Zhao H, Li X, Gao W. Inhibitor discovery from pomegranate rind for targeting human salivary alpha-amylase. *Med Chem Res*. 2018;27(6):1559-1577. doi:<https://doi.org/10.1007/s00044-018-2164-2>

207. Luyen NT, Tram LH, Hanh TTH, et al. Inhibitors of a-glucosidase, a-amylase and lipase from *Chrysanthemum morifolium*. *Phytochem Lett*. 2013;6(3):322-325. doi:<https://doi.org/10.1016/j.phytol.2013.03.015>

208. Umamaheswari S, Sangeetha S. Inhibitory action against alpha glucosidase by selected dihydroxy flavones. *Int J Curr Res Rev*. 2019;11:05-08. doi:<https://doi.org/10.31782/IJCRR.2019.0508>

209. Akkarachiyasit S, Charoenlertkul P, Yibchok-Anun S, Adisakwattana S. Inhibitory activities of cyanidin and its glycosides and synergistic effect with acarbose against intestinal α-glucosidase and pancreatic α-amylase. *Int J Mol Sci*. 2010;11(9):3387-96. doi:<https://doi.org/10.3390/ijms11093387>

210. Han L, Song J, Yan C, et al. Inhibitory activity and mechanism of calycosin and calycosin-7-O-β-D-glucoside on α-glucosidase: Spectroscopic and molecular docking analyses. *Process Biochem*. 2022;118:227-235. doi:<https://doi.org/10.1016/j.procbio.2022.04.035>

211. Kim JH, Kim HY, Yang SY, Kim JB, Jin CH, Kim YH. Inhibitory activity of (-)-epicatechin-3,5-O-digallate on alpha-glucosidase and in silico analysis. *Int J Biol Macromol*. 2018;107:1162-1167. doi:<https://doi.org/10.1016/j.ijbiomac.2017.09.091>

212. Xu L, Li W, Chen Z, et al. Inhibitory effect of epigallocatechin-3-O-gallate on alpha-glucosidase and its hypoglycemic effect via targeting PI3K/AKT signaling pathway in L6 skeletal muscle cells. *Int J Biol Macromol*. 2019;125:605-611. doi:<https://doi.org/10.1016/j.ijbiomac.2018.12.064>

213. Sadeghi M, Miroliaei M, Ghanadian M. Inhibitory effect of flavonoid glycosides on digestive enzymes: In silico, in vitro, and in vivo studies. *Int J Biol Macromol*. 2022;217:714-730. doi:<https://doi.org/10.1016/j.ijbiomac.2022.07.086>

214. Li K, Yao F, Xue Q, et al. Inhibitory effects against α-glucosidase and α-amylase of the flavonoids-rich extract from *Scutellaria baicalensis* shoots and interpretation of structure-activity relationship of its eight flavonoids by a refined assign-score method. *Chem Cent J*. 2018;12(1):82. doi:<https://doi.org/10.1186/s13065-018-0445-y>

215. Tundis R, Loizzo MR, Statti GA, Menichini F. Inhibitory effects on the digestive enzyme alpha-amylase of three *Salsola* species (Chenopodiaceae) in vitro. *Pharmazie*. 2007;62(6):473-5. doi:<https://doi.org/10.1691/ph.2007.6.6794>

216. Peng X, Zhang G, Liao Y, Gong D. Inhibitory kinetics and mechanism of kaempferol on alpha-glucosidase. *Food Chem*. 2016;190:207-215. doi:<https://doi.org/10.1016/j.foodchem.2015.05.088>

217. Li Z, Guowen Z, Suyun L, Deming G. Inhibitory mechanism of apigenin on alpha-glucosidase and synergy analysis of flavonoids. *J Agric Food Chem*. 2016;64(37):6939-6949. doi:<https://doi.org/10.1021/acs.jafc.6b02314>

218. Wu X, Hu M, Hu X, Ding H, Gong D, Zhang G. Inhibitory mechanism of epicatechin gallate on α-amylase and α-glucosidase and its combinational effect with acarbose or epigallocatechin gallate. *J Mol Liq*. 2019;290:111202. doi:<https://doi.org/10.1016/j.molliq.2019.111202>

219. Zeng L, Zhang G, Liao Y, Gong D. Inhibitory mechanism of morin on α-glucosidase and its anti-glycation properties. *Food Funct*. 2016;7(9):3953-63. doi:<https://doi.org/10.1039/c6fo00680a>

220. Ni M, Hu X, Gong D, Zhang G. Inhibitory mechanism of vitexin on α-glucosidase and its synergy with acarbose. *Food Hydrocoll*. 2020;105:105824. doi:<https://doi.org/10.1016/j.foodhyd.2020.105824>

221. Dong HQ, Li M, Zhu F, Liu FL, Huang JB. Inhibitory potential of trilobatin from *Lithocarpus polystachyus* Rehd against alpha-glucosidase and alpha-amylase linked to type 2 diabetes. *Food Chem*. 2012;130(2):261-266. doi:<https://doi.org/10.1016/j.foodchem.2011.07.030>

222. Uddin MJ, Faraone I, Haque MA, et al. Insights into the leaves of *Ceriscoides campanulata*: Natural proanthocyanidins alleviate diabetes, inflammation, and esophageal squamous cell cancer via in vitro and in silico models. *Fitoterapia*. 2022;158:105164. doi:<https://doi.org/10.1016/j.fitote.2022.105164>

223. Zhao L, Wen L, Lu Q, Liu R. Interaction mechanism between alpha-glucosidase and A-type trimer procyanidin revealed by integrated spectroscopic analysis techniques. *Int J Biol Macromol*. 2020;143:173-180. doi:<https://doi.org/10.1016/j.ijbiomac.2019.12.021>

224. Imran M, Irfan A, Khalid M, et al. In-vitro and in-silico antioxidant, α-glucosidase inhibitory potentials of abutilins C and D, new flavonoide glycosides from *Abutilon pakistanicum*. *Arab J Chem*. 2021;14(4):103021. doi:<https://doi.org/10.1016/j.arabjc.2021.103021>

225. Ha LM, Luyen NT, Phuong NT, et al. Isoflavonoids from *Desmodium heterophyllum* aerial parts. *Nat Prod Commun*. 2018;13(6):699-700. doi:<https://doi.org/10.1177/1934578x1801300612>

226. Yi J, Zhao T, Zhang Y, et al. Isolated compounds from *Dracaena angustifolia* Roxb and acarbose synergistically/additively inhibit alpha-glucosidase and alpha-amylase: an in vitro study. *BMC Complement Altern Med*. 2022;22(1):177. doi:<https://doi.org/10.1186/s12906-022-03649-3>

227. Ha TJ, Bo Song S, Ko J, et al. Isolation and identification of α-glucosidase inhibitory constituents from the seeds of *Vigna nakashimae*: Enzyme kinetic study with active phytochemical. *Food Chem*. 2018;266:483-489. doi:<https://doi.org/10.1016/j.foodchem.2018.06.039>

228. Costa GM, Cárdenas PA, Gazola AC, et al. Isolation of C-glycosylflavonoids with α-glucosidase inhibitory activity from *Passiflora bogotensis* Benth by gradient high-speed counter-current chromatography. *J Chromatogr B*. 2015;990:104-110. doi:<https://doi.org/10.1016/j.jchromb.2015.03.015>

229. Park MJ, Kang Y-H. Isolation of isocoumarins and flavonoids as α-glucosidase inhibitors from *Agrimonia pilosa* L. *Molecules*. 2020;25(11):2572. doi:<https://doi.org/10.3390/molecules25112572>

230. Marmouzi I, Ezzat SM, Mostafa ES, et al. Isolation of secondary metabolites from the mediterranean sponge species; *Hemimycale columella* and its biological properties. *SN Appl Sci*. 2021;3(2):207. doi:<https://doi.org/10.1007/s42452-020-04052-8>

231. Jiang M-Y, Lu H, Pu X-Y, et al. Laxative metabolites from the leaves of *Moringa oleifera*. *J Agric Food Chem*. 2020;68(30):7850-7860. doi:<https://doi.org/10.1021/acs.jafc.0c01564>

232. Keskes H, Belhadj S, Jlail L, et al. LC-MS-MS and GC-MS analyses of biologically active extracts and fractions from tunisian *Juniperus phoenice* leaves. *Pharm Biol*. 2017;55(1):88-95. doi:<https://doi.org/10.1080/13880209.2016.1230139>

233. Astiti MA, Jittmittraphap A, Leaungwutiwong P, et al. LC-QTOF-MS/MS based molecular networking approach for the isolation of α-glucosidase inhibitors and virucidal agents from *Coccinia grandis* (L.) voigt. *Foods*. 2021;10(12):3041. doi:<https://doi.org/10.3390/foods10123041>

234. Wang Y-L, Zhang L, Li M-Y, Wang L-W, Ma C-M. Lignans, flavonoids and coumarins from *Viola philippica* and their α-glucosidase and HCV protease inhibitory activities. *Nat Prod Res*. 2019;33(11):1550-1555. doi:<https://doi.org/10.1080/14786419.2017.1423305>

235. Wu Q, Min Y, Xiao J, et al. Liquid state fermentation vinegar enriched with catechin as an antiglycative food product. *Food Funct*. 2019;10(8):4877-4887. doi:<https://doi.org/10.1039/c8fo01892h>

236. Matsui T, Kobayashi M, Hayashida S, Matsumoto K. Luteolin, a flavone, does not suppress postprandial glucose absorption through an inhibition of alpha-glucosidase action. *Biosci Biotechnol Biochem*. 2002;66(3):689-692. doi:<https://doi.org/10.1271/bbb.66.689>

237. Djeujo FM, Ragazzi E, Urettini M, et al. Magnolol and luteolin inhibition of alpha-glucosidase activity: Kinetics and type of interaction detected by in vitro and in silico studies. *Pharmaceuticals*. 2022;15(2):205. doi:<https://doi.org/10.3390/ph15020205>

238. Visvanathan R, Houghton MJ, Williamson G. Maltoheptaoside hydrolysis with chromatographic detection and starch hydrolysis with reducing sugar analysis: Comparison of assays allows assessment of the roles of direct α-amylase inhibition and starch complexation. *Food Chem*. 2021;343:128423. doi:<https://doi.org/10.1016/j.foodchem.2020.128423>

239. Kim JH, Kim HY, Jin CH. Mechanistic investigation of anthocyanidin derivatives as alpha-glucosidase inhibitors. *Bioorg Chem*. 2019;87:803-809. doi:<https://doi.org/10.1016/j.bioorg.2019.01.033>

240. Wang X, Yang J, Li H, Shi S, Peng X. Mechanistic study and synergistic effect on inhibition of α-amylase by structurally similar flavonoids. *J Mol Liq*. 2022;360:119485. doi:<https://doi.org/10.1016/j.molliq.2022.119485>

241. Hyun TK, Eom SH, Kim JS. Molecular docking studies for discovery of plant-derived α-glucosidase inhibitors. *Plant OMICS*. 2014;7(3):166-170.

242. Şöhretoğlu D, Bakır SD, Barut B, Šoral M, Sari S. Multiple biological effects of secondary metabolites of *Ziziphus jujuba*: isolation and mechanistic insights through in vitro and in silico studies. *Eur Food Res Technol*. 2022;248(4):1059-1067. doi:<https://doi.org/10.1007/s00217-021-03946-0>

243. Priscilla DH, Roy D, Suresh A, Kumar V, Thirumurugan K. Naringenin inhibits alpha-glucosidase activity: A promising strategy for the regulation of postprandial hyperglycemia in high fat diet fed streptozotocin induced diabetic rats. *Chem Biol Interact*. 2014;210(1):77-85. doi:<https://doi.org/10.1016/j.cbi.2013.12.014>

244. Sun H, Wang D, Song X, et al. Natural prenylchalconaringenins and prenylnaringenins as antidiabetic agents: alpha-glucosidase and alpha-amylase inhibition and in vivo antihyperglycemic and antihyperlipidemic effects. *J Agric Food Chem*. 2017;65(8):1574-1581. doi:<https://doi.org/10.1021/acs.jafc.6b05445>

245. Tran C-L, Tri MD, Tien-Trung N, et al. Nervione, a new benzofuran derivative from *Nervilia concolor*. *Nat Prod Res*. 2022;36(20):5148-5154. doi:<https://doi.org/10.1080/14786419.2021.1920585>

246. Shi ZF, Lei C, Yu BW, Wang HY, Hou AJ. New alkaloids and α-glucosidase inhibitory flavonoids from *Ficus hispida*. *Chem Biodivers*. 2016;13(4):445-50. doi:<https://doi.org/10.1002/cbdv.201500142>

247. Qin NB, Jia CC, Xu J, et al. New amides from seeds of *Silybum marianum* with potential antioxidant and antidiabetic activities. *Fitoterapia*. 2017;119:83-89. doi:<https://doi.org/10.1016/j.fitote.2017.04.008>

248. Li N, Zhu HT, Wang D, Zhang M, Yang CR, Zhang YJ. New flavoalkaloids with potent α-glucosidase and acetylcholinesterase inhibitory activities from Yunnan Black Tea ‘Jin-Ya'. *J Agric Food Chem*. 2020;68(30):7955-7963. doi:<https://doi.org/10.1021/acs.jafc.0c02401>

249. Dat NT, Dang NH, Thanh le N. New flavonoid and pentacyclic triterpene from *Sesamum indicum* leaves. *Nat Prod Res*. 2016;30(3):311-5. doi:<https://doi.org/10.1080/14786419.2015.1057730>

250. Do LTM, Sichaem J. New flavonoid derivatives from *Melodorum fruticosum* and their α-glucosidase inhibitory and cytotoxic activities. *Molecules*. 2022;27(13):4023. doi:<https://doi.org/10.3390/molecules27134023>

251. Janibekov AA, Youssef FS, Ashour ML, Mamadalieva NZ. New flavonoid glycosides from two *Astragalus* species (Fabaceae) and validation of their antihyperglycaemic activity using molecular modelling and in vitro studies. *Ind Crops Prod*. 2018;118:142-148. doi:<https://doi.org/10.1016/j.indcrop.2018.03.034>

252. Ranga Rao R, Tiwari AK, Prabhakar Reddy P, et al. New furanoflavanoids, intestinal alpha-glucosidase inhibitory and free-radical (DPPH) scavenging, activity from antihyperglycemic root extract of *Derris indica* (Lam.). *Bioorg Med Chem*. 2009;17(14):5170-5. doi:<https://doi.org/10.1016/j.bmc.2009.05.051>

253. Zhang L, Tu ZC, Yuan T, et al. New gallotannin and other phytochemicals from Sycamore Maple (*Acer pseudoplatanus*) leaves. *Nat Prod Commun*. 2015;10(11):1977-1980. doi:<https://doi.org/10.1177/1934578x1501001143>

254. Wang M, Yu BW, Yu MH, et al. New isoprenylated phenolic compounds from *Morus laevigata*. *Chem Biodivers*. 2015;12(6):937-45. doi:<https://doi.org/10.1002/cbdv.201400210>

255. He X-F, Chen J-J, Li T-Z, et al. Nineteen new flavanol–fatty alcohol hybrids with α-glucosidase and PTP1B dual Inhibition: one unusual type of antidiabetic constituent from *Amomum tsao-ko*. *J Agric Food Chem*. 2020;68(41):11434-11448. doi:<https://doi.org/10.1021/acs.jafc.0c04615>

256. Ren D, Meng FC, Liu H, et al. Novel biflavonoids from *Cephalotaxus oliveri* Mast. *Phytochem Lett*. 2018;24:150-153. doi:<https://doi.org/10.1016/j.phytol.2018.02.005>

257. Nile A, Gansukh E, Park GS, Kim DH, Hariram Nile S. Novel insights on the multi-functional properties of flavonol glucosides from red onion (*Allium cepa* L) solid waste – In vitro and in silico approach. *Food Chem*. 2021;335:127650. doi:<https://doi.org/10.1016/j.foodchem.2020.127650>

258. Nguyen VB, Wang SL, Nguyen TH, et al. Novel potent hypoglycemic compounds from *Euonymus laxiflorus* Champ. and their effect on reducing plasma glucose in an ICR mouse model. *Molecules*. 2018;23(8):1928. doi:<https://doi.org/10.3390/molecules23081928>

259. Kong F, Ding Z, Zhang K, et al. Optimization of extraction flavonoids from *Exocarpium Citri Grandis* and evaluation its hypoglycemic and hypolipidemic activities. *J Ethnopharmacol*. 2020;262:113178. doi:<https://doi.org/10.1016/j.jep.2020.113178>

260. Zhang K, Ding Z, Duan W, et al. Optimized preparation process for naringenin and evaluation of its antioxidant and α-glucosidase inhibitory activities. *J Food Process Preserv*. 2020;44(12):e14931. doi:<https://doi.org/10.1111/jfpp.14931>

261. Damsud T, Grace MH, Adisakwattana S, Phuwapraisirisan P. Orthosiphol A from the aerial parts of *Orthosiphon aristatus* is putatively responsible for hypoglycemic effect via alpha-glucosidase inhibition. *Nat Prod Commun*. 2014;9(5):639-41. doi:<https://doi.org/10.1177/1934578X1400900512>

262. Xu Y, Xie L, Xie J, Liu Y, Chen W. Pelargonidin-3-O-rutinoside as a novel α-glucosidase inhibitor for improving postprandial hyperglycemia. *Chem Commun (Camb)*. 2018;55(1):39-42. doi:<https://doi.org/10.1039/c8cc07985d>

263. Xu L, Yu M, Niu L, et al. Phenolic compounds isolated from *Morus nigra* and their α-glucosidase inhibitory activities. *Nat Prod Res*. 2020;34(5):605-612. doi:<https://doi.org/10.1080/14786419.2018.1491041>

264. Tan C, Zuo J, Yi X, et al. Phenolic constituents from *Sarcopyramis nepalensis* and their α-glucosidase inhibitory activity. *Afr J Tradit Complement Altern Med*. 2015;12(3):156-160. doi:<https://doi.org/10.4314/ajtcam.v12i3.20>

265. Ho GT, Kase ET, Wangensteen H, Barsett H. Phenolic Elderberry extracts, anthocyanins, procyanidins, and metabolites influence glucose and fatty acid uptake in human skeletal muscle cells. *J Agric Food Chem*. 2017;65(13):2677-2685. doi:<https://doi.org/10.1021/acs.jafc.6b05582>

266. Toh ZS, Wang H, Yip YM, et al. Phenolic group on A-ring is key for dracoflavan B as a selective noncompetitive inhibitor of α-amylase. *Bioorg Med Chem*. 2015;23(24):7641-7649. doi:<https://doi.org/10.1016/j.bmc.2015.11.008>

267. Yang D, Xie H, Jiang Y, Wei X. Phenolics from strawberry cv. Falandi and their antioxidant and α-glucosidase inhibitory activities. *Food Chem*. 2016;194:857-863. doi:<https://doi.org/10.1016/j.foodchem.2015.08.091>

268. Nina N, Theoduloz C, Giménez A, Schmeda-Hirschmann G. Phenolics from the Bolivian highlands food plant *Ombrophytum subterraneum* (Aspl.) B. Hansen (Balanophoraceae): Antioxidant and α-glucosidase inhibitory activity. *Food Res Int*. 2020;137:109382. doi:<https://doi.org/10.1016/j.foodres.2020.109382>

269. Amin A, Tuenter E, Exarchou V, et al. Phytochemical and pharmacological investigations on *Nymphoides indica* leaf extracts. *Phytother Res*. 2016;30(10):1624-1633. doi:<https://doi.org/10.1002/ptr.5663>

270. Sadasivam M, Kumarasamy C, Thangaraj A, et al. Phytochemical constituents from dietary plant *Citrus hystrix*. *Nat Prod Res*. 2018;32(14):1721-1726. doi:<https://doi.org/10.1080/14786419.2017.1399386>

271. Kim TH, Lee J, Kim HJ, Jo C. Plasma-induced degradation of quercetin associated with the enhancement of biological activities. *J Agric Food Chem*. 2017;65(32):6929-6935. doi:<https://doi.org/10.1021/acs.jafc.7b00987>

272. Jeong GH, Kim TH. Plasma-induced oxidation products of (-)-epigallocatechin gallate with digestive enzymes inhibitory effects. *Molecules*. 2021;26(19):5799. doi:<https://doi.org/10.3390/molecules26195799>

273. Li F, Zhan Z, Liu F, et al. Polyflavanostilbene A, a new flavanol-fused stilbene glycoside from *Polygonum cuspidatum*. *Org Lett*. 2013;15(3):674-7. doi:<https://doi.org/10.1021/ol3035033>

274. Xu J, Wang X, Yue J, Sun Y, Zhang X, Zhao Y. Polyphenols from Acorn leaves (*Quercus liaotungensis*) protect pancreatic beta cells and their inhibitory activity against α-glucosidase and protein tyrosine phosphatase 1B. *Molecules*. 2018;23(9):2167. doi:<https://doi.org/10.3390/molecules23092167>

275. Tundis R, Bonesi M, Sicari V, et al. *Poncirus trifoliata* (L.) Raf.: Chemical composition, antioxidant properties and hypoglycaemic activity via the inhibition of α-amylase and α-glucosidase enzymes. *J Funct Foods*. 2016;25:477-485. doi:<https://doi.org/10.1016/j.jff.2016.06.034>

276. Islam MN, Jung HA, Sohn HS, Kim HM, Choi JS. Potent alpha-glucosidase and protein tyrosine phosphatase 1B inhibitors from *Artemisia capillaris*. *Arch Pharm Res*. 2013;36(5):542-552. doi:<https://doi.org/10.1007/s12272-013-0069-7>

277. Mohamed EA, Siddiqui MJ, Ang LF, et al. Potent α-glucosidase and α-amylase inhibitory activities of standardized 50% ethanolic extracts and sinensetin from *Orthosiphon stamineus* Benth as anti-diabetic mechanism. *BMC Complement Altern Med*. 2012;12:176. doi:<https://doi.org/10.1186/1472-6882-12-176>

278. Amin S, Ullah B, Ali M, et al. Potent in vitro α-glucosidase inhibition of secondary metabolites derived from *Dryopteris cycadina*. *Molecules*. 2019;24(3):427. doi:<https://doi.org/10.3390/molecules24030427>

279. Quan YS, Zhang XY, Yin XM, Wang SH, Jin LL. Potential alpha-glucosidase inhibitor from *Hylotelephium erythrostictum*. *Bioorg Med Chem Lett*. 2020;30(24):127665. doi:<https://doi.org/10.1016/j.bmcl.2020.127665>

280. Kim T, Choi HJ, Eom SH, Lee J, Kim TH. Potential alpha-glucosidase inhibitors from thermal transformation of (+)-catechin. *Bioorg Med Chem Lett*. 2014;24(6):1621-1624. doi:<https://doi.org/10.1016/j.bmcl.2014.01.027>

281. Arumugam B, Palanisamy UD, Chua KH, Kuppusamy UR. Potential antihyperglycaemic effect of myricetin derivatives from *Syzygium malaccense*. *J Funct Foods*. 2016;22:325-336. doi:<https://doi.org/10.1016/j.jff.2016.01.038>

282. Kim DH, Jung HA, Sohn HS, Kim JW, Choi JS. Potential of icariin metabolites from *Epimedium koreanum* Nakai as antidiabetic therapeutic agents. *Molecules*. 2017;22(6):986. doi:<https://doi.org/10.3390/molecules22060986>

283. Şöhretoğlu D, Sari S, Šoral M, Barut B, Özel A, Liptaj T. Potential of *Potentilla inclinata* and its polyphenolic compounds in α-glucosidase inhibition: Kinetics and interaction mechanism merged with docking simulations. *Int J Biol Macromol*. 2018;108:81-87. doi:<https://doi.org/10.1016/j.ijbiomac.2017.11.151>

284. Han L, Zhang L, Ma W, Li D, Shi R, Wang M. Proanthocyanidin B(2) attenuates postprandial blood glucose and its inhibitory effect on alpha-glucosidase: analysis by kinetics, fluorescence spectroscopy, atomic force microscopy and molecular docking. *Food Funct*. 2018;9(9):4673-4682. doi:<https://doi.org/10.1039/c8fo00993g>

285. Lin H-C, Lee S-S. Proanthocyanidins from the leaves of *Machilus philippinensis*. *J Nat Prod*. 2010;73(8):1375-1380. doi:<https://doi.org/10.1021/np1002274>

286. Sheikh Y, Chanu MB, Mondal G, et al. Procyanidin A2, an anti-diabetic condensed tannin extracted from *Wendlandia glabrata*, reduces elevated G-6-Pase and mRNA levels in diabetic mice and increases glucose uptake in CC1 hepatocytes and C1C12 myoblast cells. *RSC Adv*. 2019;9(30):17211-17219. doi:<https://doi.org/10.1039/c9ra02397f>

287. Braham N, Phi-Hung N, Bing-Tian Z, Quoc-Hung V, Byung Sun M, Mi Hee W. Protein tyrosine phosphatase 1B (PTP1B) inhibitory activity and glucosidase inhibitory activity of compounds isolated from *Agrimonia pilosa*. *Pharm Biol*. 2016;54(3):474-480. doi:<https://doi.org/10.3109/13880209.2015.1048372>

288. Seong SH, Roy A, Jung HA, Jung HJ, Choi JS. Protein tyrosine phosphatase 1B and alpha-glucosidase inhibitory activities of *Pueraria lobata* root and its constituents. *J Ethnopharmacol*. 2016;194:706-716. doi:<https://doi.org/10.1016/j.jep.2016.10.007>

289. Jung HA, Ali MY, Bhakta HK, Min BS, Choi JS. Prunin is a highly potent flavonoid from *Prunus davidiana* stems that inhibits protein tyrosine phosphatase 1B and stimulates glucose uptake in insulin-resistant HepG2 cells. *Arch Pharm Res*. 2017;40(1):37-48. doi:<https://doi.org/10.1007/s12272-016-0852-3>

290. Zhao BT, Duc Dat L, Phi Hung N, et al. PTP1B, alpha-glucosidase, and DPP-IV inhibitory effects for chromene derivatives from the leaves of *Smilax china* L. *Chem Biol Interact*. 2016;253:27-37. doi:<https://doi.org/10.1016/j.cbi.2016.04.012>

291. Yan S, Zhang X, Wen X, et al. Purification of flavonoids from Chinese Bayberry (*Morella rubra* Sieb. et Zucc.) fruit extracts and alpha-glucosidase inhibitory activities of different fractionations. *Molecules*. 2016;21(9):1148. doi:<https://doi.org/10.3390/molecules21091148>

292. Zhao Y, Kongstad KT, Jager AK, Nielsen J, Staerk D. Quadruple high-resolution alpha-glucosidase/alpha-amylase/PTP1B/radical scavenging profiling combined with high-performance liquid chromatography-high-resolution mass spectrometry-solid-phase extraction-nuclear magnetic resonance spectroscopy for identification of antidiabetic constituents in crude root bark of *Morus alba* L. *J Chromatogr A*. 2018;1556:55-63. doi:<https://doi.org/10.1016/j.chroma.2018.04.041>

293. Gou SH, Liu J, He M, Qiang Y, Ni JM. Quantification and bio-assay of α-glucosidase inhibitors from the roots of *Glycyrrhiza uralensis* Fisch. *Nat Prod Res*. 2016;30(18):2130-4. doi:<https://doi.org/10.1080/14786419.2015.1114940>

294. Yang H, Chan AL, LaVallo V, Cheng Q. Quantitation of alpha-glucosidase activity using fluorinated carbohydrate array and MALDI-TOF-MS. *ACS Appl Mater Interfaces*. 2016;8(4):2872-2878. doi:<https://doi.org/10.1021/acsami.5b12518>

295. Qu X, Li J, Yan P, et al. Quercetin of *Potentilla bifurca* 3-glycosylation substitution impact the inhibitory activity on alpha-glucosidase. *Pharmacogn Mag*. 2022;18(78):458-462. doi:<https://doi.org/10.4103/pm.pm_522_21>

296. Wang L, Liu Y, Luo Y, Huang K, Wu Z. Quickly screening for potential α-glucosidase Inhibitors from Guava leaves tea by bioaffinity ultrafiltration coupled with HPLC-ESI-TOF/MS Method. *J Agric Food Chem*. 2018;66(6):1576-1582. doi:<https://doi.org/10.1021/acs.jafc.7b05280>

297. Lianwu X, Qiachi F, Shuyun S, Jiawei L, Xinji Z. Rapid and comprehensive profiling of alpha-glucosidase inhibitors in Buddleja Flos by ultrafiltration HPLC-QTOF-MS/MS with diagnostic ions filtering strategy. *Food Chem*. 2021;344:128651-128651. doi:<https://doi.org/10.1016/j.foodchem.2020.128651>

298. Yang Y, Gu L, Xiao Y, et al. Rapid identification of α-glucosidase inhibitors from *Phlomis tuberosa* by sepbox chromatography and thin-layer chromatography bioautography. *PLoS One*. 2015;10(2):e0116922. doi:<https://doi.org/10.1371/journal.pone.0116922>

299. Li Y, Chen Y, Xiao C, Chen D, Xiao Y, Mei Z. Rapid screening and identification of α-amylase inhibitors from *Garcinia xanthochymus* using enzyme-immobilized magnetic nanoparticles coupled with HPLC and MS. *J Chromatogr B Analyt Technol Biomed Life Sci*. 2014;960:166-73. doi:<https://doi.org/10.1016/j.jchromb.2014.04.041>

300. Tao Y, Zhang Y, Cheng Y, Wang Y. Rapid screening and identification of α-glucosidase inhibitors from mulberry leaves using enzyme-immobilized magnetic beads coupled with HPLC/MS and NMR. *Biomed Chromatogr*. 2013;27(2):148-55. doi:<https://doi.org/10.1002/bmc.2761>

301. Li H, Song F, Xing J, Tsao R, Liu Z, Liu S. Screening and structural characterization of α-glucosidase Inhibitors from Hawthorn leaf flavonoids extract by ultrafiltration LC-DAD-MSn and SORI-CID FTICR MS. *J Am Soc Mass Spectrom*. 2009;20(8):1496-1503. doi:<https://doi.org/10.1016/j.jasms.2009.04.003>

302. Jiang W, Kan H, Li P, Liu S, Liu Z. Screening and structural characterization of potential α-glucosidase inhibitors from *Radix Astragali* flavonoids extract by ultrafiltration LC-DAD-ESI-MSn. *Anal Methods*. 2015;7(1):123-128. doi:<https://doi.org/10.1039/c4ay02081b>

303. Nguyen MTT, Nguyen NT, Nguyen HX, Huynh TNN, Min BS. Screening of alpha-glucosidase inhibitory activity of Vietnamese medicinal plants: Isolation of active principles from *Oroxylum indicum*. *Nat Prod Sci*. 2012;18(1):47-51.

304. Wu B, Song HP, Zhou X, et al. Screening of minor bioactive compounds from herbal medicines by in silico docking and the trace peak exposure methods. *J Chromatogr A*. 2016;1436:91-99. doi:<https://doi.org/10.1016/j.chroma.2016.01.062>

305. Wang YM, Zhao JQ, Yang JL, Tao YD, Mei LJ, Shi YP. Separation of antioxidant and alpha-glucosidase inhibitory flavonoids from the aerial parts of *Asterothamnus centrali-asiaticus*. *Nat Prod Res*. 2017;31(12):1365-1369. doi:<https://doi.org/10.1080/14786419.2016.1247083>

306. Yu Z, Jian-Nan MA, Chun-Li MA, Zhi QI, Chao-Mei MA. Simultaneous quantification of ten constituents of *Xanthoceras sorbifolia* Bunge using UHPLC-MS methods and evaluation of their radical scavenging, DNA scission protective, and α-glucosidase inhibitory activities. *Chin J Nat Med*. 2015;13(11):873-880. doi:<https://doi.org/10.3724/SP.J.1009.2015.00873>

307. Xiao Z, Hou X, Zhang T, et al. Starch-digesting product analysis based on the hydrophilic interaction liquid chromatography coupled mass spectrometry method to evaluate the inhibition of flavonoids on pancreatic α-amylase. *Food Chem*. 2022;372:131175. doi:<https://doi.org/10.1016/j.foodchem.2021.131175>

308. Zhang H, Yerigui, Yang Y, Ma C. Structures and antioxidant and intestinal disaccharidase inhibitory activities of A-type proanthocyanidins from peanut skin. *J Agric Food Chem*. 2013;61(37):8814-20. doi:<https://doi.org/10.1021/jf402518k>

309. Fan YH, Ye R, Xu HY, Feng XH, Ma CM. Structures and in vitro antihepatic fibrosis activities of prenylated dihydrostilbenes and flavonoids from *Glycyrrhiza uralensis* leaves. *J Food Sci*. 2019;84(5):1224-1230. doi:<https://doi.org/10.1111/1750-3841.14592>

310. Ajish KR, Antu KA, Riya MP, et al. Studies on alpha-glucosidase, aldose reductase and glycation inhibitory properties of sesquiterpenes and flavonoids of *Zingiber zerumbet* Smith. *Nat Prod Res*. 2015;29(10):947-952. doi:<https://doi.org/10.1080/14786419.2014.956741>

311. Boonsombat J, Prachyawarakorn V, Pansanit A, Mahidol C, Ruchirawat S, Thongnest S. Superbanone, a new 2-aryl-3-benzofuranone and other bioactive constituents from the tube roots of *Butea superba*. *Chem Biodivers*. 2017;14(7):e1700044. doi:<https://doi.org/10.1002/cbdv.201700044>

312. Sun H, Song X, Tao Y, et al. Synthesis & α-glucosidase inhibitory & glucose consumption-promoting activities of flavonoid–coumarin hybrids. *Future Med Chem*. 2018;10(9):1055-1066. doi:<https://doi.org/10.4155/fmc-2017-0293>

313. Hari Babu T, Rama Subba Rao V, Tiwari AK, et al. Synthesis and biological evaluation of novel 8-aminomethylated oroxylin A analogues as alpha-glucosidase inhibitors. *Bioorg Med Chem Lett*. 2008;18(5):1659-62. doi:<https://doi.org/10.1016/j.bmcl.2008.01.055>

314. Kumar GS, Tiwari AK, Rao VRS, Prasad KR, Ali AZ, Babu KS. Synthesis and biological evaluation of novel benzyl-substituted flavones as free radical (DPPH) scavengers and-glucosidase inhibitors. *J Asian Nat Prod Res*. 2010;12(11):978-984. doi:<https://doi.org/10.1080/10286020.2010.511190>

315. Cheng N, Yi WB, Wang QQ, Peng SM, Zou XQ. Synthesis and α-glucosidase inhibitory activity of chrysin, diosmetin, apigenin, and luteolin derivatives. *Chin Chem Lett*. 2014;25(7):1094-1098. doi:<https://doi.org/10.1016/j.cclet.2014.05.021>

316. Tang C, Zhu L, Li J, et al. Synthesis and structure elucidation of five new conjugates of oleanolic acid derivatives and chalcones using 1D and 2D NMR spectroscopy. *Magn Reson Chem*. 2012;50(3):236-41. doi:<https://doi.org/10.1002/mrc.2845>

317. Saleem F, Kanwal, Khan KM, et al. Synthesis of azachalcones, their α-amylase, α-glucosidase inhibitory activities, kinetics, and molecular docking studies. *Bioorg Chem*. 2021;106:104489. doi:<https://doi.org/10.1016/j.bioorg.2020.104489>

318. Saidi I, Manachou M, Znati M, Bouajila J, Ben Jannet H. Synthesis of new halogenated flavonoid-based isoxazoles: in vitro and in silico evaluation of a-amylase inhibitory potential, a SAR analysis and DFT studies. *J Mol Struct*. 2022;1247:131379. doi:<https://doi.org/10.1016/j.molstruc.2021.131379>

319. Imran S, Taha M, Ismail NH, et al. Synthesis of novel flavone hydrazones: In-vitro evaluation of α-glucosidase inhibition, QSAR analysis and docking studies. *Eur J Med Chem*. 2015;105:156-170. doi:<https://doi.org/10.1016/j.ejmech.2015.10.017>

320. Zhang Y, Wang H, Wu Y, et al. Synthesis of rottlerone analogues and evaluation of their alpha-glucosidase and DPP-4 dual Inhibitory and glucose consumption-promoting activity. *Molecules*. 2021;26(4):1024. doi:<https://doi.org/10.3390/molecules26041024>

321. Liu J, Lu JF, Kan J, Wen XY, Jin CH. Synthesis, characterization and in vitro anti-diabetic activity of catechin grafted inulin. *Int J Biol Macromol*. 2014;64:76-83. doi:<https://doi.org/10.1016/j.ijbiomac.2013.11.028>

322. Chinthala Y, Thakur S, Tirunagari S, et al. Synthesis, docking and ADMET studies of novel chalcone triazoles for anti-cancer and anti-diabetic activity. *Eur J Med Chem*. 2015;93:564-573. doi:<https://doi.org/10.1016/j.ejmech.2015.02.027>

323. Sun H, Li Y, Zhang X, et al. Synthesis, α-glucosidase inhibitory and molecular docking studies of prenylated and geranylated flavones, isoflavones and chalcones. *Bioorg Med Chem Lett*. 2015;25(20):4567-71. doi:<https://doi.org/10.1016/j.bmcl.2015.08.059>

324. Imran S, Taha M, Ismail NH, et al. Synthesis, in vitro and docking studies of new flavone ethers as α-glucosidase inhibitors. *Chem Biol Drug Des*. 2016;87(3):361-73. doi:<https://doi.org/10.1111/cbdd.12666>

325. Mphahlele MJ, Agbo EN, Choong YS. Synthesis, structure, carbohydrate enzyme inhibition, antioxidant activity, in silico drug-receptor interactions and drug-like profiling of the 5-styryl-2-aminochalcone Hybrids. *Molecules*. 2021;26(9):2692. doi:<https://doi.org/10.3390/molecules26092692>

326. Wu C, Shen J, He P, et al. The alpha-glucosidase inhibiting isoflavones isolated from *Belamcanda chinensis* leaf extract. *Rec Nat Prod*. 2012;6(2):110-120.

327. He XF, Chen JJ, Huang XY, et al. The antidiabetic potency of *Amomum tsao-ko* and its active flavanols, as PTP1B selective and α-glucosidase dual inhibitors. *Ind Crops Prod*. 2021;160:112908. doi:<https://doi.org/10.1016/j.indcrop.2020.112908>

328. Habtemariam S. The anti-obesity potential of sigmoidin A. *Pharm Biol*. 2012;50(12):1519-1522. doi:<https://doi.org/10.3109/13880209.2012.688838>

329. Taslimi P, Caglayan C, Gulcin I. The impact of some natural phenolic compounds on carbonic anhydrase, acetylcholinesterase, butyrylcholinesterase, and alpha-glycosidase enzymes: An antidiabetic, anticholinergic, and antiepileptic study. *J Biochem Mol Toxicol*. 2017;31(12):e21995. doi:<https://doi.org/10.1002/jbt.21995>

330. Zhang LL, Han L, Yang SY, Meng XM, Ma WF, Wang M. The mechanism of interactions between flavan-3-ols against a-glucosidase and their in vivo antihyperglycemic effects. *Bioorg Chem*. 2019;85:364-372. doi:<https://doi.org/10.1016/j.bioorg.2018.12.037>

331. Chen Y, Ye X, Wang L, et al. Three flavanols delay starch digestion by inhibiting alpha-amylase and binding with starch. *Int J Biol Macromol*. 2021;172:503-514. doi:<https://doi.org/10.1016/j.ijbiomac.2021.01.070>

332. Mai NTT, Hai NX, Phu DH, Trong PNH, Nhan NT. Three new geranyl aurones from the leaves of *Artocarpus altilis*. *Phytochem Lett*. 2012;5(3):647-650. doi:<https://doi.org/10.1016/j.phytol.2012.06.014>

333. Yuca H, Özbek H, Demirezer LÖ, Kasil HG, Güvenalp Z. Trans-tiliroside: A potent α-glucosidase inhibitor from the leaves of *Elaeagnus angustifolia* L. *Phytochemistry*. 2021;188:112795. doi:<https://doi.org/10.1016/j.phytochem.2021.112795>

334. Algethami FK, Saidi I, Abdelhamid HN, et al. Trifluoromethylated flavonoid-based isoxazoles as antidiabetic and anti-obesity agents: synthesis, in vitro α-amylase inhibitory activity, molecular docking and structure–activity relationship analysis. *Molecules*. 2021;26(17):5214. doi:<https://doi.org/10.3390/molecules26175214>

335. Srisurichan S, Pornpakakul S. Triterpenoids from the seedpods of *Holarrhena curtisii* King and Gamble. *Phytochem Lett*. 2015;12:282-286. doi:<https://doi.org/10.1016/j.phytol.2015.04.013>

336. Liu Y, Wang R, Ren C, et al. Two myricetin-derived flavonols from *Morella rubra* leaves as potent alpha-glucosidase inhibitors and structure-activity relationship study by computational chemistry. *Oxid Med Cell Longev*. 2022;2022:9012943. doi:<https://doi.org/10.1155/2022/9012943>

337. Fei W-T, Zhang J-J, Tang R-Y, Yue N, Zhou X, Wang L-Y. Two new prenylated flavonoids from the seeds of *Psoralea corylifolia* with their inhibitory activity on α-glucosidase. *Phytochem Lett*. 2020;39:64-67. doi:<https://doi.org/10.1016/j.phytol.2020.07.005>

338. El-Hawary SS, Mubarek MM, Lotfy RA, Hassan AR, Sobeh M, Okba MM. Validation of antidiabetic potential of *Gymnocarpos decandrus* Forssk. *Nat Prod Res*. 2021;35(24):5954-5959. doi:<https://doi.org/10.1080/14786419.2020.1805608>

339. Islam MN, Ishita IJ, Jung HA, Choi JS. Vicenin 2 isolated from *Artemisia capillaris* exhibited potent anti-glycation properties. *Food Chem Toxicol*. 2014;69:55-62. doi:<https://doi.org/10.1016/j.fct.2014.03.042>

340. Luyen NT, Binh PT, Tham PT, et al. Wedtrilosides A and B, two new diterpenoid glycosides from the leaves of *Wedelia trilobata* (L.) Hitchc. with alpha-amylase and alpha-glucosidase inhibitory activities. *Bioorg Chem*. 2019;85:319-324. doi:<https://doi.org/10.1016/j.bioorg.2019.01.010>

341. Liu M, Yin H, Liu G, Dong J, Qian Z, Miao J. Xanthohumol, a prenylated chalcone from beer hops, acts as an α-glucosidase inhibitor in vitro. *J Agric Food Chem*. 2014;62(24):5548-54. doi:<https://doi.org/10.1021/jf500426z>

342. Tan C, Wang Q, Luo C, Chen S, Li Q, Li P. Yeast alpha-glucosidase inhibitory phenolic compounds isolated from *Gynura medica* leaf. *Int J Mol Sci*. 2013;14(2):2551-2558. doi:<https://doi.org/10.3390/ijms14022551>

343. Gong T, Yang X, Bai F, et al. Young apple polyphenols as natural α-glucosidase inhibitors: In vitro and in silico studies. *Bioorg Chem*. 2020;96:103625. doi:<https://doi.org/10.1016/j.bioorg.2020.103625>

344. Proença C, Freitas M, Ribeiro D, et al. Evaluation of a flavonoids library for inhibition of pancreatic α-amylase towards a structure–activity relationship. *J Enzyme Inhib Med Chem*. 2019;34(1):577-588. doi:<https://doi.org/10.1080/14756366.2018.1558221>

345. Williams LK, Li C, Withers SG, Brayer GD. Order and disorder: differential structural impacts of myricetin and ethyl caffeate on human amylase, an antidiabetic target. *J Med Chem*. 2012;55(22):10177-10186. doi:<https://doi.org/10.1021/jm301273u>
